# Unusual tandem expansion and positive selection in subgroups of the plant GRAS transcription factor superfamily

**DOI:** 10.1186/s12870-014-0373-5

**Published:** 2014-12-19

**Authors:** Ningning Wu, Yan Zhu, Wanlu Song, Yaxuan Li, Yueming Yan, Yingkao Hu

**Affiliations:** College of Life Sciences, Capital Normal University, Beijing, 100048 China

**Keywords:** ᅟ

## Abstract

**Background:**

GRAS proteins belong to a plant transcription factor family that is involved with multifarious roles in plants. Although previous studies of this protein family have been reported for *Arabidopsis*, rice, Chinese cabbage and other species, investigation of expansion patterns and evolutionary rate on the basis of comparative genomics in different species remains inadequate.

**Results:**

A total of 289 GRAS genes were identified in *Arabidopsis*, *B. distachyon*, rice, soybean, *S. moellendorffii*, and *P. paten*s and were grouped into seven subfamilies, supported by the similarity of their exon–intron patterns and structural motifs. All of tandem duplicated genes were found in group II except one cluster of rice, indicating that tandem duplication greatly promoted the expansion of group II. Furthermore, segment duplications were mainly found in the soybean genome, whereas no single expansion pattern dominated in other plant species indicating that GRAS genes from these five species might be subject to a more complex evolutionary mechanism. Interestingly, branch-site model analyses of positive selection showed that a number of sites were positively selected under foreground branches I and V. These results strongly indicated that these groups were experiencing higher positive selection pressure. Meanwhile, the site-specific model revealed that the GRAS genes were under strong positive selection in *P. paten*s. DIVERGE v2.0 was used to detect critical amino acid sites, and the results showed that the shifted evolutionary rate was mainly attributed to the functional divergence between the GRAS genes in the two groups. In addition, the results also demonstrated the expression divergence of the GRAS duplicated genes in the evolution. In short, the results above provide a solid foundation for further functional dissection of the GRAS gene superfamily.

**Conclusions:**

In this work, differential expression, evolutionary rate, and expansion patterns of the GRAS gene family in the six species were predicted. Especially, tandem duplication events played an important role in expansion of group II. Together, these results contribute to further functional analysis and the molecular evolution of the GRAS gene superfamily.

**Electronic supplementary material:**

The online version of this article (doi:10.1186/s12870-014-0373-5) contains supplementary material, which is available to authorized users.

## Background

Transcriptional regulation of gene expression is the one of the most important regulatory mechanisms in plants. Transcription factors mediate transcriptional regulation in response to developmental and environmental changes. Generally, transcription factors can be grouped into specific families on the basis of their shared structural characteristics. GRAS proteins belong to a plant family of transcription factors and are named for the three founding members: Gibberellic Acid Insensitive (*GAI*), Repressor of Ga1 (*RGA*), and Scarecrow (*SCR*) [1-5]. Recently, GRAS proteins were also identified in bacterial [6]. Typically, GRAS proteins are 400–700 amino acids in length. They share a variable N-terminus and a highly conserved C-terminus that contains five recognizable motifs, found in the following order: leucine heptad repeat I (LHR I), VHIID, leucine heptad repeat II (LHR II), PFYRE, and SAW [7]. Among these, the PFYRE motif consists of three units: P, FY, and RE and the SAW motif is characterized by three pairs of conserved residues: R-E, W-G, and W-W [5]. Significantly, the VHIID, PFYRE, and SAW domains act as repression domains in SLR1 protein [8]. The distinguishing domains of GRAS proteins are two leucine-rich areas flanking a VHIID motif, which may act as a DNA-binding domain, analogous to the bZIP protein–DNA interaction domain [4]. Moreover, most GRAS proteins are nuclear localized except the PAT1 and SCL13, which are dual-localized to cytoplasm and nucleus [9].

As transcription factors, GRAS proteins have been shown to play critical roles in many specific biological processes related to gibberellin signal transduction [3,10,11], axillary meristem initiation [12-14], shoot meristem maintenance [15], root radial pattering [1,16], phytochrome A signal transduction [9], and male gametogenesis [17]. For example, in *Arabidopsis*, five DELLA proteins—*GAI*, *RGA*, *RGL1*, *RGL2*, and *RGL3*—act as repressors of gibberellin-responsive plant growth. In rice, *OsMOCI* has been demonstrated to control tillering [14]. In petunia, *PhHAM* is essential for maintaining the shoot apical meristem [15]. Recently, thanks to the development of bioinformatics and novel molecular biology techniques, comprehensive expression analyses have been carried out by reverse transcription-PCR (RT-PCR), cDNA or oligo microarray, and cDNA real-time PCR at the genome-wide level. These analyses contribute to our understanding of the function of the GRAS family [18].

After the first member of GRAS protein, *Scarecrow*, being isolated from *Arabidopsis* [1], GRAS proteins in different taxonomic groups have been identified, including tomato, petunia, lily, rice, grape, pine, maize, and barley. A great diversity of GRAS genes exists, depending on the species. So far, various in silico analysis have predicted 33, 60, and 48 GRAS genes in *Arabidopsis*, rice, and Chinese cabbage [7,19], respectively. Meanwhile, the rapid development of large-scale genome sequencing and comparative genomics would likely lead to the discovery of GRAS proteins in other plants. Although great diversity exists among species in terms of genome size, ploidy level and chromosome numbers, attempts have been made to reveal the existing synteny and colinearity on the basis of comparative genomics.

The recently completed sequencing and assembly work provide an opportunity to better understand the evolution of the GRAS superfamily at the whole-genome level. In present work, we identified GRAS gene families in six plant species: *Arabidopsis*, *B. distachyon*, rice, soybean, *S. moellendorffii*, and *P. patens*. Then we constructed a phylogenetic tree to evaluate evolutionary relationships among the GRAS genes in the six plant species and calculated the synonymous substitution rates (Ks) to date the duplication events. Then, we analyzed the expression profiles of GRAS genes in different tissues, which indicated broad functional divergence within this family. To examine the driving force for the evolution of function, we further analyzed functional divergence and adaptive evolution at the amino acid level. Our systematic analysis provided a solid foundation for further functional dissection and molecular evolution of GRAS genes in plants.

## Results

### Genome-wide identification of GRAS gene family

In silico analyses have predicted that 33, 44, 47, 106, 21, and 38 GRAS genes exist in *Arabidopsis*, *B. distachyon*, rice, soybean, *S. moellendorffii*, and *P. paten*s, respectively (Additional files 1 and 2). The names of the GRAS genes, the locus gene, the chromosome and location, the length of the amino acid sequence, the isoelectric point (pI), and the molecular weight (Mw) were supplied in Additional files 3, 4, 5, 6, 7 and 8. Most of the deduced GRAS amino acid sequence lengths varied from 400 to 700 amino acids, while more than half of proteins from *P. patens* contained more than 700 amino acids. The pI of the majority of GRAS proteins varied from 4.68 to 6.92 (faintly acidic), and a minority of GRAS proteins were alkalescent. Of all the GRAS proteins, those from *Arabidopsis* and *P. patens* were all faintly acid, whereas the highest pI of the GRAS proteins, 9.57, was found in *B. distachyon*. The Mw of all GRAS proteins ranged from 39.2 kD to 111.4 kD. These results implied that the amino acid sequence length and physicochemical properties of GRAS proteins may have changed to meet different functions.

All GRAS proteins were mapped onto the corresponding chromosomes except *S. moellendorffii* and *P. patens* (Additional file 9). In *Arabidopsis*, the predicted 33 AtGRAS (*Arabidopsis thaliana* GRAS protein) genes were distributed among the five chromosomes. Chromosomes 1 and 3 had a maximum of nine and seven AtGRAS genes, respectively, whereas six AtGRAS genes were found on each of chromosomes 2 and 5. In *B. distachyon*, the predicted 44 BdGRAS (*B. distachyon* GRAS protein) genes were also distributed among the five chromosomes. Chromosomes 1 and 4 had a maximum of 17 and 14 BdGRAS genes, respectively, while chromosome 5 had a minimum of two BdGRAS genes. In rice, the putative 47 OsGRAS (*Oryza sativa* GRAS protein) genes were organized on 10 out of the 12 chromosomes. Chromosome 11 had a maximum of nine OsGRAS genes, while chromosome 10 had a minimum of two OsGRAS genes. Chromosomes 1, 5, and 7 contained five OsGRAS genes each, and chromosomes 2, 4, and 12 contained four OsGRAS genes each. In soybean, the 106 GmGRAS (*Glycine max* GRAS protein) genes were dispersed on the 20 chromosomes, with 14 members, the highest density of GmGRAS genes, on chromosome 11. Five GmGRAS genes were found on each of chromosomes 1, 2, 5, 9, 10, 16, 17, and 18, four each on chromosomes 3, 4, 6, and 7, and three each on chromosomes 8, 14, and 20.

### Phylogenetic relationships among GRAS proteins

**Figure 1:**
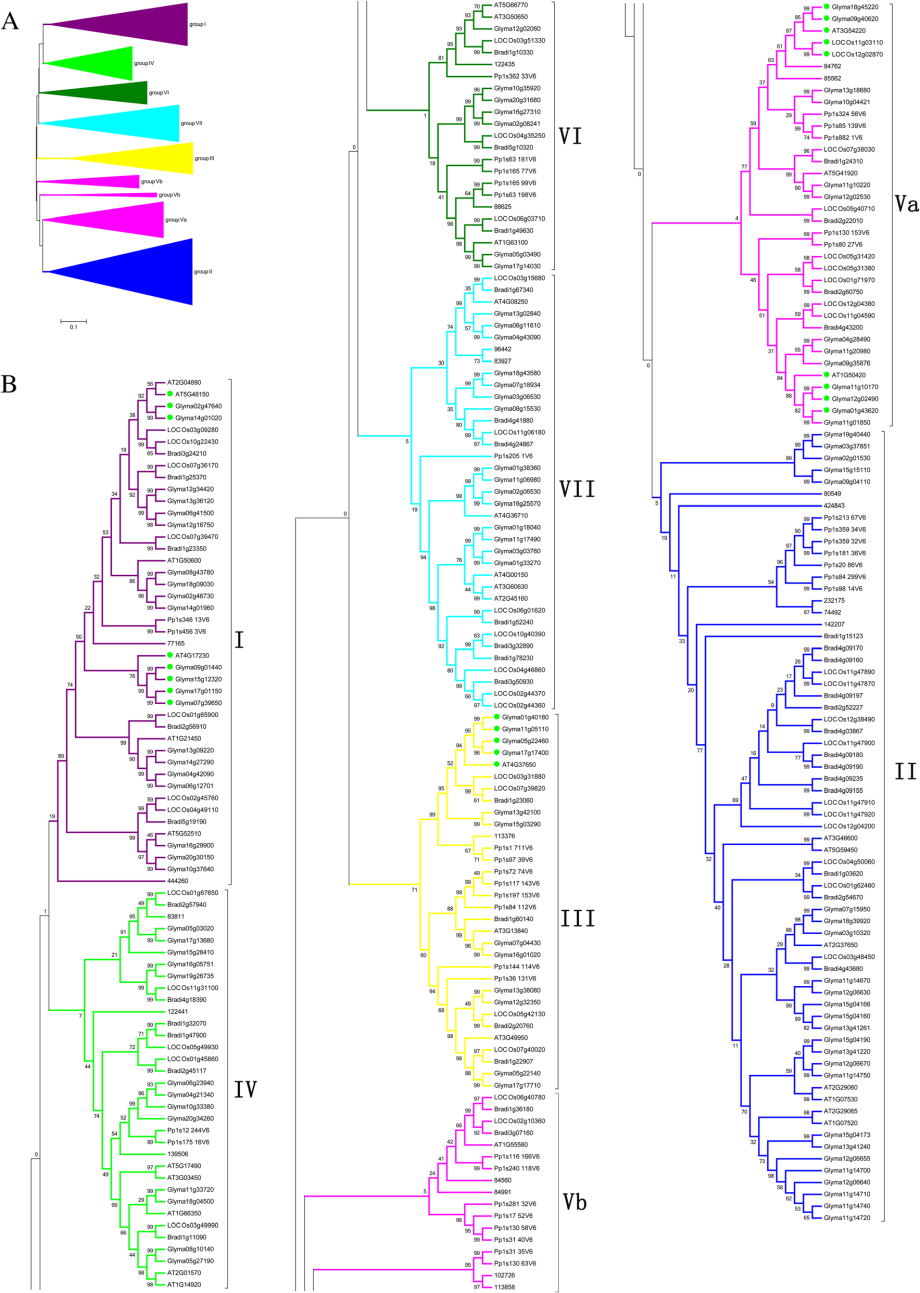
**Phylogenetic tree of GRAS proteins among** ***Arabidopsis*** **,** ***Brachypodium distachyon*** **, rice, soybean,** ***Physcomitrella patens*** **, and** ***Selaginella moellendorffii*** **. A)** The major clusters of orthologous genes are shown in different colors: group I = purple, group II = dark blue, group III = yellow, group IV = light green, group V = pink, group VI = dark green, and group VII = light blue. The scale bar corresponds to 0.1 estimated amino acid substitutions per site; **B)** Genes belonging to the different groups are shown. Among them, the deduced DELLA proteins are indicated by a filled red square, and genes with similar functions clustered together are indicated by filled green circles.

Comparison of conserved motifs among members of the GRAS family implied that they can be divided into different groups and subgroups. To better separate the groups and investigate the evolutionary relationships among GRAS proteins in *Arabidopsis*, *B. distachyon*, rice, soybean, *S. moellendorffii*, and *P. patens*, an unrooted phylogenetic tree was constructed from 289 full-length amino acid sequences using the neighbor-joining (NJ) algorithm (Figure 1 and Additional file 10). To confirm the tree topologies, a ML (maximum likelihood) phylogenetic tree was also constructed, and it showed similar topology to the NJ tree with only minor modifications (Additional file 11). A ME (Minimum-Evolution) phylogenetic tree was also constructed, which showed the same topology to the NJ tree (Additional file 12). Although the NJ tree was usually the same as the ME tree, when the number of taxa was small the difference between the NJ and ME trees can be substantial [20]. In this case if a long DNA or amino acid sequence was used, the ME tree was preferable. When the number of nucleotides or amino acids used was relatively small, the NJ method generated the correct topology more often than did the ME method [21,22]. In this study, the average amino acid-length of 289 GRAS proteins was ~580, so the ME tree was credible. Taken together, the NJ phylogenetic tree was adopted for further analysis. Based on the information from previous analyses and from the topology of the tree and position of conserved motifs, we grouped all the GRAS genes into seven major clusters, group I–VII [7,18]. Group V was further divided into two subgroups, Va and Vb. The numbers of GRAS proteins in different groups were shown in Additional file 1. Among the groups, group II constituted the largest clade. It contained 67 members and accounted for 23.2% of the total GRAS genes. Meanwhile, the number of group II genes from angiosperm also reached the maximum in comparison with the other subgroups, which strongly indicates that these GRAS genes were more likely to be retained in group II. On the contrary, the members of *S. moellendorffii* and *P. paten*s more gathered in group V. Moreover, the identified DELLA proteins: *GAI*, *RGA*, *RGL1*, *RGL2*, *RGL3*, and *SLR1* (*LOC_Os03g49990*) were all present in group IV [8,18]. We also deduced twelve DELLA proteins (*Bradi1g11090*, *Glyma10g33380*, *Glyma08g10140*, *Glyma06g23940*, *Glyma04g21340*, *Glyma05g27190*, *Glyma11g33720*, *Glyma18g04500*, *139506*, *122441*, *Pp1s12_244V6*, and *Pp1s175_16V6*) on the basis of the feature that DELLA proteins contain conserved DELLA and VHYNP motifs in their N-terminal regions and belong to group IV. Moreover, the tree (Figure 1) also showed many putative orthologs (e.g., *Bradi4g03867*/*LOC_Os12g38490*, *Bradi4g43680*/*LOC_Os03g48450*) supported by the high bootstrap values.

The comparative analyses of the complete amino acid sequences of the GRAS proteins were in agreement with the presented phylogenetic analysis, and showed that several family- and subfamily-specific conserved motifs could be determined for each of the defined groups. GRAS proteins share a highly conserved C-terminal region containing the VHIID motif flanked by two leucine heptad repeats (LHRI and LHRII), then the PFYRE motif, and finally the SAW motif. The feature of five motifs has been reported many times in previous studies [4,5,23]. For example, LHR I and LHR II appear to consist of two repeat units (A and B). The VHIID motif is readily recognizable in all members because of its P-N-H-D-Q-L residues. Significantly, our results were quite similar to their statements, and the multiple sequence alignment of the six plant species’ GRAS domains were listed in Additional files 13 and 14. In short, a large number of C-terminal homologies exist between GRAS proteins, suggesting that these conserved residues were required to enable the activity of the GRAS gene products. In addition, a MEME search for conserved protein motifs outside the GRAS domain was conducted to determine possible mechanisms for the structural evolution of GARS genes. As a few SmGRAS (*S. moellendorffii* GRAS protein) and PpGRAS (*P.* patens GRAS protein) genes shared the same motif with the four other species, only the motif data of angiosperms were presented in Additional file 15. Among them, five motif components (motifs 1, 2, 3, 5, and 6) were only detected in group II. Interestingly, motif 5 was found only in monocots (*B. distachyon* and rice), suggesting that these genes diverged after the monocot–dicot split. DELLA proteins shared the same two motif components (the DELLA and VHYNP motifs) in group IV, which was significantly different from the other groups. Most of the members in group I contained motif 4. A schematic diagram of the GRAS protein motifs was shown in Additional file 16. In short, the differences of motif distribution in different groups or subgroups of GRAS genes revealed that the function of the GRAS genes may have diverged in the evolution.

The intron distribution can also provide important evidence to support phylogenetic relationships within a gene family. To identify the gene structure evolution of GRAS proteins, Gene Structure Display Server analysis was applied to 289 GRAS genes. The putative gene structure of the predicted GRAS gene family was shown in Additional files 3, 4, 5, 6, 7 and 8. Of the 289 GRAS genes, 53 had introns and 236 had no introns. Among these, *LOC_Os10g40390* seemed to have a complex gene structure with nine introns. In short, a majority of GRAS genes from angiosperm and *S. moellendorffii* (243 of 251; 96.8%) either lacked introns or had only a single intron, which suggests that these GRAS genes were conserved. However, the GRAS genes from *P. patens* were quite different from those of other species, 36.8% (14 of 38) genes had more than one intron, including three PpGRAS genes with six introns, one PpGRAS gene with five introns, seven PpGRAS genes with four introns, and three PpGRAS genes with three introns. These results revealed that the intron evolution of GRAS genes may have a higher variability in *P. patens*. In addition, 63.2% (24 of 38) PpGRAS genes had one or zero intron, which was similar to that of angiosperm and *S. moellendorffii*. This phenomenon indicated that the ancient PpGRAS genes may have multiple introns but gradually lose some introns in evolution. Finally, most PpGRAS genes lost all introns or only retained a single intron.

Together, these results showed that GRAS proteins can be classified into seven large groups (groups I–VII), and this classification was supported by the position of conserved motifs. Most GRAS proteins had a similar exon–intron structure except *P. patens*, indicating that these conserved intron structures were something like necessary for the regulation of GRAS gene expression.

### Duplication events in the GRAS gene family

It is well known that gene duplication provides the raw material for function diversification. Gene families can arise through tandem amplification, resulting in a clustered occurrence, or through segmental duplication of chromosomal regions, resulting in a scattered occurrence of family members. In this analysis, we focused on the tandem and segmental duplication modes. To identify the amplification patterns of the GRAS gene family, we first identified the existence of tandem duplications. Of the 289 GRAS genes, 36 (12.5%) were clustered together, with a maximum of 10 extra genes between them, and may be considered tandemly duplicated genes [24]. The members of tandemly duplicated genes in the six plant species were listed in Table 1, including 4, 6, 7, 17, 0, and 2genes in *Arabidopsis*, *B. distachyon*, rice, soybean, *S. moellendorffii*, and *P. Patens* respectively. Intriguingly, all the putative tandemly duplicated genes were found in group II except *LOC_Os02g44360* and *LOC_Os02g44370*, suggesting that tandem duplication may contribute more to the expansion of the GRAS genes family in group II than in other groups. An effective and efficient way to detect segmental duplication events is to identify additional paralogous protein pairs in the neighborhood of each of the GRAS genes [25]. As shown in Table 2, 107 pairs (43.9%; 127 of 289genes) of paralogous genes were detected, supported by the high bootstrap values in the phylogenetic tree and the similar exon–intron structures, which suggests that segmental duplication has contributed to the expansion of the GRAS gene family. More intriguingly, segmental duplication events appeared to be rare in the GRAS gene family except in soybean (82 pairs), with 6, 4, 10, 0, and 4 pairs in *Arabidopsis*, *B. distachyon*, rice, *S. moellendorffii*, and *P. patens* respectively. About 79% (84 of 106) of GmGRAS genes included segmental duplications, indicating that segmental duplication events were mainly found in the soybean genome. In short, segmental and tandem duplication events were involved in the expansion of the GRAS superfamily in all species except *S. moellendorffii*. Among these, tandem duplication greatly amplified group II, and segmental duplication were the dominant pattern in the evolution of GmGRAS genes. However, in *Arabidopsis*, *B. distachyon*, rice, *S. moellendorffii*, and *P. patens*, no single expansion pattern exhibited dominance, indicating that GRAS genes from these species might have been subjected to a more complex evolutionary mechanism.

**Table 1:** **Genes involved in tandem duplication**

**Tandem duplicated gene**	**Chromosome**	**group**
AT1G07520	1	II
AT1G07530	1	II
AT2G29060	2	II
AT2G29065	2	II
Bradi4g09155	4	II
Bradi4g09160	4	II
Bradi4g09170	4	II
Bradi4g09180	4	II
Bradi4g09190	4	II
Bradi4g09197	4	II
LOC_Os02g44360	2	VI
LOC_Os02g44370	2	VI
LOC_Os11g47870	11	II
LOC_Os11g47890	11	II
LOC_Os11g47900	11	II
LOC_Os11g47910	11	II
LOC_Os11g47920	11	II
Glyma11g14670	11	II
Glyma11g14700	11	II
Glyma11g14710	11	II
Glyma11g14720	11	II
Glyma11g14740	11	II
Glyma11g14750	11	II
Glyma12g06630	12	II
Glyma12g06640	12	II
Glyma12g06655	12	II
Glyma12g06670	12	II
Glyma13g41220	13	II
Glyma13g41240	13	II
Glyma13g41261	13	II
Glyma15g04160	15	II
Glyma15g04166	15	II
Glyma15g04173	15	II
Glyma15g04190	15	II
Pp1s359_32V6	*	II
Pp1s359_34V6	*	II

Note: *represents the unknown data.

**Table 2:** **Estimates of the dates for the segmental duplication events of GRAS gene superfamily in six species**

**Segment pairs**	**Number of anchors**	**Ks (mean ± s.d.)**	**Estimated time (mya)**
AT1G07520 & AT2G29065	10	0.819 ± 0.117	27.3
AT1G14920 & AT2G01570	12	0.737 ± 0.106	24.6
AT2G45160 & AT3G60630	17	0.714 ± 0.143	23.8
AT3G03450 & AT5G17490	18	0.759 ± 0.158	25.3
AT3G46600 & AT5G59450	7	0.817 ± 0.104	27.2
AT3G50650 & AT5G66770	15	0.837 ± 0.105	27.9
Bradi1g36180 & Bradi3g07160	5	0.754 ± 0.162	55.6
Bradi1g78230 & Bradi3g32890	13	0.784 ± 0.160	60.3
Bradi3g32890 & Bradi3g50930	2	0.550 ± 0.283	42.3
Bradi4g24867 & Bradi4g41880	9	0.723 ± 0.164	55.6
LOC_Os01g45860 & LOC_Os05g49930	5	0.540 ± 0.093	41.5
LOC_Os01g71970 & LOC_Os05g31380	3	0.517 ± 0.220	39.8
LOC_Os02g10360 & LOC_Os06g40780	7	0.613 ± 0.165	47.2
LOC_Os02g44360 & LOC_Os10g40390	2	0.725 ± 0.120	55.8
LOC_Os02g44360 & LOC_Os04g46860	6	0.750 ± 0.287	57.7
LOC_Os02g45760 & LOC_Os04g49110	7	0.619 ± 0.150	47.6
LOC_Os03g09280 & LOC_Os10g22430	3	0.760 ± 0.096	58.5
LOC_Os04g46860 & LOC_Os10g40390	4	0.568 ± 0.152	43.7
LOC_Os05g40710 & LOC_Os12g02870	3	0.677 ± 0.040	52.1
LOC_Os11g03110 & LOC_Os12g02870	20	0.103 ± 0.066	7.9
Glyma01g33270 & Glyma03g03760	5	0.104 ± 0.038	8.5
Glyma01g38360 & Glyma02g06530	9	0.697 ± 0.179	13.9
Glyma01g38360 & Glyma11g06980	21	0.170 ± 0.096	57.1
Glyma01g38360 & Glyma16g25570	7	0.764 ± 0.192	62.6
Glyma01g40180 & Glyma05g22460	5	0.604 ± 0.185	12.0
Glyma01g40180 & Glyma11g05110	35	0.147 ± 0.080	49.5
Glyma01g40180 & Glyma17g17400	7	0.656 ± 0.178	53.8
Glyma01g43620 & Glyma11g01850	33	0.129 ± 0.051	10.6
Glyma01g43620 & Glyma11g10170	5	0.452 ± 0.066	35.9
Glyma01g43620 & Glyma12g02490	5	0.438 ± 0.082	37.0
Glyma02g01530 & Glyma03g37851	13	0.654 ± 0.104	13.6
Glyma02g01530 & Glyma19g40440	16	0.682 ± 0.163	55.9
Glyma02g06530 & Glyma11g06980	12	0.779 ± 0.166	16.6
Glyma02g06530 & Glyma16g25570	16	0.203 ± 0.108	63.9
Glyma02g08241 & Glyma16g27310	23	0.172 ± 0.077	14.1
Glyma02g46730 & Glyma08g43780	10	0.567 ± 0.125	11.2
Glyma02g46730 & Glyma14g01960	42	0.137 ± 0.126	43.2
Glyma02g46730 & Glyma18g09030	7	0.527 ± 0.097	46.5
Glyma02g47640 & Glyma14g01020	41	0.125 ± 0.068	10.2
Glyma03g06530 & Glyma07g18934	8	0.666 ± 0.083	54.3
Glyma03g06530 & Glyma18g43580	6	0.663 ± 0.078	54.6
Glyma03g37851 & Glyma19g40440	38	0.164 ± 0.121	62.1
Glyma04g42090 & Glyma06g12701	35	0.163 ± 0.090	13.4
Glyma04g42090 & Glyma13g09220	5	0.638 ± 0.111	45.1
Glyma04g42090 & Glyma14g27290	2	0.550 ± 0.085	52.3
Glyma04g43090 & Glyma06g11610	28	0.143 ± 0.074	11.7
Glyma04g43090 & Glyma13g02840	3	0.777 ± 0.135	63.7
Glyma05g03020 & Glyma17g13680	29	0.135 ± 0.067	11.1
Glyma05g03490 & Glyma17g14030	31	0.159 ± 0.065	13.0
Glyma05g22140 & Glyma17g17710	7	0.206 ± 0.114	16.7
Glyma05g22460 & Glyma11g05110	5	0.526 ± 0.062	14.3
Glyma05g22460 & Glyma17g17400	11	0.174 ± 0.093	43.1
Glyma05g27190 & Glyma08g10140	27	0.157 ± 0.107	12.9
Glyma06g11610 & Glyma13g02840	6	0.828 ± 0.141	67.9
Glyma06g12701 & Glyma13g09220	5	0.664 ± 0.090	50.4
Glyma06g12701 & Glyma14g27290	2	0.615 ± 0.106	54.4
Glyma06g41500 & Glyma12g16750	5	0.270 ± 0.280	22.1
Glyma06g41500 & Glyma12g34420	9	0.523 ± 0.070	41.8
Glyma06g41500 & Glyma13g36120	9	0.510 ± 0.150	42.9
Glyma07g04430 & Glyma16g01020	29	0.172 ± 0.144	14.1
Glyma07g15950 & Glyma18g39920	6	0.145 ± 0.092	11.9
Glyma07g18934 & Glyma18g43580	15	0.160 ± 0.048	13.1
Glyma07g39650 & Glyma09g01440	17	0.632 ± 0.156	11.4
Glyma07g39650 & Glyma15g12320	17	0.681 ± 0.173	51.8
Glyma07g39650 & Glyma17g01150	40	0.139 ± 0.123	55.8
Glyma08g43780 & Glyma14g01960	8	0.579 ± 0.138	10.2
Glyma08g43780 & Glyma18g09030	13	0.124 ± 0.028	47.5
Glyma09g01440 & Glyma15g12320	40	0.143 ± 0.070	11.7
Glyma09g01440 & Glyma17g01150	18	0.684 ± 0.162	56.1
Glyma09g40620 & Glyma18g45220	22	0.194 ± 0.140	15.9
Glyma10g04421 & Glyma13g18680	28	0.136 ± 0.065	11.1
Glyma10g33380 & Glyma20g34260	31	0.171 ± 0.115	14.0
Glyma10g35920 & Glyma20g31680	30	0.130 ± 0.062	10.7
Glyma10g37640 & Glyma16g29900	11	0.614 ± 0.125	11.6
Glyma10g37640 & Glyma20g30150	32	0.141 ± 0.068	50.3
Glyma11g01850 & Glyma11g10170	4	0.393 ± 0.025	31.0
Glyma11g01850 & Glyma12g02490	4	0.378 ± 0.029	32.2
Glyma11g05110 & Glyma17g17400	8	0.671 ± 0.175	55.0
Glyma11g06980 & Glyma16g25570	7	0.729 ± 0.149	59.8
Glyma11g10170 & Glyma12g02490	39	0.140 ± 0.076	11.5
Glyma11g10220 & Glyma12g02530	41	0.148 ± 0.076	12.1
Glyma11g14670 & Glyma12g06630	28	0.125 ± 0.048	10.2
Glyma11g14670 & Glyma15g04160	17	0.575 ± 0.157	47.1
Glyma11g14700 & Glyma12g06640	27	0.120 ± 0.034	9.8
Glyma11g14700 & Glyma13g41240	16	0.629 ± 0.182	47.1
Glyma11g14700 & Glyma15g04173	17	0.575 ± 0.158	51.6
Glyma11g33720 & Glyma18g04500	20	0.175 ± 0.177	14.3
Glyma12g06630 & Glyma13g41240	13	0.578 ± 0.148	46.8
Glyma12g06630 & Glyma15g04160	16	0.571 ± 0.163	47.4
Glyma12g06640 & Glyma13g41220	13	0.578 ± 0.148	46.7
Glyma12g06640 & Glyma15g04173	16	0.570 ± 0.160	47.4
Glyma12g16750 & Glyma12g34420	4	0.513 ± 0.130	42.0
Glyma12g16750 & Glyma13g36120	4	0.543 ± 0.217	44.5
Glyma12g32350 & Glyma13g38080	28	0.189 ± 0.147	15.5
Glyma12g34420 & Glyma13g36120	27	0.149 ± 0.080	12.2
Glyma13g09220 & Glyma14g27290	2	0.115 ± 0.021	9.4
Glyma13g41220 & Glyma15g04173	43	0.149 ± 0.110	12.2
Glyma13g42100 & Glyma15g03290	38	0.149 ± 0.139	12.2
Glyma14g01960 & Glyma18g09030	6	0.548 ± 0.120	44.9
Glyma15g12320 & Glyma17g01150	16	0.682 ± 0.156	55.9
Glyma16g05751 & Glyma19g26735	9	0.132 ± 0.057	10.8
Glyma16g29900 & Glyma20g30150	8	0.633 ± 0.110	51.9
Pp1s165_77V6 & Pp1s63_181V6	2	0.480 ± 0.030	*
Pp1s130_58V6 & Pp1s31_40V6	7	0.780 ± 0.119	*
Pp1s31_35V6 & Pp1s130_63V6	8	0.749 ± 0.121	*
Pp1s72_74V6 & Pp1s117_143V6	2	0.685 ± 0.265	*

Note: *represents the unknown data.

Previous studies have reported several rounds of whole-genome duplication (WGD) in *Arabidopsis*, *B. distachyon*, rice, soybean, and *P. patens*. Thus, the approximate dates of the segmental duplication events were estimated using Ks. The mean Ks values, standard deviations, and estimated dates for all segmental duplication events corresponding to GRAS genes were listed in Table 2. In *Arabidopsis*, six pairs of AtGRAS paralogous genes originated around 23.8 Mya (million years ago) to 27.9 Mya, which was consistent with the date of the recent large-scale duplications which occurred at 24–40 Mya [26]. In *B. distachyon*, three pairs of BdGRAS paralogous genes corresponded to a WGD event that is thought to have occurred around 56–73 Mya [27]. The other two pairs likely resulted from a single duplication event which occurred at about 40 Mya. In rice, nine pairs of OsGRAS paralogous genes appeared to be derived from a WGD which occurred at 40–50 Mya [28]. One pair (*LOC_Os11g03110* and *LOC_Os12g02870*) of segmental duplicates were estimated to originate around 7 Mya, which was compatible with a segmental duplication that occurred on the ends of chromosomes 11 and 12, estimated to have been separated in evolution for 5–10 Mya [7]. In soybean, Schmutz et al. have found that two large-scale duplication events occurred at approximately 59 and 13 Mya, respectively [29]. Our results focused on two periods, 9–16 Mya and 40–70 Mya, which were roughly consistent with the age of the two duplication events. In the previous study, Du et al. [30] have identified genes which originate from WGD duplication and independent duplication in soybean genome. To further verify the results, we compared the 84 segmentally duplicated GmGRAS genes identified in our study with the results of Du et al. [30]. We concluded that 70 of 84 (83.3%) GmGRAS genes were originated from WGDs, whereas 10 of 84 (11.6%) GmGRAS genes were derived from independent duplication events (data not shown). In *P. patens*, Rensing et al. found an ancient genome duplication event that was thought to have occurred between 30 and 60 Mya [31]. Later, they reported that the Ks distribution plot (i.e., the frequency classes of synonymous substitutions) among paralogs showed a clear peak at around 0.5 to 0.9 in 2008, which suggests that a large-scale duplication, possibly involving the whole genome, has occurred [32]. Our results showed that the K_s_ value of four pairs of PpGRAS paralogous genes range from 0.48 to 0.78, which was compatible with the previous study. In *S. moellendorffii*, no segmental and tandem duplication events were detected, and this result may have some connection with the fact that the *Selaginella* genome lacks evidence of an ancient whole-genome duplication or polyploidy [33]. In addition, these results were consistent with the analyses of Edger et al. that transcription factors were preferentially retained following WGDs [34]. We also submitted all deduced tandemly duplicated genes to the Plant Genome Duplication Database to obtain tandemly duplicated pairs in six species. However, no homologous genes were found among species, indicating that those tandemly duplicated genes were retained after speciation of six species we studied.

In short, tandem duplication events played an important role in the expansion of group II. Segmental duplication was predominant among GRAS genes in soybean. Moreover, a great majority of the genes involved in segmental duplication were retained after WGDs.

### Functional divergence analysis of GRAS family

Two types (Type I and Type II) of functional divergence between gene clusters of the GRAS subfamily were inferred by posterior analysis using DIVERGE2, which estimates significant changes in the site-specific shift of evolutionary rate (Type I) or the site-specific shift of amino acid properties (Type II) after the emergence of two paralogous sequences [35]. The advantage of these methods is that they use amino acid sequences and therefore are not sensitive to the saturation of synonymous sites [36]. The estimation was based on the GRAS protein NJ tree, in which eight major subfamilies were clearly presented with highly significant support from bootstrap values. The result showed that the coefficient of Type I functional divergence (θ_I_) between any two relevant clusters was significantly greater than 0 (*p* < 0.05, Table 3), which indicates a highly different site-specific altered selective constraint between them. The coefficients of Type II functional divergence (θ_II_) were only significant (*p* < 0.05) between I/III, III/IV, and III/V, particularly III/V. The coefficient of Type II functional divergence (θ_II_) between other groups was smaller than 0, while the standard errors were relatively high. These results revealed that the functional evolution of subfamilies of the GRAS gene family might adopt Type I and Type II functional divergence in different degrees.

**Table 3:** **Functional divergence between subfamilies of the GRAS gene superfamily in six species**

	**Type-I**			**Type-II**	
	**θ**_**I**_ **± s.e.**	**LRT**	**Qk > 0.95**	**θ**_**II**_ **± s.e.**	**Qk > 0.95**
group I/group II	0.646 ± 0.046	193.083	23	0.305 ± 0.693	46
group I/group III	0.694 ± 0.061	127.491	20	−0.144 ± 0.654	0
group I/group IV	0.530 ± 0.053	100.097	11	−0.125 ± 0.387	0
group I/group Va	0.433 ± 0.059	53.918	3	0.323 ± 0.476	57
group I/group Vb	0.430 ± 0.055	61.083	3	−0.042 ± 0.358	6
group I/group VI	0.507 ± 0.062	67.151	8	0.008 ± 0.436	8
group I/group VII	0.682 ± 0.058	139.147	24	0.175 ± 0.579	31
group II/group III	0.527 ± 0.058	82.457	9	−0.667 ± 1.661	0
group II/group IV	0.637 ± 0.052	148.226	24	−0.368 ± 0.782	0
group II/group Va	0.518 ± 0.050	108.565	13	−0.306 ± 1.302	0
group II/group Va	0.553 ± 0.061	83.348	9	−0.534 ± 0.876	0
group II/group VI	0.569 ± 0.054	110.105	13	−1.387 ± 1.364	0
group II/group VII	0.571 ± 0.050	131.122	13	−0.800 ± 1.793	0
group III/group IV	0.312 ± 0.063	24.427	2	−0.018 ± 0.502	7
group III/group Va	0.365 ± 0.068	28.522	0	−0.521 ± 1.068	0
group III/group Vb	0.155 ± 0.074	4.399	0	−0.365 ± 0.600	0
group III/group VI	0.232 ± 0.057	16.351	0	−0.167 ± 0.667	1
group III/group VII	0.150 ± 0.066	5.175	0	−0.934 ± 1.358	0
group IV/group Va	0.218 ± 0.063	12.172	0	−0.262 ± 0.505	0
group IV/group Vb	0.072 ± 0.050	2.053	0	−0.458 ± 0.322	0
group IV/group VI	0.335 ± 0.053	40.429	5	−0.517 ± 0.402	0
group IV/group VII	0.287 ± 0.052	31.042	1	−0.579 ± 0.631	0
group Va/group VI	0.286 ± 0.058	24.61	3	−0.303 ± 0.642	0
group Va/group II	0.373 ± 0.062	36.252	1	−1.393 ± 1.367	0
group Vb/group VI	0.001 ± 0.22	0	0	−0.852 ± 0.422	0
groupVb/group VII	0.094 ± 0.043	4.769	0	−0.504 ± 0.661	0
group VI/group VII	0.190 ± 0.059	10.338	0	−1.113 ± 0.929	0

Note: θI and θII, the coefficients of Type-I and Type-II functional divergence.

LRT, Likelihood Ratio Statistic.

Q_k_, posterior probability.

To identify the critical amino acid sites (CAASs) that may be responsible for functional divergence between GRAS subgroups, the posterior probability (Q_k_) of divergence was identified using functional divergence-related residues [35]. A large Q_k_ value indicates a high possibility that the functional constraint or amino acid physiochemical property of a site differ between two clusters. In this study, Q_k_ > 0.95 was used as the cutoff to identify CAASs between gene clusters. Our results showed distinct differences in the number of sites for which functional divergence was predicted within each pair. A total of 66 CAASs (amino acids referring to the *AT3G54220* sequence) were predicted by Type I functional divergence analysis. Of these, 24, 24, 23, and 20 Type I-related CAASs were identified for the I/VII, II/IV, I/II, and I/III pairs, respectively, which suggests that these sites might act as a major evolutionary force driving the divergence of I/VII, II/IV, I/II, and I/III. Meanwhile, 87 Type II-related CAASs were identified for I/II, I/V, I/VI, I/VII, III/IV, and III/VII pairs. Compared with only three CAASs for the Type I functional divergence between I/Va, there were 57 predicted sites for Type II functional divergence, indicating that the rapid change in amino acid physiochemical properties was mainly attributed to the functional divergence between the two groups of genes, and secondarily attributed to the shift in evolution rate. The case was similar for I/II and I/VII pairs. However, most of the pairs did not follow the above model, indicating that site-specific shifts in evolutionary rate and changes in amino acid property do not uniformly act on the GRAS subfamily members over evolutionary time. Finally, 44 amino acids were identified as co-occurring amino acids for both Type I and Type II functional divergence (Additional file 17), suggesting that these sites were important for the subgroup-specific functional evolution of the GRAS gene.

### Positive selection in the GRAS gene family

Positive selection is one of the major forces in the emergence of new motifs and functions in proteins after gene duplication. In this study, likelihood ratio tests were implemented in the PAML v4.4 software package [37] to test the hypothesis of positive selection in the GRAS gene family using a site-specific model. First, we performed independent analyses of positive selection using full-length protein GRAS sequences from six different species. The results (Additional files 18, 19, 20, 21, 22 and 23) showed that none CAASs for positive selection were identified in *Arabidopsis*, rice, or soybean, *B. distachyon*, *S. moellendorfii*, while 30 (11 of them were at the 0.05 significance level and 19 of them were at the 0.01 significance level) positive selection sites were identified in *P. patens* based on the Bayes empirical Bayes (BEB) estimation method. These results implied that PpGRAS genes were under higher positive selection pressure, while the other five species appeared to be more conservative. Analysis of the combined six species was also performed, and the parameter estimates and log-likelihood values for each model are provided in Table 4. The LRT statistic for M3 vs. M0 comparison was 2∆ℓ = 3508.354, much greater than critical values from aχ^2^distribution with d.f. = 4, indicating that one category of ω was insufficient to describe the variability in selection pressure across amino acid sites. However, when M7/M8 was compared, none CAASs were identified as positively selected sites. This result suggested that GRAS gene superfamily was relatively conserved during evolution. In short, GRAS genes were subject to different levels of positive selection pressure, regardless of whether the genes were intraspecific or interspecific.

**Table 4:** **Tests for positive selection among codons of GRAS genes using site-specific model**

**Model**	**lnL**	**Estimates of parameter** ^**a**^	**2**Δ**lnL**	**Positive selection sites** ^**b**^
M0(one-ratio)	−82992.756	ω = 0.12433	3508.354	Not allowed
M3(discrete)	−81238.579	p_0_ = 0.20058 ω_0_ = 0.03406	(M3vsM0)**	None
		p_1_ = 0.55607 ω_1_ = 0.10851		
		p_2_ = 0.23655 ω_2_ = 0.28245		
M7(beta)	−81023.838	p = 0.99909 q = 4.93337	0.002	Not allowed
M8(beta & ω)	−81023.839	p_0_ = 0.99999 p = 0.99909	(M8vsM7)	None
		q = 4.93337 p_1_ = 0.00001		
		ω = 1.00000		

Note: *p < 0.05 and **p < 0.01 (*x* ^2^ test).

^a^ω was estimated under model M0,M3,M7, and M8; p and q are the parameters of the beta distribution.

^b^The number of amino acid sites estimated to have undergone positive selection.

To study the adaptive evolution of the GRAS subfamilies, we further analyzed the branch-site model. On the GRAS gene tree (Figure 1), seven branches (I, II, III, IV, V, VI, and VII) were independently defined as the foreground branch. Table 5 listed parameter estimates and log-likelihood values under the branch-site models. None or a few remarkably significant sites were found under the x^2^ test (*p* < 0.05) in groups II, III, IV, VI, and VII. However, significant positive selection was detected when group I and V were defined as the foreground branch. Among them, 16 sites were identified as positively selected sites when branch I was considered to be the foreground branch and four of them (415P, 453 F, 476E, and 505 T) were significant according to the x^2^ test (*p* < 0.01). 11 sites were identified as positively selected sites when branch V was considered the foreground branch. Of these sites, one positive selection site (418 F) was at the 0.05 significance level, while ten sites (296Q, 303A, 412 L, 453 F, 490 W, 497D, 508 L, 511R, 513A, and 518 T) were at the 0.01 significance level. These results suggested that groups I and V were confronted with strong positive selection pressure, as many highly significant positive sites were present, whereas the other groups were likely experiencing strong purifying or neutral selection pressure.

**Table 5:** **Parameters estimation and likelihood ratio tests for the branch-site models**

**Foreground branches**	**Estimates of parameter**				**positive selection sites (BEB)** ^**4**^
	**Site class** ^**1**^ **0**	**Site class 1**	**Site class 2a**	**Site class 2b**	
Group I	P0 = 0.50799	P1 = 0.05716	P2a = 0.39087	P2b = 0.04398	296Q*,337A*,397 K*,407D*,
	ω0(b)^2^ = 0.13998	ω1(b) = 1.00000	ω2a(b) = 0.13998	ω2b(b) = 1.00000	412 L*,415P**,419H*,446 T*,
	ω0(f)^3^ = 0.13998	ω1(f) = 1.00000	ω2a(f) = 3.03087	ω2b(f) = 3.03087	453 F**,457 L*,464C*,476E**, 505 T**,510Q*,527A*, 535 V*
Group II	P0 = 0.63235	P1 = 0.07147	P2a = 0.26611	P2b = 0.03007	644 L*
	ω0(b) = 0.13987	ω1(b) = 1.00000	ω2a(b) = 0.13987	ω2b(b) = 1.00000	
	ω0(f) = 0.13987	ω1(f) = 1.00000	ω2a(f) = 1.16777	ω2b(f) = 1.16777	
Group III	P0 = 0.69273	P1 = 0.07803	P2a = 0.20604	P2b = 0.02321	None
	ω0(b) = 0.14005	ω1(b) = 1.00000	ω2a(b) = 0.14005	ω2b(b) = 1.00000	
	ω0(f) = 0.14005	ω1(f) = 1.00000	ω2a(f) = 999.00000	ω2b(f) = 999.00000	
Group IV	P0 = 0.86848	P1 = 0.04356	P2a = 0.08376	P2b = 0.00420	328Q*, 368S**
	ω0(b) = 0.13026	ω1(b) = 1.00000	ω2a(b) = 0.13026	ω2b(b) = 1.00000	
	ω0(f) = 0.13026	ω1(f) = 1.00000	ω2a(f) = 20.88429	ω2b(f) = 20.88429	
Group V	P0 = 0.63670	P1 = 0.03187	P2a = 0.31563	P2b = 0.01580	296Q**,303A**,412 L**,418 F*, 453 F*,490 W**,497D**,508 L**, 511R**, 513A**, 518 T**,
	ω0(b) = 0.12995	ω1(b) = 1.00000	ω2a(b) = 0.12995	ω2b(b) = 1.00000	
	ω0(f) = 0.12995	ω1(f) = 1.00000	ω2a(f) = 1.56269	ω2b(f) = 1.56269	
Group VI	P0 = 0.72413	P1 = 0.03621	P2a = 0.22825	P2b = 0.01141	448 K*, 456 K**, 515 K**
	ω0(b) = 0.12948	ω1(b) = 1.00000	ω2a(b) = 0.12948	ω2b(b) = 1.00000	
	ω0(f) = 0.12948	ω1(f) = 1.00000	ω2a(f) = 1.26601	ω2b(f) = 1.26601	
Group VII	P0 = 0.71371	P1 = 0.03569	P2a = 0.23866	P2b = 0.01194	297C**, 335S*, 497D*, 551R*
	ω0(b) = 0.12951	ω1(b) = 1.00000	ω2a(b) = 0.12951	ω2b(b) = 1.00000	
	ω0(f) = 0.12951	ω1(f) = 1.00000	ω2a(f) = 76.78801	ω2b(f) = 76.78801	

Note: *p < 0.05 and **p < 0.01 (*x* ^2^ test).

^1^The sites in the sequence evolve according to the same process, the transition probability matrix is calculated only once for all sites for each branch.

^2^Background ω.

^3^Foreground ω.

^4^The number of amino acid sites estimated to have undergone positive selection; BEB: Bayes Empirical Bayes.

**Figure 2:**
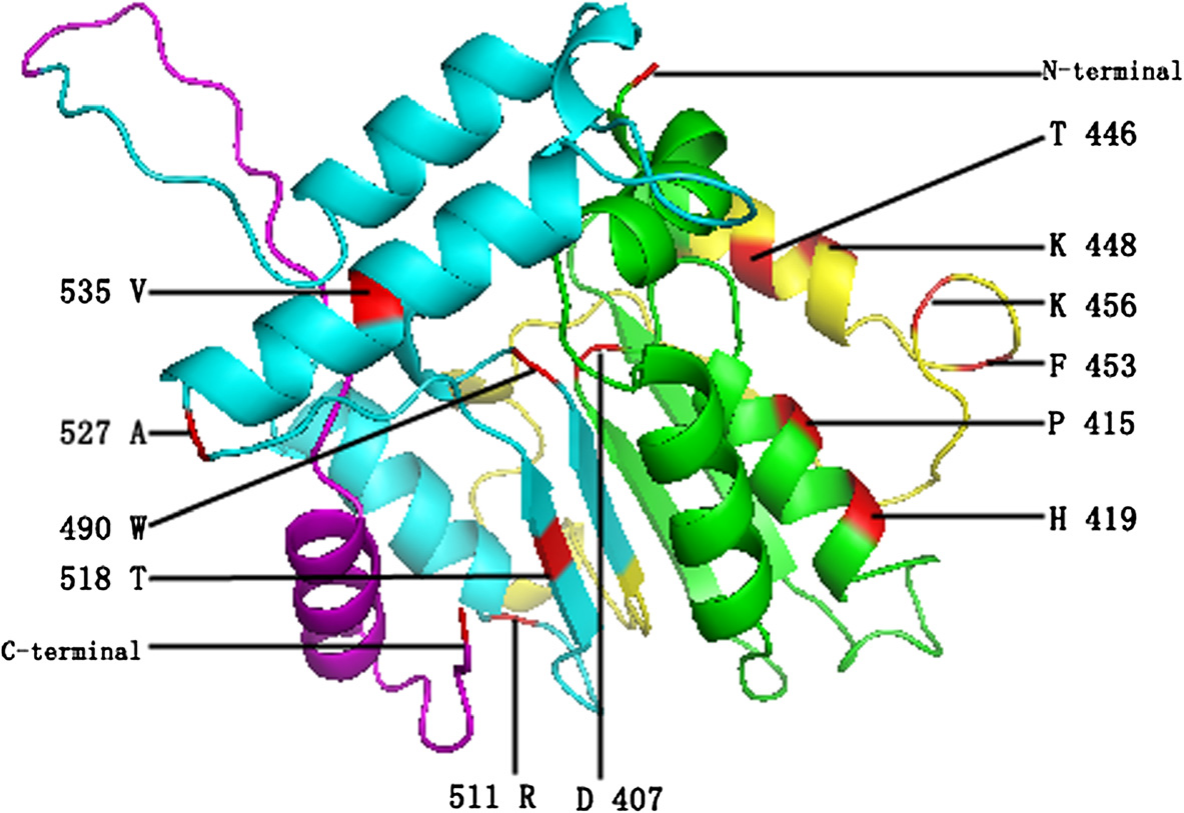
**Model building of the three-dimensional structure of the GRAS protein.** The VHIID, LHRII, PFYRE, and SAW motifs are presented in green, yellow, blue, and pink, respectively. The figure was produced using the CPHmodels program, and amino acids refer to the *AT3G54220* sequence.

Finally, we observed relationships between amino acid sites under positive selection and functional divergence, 14 critical amino acid sites were under positive selection as well as Type I and Type II functional divergence (Additional file 17). We located them on the three-dimensional GRAS structure and performed multiple sequence alignment to further investigate their function. As the displayed sequence produced incompetence by CPHmodels [38], only 12 sites were labeled on the three-dimensional structure, and other amino acid sites were labeled in multiple sequence alignment (Figure 2 and Additional file 13). Among these, two amino acids (296Q and 368S) was located on the LHRI motif, three amino acids (407D, 415P, and 419H) were located on the VHIID motif, and four amino acids (446 T, 448 K, 453 F, and 456 K) were located on the LHRII motif, and five amino acids (490 W, 511R, 518 T, 527A, and 535 V) were located on the PFYRE motif. In short, most of the amino acids were located on the α - helix. These results revealed that these amino acids may act as a major evolutionary force driving the divergence of GRAS-conserved motifs and may further affect the divergence of GRAS subgroup functions. More experimental evidence is needed to understand the functional importance of the identified CAASs. In addition, Zhang et al. recovered significant hits to several Rossmann fold methyltransferase domains in bacterial GRAS proteins [6]. Surprisingly, we also found the Rossmann fold (βαβαβ) in our protein (AT3G54220). These results also showed that the structure of GRAS proteins was conserved in lower and higher organisms.

### Expression analysis of GRAS genes

To investigate the expression patterns of homologous GRAS genes in subgroups involved in plant growth and development, we constructed a heat map using the Gene Pattern program. As the microarray data and RNA-Seq atlas of *B. distachyon* was incomplete, we focused on the three other species (*Arabidopsis*, soybean, and rice) studied in this paper. In *Arabidopsis*, the probeset ID of *AT2G29060* cannot be found in the ATH1 data source. Thus, only 32 AtGRAS genes were considered in our analysis of differential expression. In rice, eight genes (*LOC_Os11g47890*, *LOC_Os11g47910*, *LOC_Os11g47920*, *LOC_Os12g04200*, *LOC_Os05g4071*0, *LOC_Os12g02870*, *LOC_Os12g04380*, and *LOC_Os06g40780*) cannot be found in their corresponding probeset. A total of 39 probesets corresponding to 39 out of 47 (83%) unigenes were found. In soybean, the expression values of *Glyma02g01530*, *Glyma03g06530*, *Glyma10g35920*, *Glyma11g20980*, *Glyma12g16750*, *Glyma15g28410*, *Glyma17g13680*, *Glyma19g40440*, and *Glyma20g31680* were zero, indicating that these 9 genes were expressed in some special tissues or organs were stress induced (i.e., induced genes). Moreover, *Glyma01g18040* lacked expression information in SoyBase. Consequently, distinct transcript abundance patterns for only 96 GmGRAS genes were readily identifiable in the RNA-Seq atlas dataset.

**Figure 3:**
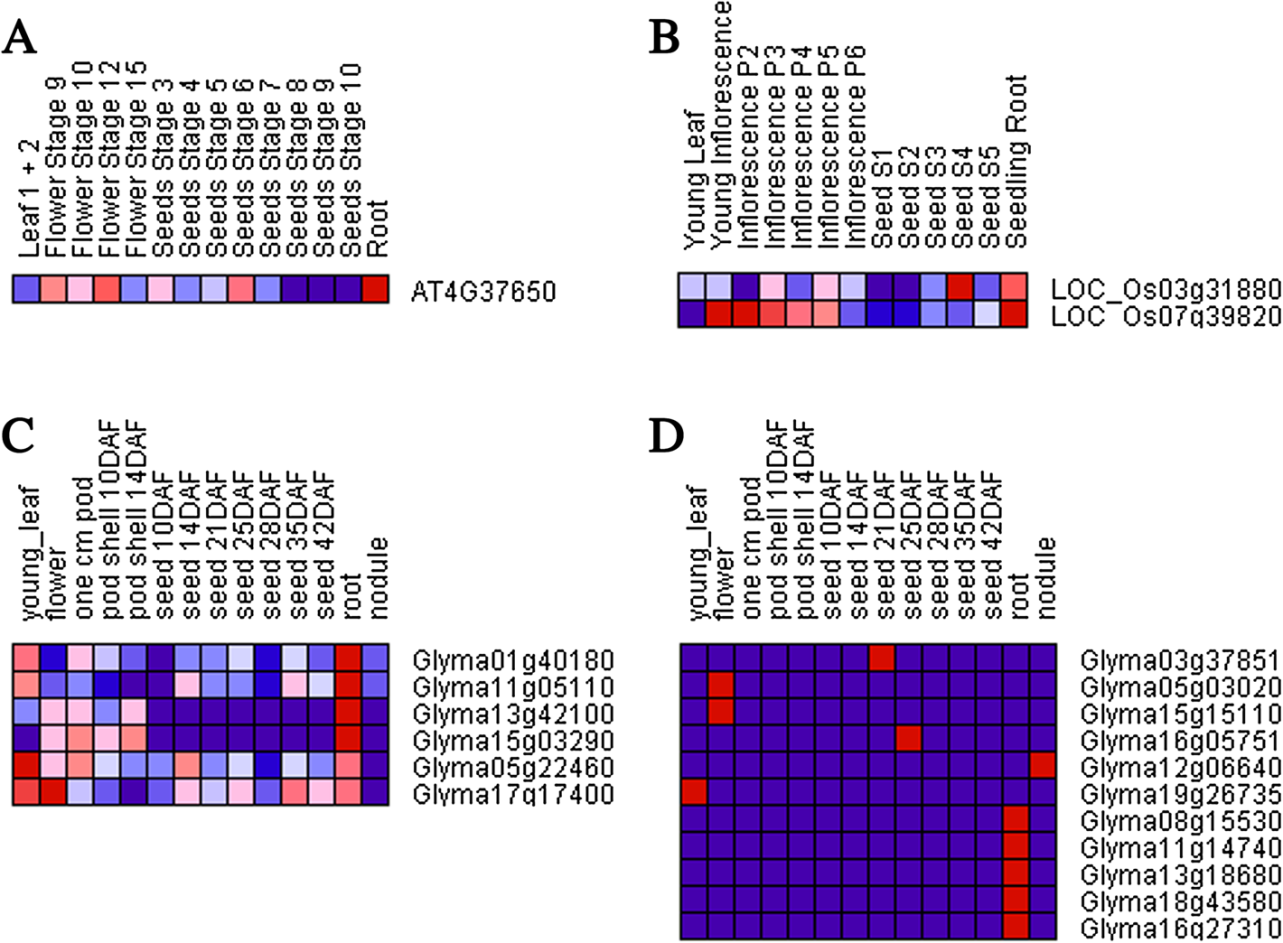
**Expression profiles of** ***Arabidopsis*** **, rice, and soybean GRAS genes.** According to the hierarchical cluster color code, the largest values are displayed as the most red (hot), the smallest values are displayed as the most blue (cool), and the intermediate values are lighter shades of blue or red. **A**, **B**, and **C** show that 9 GRAS genes clustered together in the tree have a similar preferential expression in the root. **D** shows the tissue-specific genes in soybean.

According to the expression profiles in Additional files 24, 25 and 26, broadly, our results showed that most GRASs had different expression levels in different tissues or organs. Further, some of the GRAS genes were obviously expressed in the vegetative growth stage and reproductive growth stages, suggesting that these GRAS genes may regulate specific functions corresponding to different stages in plant growth and development. Meanwhile, the same tissues and organs were regulated by multiple genes and the levels of expression differed in different GRAS genes, suggesting that multiple GRAS genes were involved in regulating the growth and development of the same tissues or organs. The GRAS genes showed different preferential expression in different species, and most GRAS genes exhibited expression profiles with marked peaks in only a single tissue type. In particular, there were many tissue-specific genes in soybean (Figure 3). For example, five genes were expressed only in the root, and two genes were expressed only in the seed. These results indicated that those GRAS proteins function as tissue-specific regulators or were limited to a single organ or cell type. Moreover, Lee et al. have described the expression analysis of some GRAS genes in *Arabidopsis* [18]. Although the processing time was different, our results showed that many ATGRAS genes had the similar level of expression. For example, SCL23 showed higher levels of expression in the leaves, flowers, and seeds than in the roots, which confirmed the previous view that SCL23 played a role in the aerial parts. Many of the other SCL genes showed expression in the root, including SCL4, SCL9, SCL11, SCL28, SCL30, SCL31, and so on. In addition, there were subgroups of genes that exhibited similar expression profiles in the same species but were relatively phylogenetically distinct. However, several phylogenetic clades shared the same transcript abundance profile to a large extent. In group III, a phylogenetic clade included nine GRAS genes from three species (Figure 3) that were preferentially expressed in the root. Evidently, the expression patterns of homologous gene subgroups are conserved at different degrees among the three species we studied.

It is well known that gene duplication increases expression diversity and enables tissue or developmental specialization to evolve. The Ohno’s classic model [39] concerning the fate of duplicated genes and the duplication–degeneration–complementation (DDC) model, predict for each one of the duplicates the gain of a new function (neofunctionalization), its loss (pseudogenization) or the development of overlapping redundant functions and expression patterns (subfunctionalization) [40,41]. To trace expression diversification and functionality of GRAS duplicated genes, *Arabidopsis* represents a model system for which both genome structure and gene expression patterns have been extensively studied. As shown in addition file 19, one pair of duplicated genes (*AT2G45160* and *AT3G60630*) had a same expression patterns. However, *AT3G4660* and *AT5G17490*, which exhibited the most redundant expression, develop opposite regulatory actions as they promote/repress, respectively, germination in response to leaves and roots. This effect would be more related to a case of neofunctionalization. The similar cases were found in the remaining duplicated genes. In addition, a pseudogenization process might be occurring in another pair of duplicated genes (*AT1G07520* and *AT2G29065*). The former seems to have a noticeably weaker expression than the latter in seeds. However, the fact that *AT1G07520* has a certain level of expression in the seeds could mean that the pseudogenization has not been completed.

In short, the expression profiles of the members of the GRAS subgroups were different in various organs and species, indicating that GRAS genes were differentially expressed in different groups and species, and the regulatory regions of GRAS genes may have diverged. Significantly, the results also demonstrated the expression divergence of the GRAS duplicated genes in the evolution.

## Discussion

### Comparative genomic analysis of the GRAS gene families

In this study, we identified 289 GRAS genes from six plant species and constructed a phylogenetic tree (Figure 1) that classified all the GRAS genes into seven major clusters, groups I–VII supported by the positions of conserved motifs. There was considerable bootstrapping value support for many of the defined groups and subgroups in the tree, but poor supporting values remained for several clusters. This was an expected consequence of performing a study like the present one with an average about 580 amino acid-length sequences, a constraint imposed by a large number of substitutable residues among GRAS-conserved motifs. It is worth mentioning that the definitions of most of the groups were supported by the presence of common protein motifs outside the GRAS domain. In each group, the number of GRAS genes in soybean was two or three times as high as the number of GRAS genes in other species, and there were many more tandem and segmental duplication events in soybean than in other species. The main reason may be that soybean has a highly duplicated genome (1,115 Mb) with more duplications than *Arabidopsis* (145 Mb) [42], *B. distachyon* (272 Mb) [27], rice (430 Mb) [43], *S. moellendorffii* (212.6 Mb) [33] or *P. patens* (511 Mb) [44] and nearly 75% of the genes present showed multiple copies [29].

On the other hand, most of the closely related members in the phylogenetic tree had common motif compositions, suggesting that there were functional similarities among the GRAS proteins within the same subfamily, so phylogenetic analysis will also facilitate functional genomics studies. For instance, the deduced twelve DELLA proteins clustered well with the determined six DELLA proteins (*GAI*, *RGA*, *RGL1*, *RGL2*, *RGL3*, and *SLR1*), which mediate the regulation of gene expression by gibberellins [45]. In the tree (Figure 1), one cluster of two GmGRAS proteins (*Glyma02g47640* and *Glyma14g01020*) was clustered well with *PAT1*, which functions in the response to far-red light and appears to act early in the phytochrome a signaling pathway. Thus, the mechanism of action of these two GmGRAS proteins may be similar to that of the *PAT1* protein. Similar cases were found in clusters consisting of *SHR* (*At4g37650*)/*Glyma01g40180*/*Glyma11g05110*/*Glyma05g22460*/*Glyma17g17400*, *SCL13* (*AT4G17230*)/*Glyma17g01150*/*Glyma07g39650*/*Glyma09g01440*/*Glyma15g12320*, *SCL3* (*At1G50420*)/*Glyma01g43620*/*Glyma11g01850*/*Glyma11g10170*/*Glyma12g02490*, and *SCR* (*At3g54220*)/*Glyma18g45220*/*Glyma09g40620*/*LOC_Os11g03110*/*LOC_Os12g02870*. Among these, *SHR* is involved in the radial organization of the root and shoot axial organs [5], *SCL13* is a positive regulator of phytochrome-dependent red-light signaling [46], *SCL3* promotes gibberellin signaling by antagonizing master growth repressor DELLA in *Arabidopsis* [47], and the *SCR* gene regulates an asymmetric cell division [1].

Intron evolution is an important part of genomic evolution, as well as being an adaptive process for speciation. Our results showed that most GRAS proteins had few introns (zero or one intron), and only a few GRAS genes had two introns. The similar gene structure of highly conserved introns was important to the molecular evolution of the GRAS family. However, most GRAS genes from *P. patens* had a different number of introns, and almost half of them had a longer sequence outside the GRAS domain than other species, suggesting that the evolution of introns in PpGRAS genes was a diverse and complex process.

### Expansion pattern of the GRAS gene family

Edger et al. [34] stated that dosage-sensitive genes, including transcription factors, were preferentially retained following WGDs. Recently, it was verified that some transcription factor families, such as WRKY and DOF, expanded through segmental duplication events, and most of them were retained after WGDs [48,49]. Some large multiprotein complexes also follow the same pattern. For example, Zhu et al. demonstrated that most of the segmentally duplicated soybean expansin genes have been retained from WGDs [50]. The present study showed that most identified segmentally duplicated genes in six species were also retained by WGD, which supported the results of Edger et al. On the other hand, in terms of groups, group II (67 genes, 23.2%) was the largest clade within the total group of GRAS genes, and most of the deduced tandemly duplicated genes were found in that group. This result demonstrated that tandem duplication greatly promoted the expansion of group II. However, the reasons for this result were unclear, and further research was needed. In terms of species, soybean had the most GRAS genes members in the six species we studied, and several factors may account for this. One reason is that soybean is an ancient polyploid with a larger genome than many other species. Another reason is two large-scale WGDs, which occurred at approximately 59 and 13 Mya resulted in a highly duplicated genome with nearly 75% of the genes present in multiple copies, and most genes involved in segmental duplication were retained after WGDs [29; 34]. Specially, segmental duplication is the predominant expansion pattern for GRAS genes in soybean. Among these, four pairs of GmGRAS genes (*Glyma11g14670*/*Glyma11g14700*, *Glyma12g06630*/*Glyma12g06640*, *Glyma13g41220*/*Glyma13g41240*, and *Glyma15g04160*/*Glyma15g04173*) were detected in both tandem and segmental duplication events, demonstrating that four pairs of GmGRAS genes experienced two different types of expansions. However, the estimated dates of these genes originated from segmental duplication events were obviously different, revealing that these GmGRAS genes first underwent tandem duplication and secondly segmental duplication. In short, the GRAS genes family showed different preferential the expansion patterns in different species. These different evolutionary patterns of the GRAS gene family in different species will help to facilitate further gene function analysis.

As Table 2 shown, the estimated dates of all deduced paralogous gene pairs ranged from 7.9 to 67.9 Mya, and all deduced tandemly duplicated genes may have originated after the speciation of their respective species. Taken together, the results clearly indicated that these GRAS duplicated genes, including 42% (14 of 33), 32% (14 of 44), 47% (22 of 47), 89% (94 of 106), and 26% (10 of 38) genes in *Arabidopsis*, *B. distachyon*, rice, soybean, and *P. patens* respectively, postdate the monocot–dicot split by approximately 200 Mya [51]. However, the presence of some *P. patens* sequences in the seven subfamilies suggests that GRAS gene family was formed before the divergence of mosses and the seed plant ancestors. Engstrom (2011) found that major GRAS protein subfamilies are ancient, which is consistent with results of Nishiyama et al. that the GRAS gene family arose before the appearance of land plants, over 400 million years ago [52,53]. The above analysis revealed that the GRAS gene family may originate from a common ancestor, followed by lineage-specific expansion and divergence in each lineage and species during its evolution. Moreover, the change of number of introns also revealed the evolution of introns of GRAS gene family. Most GRAS genes from angiosperm and *S. moellendorffii* either lacked introns or had only a single intron, while 36.8% PpGRAS genes had multiple introns, which suggests that GRAS gene family may initially contain multiple introns then lost all introns or only retained a single intron in evolution. In addition, Tian et al. deduced that there were two pairs of OsGRAS ancient duplicates, on the basis of the juxtaposition of *LOC_Os05g42130*/*LOC_Os07g40020* with *At3g49950* and *LOC_Os03g31880*/*LOC_Os07g39820* with *At4g37650* in the phylogenetic tree, and *At3g49950* and *At4g37650* were ancient duplicates that appeared to be derived from a genome duplication event predating the monocot–dicot divergence [7]. The same method was used in this study, and we deduced that there were three ancient BdGRAS genes, *Bradi1g22907*, *Bradi2g20760*, and *Bradi1g23060*. Furthermore, all the deduced ancient GRAS genes were from group III and contained no segmental or tandem duplication events, implying that these ancient GRAS genes from three species, over the course of evolution, experienced little or no amplification.

### Analysis of positive selection and functional divergence

In a gene family, new genes produced by duplication either evolve a new function and are retained because of positive selection or are lost during the course of evolution [54]. Usually, in the early stages of the evolution of duplicated genes, the genes are not subject to selection pressure (ka/ks ≈ 1) or display traits that subject them to positive selection (ka/ks > 1). In specific functional evolution, every gene has a fixed function, and selection pressure tends to purify selection (ka/ks < 1) [55,56]. Therefore, it is difficult to observe positive selection pressure when a duplicated gene is very old. In this study, whether the site-specific model or branch-site model was used, no or few significant sites were found in GRAS subfamilies except group I and group V (Tables 4 and 5). It is possible that some ancient GRAS proteins subject to purifying selection are the dominant evolutionary type, which would partially explain the above result. Nevertheless, we detected several CAASs that were under positive selection pressure. By contrast, PpGRAS genes experienced a relatively higher positive selection pressure, as they 30 positive selection sites, whereas the other five species studied appeared to be more conservative and no positive selection sites were detected. In addition, *P. patens* had a variety of exon–intron structures and longer sequence outside the GRAS domain than other species, which strongly supported this view. On the other hand, we detected 16 significant sites in group I, suggesting that these amino acid sites may act as a major evolutionary force in group I. Moreover, the analysis of functional divergence also supported this hypothesis. The CAASs were always identified when group I was compared with other groups in Type I (shift in evolutionary rate), strongly suggesting that group I-specific functional evolution of the GRAS gene is occurring or has occurred. Meanwhile, 11 CAASs were detected in group V. It is rather remarkable that the number of group V genes from *S. moellendorffii* and *P. paten*s reached the maximum in comparison with the other subgroups. Furthermore, compared with only three CAASs for the Type I functional divergence, there were 57 and 6 Type II-related CAASs were identified for the I/Va and I/Vb pairs, respectively, which strongly indicated that the physiochemical properties of some ancient amino acids may have changed in evolution, further driving the functional divergence of group I and group V. In addition, we identified twelve sites which were responsible for both functional divergence and positive selection. Typically, an amino acid residue is highly conserved in one duplicate gene, but highly variable in the other one [57]. So these CAASs partly reflect the coding regions of GRAS gene family may have diverged, and these CAASs may act as a major evolutionary force driving the functional divergence of GRAS gene family. On the other hand, functional divergence might reflect the existence of long-term selective pressures. Especially, significant differences in Type-I functional divergence between subfamily pairs indicated that different site-specific shifts in evolutionary rate may have occurred. In short, duplicated genes through long-term selection result in altered functional constraints between the gene clusters of GRAS gene family.

### Expression analysis of DELLA proteins

DELLA proteins constitute a subgroup of the GRAS family of plant-specific proteins. In this paper, we predicted the existence of 14 DELLA proteins that mediate the regulation of gene expression by gibberellins, which are involved in the transition from vegetative to reproductive growth [58]. Previous studies showed that they promote seed germination, leaf expansion, flowering, stem elongation, and flower development. In our expression profiles, *RGL1*, *RGL2*, *RGA*, *GAI* were preferentially expressed in flowers, which agrees with results from Cao et al. that gibberellin mobilizes distinct DELLA-dependent transcriptomes to regulate floral development in *Arabidopsis* [59]. Meanwhile, other DELLA proteins (*LOC_Os03g4990*, *Glyma08g10140*, and *Glyma05g27190*) from rice and soybean also showed a high expression level in flower (Additional files 24, 25 and 26). Furthermore, *RGL3* was preferentially expressed in seed, as were *Glyma10g33380*, *Glyma06g23940*, *Glyma04g21340*, and *Glyma18g04500*. However, *Glyma11g33720* was preferentially expressed in nodules. These results indicated that the functions of DELLA proteins were relatively conserved, but functional divergence still existed to meet special requirements in different species. Gallego-Bartolome et al. reported that functional diversification of different DELLA proteins in *Arabidopsis* is the result of subfunctionalization, probably due to changes in the proteins’ regulatory sequences [60]. More experiments are needed to reveal different mechanisms of transcription by DELLA proteins in different species.

## Conclusions

This study provides a comparative genomic analysis of the GRAS gene family in *Arabidopsis*, *B. distachyon*, rice, soybean, *S. moellendorffii*, and *P. patens*, assigning the GRAS genes to seven major clusters. The results of differential expression of the duplicated GRAS genes indicated that the proteins’ functions may have diverged to meet the special requirements of different species. The GRAS family of genes showed different expansion patterns in different species and groups. Segmental duplication was the predominant expansion pattern of the GRAS gene family in soybean, while tandem duplication events played an important role in the expansion of genes in group II. All putative duplicated genes were identified postdate the monocot–dicot split. Furthermore, these genes from group I and group V were under a higher positive selection pressure, which was revealed by the branch-site model. In addition, the site-specific model showed that GRAS genes experienced a higher positive selection pressure in *P. paten*s than in the other five more conservative species. Analyses of functional divergence showed that the CAASs were always identified when group I was compared with other groups in Type I, strongly suggesting that the shifted evolutionary rate may mainly attributed to group I-specific functional evolution. Finally, although the predicted 18 DELLA proteins were relatively conserved, their functions are diverging according to the expression profiles of the GRAS family. In short, our analysis provides a solid foundation for further functional dissection of GRAS genes in plants.

## Methods

### Identification of GRAS family members in four plant species

In plants, the model organism *Arabidopsis* is commonly used to predict the function of a gene in a newly or partially sequenced organism. Lee et al. identified 33 GRAS members in *Arabidopsis*, of which we excluded one pseudogene, *At5g67411*, from our analysis [18]. The 32 non-redundant GRAS gene sequences from the *Arabidopsis* Information Resource (TAIR) were used to blast against the Phytozome database http://www.phytozome.net. A data file containing all the information regarding the target genes, including location on chromosomes, genomic sequences, full coding sequences, and protein sequences, was collected from the above website. Sequences were selected as candidate proteins if their E value was ≤ 1e-5. The unique GRAS genes were identified by removing the redundant genes and the incomplete open reading frame sequences. The GRAS domain for each predicted protein was detected by searching against the SMART database (http://smart.emblheidelberg.de/). Then, genes without a typical GRAS domain (five recognizable motifs, LHR I, VHIID, LHR II, PFYRE, and SAW) were deleted. Moreover, the putative GRAS proteins that contain more than one GRAS domain were also excluded. Finally, the GRAS proteins were submitted to the ExPASY database to determine the Mw and pI.

To avoid the interference of pseudogenes, we exclude the pseudogenes with the following steps. Firstly, genes without a complete domain were excluded. Secondly, to identify the ESTs or full-length cDNA, the coding regions of GRAS genes were searched against the non-mouse and non-human EST databases of GenBank with BLASTN. Thirdly, we try to find out whether these genes possess real promoters by PlantCARE database (http://bioinformatics.psb.ugent.be/webtools/plantcare/html/). A total of 1500-bp nucleotide sequences upstream of the translation initiation codon for all GRAS genes were subjected to search for in-silico analysis. Those genes that contain general cis-acting elements of eukaryotes, such as TATA-box, CAAT-box, were not considered as pseudogenes.

### Alignment, phylogenetic analysis, and gene structure prediction

The identified GRAS proteins were aligned using the MUSCLE program [61] with the default parameters. The unrooted phylogenetic trees were inferred by three different analysis (neighbor-joining, maximum-likelihood, and Minimum-Evolution) using MEGA5.0 and the reliability of interior branches was assessed with 1000-bootstrap resampling [62,63]. Other motifs in the GRAS family, except the GRAS domain, were identified statistically using MEME with default settings. The number for the maximum number of motifs to find was 7. The analysis of the exon–intron gene structure of predicted GRAS genes was carried out using Gene Structure Display Server and comparison with the coding sequence of their corresponding genomic DNA sequences from Phytozome [64].

### Calculating Ks to date the duplication events of the GRAS gene family

GRAS genes showed a scattered distribution pattern on chromosomes. Several genes were clearly adjacent to one another based on their loci. Therefore, we focused on the process of segmental and tandem duplication. According to Schauser et al., an effective way to detect a segmental duplication event was to identify additional paralogous protein pairs in the neighborhood of each of the family members [25]. Segmental duplication information was collected from the Plant Genome Duplication Database (PGDD; http://chibba.agtec.uga.edu/duplication), and we selected the 200 kb parameter model to run the query.

Ks of duplication genes are expected to be similar over time, so in order to date segmental duplication events, we used Ks as the proxy for the time to estimate the dates of the segmental duplication events, and we chose Ks values ranging from 0 to 1. The approximate date of the duplication event was calculated using the mean K_S_ values from T = K_S_/2λ, assuming clocklike rates (λ) of synonymous substitution of 6.5 × 10^−9^ substitutions per synonymous site per year for monocots [65], 1.5 × 10^−8^ for *Arabidopsis* [26], and 6.1 × 10^−9^ for soybean [54]. However, an accurate λ for *P. patens* had not been found.

### Estimation of functional divergence

A maximum likelihood test of functional divergence was performed following Gu [66], using the DIVERGE v2.0 package [67], which estimates significant changes in the site-specific shift of evolutionary rate (Type I) or of amino acid properties (Type II) after the emergence of two paralogous sequences [35]. Type I designates amino acid configurations that are highly conserved in gene 1 but highly variable in gene 2, or vice versa, implying that these residues have experienced altered functional constraints [35,66]. Type II designates amino acid configurations that are highly conserved in both genes but whose biochemical properties are very different, implying that these residues may be responsible for functional specification [35]. The coefficients of Type I and Type II functional divergence (θ_I_ and θ_II_) between any two interesting clusters were calculated. A value for θ_I_ or θ_II_ that was significantly greater than 0 indicates that site-specific altered selective constraints were present (i.e., the types are experiencing different evolutionary rates) or a radical shift in amino acid physiochemical properties had occurred (e.g., positive versus negative charge) after gene duplication and/or speciation [35,66].

### Adaptive evolution analysis

Positive selection was identified using the CODEML program contained in the PAML v4.4 software package [37], using the site-specific model and the branch-site model. In the site-specific model, the non-synonymous substitution rate (dN) is higher than the synonymous rate (dS); when the ratio ω (dN/dS) is higher than 1, it represents evidence for positive selection at the molecular level. In the analysis, two pairs of models were chosen to identify positively selected sites using the BEB [68] estimation method. Model M0 assumed a single ω ratio for all sites. Model M3 allowed three unconstrained ω categories (ω < 1, purifying selection; ω = 1 neutral or positive selection) for each site. Models M0 (one ratio) and M3 (discrete) were compared, using a test for heterogeneity between codon sites in the dN/dS ratio value, ω. Model M7 was a null test for positive selection, assuming a Beta distribution with ω between 0 and 1. Model M8 added an extra class with the same ratio ω [69]. The comparison of M7 (beta) with M8 (beta + ω > 1) is the most stringent test of positive selection [70]. Finally, the likelihood ratio test was used to determine whether the ω ratios differed among lineages; that is, positive selection was indicated when the models that allow for selection (M3 and M8) were significantly better than the null model (no selection).

The branch-site method assumes that the branches of the phylogenetic tree are divided a *priori* into foreground and background lineages and that the ω ratio varies between codon sites. There are four site classes in the sequence. The first class of sites is highly conserved throughout the tree with 0 < ω_0_ < 1. The second class includes codons that are evolving neutrally throughout the tree with ω_1_ = 1. In the third and fourth classes, the background lineages are conserved or neutral, but positive selection on the foreground branches with ω_2_ > 1; that is, only foreground lineages experience positive selection. The likelihood ratio test was calculated using the BEB estimation method [68].

### Extraction of microarray data or RNA-Seq atlas

The expression microarray data for the genes studied in different developmental contexts in *Arabidopsis* were obtained from the TAIR (http://www.Arabidopsis.org/) experiment gene expression map of *Arabidopsis* development [71]. The expression values were determined from the following tissues: young leaf, flower stage (9, 10, 12, 15), seeds stage 3 with siliques, seeds stage 4 with siliques, seeds stage 5 with siliques, seeds stage 6 without siliques, seeds stage 7 without siliques, seeds stage 8 without siliques, seeds stage 9 without siliques, seeds stage 10 without siliques, root. Data were normalized by the GCOS method, TGT value of 100.

The Rice eFP Browser (http://www.bar.utoronto.ca/efprice/cgi-bin/efpWeb.cgi) tool was used to search the microarray data for rice. We also used experiment GSE6893, which was used to analyze the spatial and temporal gene expression in various tissues and various stages of reproductive development of rice [72]. The expression values from the following tissues and development stages were retrieved: young leaf, various stages of panicle (P1–P6), seedling root, and seed (S1–S5). Data were normalized by MAS.5.0 and the RMA method. The TGT value of 100 was used, and all tissues were sampled in triplicate.

RNA-Seq data were introduced to analyze the expression of GmGRAS genes. Data were normalized using a variation of the read s/Kb/Million method, and Z-score analysis was obtained from SoyBase (http://soybase.org/soyseq/). The expression analyses were performed in several organs: young leaf, flower, one-cm pod, pod shell (10 and 14 days after flowering), seed (10, 14, 21, 25, 28, 35, and 42 days after flowering), root, and nodule. Meanwhile, all heat maps were generated using the Gene Pattern program (http://www.broadinstitute.org/cancer/software/genepattern/).

## Availability of supporting data

- The data sets supporting this article are included in:
- Additional file 2. Protein sequences data of the GRAS gene subfamily in *Arabidopsis*, *Brachypodium distachyon*, rice, soybean, *Selaginella moellendorffii*, and *Physcomitrella patens*.
- Additional file 10. The phylogenetic tree data of the GRAS gene subfamily in *Arabidopsis*, *Brachypodium distachyon*, rice, soybean, *Selaginella moellendorffii*, and *Physcomitrella patens*.

## Additional files

Additional file 1:
**The number of the GRAS gene subfamily in** ***Arabidopsis*** **,** ***Brachypodium distachyon*** **, rice, soybean,** ***Selaginella moellendorffii*** **, and** ***Physcomitrella patens*** **.**
Additional file 2:
**Protein sequences data of the GRAS gene subfamily in** ***Arabidopsis*** **,** ***Brachypodium distachyon*** **, rice, soybean,** ***Selaginella moellendorffii*** **, and** ***Physcomitrella patens*** **.**
Additional file 3:
**Predicted AtGRAS genes and related information.** a.aa = amino acids; b. pI = isoelectric point of the deduced polypeptide; c.Mw = molecular weight; d. the relative position of introns are indicated by the red square. Additional file 4:
**Predicted BdGRAS genes and related information.** a.aa = amino acids; b. pI = isoelectric point of the deduced polypeptide; c.Mw = molecular weight; d. the relative position of introns are indicated by the red square. Additional file 5:
**Predicted OsGRAS genes and related information.** a.aa = amino acids; b. pI = isoelectric point of the deduced polypeptide; c.Mw = molecular weight; d. the relative position of introns are indicated by the red square. Additional file 6:
**Predicted GmGRAS genes and related information.** a.aa = amino acids; b. pI = isoelectric point of the deduced polypeptide; c.Mw = molecular weight; d. the relative position of introns are indicated by the red square. Additional file 7:
**Predicted SmGRAS genes and related information.** a.aa = amino acids; b. pI = isoelectric point of the deduced polypeptide; c.Mw = molecular weight; d. the relative position of introns are indicated by the red square. Additional file 8:
**Predicted PpGRAS genes and related information.** a.aa = amino acids; b. pI = isoelectric point of the deduced polypeptide; c.Mw = molecular weight; d. the relative position of introns are indicated by the red square. Additional file 9:
**Chromosome distribution of GRAS genes were from** ***Arabidopsis*** **,** ***Brachypodium distachyon*** **, rice, and soybean.** The size of a chromosome is indicated by its relative length. Red genes represent tandemly duplicated genes, and green circle represent segmentally duplicated genes. The location information and chromosome information were obtained from Phytozome. The figure was produced using the MapInspector program. Additional file 10:
**The phylogenetic tree data of the GRAS gene subfamily in** ***Arabidopsis*** **,** ***Brachypodium distachyon*** **, rice, soybean,** ***Selaginella moellendorffii*** **, and** ***Physcomitrella patens*** **.**
Additional file 11:
**The ML Phylogenetic tree of GRAS proteins among** ***Arabidopsis*** **,** ***Brachypodium distachyon*** **, rice, soybean,** ***Selaginella moellendorffii*** **, and** ***Physcomitrella patens*** **.** The major clusters of orthologous genes are shown in different colors: group I = purple, group II = dark blue, group III = yellow, group IV = light green, group V = pink, group VI = dark green, and group VII = light blue. The scale bar corresponds to 0.1 estimated amino acid substitutions per site. Additional file 12:
**The ME Phylogenetic tree of GRAS proteins among** ***Arabidopsis*** **,** ***Brachypodium distachyon*** **, rice, soybean,** ***Selaginella moellendorffii*** **, and** ***Physcomitrella patens*** **.** The major clusters of orthologous genes are shown in different colors: group I = purple, group II = dark blue, group III = yellow, group IV = light green, group V = pink, group VI = dark green, and group VII = light blue. The scale bar corresponds to 0.1 estimated amino acid substitutions per site. Additional file 13:
**Multiple sequence alignment of GRAS proteins in seven groups.** Multiple sequence alignment (Corpet 1988) was applied to do complete alignment of conserved GRAS domain residues. GRAS proteins share five conserved motifs: LHRI, VHIID, LHRII, PFYRE, and SAW motif. Green arrow represent 16 critical amino acid residues responsible for positive selection and two types of functional divergence. Additional file 14:
**Multiple sequence alignment of GRAS proteins in six species.** Multiple sequence alignment (Corpet 1988) was applied to do complete alignment of conserved GRAS domain residues. GRAS proteins share five conserved motifs: LHRI, VHIID, LHRII, PFYRE, and SAW motif. Additional file 15:
**Schematic distribution of conserved motifs identified by means of MEME software among defined gene clusters.** Position of each identified motif in all GRAS proteins represented in parenthesis. The highlighted with blue and red represents the conserved GRAS and DELLA domain, respectively. Additional file 16:
**Multilevel consensus sequences for the MEME defined motifs observed among different GRAS proteins from Arabidopsis, Brachypodium distachyon, rice, and soybean.**
Additional file 17:
**The relationships between amino acid sites under positive selection and two types functional divergence.**
Additional file 18:
**Parameters estimation and likelihood ratio tests for the site-specific model in Arabidopsis.** Note: *p < 0.05 and **p < 0.01 (*x* ^2^ test). a ω was estimated under model M0,M3,M7, and M8; p and q are the parameters of the beta distribution. b The number of amino acid sites estimated to have undergone positive selection. Additional file 19:
**Parameters estimation and likelihood ratio tests for the site-specific model in** ***Brachypodium distachyon*** **.** Note: *p < 0.05 and **p < 0.01 (*x* ^2^ test). a ω was estimated under model M0,M3,M7, and M8; p and q are the parameters of the beta distribution. b The number of amino acid sites estimated to have undergone positive selection. Additional file 20:
**Parameters estimation and likelihood ratio tests for the site-specific model in rice.** Note: *p < 0.05 and **p < 0.01 (*x* ^2^ test). a ω was estimated under model M0,M3,M7, and M8; p and q are the parameters of the beta distribution. b The number of amino acid sites estimated to have undergone positive selection. Additional file 21:
**Parameters estimation and likelihood ratio tests for the site-specific model in soybean.** Note: *p < 0.05 and **p < 0.01 (*x* ^2^ test). a ω was estimated under model M0,M3,M7, and M8; p and q are the parameters of the beta distribution. b The number of amino acid sites estimated to have undergone positive selection. Additional file 22:
**Parameters estimation and likelihood ratio tests for the site-specific model in** ***Selaginella moellendorffii*** **.** Note: *p < 0.05 and **p < 0.01 (*x* ^2^ test). a ω was estimated under model M0,M3,M7, and M8; p and q are the parameters of the beta distribution. b The number of amino acid sites estimated to have undergone positive selection. Additional file 23:
**Parameters estimation and likelihood ratio tests for the site-specific model in** ***Physcomitrella patens*** **.** Note: *p < 0.05 and **p < 0.01 (*x* ^2^ test). a ω was estimated under model M0,M3,M7, and M8; p and q are the parameters of the beta distribution. b The number of amino acid sites estimated to have undergone positive selection, and amino acids refer to Pp1s84_112V6 sequence. Additional file 24:
**Expression of the GRAS genes in various organs of Arabidopsis.** Gene names are displayed to the right of each row. The color scheme used to represent expression level is red/blue: blue boxes indicate a low expression, red boxes indicate a high expression. Additional file 25:
**Expression of the GRAS genes in various organs of rice.** Gene names are displayed to the right of each row. The color scheme used to represent expression level is red/blue: blue boxes indicate a low expression, red boxes indicate a high expression. Additional file 26:
**Expression of the GRAS genes in various organs of soybean.** Gene names are displayed to the right of each row. The color scheme used to represent expression level is red/blue: blue boxes indicate a low expression, red boxes indicate a high expression.
