# Supplementary material for: Unusual tandem expansion and positive selection in subgroups of the plant GRAS transcription factor superfamily
Source: BMC Plant Biol. 2014 Dec 19;14:373. doi: 10.1186/s12870-014-0373-5 (PMC4279901; doi:10.1186/s12870-014-0373-5)
Supplement: Additional file 1: — The number of the GRAS gene subfamily in Arabidopsis , Brachypodium distachyon , rice, soybean, Selaginella moellendorffii , and Physcomitrella patens . [file 12870_2014_373_MOESM1_ESM.doc]

|  | Group | Group | Group | Group | Group | Group | Group | Group | total |
| --- | --- | --- | --- | --- | --- | --- | --- | --- | --- |
|  | I | II | III | IV | Va | Vb | VI | VII |  |
| *Arabidopsis* | 6 | 7 | 3 | 5 | 3 | 1 | 3 | 5 | 33 |
| soybean | 21 | 25 | 12 | 13 | 13 | 0 | 7 | 15 | 106 |
| *Brachypodium distachyon* | 5 | 13 | 4 | 6 | 2 | 4 | 3 | 7 | 44 |
| rice | 7 | 10 | 4 | 5 | 9 | 2 | 3 | 7 | 47 |
| *Physcomitrella patens* | 2 | 7 | 8 | 2 | 5 | 8 | 5 | 1 | 38 |
| *Selaginella moellendorffii* | 2 | 5 | 1 | 3 | 2 | 4 | 2 | 2 | 21 |
| total | 43 | 67 | 32 | 34 | 34 | 19 | 23 | 37 | 289 |

**Additional file 1**. **The number of the seven GRAS subfamilies in *Arabidopsis*, *Brachypodium distachyon*, rice, soybean, *Selaginella moellendorffii*, and *Physcomitrella patens*.**
