# Supplementary material for: Unusual tandem expansion and positive selection in subgroups of the plant GRAS transcription factor superfamily
Source: BMC Plant Biol. 2014 Dec 19;14:373. doi: 10.1186/s12870-014-0373-5 (PMC4279901; doi:10.1186/s12870-014-0373-5)
Supplement: Additional file 2: — Protein sequences data of the GRAS gene subfamily in Arabidopsis , Brachypodium distachyon , rice, soybean, Selaginella moellendorffii , and Physcomitrella patens . [file 12870_2014_373_MOESM2_ESM.doc]

>AT1G63100

MLAGCSSSSLLSPTRRLRSEAVAATSATVSAHFPMNTQRLDLPCSSSFSRKETPSSRPLGRSISLDNSNNNNNKPIERKTKTSGCSLKQNIKLPPLATTR

GNGEGFSWNNDNNNRGKSLKRLAEEDESCLSRAKRTKCENEGGFWFEHFTGQDSSSPALPFSLTCSGDDEEKVCFVPSEVISQPLPNWVDSVITELAGIG

DKDVESSLPAAVKEASGGSSTSASSESRSLSHRVPEPTNGSRNPYSHRGATEERTTGNINNNNNRNDLQRDFELVNLLTGCLDAIRSRNIAAINHFIART

GDLASPRGRTPMTRLIAYYIEALALRVARMWPHIFHIAPPREFDRTVEDESGNALRFLNQVTPIPKFIHFTANEMLLRAFEGKERVHIIDFDIKQGLQWP

SFFQSLASRINPPHHVRITGIGESKLELNETGDRLHGFAEAMNLQFEFHPVVDRLEDVRLWMLHVKEGESVAVNCVMQMHKTLYDGTGAAIRDFLGLIRS

TNPIALVLAEQEAEHNSEQLETRVCNSLKYYSAMFDAIHTNLATDSLMRVKVEEMLFGREIRNIVACEGSHRQERHVGFRHWRRMLEQLGFRSLGVSERE

VLQSKMLLRMYGSDNEGFFNVERSDEDNGGEGGRGGGVTLRWSEQPLYTISAWTTGGN

>AT3G54220

MAESGDFNGGQPPPHSPLRTTSSGSSSSNNRGPPPPPPPPLVMVRKRLASEMSSNPDYNNSSRPPRRVSHLLDSNYNTVTPQQPPSLTAAATVSSQPNPP

LSVCGFSGLPVFPSDRGGRNVMMSVQPMDQDSSSSSASPTVWVDAIIRDLIHSSTSVSIPQLIQNVRDIIFPCNPNLGALLEYRLRSLMLLDPSSSSDPS

PQTFEPLYQISNNPSPPQQQQQHQQQQQQHKPPPPPIQQQERENSSTDAPPQPETVTATVPAVQTNTAEALRERKEEIKRQKQDEEGLHLLTLLLQCAEA

VSADNLEEANKLLLEISQLSTPYGTSAQRVAAYFSEAMSARLLNSCLGIYAALPSRWMPQTHSLKMVSAFQVFNGISPLVKFSHFTANQAIQEAFEKEDS

VHIIDLDIMQGLQWPGLFHILASRPGGPPHVRLTGLGTSMEALQATGKRLSDFADKLGLPFEFCPLAEKVGNLDTERLNVRKREAVAVHWLQHSLYDVTG

SDAHTLWLLQRLAPKVVTVVEQDLSHAGSFLGRFVEAIHYYSALFDSLGASYGEESEERHVVEQQLLSKEIRNVLAVGGPSRSGEVKFESWREKMQQCGF

KGISLAGNAATQATLLLGMFPSDGYTLVDDNGTLKLGWKDLSLLTASAWTPRS

>AT1G66350

MKREHNHRESSAGEGGSSSMTTVIKEEAAGVDELLVVLGYKVRSSDMADVAHKLEQLEMVLGDGISNLSDETVHYNPSDLSGWVESMLSDLDPTRIQEKP

DSEYDLRAIPGSAVYPRDEHVTRRSKRTRIESELSSTRSVVVLDSQETGVRLVHALLACAEAVQQNNLKLADALVKHVGLLASSQAGAMRKVATYFAEGL

ARRIYRIYPRDDVALSSFSDTLQIHFYESCPYLKFAHFTANQAILEVFATAEKVHVIDLGLNHGLQWPALIQALALRPNGPPDFRLTGIGYSLTDIQEVG

WKLGQLASTIGVNFEFKSIALNNLSDLKPEMLDIRPGLESVAVNSVFELHRLLAHPGSIDKFLSTIKSIRPDIMTVVEQEANHNGTVFLDRFTESLHYYS

SLFDSLEGPPSQDRVMSELFLGRQILNLVACEGEDRVERHETLNQWRNRFGLGGFKPVSIGSNAYKQASMLLALYAGADGYNVEENEGCLLLGWQTRPLI

ATSAWRINRVE

>AT2G01570

MKRDHHQFQGRLSNHGTSSSSSSISKDKMMMVKKEEDGGGNMDDELLAVLGYKVRSSEMAEVALKLEQLETMMSNVQEDGLSHLATDTVHYNPSELYSWL

DNMLSELNPPPLPASSNGLDPVLPSPEICGFPASDYDLKVIPGNAIYQFPAIDSSSSSNNQNKRLKSCSSPDSMVTSTSTGTQIGGVIGTTVTTTTTTTT

AAGESTRSVILVDSQENGVRLVHALMACAEAIQQNNLTLAEALVKQIGCLAVSQAGAMRKVATYFAEALARRIYRLSPPQNQIDHCLSDTLQMHFYETCP

YLKFAHFTANQAILEAFEGKKRVHVIDFSMNQGLQWPALMQALALREGGPPTFRLTGIGPPAPDNSDHLHEVGCKLAQLAEAIHVEFEYRGFVANSLADL

DASMLELRPSDTEAVAVNSVFELHKLLGRPGGIEKVLGVVKQIKPVIFTVVEQESNHNGPVFLDRFTESLHYYSTLFDSLEGVPNSQDKVMSEVYLGKQI

CNLVACEGPDRVERHETLSQWGNRFGSSGLAPAHLGSNAFKQASMLLSVFNSGQGYRVEESNGCLMLGWHTRPLITTSAWKLSTAAY

>AT1G50420

MVAMFQEDNGTSSVASSPLQVFSTMSLNRPTLLASSSPFHCLKDLKPEERGLYLIHLLLTCANHVASGSLQNANAALEQLSHLASPDGDTMQRIAAYFTE

ALANRILKSWPGLYKALNATQTRTNNVSEEIHVRRLFFEMFPILKVSYLLTNRAILEAMEGEKMVHVIDLDASEPAQWLALLQAFNSRPEGPPHLRITGV

HHQKEVLEQMAHRLIEEAEKLDIPFQFNPVVSRLDCLNVEQLRVKTGEALAVSSVLQLHTFLASDDDLMRKNCALRFQNNPSGVDLQRVLMMSHGSAAEA

RENDMSNNNGYSPSGDSASSLPLPSSGRTDSFLNAIWGLSPKVMVVTEQDSDHNGSTLMERLLESLYTYAALFDCLETKVPRTSQDRIKVEKMLFGEEIK

NIISCEGFERRERHEKLEKWSQRIDLAGFGNVPLSYYAMLQARRLLQGCGFDGYRIKEESGCAVICWQDRPLYSVSAWRCRK

>AT1G14920

MKRDHHHHHHQDKKTMMMNEEDDGNGMDELLAVLGYKVRSSEMADVAQKLEQLEVMMSNVQEDDLSQLATETVHYNPAELYTWLDSMLTDLNPPSSNAEY

DLKAIPGDAILNQFAIDSASSSNQGGGGDTYTTNKRLKCSNGVVETTTATAESTRHVVLVDSQENGVRLVHALLACAEAVQKENLTVAEALVKQIGFLAV

SQIGAMRKVATYFAEALARRIYRLSPSQSPIDHSLSDTLQMHFYETCPYLKFAHFTANQAILEAFQGKKRVHVIDFSMSQGLQWPALMQALALRPGGPPV

FRLTGIGPPAPDNFDYLHEVGCKLAHLAEAIHVEFEYRGFVANTLADLDASMLELRPSEIESVAVNSVFELHKLLGRPGAIDKVLGVVNQIKPEIFTVVE

QESNHNSPIFLDRFTESLHYYSTLFDSLEGVPSGQDKVMSEVYLGKQICNVVACDGPDRVERHETLSQWRNRFGSAGFAAAHIGSNAFKQASMLLALFNG

GEGYRVEESDGCLMLGWHTRPLIATSAWKLSTN

>AT5G41920

MTTKRIDRDLPSSDDPSSAKRRIEFPEETLENDGAAAIKLLSLLLQCAEYVATDHLREASTLLSEISEICSPFGSSPERVVAYFAQALQTRVISSYLSGA

CSPLSEKPLTVVQSQKIFSALQTYNSVSPLIKFSHFTANQAIFQALDGEDSVHIIDLDVMQGLQWPALFHILASRPRKLRSIRITGFGSSSDLLASTGRR

LADFASSLNLPFEFHPIEGIIGNLIDPSQLATRQGEAVVVHWMQHRLYDVTGNNLETLEILRRLKPNLITVVEQELSYDDGGSFLGRFVEALHYYSALFD

ALGDGLGEESGERFTVEQIVLGTEIRNIVAHGGGRRKRMKWKEELSRVGFRPVSLRGNPATQAGLLLGMLPWNGYTLVEENGTLRLGWKDLSLLTASAWK

SQPFD

>AT5G17490

MKRSHQETSVEEEAPSMVEKLENGCGGGGDDNMDEFLAVLGYKVRSSDMADVAQKLEQLEMVLSNDIASSSNAFNDTVHYNPSDLSGWAQSMLSDLNYYP

DLDPNRICDLRPITDDDECCSSNSNSNKRIRLGPWCDSVTSESTRSVVLIEETGVRLVQALVACAEAVQLENLSLADALVKRVGLLAASQAGAMGKVATY

FAEALARRIYRIHPSAAAIDPSFEEILQMNFYDSCPYLKFAHFTANQAILEAVTTSRVVHVIDLGLNQGMQWPALMQALALRPGGPPSFRLTGVGNPSNR

EGIQELGWKLAQLAQAIGVEFKFNGLTTERLSDLEPDMFETRTESETLVVNSVFELHPVLSQPGSIEKLLATVKAVKPGLVTVVEQEANHNGDVFLDRFN

EALHYYSSLFDSLEDGVVIPSQDRVMSEVYLGRQILNLVATEGSDRIERHETLAQWRKRMGSAGFDPVNLGSDAFKQASLLLALSGGGDGYRVEENDGSL

MLAWQTKPLIAASAWKLAAELRR

>AT3G03450

MKRGYGETWDPPPKPLPASRSGEGPSMADKKKADDDNNNSNMDDELLAVLGYKVRSSEMAEVAQKLEQLEMVLSNDDVGSTVLNDSVHYNPSDLSNWVES

MLSELNNPASSDLDTTRSCVDRSEYDLRAIPGLSAFPKEEEVFDEEASSKRIRLGSWCESSDESTRSVVLVDSQETGVRLVHALVACAEAIHQENLNLAD

ALVKRVGTLAGSQAGAMGKVATYFAQALARRIYRDYTAETDVCAAVNPSFEEVLEMHFYESCPYLKFAHFTANQAILEAVTTARRVHVIDLGLNQGMQWP

ALMQALALRPGGPPSFRLTGIGPPQTENSDSLQQLGWKLAQFAQNMGVEFEFKGLAAESLSDLEPEMFETRPESETLVVNSVFELHRLLARSGSIEKLLN

TVKAIKPSIVTVVEQEANHNGIVFLDRFNEALHYYSSLFDSLEDSYSLPSQDRVMSEVYLGRQILNVVAAEGSDRVERHETAAQWRIRMKSAGFDPIHLG

SSAFKQASMLLSLYATGDGYRVEENDGCLMIGWQTRPLITTSAWKLA

>AT1G55580

MLTSFKSSSSSSEDATATTTENPPPLCIASSSAATSASHHLRRLLFTAANFVSQSNFTAAQNLLSILSLNSSPHGDSTERLVHLFTKALSVRINRQQQDQ

TAETVATWTTNEMTMSNSTVFTSSVCKEQFLFRTKNNNSDFESCYYLWLNQLTPFIRFGHLTANQAILDATETNDNGALHILDLDISQGLQWPPLMQALA

ERSSNPSSPPPSLRITGCGRDVTGLNRTGDRLTRFADSLGLQFQFHTLVIVEEDLAGLLLQIRLLALSAVQGETIAVNCVHFLHKIFNDDGDMIGHFLSA

IKSLNSRIVTMAEREANHGDHSFLNRFSEAVDHYMAIFDSLEATLPPNSRERLTLEQRWFGKEILDVVAAEETERKQRHRRFEIWEEMMKRFGFVNVPIG

SFALSQAKLLLRLHYPSEGYNLQFLNNSLFLGWQNRPLFSVSSWK

>AT5G66770

MAYMCTDSGNLMAIAQQVIKQKQQQEQQQQQHHQDHQIFGINPLSLNPWPNTSLGFGLSGSAFPDPFQVTGGGDSNDPGFPFPNLDHHHATTTGGGFRLS

DFGGGTGGGEFESDEWMETLISGGDSVADGPDCDTWHDNPDYVIYGPDPFDTYPSRLSVQPSDLNRVIDTSSPLPPPTLWPPSSPLSIPPLTHESPTKED

PETNDSEDDDFDLEPPLLKAIYDCARISDSDPNEASKTLLQIRESVSELGDPTERVAFYFTEALSNRLSPNSPATSSSSSSTEDLILSYKTLNDACPYSK

FAHLTANQAILEATEKSNKIHIVDFGIVQGIQWPALLQALATRTSGKPTQIRVSGIPAPSLGESPEPSLIATGNRLRDFAKVLDLNFDFIPILTPIHLLN

GSSFRVDPDEVLAVNFMLQLYKLLDETPTIVDTALRLAKSLNPRVVTLGEYEVSLNRVGFANRVKNALQFYSAVFESLEPNLGRDSEERVRVERELFGRR

ISGLIGPEKTGIHRERMEEKEQWRVLMENAGFESVKLSNYAVSQAKILLWNYNYSNLYSIVESKPGFISLAWNDLPLLTLSSWR

>AT3G50650

MAYMCTDSGNLMAIAQQLIKQKQQQQSQHQQQEEQEQEPNPWPNPSFGFTLPGSGFSDPFQVTNDPGFHFPHLEHHQNAAVASEEFDSDEWMESLINGGD

ASQTNPDFPIYGHDPFVSFPSRLSAPSYLNRVNKDDSASQQLPPPPASTAIWSPSPPSPQHPPPPPPQPDFDLNQPIFKAIHDYARKPETKPDTLIRIKE

SVSESGDPIQRVGYYFAEALSHKETESPSSSSSSSLEDFILSYKTLNDACPYSKFAHLTANQAILEATNQSNNIHIVDFGIFQGIQWSALLQALATRSSG

KPTRIRISGIPAPSLGDSPGPSLIATGNRLRDFAAILDLNFEFYPVLTPIQLLNGSSFRVDPDEVLVVNFMLELYKLLDETATTVGTALRLARSLNPRIV

TLGEYEVSLNRVEFANRVKNSLRFYSAVFESLEPNLDRDSKERLRVERVLFGRRIMDLVRSDDDNNKPGTRFGLMEEKEQWRVLMEKAGFEPVKPSNYAV

SQAKLLLWNYNYSTLYSLVESEPGFISLAWNNVPLLTVSSWR

>AT1G50600

MRLSVFIIPLVESRQASGIINKQSTSLLIRFSLYLEASISTKSFFSKSQRISQTQSPICLSANYYQPDNLDMEATQKHMIQEGSSMFYHQPSSVKQMDLS

VQTFDSYCTLESSSGTKSHPCLNNKNNSSSTTSFSSNESPISQANNNNLSRFNNHSPEENNNSPLSGSSATNTNETELSLMLKDLETAMMEPDVDNSYNN

QGGFGQQHGVVSSAMYRSMEMISRGDLKGVLYECAKAVENYDLEMTDWLISQLQQMVSVSGEPVQRLGAYMLEGLVARLASSGSSIYKALRCKDPTGPEL

LTYMHILYEACPYFKFGYESANGAIAEAVKNESFVHIIDFQISQGGQWVSLIRALGARPGGPPNVRITGIDDPRSSFARQGGLELVGQRLGKLAEMCGVP

FEFHGAALCCTEVEIEKLGVRNGEALAVNFPLVLHHMPDESVTVENHRDRLLRLVKHLSPNVVTLVEQEANTNTAPFLPRFVETMNHYLAVFESIDVKLA

RDHKERINVEQHCLAREVVNLIACEGVEREERHEPLGKWRSRFHMAGFKPYPLSSYVNATIKGLLESYSEKYTLEERDGALYLGWKNQPLITSCAWR

>AT5G48150

MYKQPRQELEAYYFEPNSVEKLRYLPVNNSRKRFCTLEPFPDSPPYNALSTATYDDTCGSCVTDELNDFKHKIREIETVMMGPDSLDLLVDCTDSFDSTA

SQEINGWRSTLEAISRRDLRADLVSCAKAMSENDLMMAHSMMEKLRQMVSVSGEPIQRLGAYLLEGLVAQLASSGSSIYKALNRCPEPASTELLSYMHIL

YEVCPYFKFGYMSANGAIAEAMKEENRVHIIDFQIGQGSQWVTLIQAFAARPGGPPRIRITGIDDMTSAYARGGGLSIVGNRLAKLAKQFNVPFEFNSVS

VSVSEVKPKNLGVRPGEALAVNFAFVLHHMPDESVSTENHRDRLLRMVKSLSPKVVTLVEQESNTNTAAFFPRFMETMNYYAAMFESIDVTLPRDHKQRI

NVEQHCLARDVVNIIACEGADRVERHELLGKWRSRFGMAGFTPYPLSPLVNSTIKSLLRNYSDKYRLEERDGALYLGWMHRDLVASCAWK

>AT2G04890

MDNVRGSIMLQPLPEIAESIDDAICHELSMWPDDAKDLLLIVEAISRGDLKLVLVACAKAVSENNLLMARWCMGELRGMVSISGEPIQRLGAYMLEGLVA

RLAASGSSIYKSLQSREPESYEFLSYVYVLHEVCPYFKFGYMSANGAIAEAMKDEERIHIIDFQIGQGSQWIALIQAFAARPGGAPNIRITGVGDGSVLV

TVKKRLEKLAKKFDVPFRFNAVSRPSCEVEVENLDVRDGEALGVNFAYMLHHLPDESVSMENHRDRLLRMVKSLSPKVVTLVEQECNTNTSPFLPRFLET

LSYYTAMFESIDVMLPRNHKERINIEQHCMARDVVNIIACEGAERIERHELLGKWKSRFSMAGFEPYPLSSIISATIRALLRDYSNGYAIEERDGALYLG

WMDRILVSSCAWK

>AT2G29060

MGSYSAGFPGSLDWFDFPGLGNGSYLNDQPLLDIGSVPPPLDPYPQQNLASADADFSDSVLKYISQVLMEEDMEDKPCMFHDALSLQAAEKSLYEALGEK

YPVDDSDQPLTTTTSLAQLVSSPGGSSYASSTTTTSSDSQWSFDCLENNRPSSWLQTPIPSNFIFQSTSTRASSGNAVFGSSFSGDLVSNMFNDTDLALQ

FKKGMEEASKFLPKSSQLVIDNSVPNRLTGKKSHWREEEHLTEERSKKQSAIYVDETDELTDMFDNILIFGEAKEQPVCILNESFPKEPAKASTFSKSPK

GEKPEASGNSYTKETPDLRTMLVSCAQAVSINDRRTADELLSRIRQHSSSYGDGTERLAHYFANSLEARLAGIGTQVYTALSSKKTSTSDMLKAYQTYIS

VCPFKKIAIIFANHSIMRLASSANAKTIHIIDFGISDGFQWPSLIHRLAWRRGSSCKLRITGIELPQRGFRPAEGVIETGRRLAKYCQKFNIPFEYNAIA

QKWESIKLEDLKLKEGEFVAVNSLFRFRNLLDETVAVHSPRDTVLKLIRKIKPDVFIPGILSGSYNAPFFVTRFREVLFHYSSLFDMCDTNLTREDPMRV

MFEKEFYGREIMNVVACEGTERVERPESYKQWQARAMRAGFRQIPLEKELVQKLKLMVESGYKPKEFDVDQDCHWLLQGWKGRIVYGSSIWVPL

>AT4G17230

MQTSQKHHSAAGLHMLYPQVYCSPQFQAKDNKGFSDIPSKENFFTLESSTASGSLPSYDSPSVSITSGRSPFSPQGSQSCISDLHHSPDNVYGSPLSGVS

SLAYDEAGVKSKIRELEVSLLSGDTKVEEFSGFSPAAGKSWNWDELLALTPQLDLKEVLVEAARAVADGDFATAYGFLDVLEQMVSVSGSPIQRLGTYMA

EGLRARLEGSGSNIYKSLKCNEPTGRELMSYMSVLYEICPYWKFAYTTANVEILEAIAGETRVHIIDFQIAQGSQYMFLIQELAKRPGGPPLLRVTGVDD

SQSTYARGGGLSLVGERLATLAQSCGVPFEFHDAIMSGCKVQREHLGLEPGFAVVVNFPYVLHHMPDESVSVENHRDRLLHLIKSLSPKLVTLVEQESNT

NTSPFLSRFVETLDYYTAMFESIDAARPRDDKQRISAEQHCVARDIVNMIACEESERVERHEVLGIWRVRMMMAGFTGWPVSTSAAFAASEMLKAYDKNY

KLGGHEGALYLFWKRRPMATCSVWKPNPN

>AT1G21450

MVEQTVVREHIKARVMSLVRSAEPSSYRNPKLYTLNENGNNNGVSSAQIFDPDRSKNPCLTDDSYPSQSYEKYFLDSPTDEFVQHPIGSGASVSSFGSLD

SFPYQSRPVLGCSMEFQLPLDSTSTSSTRLLGDYQAVSYSPSMDVVEEFDDEQMRSKIQELERALLGDEDDKMVGIDNLMEIDSEWSYQNESEQHQDSPK

ESSSADSNSHVSSKEVVSQATPKQILISCARALSEGKLEEALSMVNELRQIVSIQGDPSQRIAAYMVEGLAARMAASGKFIYRALKCKEPPSDERLAAMQ

VLFEVCPCFKFGFLAANGAILEAIKGEEEVHIIDFDINQGNQYMTLIRSIAELPGKRPRLRLTGIDDPESVQRSIGGLRIIGLRLEQLAEDNGVSFKFKA

MPSKTSIVSPSTLGCKPGETLIVNFAFQLHHMPDESVTTVNQRDELLHMVKSLNPKLVTVVEQDVNTNTSPFFPRFIEAYEYYSAVFESLDMTLPRESQE

RMNVERQCLARDIVNIVACEGEERIERYEAAGKWRARMMMAGFNPKPMSAKVTNNIQNLIKQQYCNKYKLKEEMGELHFCWEEKSLIVASAWR

>AT1G07530

MGSYPDGFPGSMDELDFNKDFDLPPSSNQTLGLANGFYLDDLDFSSLDPPEAYPSQNNNNNNINNKAVAGDLLSSSSDDADFSDSVLKYISQVLMEEDME

EKPCMFHDALALQAAEKSLYEALGEKYPSSSSASSVDHPERLASDSPDGSCSGGAFSDYASTTTTTSSDSHWSVDGLENRPSWLHTPMPSNFVFQSTSRS

NSVTGGGGGGNSAVYGSGFGDDLVSNMFKDDELAMQFKKGVEEASKFLPKSSQLFIDVDSYIPMNSGSKENGSEVFVKTEKKDETEHHHHHSYAPPPNRL

TGKKSHWRDEDEDFVEERSNKQSAVYVEESELSEMFDKILVCGPGKPVCILNQNFPTESAKVVTAQSNGAKIRGKKSTSTSHSNDSKKETADLRTLLVLC

AQAVSVDDRRTANEMLRQIREHSSPLGNGSERLAHYFANSLEARLAGTGTQIYTALSSKKTSAADMLKAYQTYMSVCPFKKAAIIFANHSMMRFTANANT

IHIIDFGISYGFQWPALIHRLSLSRPGGSPKLRITGIELPQRGFRPAEGVQETGHRLARYCQRHNVPFEYNAIAQKWETIQVEDLKLRQGEYVVVNSLFR

FRNLLDETVLVNSPRDAVLKLIRKINPNVFIPAILSGNYNAPFFVTRFREALFHYSAVFDMCDSKLAREDEMRLMYEKEFYGREIVNVVACEGTERVERP

ETYKQWQARLIRAGFRQLPLEKELMQNLKLKIENGYDKNFDVDQNGNWLLQGWKGRIVYASSLWVPSSS

>AT4G08250

MNYPYEDFLDLFFSTHTDPLATAASTSSNGYSLNDLDIDWDCDFRDVIESIMGDEGAMMEPESEAVPMLHDQEGLCNSASTGLSVADGVSFGEPKTDESK

GLRLVHLLVAAADASTGANKSRELTRVILARLKDLVSPGDRTNMERLAAHFTNGLSKLLERDSVLCPQQHRDDVYDQADVISAFELLQNMSPYVNFGYLT

ATQAILEAVKYERRIHIVDYDINEGVQWASLMQALVSRNTGPSAQHLRITALSRATNGKKSVAAVQETGRRLTAFADSIGQPFSYQHCKLDTNAFSTSSL

KLVRGEAVVINCMLHLPRFSHQTPSSVISFLSEAKTLNPKLVTLVHEEVGLMGNQGFLYRFMDLLHQFSAIFDSLEAGLSIANPARGFVERVFIGPWVAN

WLTRITANDAEVESFASWPQWLETNGFKPLEVSFTNRCQAKLLLSLFNDGFRVEELGQNGLVLGWKSRRLVSASFWASCQTNQ

>AT2G29065

MDPNFSESLNGFEYFDGNPNLLTDPMEDQYPPPSDTLLKYVSEILMEESNGDYKQSMFYDSLALRKTEEMLQQVITDSQNQSFSPADSLITNSWDASGSI

DESAYSADPQPVNEIMVKSMFSDAESALQFKKGVEEASKFLPNSDQWVINLDIERSERRDSVKEEMGLDQLRVKKNHERDFEEVRSSKQFASNVEDSKVT

DMFDKVLLLDGECDPQTLLDSEIQAIRSSKNIGEKGKKKKKKKSQVVDFRTLLTHCAQAISTGDKTTALEFLLQIRQQSSPLGDAGQRLAHCFANALEAR

LQGSTGPMIQTYYNALTSSLKDTAADTIRAYRVYLSSSPFVTLMYFFSIWMILDVAKDAPVLHIVDFGILYGFQWPMFIQSISDRKDVPRKLRITGIELP

QCGFRPAERIEETGRRLAEYCKRFNVPFEYKAIASQNWETIRIEDLDIRPNEVLAVNAGLRLKNLQDETGSEENCPRDAVLKLIRNMNPDVFIHAIVNGS

FNAPFFISRFKEAVYHYSALFDMFDSTLPRDNKERIRFEREFYGREAMNVIACEEADRVERPETYRQWQVRMVRAGFKQKTIKPELVELFRGKLKKWRYH

KDFVVDENSKWLLQGWKGRTLYASSCWVPA

>AT3G46600

MDAILPVPVDGFRFDTGSGSCCKPRNNLESGTTNRFTCFNESESQSNPSPTESKVCSDYLPVFKYINDMLMEEDLEGQSCMLEDSLALQAAERSFFEVLQ

DQTPISGDLEDGSLGNFSSITSLHQPEVSEESTRRYRHRDDDEDDDLESGRKSKLPAISTVDELAEKFEEVLLVCQKNDQGEATEKKTRHVKGSSNRYKQ

QKSDQPVDMRNLLMQCAQAVASFDQRRAFEKLKEIREHSSRHGDATQRLGYHFAEALEARITGTMTTPISATSSRTSMVDILKAYKGFVQACPTLIMCYF

TANRTINELASKATTLHIIDFGILYGFQWPCLIQALSKRDIGPPLLRVTGIELPQSGFRPSERVEETGRRLKRFCDKFNVPFEYSFIAKNWENITLDDLV

INSGETTVVNCILRLQYTPDETVSLNSPRDTALKLFRDINPDLFVFAEINGTYNSPFFLTRFREALFHCSSLFDMYETTLSEDDNCRTLVERELIIRDAM

SVIACEGSERFARPETYKQWQVRILRAGFRPAKLSKQIVKDGKEIVKERYHKDFVIDNDNHWMFQGWKGRVLYAVSCWKPAKK

>AT5G59450

MDALLQVSVDGFRFENGSGSCCKPRNNLESGNNLFPDFHESQNQSSPNDSPPTVCLDNSPVLKYINDMLMDEEDFVGISRDDLALQAAERSFYEIIQQQS

PESDQNTSSSSDQNSGDQDFCFPSTTTDSSALVSSGESQRKYRHRNDEEDDLENNRRNKQPAIFVSEMEELAVKLEHVLLVCKTNQEEEEERTVITKQST

PNRAGRAKGSSNKSKTHKTNTVDLRSLLTQCAQAVASFDQRRATDKLKEIRAHSSSNGDGTQRLAFYFAEALEARITGNISPPVSNPFPSSTTSMVDILK

AYKLFVHTCPIYVTDYFAANKSIYELAMKATKLHIVDFGVLYGFQWPCLLRALSKRPGGPPMLRVTGIELPQAGFRPSDRVEETGRRLKRFCDQFNVPFE

FNFIAKKWETITLDELMINPGETTVVNCIHRLQYTPDETVSLDSPRDTVLKLFRDINPDLFVFAEINGMYNSPFFMTRFREALFHYSSLFDMFDTTIHAE

DEYKNRSLLERELLVRDAMSVISCEGAERFARPETYKQWRVRILRAGFKPATISKQIMKEAKEIVRKRYHRDFVIDSDNNWMLQGWKGRVIYAFSCWKPA

EKFTNNNLNI

>AT1G07520

MESNYSGVVNGYDVSFLPTSIPDLGFGVPSSSDFDLRMDQYYHQPSIWVPDQDHHFSPPADEIDSENTLLKYVNQLLMEESLAEKQSIFYDSLALRQTEE

MLQQVISDSQTQSSIPNNSITTSSSSNSGDYSNSSNSSVRIENEVLFDNKHLGDSGVVSFPGSNMLRGGEQFGQPANEILVRSMFSDAESVLQFKRGLEE

ASKFLPNTDQWIFNLEPEMERVVPVKVEEGWSAISKTRKNHHEREEEEDDLEEARRRSKQFAVNEEDGKLTEMFDKVLLLDGECDPQIIEDGENGSSKAL

VKKGRAKKKSRAVDFRTLLTLCAQSVSAGDKITADDLLRQIRKQCSPVGDASQRLAHFFANALEARLEGSTGTMIQSYYDSISSKKRTAAQILKSYSVFL

SASPFMTLIYFFSNKMILDAAKDASVLHIVDFGILYGFQWPMFIQHLSKSNPGLRKLRITGIEIPQHGLRPTERIQDTGRRLTEYCKRFGVPFEYNAIAS

KNWETIKMEEFKIRPNEVLAVNAVLRFKNLRDVIPGEEDCPRDGFLKLIRDMNPNVFLSSTVNGSFNAPFFTTRFKEALFHYSALFDLFGATLSKENPER

IHFEGEFYGREVMNVIACEGVDRVERPETYKQWQVRMIRAGFKQKPVEAELVQLFREKMKKWGYHKDFVLDEDSNWFLQGWKGRILFSSSCWVPS

>AT4G37650

MDTLFRLVSLQQQQQSDSIITNQSSLSRTSTTTTGSPQTAYHYNFPQNDVVEECFNFFMDEEDLSSSSSHHNHHNHNNPNTYYSPFTTPTQYHPATSSTP

SSTAAAAALASPYSSSGHHNDPSAFSIPQTPPSFDFSANAKWADSVLLEAARAFSDKDTARAQQILWTLNELSSPYGDTEQKLASYFLQALFNRMTGSGE

RCYRTMVTAAATEKTCSFESTRKTVLKFQEVSPWATFGHVAANGAILEAVDGEAKIHIVDISSTFCTQWPTLLEALATRSDDTPHLRLTTVVVANKFVND

QTASHRMMKEIGNRMEKFARLMGVPFKFNIIHHVGDLSEFDLNELDVKPDEVLAINCVGAMHGIASRGSPRDAVISSFRRLRPRIVTVVEEEADLVGEEE

GGFDDEFLRGFGECLRWFRVCFESWEESFPRTSNERLMLERAAGRAIVDLVACEPSDSTERRETARKWSRRMRNSGFGAVGYSDEVADDVRALLRRYKEG

VWSMVQCPDAAGIFLCWRDQPVVWASAWRPT

>AT2G37650

MITEPSLTGISGMVNRNRLSGLPDQPSSHSFTPVTLYDGFNYNLSSDHINTVVAAPENSVFIREEEEEEDPADDFDFSDAVLGYISQMLNEEDMDDKVCM

LQESLDLEAAERSLYEAIGKKYPPSPERNLAFAERNSENLDRVVPGNYTGGDCIGFGNGGIKPLSSGFTLDFRNPQSCSSILSVPQSNGLITIYGDGIDE

SSKNNRENHQSVWLFRREIEEANRFNPEENELIVNFREENCVSKARKNSSRDEICVEEERSSKLPAVFGEDILRSDVVDKILVHVPGGESMKEFNALRDV

LKKGVEKKKASDAQGGKRRARGRGRGRGRGGGGGQNGKKEVVDLRSLLIHCAQAVAADDRRCAGQLLKQIRLHSTPFGDGNQRLAHCFANGLEARLAGTG

SQIYKGIVSKPRSAAAVLKAHQLFLACCPFRKLSYFITNKTIRDLVGNSQRVHVIDFGILYGFQWPTLIHRFSMYGSPKVRITGIEFPQPGFRPAQRVEE

TGQRLAAYAKLFGVPFEYKAIAKKWDAIQLEDLDIDRDEITVVNCLYRAENLHDESVKVESCRDTVLNLIGKINPDLFVFGIVNGAYNAPFFVTRFREAL

FHFSSIFDMLETIVPREDEERMFLEMEVFGREALNVIACEGWERVERPETYKQWHVRAMRSGLVQVPFDPSIMKTSLHKVHTFYHKDFVIDQDNRWLLQG

WKGRTVMALSVWKPESKA

>AT3G49950

MTKTRILNPTRFPSPKPLRGCGDANFMEQLLLHCATAIDSNDAALTHQILWVLNNIAPPDGDSTQRLTSAFLRALLSRAVSKTPTLSSTISFLPQADELH

RFSVVELAAFVDLTPWHRFGFIAANAAILTAVEGYSTVHIVDLSLTHCMQIPTLIDAMASRLNKPPPLLKLTVVSSSDHFPPFINISYEELGSKLVNFAT

TRNITMEFTIVPSTYSDGFSSLLQQLRIYPSSFNEALVVNCHMMLRYIPEEPLTSSSSSLRTVFLKQLRSLNPRIVTLIEEDVDLTSENLVNRLKSAFNY

FWIPFDTTDTFMSEQRRWYEAEISWKIENVVAKEGAERVERTETKRRWIERMREAEFGGVRVKEDAVADVKAMLEEHAVGWGMKKEDDDESLVLTWKGHS

VVFATVWVPI

>AT3G60630

MPLPFEQFQGKGVLGFLDSSSSPGYKIWANPEKLHGRVEEDLCFVVNNGGFSEPTSVLDSVRSPSPFVSSSTTTLSSSHGGPSGGGAAAATFSGADGKCD

QMGFEDLDGVLSGGSPGQEQSIFRLIMAGDVVDPGSEFVGFDIGSGSDPVIDNPNPLFGYGFPFQNAPEEEKFQISINPNPGFFSDPPSSPPAKRLNSGQ

PGSQHLQWVFPFSDPGHESHDPFLTPPKIAGEDQNDQDQSAVIIDQLFSAAAELTTNGGDNNPVLAQGILARLNHNLNNNNDDTNNNPKPPFHRAASYIT

EALHSLLQDSSLSPPSLSPPQNLIFRIAAYRAFSETSPFLQFVNFTANQTILESFEGFDRIHIVDFDIGYGGQWASLIQELAGKRNRSSSAPSLKITAFA

SPSTVSDEFELRFTEENLRSFAGETGVSFEIELLNMEILLNPTYWPLSLFRSSEKEAIAVNLPISSMVSGYLPLILRFLKQISPNVVVCSDRSCDRNNDA

PFPNGVINALQYYTSLLESLDSGNLNNAEAATSIERFCVQPSIQKLLTNRYRWMERSPPWRSLFGQCGFTPVTLSQTAETQAEYLLQRNPMRGFHLEKRQ

SSSPSLVLCWQRKELVTVSAWKC

>AT4G00150

MPLPFEEFQGKGISCFSSFSSSFPQPPSSPLLSHRKARGGEEEEEEVPAAEPTSVLDSLISPTSSSTVSSSHGGNSAVGGGGDATTDEQCGAIGLGDWEE

QVPHDHEQSILGLIMGDSTDPSLELNSILQTSPTFHDSDYSSPGFGVVDTGFGLDHHSVPPSHVSGLLINQSQTHYTQNPAAIFYGHHHHTPPPAKRLNP

GPVGITEQLVKAAEVIESDTCLAQGILARLNQQLSSPVGKPLERAAFYFKEALNNLLHNVSQTLNPYSLIFKIAAYKSFSEISPVLQFANFTSNQALLES

FHGFHRLHIIDFDIGYGGQWASLMQELVLRDNAAPLSLKITVFASPANHDQLELGFTQDNLKHFASEINISLDIQVLSLDLLGSISWPNSSEKEAVAVNI

SAASFSHLPLVLRFVKHLSPTIIVCSDRGCERTDLPFSQQLAHSLHSHTALFESLDAVNANLDAMQKIERFLIQPEIEKLVLDRSRPIERPMMTWQAMFL

QMGFSPVTHSNFTESQAECLVQRTPVRGFHVEKKHNSLLLCWQRTELVGVSAWRCRSS

>AT5G52510

MESGFSGGGGGSDFYGGGGGRSIPGGPGTVINVGNNNPQTTYRNQIPGIFFDQIGNRVAGGNGFSGKRTLADFQAAQQHQQQQQQQPFYSQAALNAFLSR

SVKPRNYQNFQSPSPMIDLTSVNDMSLFGGSGSSQRYGLPVPRSQTQQQQSDYGLFGGIRMGIGSGINNYPTLTGVPCIEPVQNRVHESENMLNSLRELE

KQLLDDDDESGGDDDVSVITNSNSDWIQNLVTPNPNPNPVLSFSPSSSSSSSSPSTASTTTSVCSRQTVMEIATAIAEGKTEIATEILARVSQTPNLERN

SEEKLVDFMVAALRSRIASPVTELYGKEHLISTQLLYELSPCFKLGFEAANLAILDAADNNDGGMMIPHVIDFDIGEGGQYVNLLRTLSTRRNGKSQSQN

SPVVKITAVANNVYGCLVDDGGEERLKAVGDLLSQLGDRLGISVSFNVVTSLRLGDLNRESLGCDPDETLAVNLAFKLYRVPDESVCTENPRDELLRRVK

GLKPRVVTLVEQEMNSNTAPFLGRVSESCACYGALLESVESTVPSTNSDRAKVEEGIGRKLVNAVACEGIDRIERCEVFGKWRMRMSMAGFELMPLSEKI

AESMKSRGNRVHPGFTVKEDNGGVCFGWMGRALTVASAWR

>AT2G45160

MPLSFERFQGEGVFGLSSSSFYSDSQKIWSNQDKTEAKQEDLGYVVGGFLPEPTSVLDALRSPSPLASYSSTTTTLSSSHGGGGTTVTNTTVTAGDDNNN

NKCSQMGLDDLDGVLSASSPGQEQSILRLIMDPGSAFGVFDPGFGFGSGSGPVSAPVSDNSNLLCNFPFQEITNPAEALINPSNHCLFYNPPLSPPAKRF

NSGSLHQPVFPLSDPDPGHDPVRRQHQFQFPFYHNNQQQQFPSSSSSTAVAMVPVPSPGMAGDDQSVIIEQLFNAAELIGTTGNNNGDHTVLAQGILARL

NHHLNTSSNHKSPFQRAASHIAEALLSLIHNESSPPLITPENLILRIAAYRSFSETSPFLQFVNFTANQSILESCNESGFDRIHIIDFDVGYGGQWSSLM

QELASGVGGRRRNRASSLKLTVFAPPPSTVSDEFELRFTEENLKTFAGEVKIPFEIELLSVELLLNPAYWPLSLRSSEKEAIAVNLPVNSVASGYLPLIL

RFLKQLSPNIVVCSDRGCDRNDAPFPNAVIHSLQYHTSLLESLDANQNQDDSSIERFWVQPSIEKLLMKRHRWIERSPPWRILFTQCGFSPASLSQMAEA

QAECLLQRNPVRGFHVEKRQSSLVMCWQRKELVTVSAWKC

>AT4G36710

MKIPASSPQDTTNNNNNTNSTDSNHLSMDEHVMRSMDWDSIMKELELDDDSAPNSLKTGFTTTTTDSTILPLYAVDSNLPGFPDQIQPSDFESSSDVYPG

QNQTTGYGFNSLDSVDNGGFDFIEDLIRVVDCVESDELQLAQVVLSRLNQRLRSPAGRPLQRAAFYFKEALGSFLTGSNRNPIRLSSWSEIVQRIRAIKE

YSGISPIPLFSHFTANQAILDSLSSQSSSPFVHVVDFEIGFGGQYASLMREITEKSVSGGFLRVTAVVAEECAVETRLVKENLTQFAAEMKIRFQIEFVL

MKTFEMLSFKAIRFVEGERTVVLISPAIFRRLSGITDFVNNLRRVSPKVVVFVDSEGWTEIAGSGSFRREFVSALEFYTMVLESLDAAAPPGDLVKKIVE

AFVLRPKISAAVETAADRRHTGEMTWREAFCAAGMRPIQLSQFADFQAECLLEKAQVRGFHVAKRQGELVLCWHGRALVATSAWRF

>AT3G13840

MLLEETEPPNQTLDHVLSWLEDSVSLSPLPGFDDSYLLHEFDGSQTWEWDQTQDPEHGFIQSYSQDLSAAYVGCEATNLEVVTEAPSIDLDLPPEIQQPN

DQSRKRSHDGFLEAQQVKKSARSKRKAIKSSEKSSKDGNKEGRWAEKLLNPCALAITASNSSRVQHYLCVLSELASSSGDANRRLAAFGLRALQHHLSSS

SVSSSFWPVFTFASAEVKMFQKTLLKFYEVSPWFALPNNMANSAILQILAQDPKDKKDLHIIDIGVSHGMQWPTLLEALSCRLEGPPPRVRITVISDLTA

DIPFSVGPPGYNYGSQLLGFARSLKINLQISVLDKLQLIDTSPHENLIVCAQFRLHHLKHSINDERGETLKAVRSLRPKGVVLCENNGECSSSADFAAGF

SKKLEYVWKFLDSTSSGFKEENSEERKLMEGEATKVLMNAGDMNEGKEKWYERMREAGFFVEAFEEDAVDGAKSLLRKYDNNWEIRMEDGDTFAGLMWKG

EAVSFCSLWK

>LOC_Os06g03710

MLAGCSFSSSRHQMSTAQRFDILPCGFSKRGSRGDGAAPRVAGDARSGATTCSFRTHPAPPVTQSVSWGAKPEPGGNGNGAHRAVKRAHDEDAVEEYGPI

VRAKRTRMGGDGDEVWFHQSIAGTMQATAAGEGEEAEEEKVFLVPSAAAFPHGMAAAGPSLAAAKKEEYSKSPSDSSSSSGTDGGSSAMMPPPQPPEFDA

RNGVPAPGQAEREALELVRALTACADSLSAGNHEAANYYLARLGEMASPAGPTPMHRVAAYFTEALALRVVRMWPHMFDIGPPRELTDDAFGGGDDDAMA

LRILNAITPIPRFLHFTLNERLLREFEGHERVHVIDFDIKQGLQWPGLLQSLAARAVPPAHVRITGVGESRQELQETGARLARVAAALGLAFEFHAVVDR

LEDVRLWMLHVKRGECVAVNCVLAMHRLLRDDAALTDFLGLARSTGATILLLGEHEGGGLNSGRWEARFARALRYYAAAFDAVDAAGLPEASPARAKAEE

MFAREIRNAVAFEGPERFERHESFAGWRRRMEDGGGFKNAGIGEREAMQGRMIARMFGPDKYTVQAHGGGGSGGGEALTLRWLDQPLYTVTAWTPAGDGA

GGSTVSASTTASHSQQS

>LOC_Os11g31100

MAGGGAKLQQQQAPTSPTASVSESNIVASTASADPEANDALAGLQALRFDGGGDIDDVEIQSPDIALWESIFADQIGVSGAGADFLLSMSSAAASPRRDF

MACSPKRDYMVTTSSPKRDYMVTSSPKRDYMVSSPKREYMVTSPRREMATSPRRATFSNLYTSSHGGGGGGGHHLHHQSYVHGGGMEGGGGGHGAQPQYG

GLAGHGKGKAQSPLHKVYINNVGGGSGGGGVKSNTPSTLSCASSYVVHGGESGLPSLPSMDPFLEEGYLGSYQLPEKAAGGVGGGGGGDINRSGASVSVV

TAPASSQLLPTLSECLAMPEPAAYRGGGDEAVAAAMAVAGELPVGAFVQPEMYYGGGGEFGGEGMTPPLQHQMAADSSLHSMLGSVIQSEAEQEQDSGLQ

LVHLLLACADLVSKGDHPAALRHLHLLRRVASPLGDSMQRVASHFADALAARLSLLSSPTSASPSPRAAAAAAPYPFPPSPETLKVYQILYQACPYIKFA

HFTANQAIFEAFHGEDRVHVVDLDILQGYQWPAFLQALAARPGGPPTLRLTGVGHPPAAVRETGRHLASLAASLRVPFEFHAAAADRLERLRPAALHRRV

GEALAVNAVNRLHRVPSSHLPPLLSMIRDQAPKIITLVEQEAAHNGPYFLGRFLEALHYYSAIFDSLDATFPAESTARMKVEQCLLAPEIRNVVACEGAE

RVARHERLERWRRLMEGRGFEAVPLSAAAVGQSQVLLGLYGAGDGYRLTEDSGCLLLGWQDRAIIAASAWRC

>LOC_Os11g03110

MGSSSLLLFPSSSSSATHSSYSPSSSSHAITSLLPPLPSDHHLLLYLDHQEQHHLAAAMVRKRPASDMDLPPPRRHVTGDLSDVTAAAAPSSASAQLPAL

PTQLPAFHHTDMDLAAPAPPPPQQQVAAGEGGPPSTAWVDGIIRDIIASSGAAVSVAQLIHNVREIIRPCNPDLASILELRLRSLLTSDPAPPPPPPPSH

PALLPPDATAPPPPPTSVAALPPPPPPQPDKRRREPQCQEQEPNQPQSPKPPTAEETAAAAAAAKERKEEQRRKQRDEEGLHLLTLLLQCAESVNADNLD

EAHRALLEIAELATPFGTSTQRVAAYFAEAMSARLVSSCLGLYAPLPNPSPAAARLHGRVAAAFQVFNGISPFVKFSHFTANQAIQEAFEREERVHIIDL

DIMQGLQWPGLFHILASRPGGPPRVRLTGLGASMEALEATGKRLSDFADTLGLPFEFCPVADKAGNLDPEKLGVTRREAVAVHWLRHSLYDVTGSDSNTL

WLIQRLAPKVVTMVEQDLSHSGSFLARFVEAIHYYSALFDSLDASYSEDSPERHVVEQQLLSREIRNVLAVGGPARTGDVKFGSWREKLAQSGFRVSSLA

GSAAAQAVLLLGMFPSDGYTLIEENGALKLGWKDLCLLTASAWRPIQASGR

>LOC_Os12g02870

MGSSSLLLFPSSSSSATHSSYSPSSSSHAITSLLPPLPSDHHLLLYLDHQEQHHLAAAMVRKRPASDMDLPPPRRHVTGDLSDVTAAAAGAPTLSASAQL

PALPTQLPAFHHTDMDLAAPAPPAPQQVAAGEGGPPSTAWVDGIIRDIIASSGAAVSVAQLIHNVREIIRPCNPDLASILELRLRSLLNSDPAPPPPPPS

HPALLPPDATAPPPPPTSVAALPPPPPAQPDKRRREPQCQEQEPNQPQSPKPPTAEETAAAAAAAAAAAAAAAKERKEEQRRKQRDEEGLHLLTLLLQCA

ESVNADNLDEAHRALLEIAELATPFGTSTQRVAAYFAEAMSARLVSSCLGLYAPLPSPSPAGARVHGRVAAAFQVFNGISPFVKFSHFTANQAIQEAFER

EERVHIIDLDIMQGLQWPGLFHILASRPGGPPRVRLTGLGASMEALEATGKRLSDFADTLGLPFEFCPVADKAGNLDPEKLGVTRREAVAVHWLRHSLYD

VTGSDSNTLWLIQRLAPKVVTMVEQDLSHSGSFLARFVEAIHYYSALFDSLDASYSEDSPERHVVEQQLLSREIRNVLAVGGPARTGDVKFGSWREKLAQ

SGFRVSSLAGSAAAQAALLLGMFPSDGYTLIEENGALKLGWKDLCLLTASAWRPIQASGR

>LOC_Os03g49990

MKREYQEAGGSSGGGSSADMGSCKDKVMAGAAGEEEDVDELLAALGYKVRSSDMADVAQKLEQLEMAMGMGGVSAPGAADDGFVSHLATDTVHYNPSDLS

SWVESMLSELNAPLPPIPPAPPAARHASTSSTVTGGGGSGFFELPAAADSSSSTYALRPISLPVVATADPSAADSARDTKRMRTGGGSTSSSSSSSSSLG

GGASRGSVVEAAPPATQGAAAANAPAVPVVVVDTQEAGIRLVHALLACAEAVQQENFAAAEALVKQIPTLAASQGGAMRKVAAYFGEALARRVYRFRPAD

STLLDAAFADLLHAHFYESCPYLKFAHFTANQAILEAFAGCHRVHVVDFGIKQGMQWPALLQALALRPGGPPSFRLTGVGPPQPDETDALQQVGWKLAQF

AHTIRVDFQYRGLVAATLADLEPFMLQPEGEADANEEPEVIAVNSVFELHRLLAQPGALEKVLGTVHAVRPRIVTVVEQEANHNSGSFLDRFTESLHYYS

TMFDSLEGGSSGQAELSPPAAGGGGGTDQVMSEVYLGRQICNVVACEGAERTERHETLGQWRNRLGRAGFEPVHLGSNAYKQASTLLALFAGGDGYRVEE

KEGCLTLGWHTRPLIATSAWRVAAA

>LOC_Os01g45860

MAMDTFPFQWPMDPAASSGLDAGFLPPPAAVAPDDGVGYYDPPAGADVDAAALPEFAAAFPPCAPDAAAAVLAMRREEEEVAGIRLVHLLMSCAGAIEAG

DHALASAQLADSHAALAAVSAASGIGRVAVHFTTALSRRLFPSPVAPPTTDAEHAFLYHHFYEACPYLKFAHFTANQAILEAFHGCDHVHVIDFSLMQGL

QWPALIQALALRPGGPPFLRITGIGPPSPTGRDELRDVGLRLADLARSVRVRFSFRGVAANSLDEVRPWMLQIAPGEAVAFNSVLQLHRLLGDPADQAPI

DAVLDCVASVRPKIFTVIEQEADHNKTGFLDRFTEALFYYSAVFDSLDAASASGGAGNAMAEAYLQREICDIVCGEGAARRERHEPLSRWRDRLTRAGLS

AVPLGSNALRQARMLVGLFSGEGHSVEEADGCLTLGWHGRPLFSASAWEAAGDGGGDNNNNSNSNVSGSSGSDSNNSGSSNGKSSGARDGSSVCL

>LOC_Os05g49930

MAQFGGFGGWSAMDVAAAAAAALGNVSGAVYHADPAAAVYASLVPGMAVVPGRAPPSAVQIEAARRWKELEKMALRSVNLMVTCAGAIQAGDYAAAAGSL

SDAREIFAKMPTTRTGIGRVLTHFADALAERLFPAFPQSAPPPPPPRGEQRELFRGFYEAGPYLKFAHLAANQAILEAFEGCNSVHVIDFALTDGIQWPS

LIQALAVRPGGPPFLRITGIGPHAAGNRDELRDVGLRLAEFARSCSVPFAFRGIAADQLDGLRPWMFQVAPGEAVAINSVLQLHRLLVDQDAAAAASFPA

PIDGVLDWVASMNPRVFTVVEQEADHNKSSLLERFTNSLFYYASMFDSLEAISRHGGGDGAGNPLAEAYLQGEIADIVSREGSSRVERHEQMPRWVERLR

RGGMTQLPLGATGLWQAAMQLREFSGAGFGVQENGGFLTLTWHSQRLYSASAWRATAGKKMTMMASGAADAMEESQNSNTNGGGGGSSGGGHGALNQIMQ

>LOC_Os07g39470

MADTPTSRMIHPFSNIPSQNLKQFQYSDNPQHPCHPYRAPSDTHVVPHHYGLKSHSPDAGYESQATPNKYTLDSSEGAGCMRHDSPSSQSFTTRSGSPLS

QEDSHSDSTDGSPVGASCVTEDPNDLKQKLKDLEAVMLGPDSEIVNSLENSVANQLSLEPEKWVRMMGIPRGNLKELLIACARAVEEKNSFAIDMMIPEL

RKIVSVSGEPLERLGAYMVEGLVARLASSGISIYKALKCKEPKSSDLLSYMHFLYEACPYFKFGYMSANGAIAEAVKGEDRIHIIDFHISQGAQWISLLQ

ALAARPGGPPTVRITGIDDSVSAYARGGGLELVGRRLSHIASLCKVPFEFHPLAISGSKVEAAHLGVIPGEALAVNFTLELHHIPDESVSTANHRDRLLR

MVKSLSPKVLTLVEMESNTNTAPFPQRFAETLDYYTAIFESIDLTLPRDDRERINMEQHCLAREIVNLIACEGEERAERYEPFGKWKARLTMAGFRPSPL

SSLVNATIRTLLQSYSDNYKLAERDGALYLGWKSRPLVVSSAWH

>LOC_Os07g38030

MLQGVLSRAPGADAAAMKAKRAADDEEEGGERERARGKRLAAEGKQGLVVVSTGEEEEAAAETRGLRLLSLLLRCAEAVAMDQLPEARDLLPEIAELASP

FGSSPERVAAYFGDALCARVLSSYLGAYSPLALRPLAAAQSRRISGAFQAYNALSPLVKFSHFTANQAIFQALDGEDRVHVIDLDIMQGLQWPGLFHILA

SRPTKPRSLRITGLGASLDVLEATGRRLADFAASLGLPFEFRPIEGKIGHVADAAALLGPRHHGEATVVHWMHHCLYDVTGSDAGTVRLLKSLRPKLITI

VEQDLGHSGDFLGRFVEALHYYSALFDALGDGAGAAEEEAAERHAVERQLLGAEIRNIVAVGGPKRTGEVRVERWGDELRRAGFRPVTLAGSPAAQARLL

LGMYPWKGYTLVEEDGCLKLGWKDLSLLTASSWEPTDGDADADVAVAGDTHHESHDS

>LOC_Os10g22430

MSARASNHAYICSDDSQMPYYNNSVPSGGNGRFYITQNHQDAHYASSDDGSQKIGSSPQAFEAPYCTLESSSANGAHPAHSSASSHSISPISGSPLSHHD

SHSDHTYNSPPSASCVTEITDLQIKLRELENAILGPELDIAYDSPESALQPNIMATPENWRQLLGINTGDLKQVIIACGKAVAENDVRLTELLISELGQM

VSVSGDPLQRLGAYMLEGLVARLSSSGSKIYKSLKCKEPTSSELMSYMHLLYEICPFFKFGYMSANGAIAEAIKGENFVHIIDFQIAQGSQWMTLIQALA

ARPGGPPFLRITGIDDSNSAYARGGGLDIVGMRLYKVAQSFGLPFEFNAVPAASHEVYLEHLDIRVGEVIVVNFAYQLHHTPDESVSTENHRDRILRMVK

SLSPRLVTLVEQESNTNTRPFFPRYLETLDYYTAMFESIDVALPRDDKRRMSAEQHCVARDIVNLIACEGAERVERHEVFGKWKARLTMAGFRPYPLSSV

VNSTIKTLLHTYNSFYRLEERDGVLYLGWKNRVLVVSSAWC

>LOC_Os03g09280

MSMQASNRPYRYPDNSQIPYYSRSSMHVGQNGTYHVQQNHEDLYASSDDGSQNGNSKAQGLQAQYCTLDSSSGNFVYPAHSSTSSHISGSPISQQDSHSE

HTSGSPASASCVTEVPGLRFTTIEEIENAMFGPEPDTVSSDCSLLTDSAFYQDNWREHLGINTGDLKQVIAACGKAVDENSWYRDLLISELRNMVSISGE

PMQRLGAYMLEGLVARLSSTGHALYKSLKCKEPTSFELMSYMHLLYEICPFFKFGYMSANGAIAEAVKGENFVHIIDFQIAQGSQWATMIQALAARPGGP

PYLRITGIDDSNSAHARGGGLDIVGRRLFNIAQSCGLPFEFNAVPAASHEVMLEHLDIRSGEVIVVNFAYQLHHTPDESVGIENHRDRILRMVKGLSPRV

VTLVEQEANTNTAPFFNRYLETLDYYTAMFEAIDVACPRDDKKRISTEQHCVARDIVNLIACEGAERVERHEPFGKWRARLSMAGFRPYPLSALVNNTIK

KLLDSYHSYYKLEERDGALYLGWKNRKLVVSSAWR

>LOC_Os03g51330

MAYMCADSGNLMAIAQQVIQQQQQQQQQQQRHHHHHHLPPPPPPQSMAPHHHQQKHHHHHQQMPAMPQAPPSSHGQIPGQLAYGGGAAWPAGEHFFADAF

GASAGDAVFSDLAAAADFDSDGWMESLIGDAPFQDSDLERLIFTTPPPPVPSPPPTHAAATATATAATAAPRPEAAPALLPQPAAATPVACSSPSPSSAD

ASCSAPILQSLLSCSRAAATDPGLAAAELASVRAAATDAGDPSERLAFYFADALSRRLACGTGAPPSAEPDARFASDELTLCYKTLNDACPYSKFAHLTA

NQAILEATGAATKIHIVDFGIVQGIQWAALLQALATRPEGKPTRIRITGVPSPLLGPQPAASLAATNTRLRDFAKLLGVDFEFVPLLRPVHELNKSDFLV

EPDEAVAVNFMLQLYHLLGDSDELVRRVLRLAKSLSPAVVTLGEYEVSLNRAGFVDRFANALSYYRSLFESLDVAMTRDSPERVRVERWMFGERIQRAVG

PEEGADRTERMAGSSEWQTLMEWCGFEPVPLSNYARSQADLLLWNYDSKYKYSLVELPPAFLSLAWEKRPLLTVSAWR

>LOC_Os01g67650

MGMSPEPCNSISDCSQQQSHHLTLTQQQDSTIICTNQELDYYYRFYDVDEAAFDGNEVELVSRFSKVTRMDHMISSPYQPTWSPAQAAVDVVGSSETSRV

RKKRFWDVLESCKQKVEAMEAMDTPATATFRVGAGDGGGGGGGGAGGGGGGADGMRLVQLLVACAEAVACRDRAQAAALLRELQAGAPVHGTAFQRVASC

FVQGLADRLPLAHPPALGPASMAFCIPPSSCAGRDGARGEALALAYELCPYLRFAHFVANACMLEAFEGESNVHVVDLGMTLGLDRGHQWRGLLDGLAAR

ASGKPARVRVTGVGARMDTMRAIGRELEAYAEGLGMYLEFRGINRGLESLHIDDLGVDADEAVAINSVLELHSVVKESRGALNSVLQTIRKLSPRAFVLV

EQDAGHNGPFFLGRFMEALHYYAALFDALDAALPRYDARRARVEQFHFGAEIRNVVGCEGAARVERHERADQWRRRMSRAGFQSVPIKMAAKAREWLDEN

AGGGGYTVAEEKGCLVLGWKGKPVIAASCWKC

>LOC_Os01g65900

MSFIRRADPSTTYADNLYIHKFGTPNSNFAARRYASDTQLFRYGPEPYNPENSFYNQQASPMPYMVTADGHSPSSADNSCSDVAKDSPLVSNVSQQNSQS

ISDNQSSELEVEFDEDDIRMKLQELEHALLDDSDDILYEISQAGSINDEWADPMKNVILPNSPKESESSISCAGSNNGEPRTPKQLLFDCAMALSDYNVD

EAQAIITDLRQMVSIQGDPSQRIAAYLVEGLAARIVASGKGIYKALSCKEPPTLYQLSAMQILFEICPCFRFGFMAANYAILEACKGEDRVHIIDFDINQ

GSQYITLIQFLKNNANKPRHLRITGVDDPETVQRTVGGLKVIGQRLEKLAEDCGISFEFRAVGANIGDVTPAMLDCCPGEALVVNFAFQLHHLPDESVSI

MNERDQLLRMVKGLQPKLVTLVEQDANTNTAPFQTRFREVYDYYAALFDSLDATLPRESPDRMNVERQCLAREIVNILACEGPDRVERYEVAGKWRARMT

MAGFTPCPFSSNVISGIRSLLKSYCDRYKFEEDHGGLHFGWGEKTLIVSSAWQ

>LOC_Os07g36170

MDLHQLLKYRLTGANVVYEIPTENNLQNSPWQANPLKYEFSDSPYTPLSSQFECDNLSALTNTPDNQSSTETISAQPISPLEADSSYRQAGILLQENIQV

GADPLYATSRHNMQHALREIETVLMAPDTDDATTSTKHEFEEIKPAQLVRQRSRTWSHESRQPLPGVGRSQFASGGYPTASYEFRPEKRQRELREDPQII

VKQLLTRCAEALSEDRTEEFHKLVQEARGVVSINGEPIQRLGAYLLEGLVARHGNSGTNIYRALKCREPESKELLSYMRILYNICPYFKFGYMAANGAIA

EALRTENNIHIIDFQIAQGTQWITLIQALAARPGGPPRVRITGIDDPVSEYARGEGLDIVGKMLKSMSEEFKIPLEFTPLSVYATQVTKEMLEIRPGEAL

SVNFTLQLHHTPDESVDVNNPRDGLLRMVKGLSPKVTTLVEQESHTNTTPFLMRFGETMEYYSAMFESIDANLPRDNKERISVEQHCLAKDIVNIIACEG

KDRVERHELLGKWKSRLTMAGFRPYPLSSYVNSVIRKLLACYSDKYTLDEKDGAMLLGWRSRKLISASAWH

>LOC_Os01g71970

MVQDEGSSSSVTSSPLHNFSNMPLHPAAAASPTPPWMVRELRSDERGLCLIHLLLNCAAAAAAGRLDAANAALEHIASLAAPDGDAMQRVAAAFAEALAR

RALRAWPGLCRALLLPRASPTPAEVAAARRHFLDLCPFLRLAGAAANQSILEAMESEKIVHVIDLGGADATQWLELLHLLAARPEGPPHLRLTSVHEHKE

LLTQTAMALTKEAERLDVPFQFNPVVSRLDALDVESLRVKTGEALAICSSLQLHCLLASDDDAAAVAGGDKERRSPESGLSPSTSRADAFLGALWGLSPK

VMVVAEQEASHNAAGLTERFVEALNYYAALFDCLEVGAARGSVERARVERWLLGEEIKNIVACDGGERRERHERLERWARRLEGAGFGRVPLSYYALLQA

RRVAQGLGCDGFKVREEKGNFFLCWQDRALFSVSAWRGRRFD

>LOC_Os05g40710

MQDSLGLMQFLDHHQYLYSSSSSNLPLQQPLLSHHHRFLEANEGCAGEDDSPEFVEPPAAAAAAGTFEQRPELGACKEVYSEEGGAAEERTGVAMAGADV

EQVAVEDEEEAHGVRMIALLMECAAAMSVGNLAGANGALLELSQMASPYAASCGERLVAYFARAMAARLVGSWVGVVAPMAPPPSCGAINAAFRALYNVA

PFARLAYLACNQAILEAFHGKRLVHIVDLDVVPGGALQWLSLLPALAARPGGPPVIRVTGFGMSASVLHDTGNQLAGLARKLCMFFEFYAVAKRPGDADA

VADMPGRRPGEAVAVHWLRHAMYDAAGDDGASMRLVRWLEPAAVTLVEQERAHGGGGGHGRFLDRFVSALHHYSAVFDAMGASRPDGEDASRHLAEHGVL

GREIANVLAVGGPARSSGREGPGSWREVLARHGFAHAGGGGGGRAQLVAAACPGGLGYTVAGDHDGTVRLGWKGTPLYAVSAWTWCSPPHARA

>LOC_Os01g62460

MLMAEDIDEKFEHYPVNADDLLAAEKPFLEILADQSPYSGGSSVESPDGSSAANSCNSLSPCNCSSSSDGLGAVPQTPVLEFPTAAFSQTPQLYGDLIPT

GGMVESGGAWPYDPTEFYQLQTKPVRENLPSQSSSFASSNGSSVTFSEGFESLLSPAGVLPDVSLNDFVVQNQQALQFRRGFQEASKFLPDESKLVIDVD

KLYSGDEGSRFLGEVRQEKKLVKVKTETSDVESAGHRGKKHFYGDDLDAEEGRCSKHSAQGIDTDHLVRDLMDKVLLCNGETCSKGVKELREALQHDVAK

HSGGGHGKGSSHGKGRGKKQPKKEVVDLETLLIHCAQSVATDDRRSATELLKQIRQHAHANGDGDQRLAHCFANGLEARLAGTGSQIYKNYTITRLPCTD

VLKAYQLYLAACPFKKISHYFANQTILNAVEKAKKVHIVDYGIYYGFQWPCLIQRLSNRPGGPPKLRITGIDTPQPGFRPAERTEETGRYLSDYAQTFNV

PFEFQAIASRFEAVRMEDLHIEEDEVLIVNCMFKFKNLMDESVVAESPRNMALKTIRKMNPHVFIHGVVNGSYNAPFFVTRFREALFHYSAIFDMLETNI

PKDNEQRLLIESALFSREAINVISCEGLERMERPETYKQWQVRNQRVGFKQLPLNQDMMKRAREKVRCYHKDFIIDEDNRWLLQGWKGRILFALSTWKPD

NRSSS

>LOC_Os04g50060

MLDSGSYDDVDYGDLFSIPNPPAPHLLNFPLQFFPSNGFISSADDSHRSPAGMFGSTPSPTSTTTELENSEDLSESADDAVLAYINQFLLEDEEDESCPG

TITSVEDSALLAVEKPFVDILTASQEACQENSWIDSCCDFTGNGGLLDTFTTTHAACQPAPCEFEKEKGECAVHKGRKNPHDDCLLFEEESRRSKQLAVS

EEETVREMFDKVLLCNGECELRAPLPAEARNCGVYVKGSGNKRGRKKGKSGASAEDDAVDLTTLLIHCAQAAAIDDHRNSNELLKQIRQRSSAYGDAGQR

LAHCFANALEARLAGTGSNIYRSLAAKRTSVYDILNAFKLYVTACPFKKISNFFSIEAILNASKGMTRLHIVDYGIQYGFQWPIFFQRISKRPGGPPSVR

ITGVDLPQPGFRPAQLIEATGRRLHDYARMFNVPFEYHAIAAKWDTIRVEDLKIDKDKDELLVVNCLFRMRNMMDEMVTDDSPRMQVLKTIRKMNPNLFI

HGVVNGTYNAPFFVTRFKEALFYYSSLFDMLETTASRVDENRLLIERDLFGREALNVVACEGTERVERPETYKQWQVRNIRAGFKQLPLNQETVKKARYK

VKKSYHRDFLVDEDNKWMLQGWKGRIIFALSAWEPN

>LOC_Os03g48450

MGLDNNFGELSGMFCGLSYDGYTDHGSQSDYFRFADPQPAIVPQMDAGPSSAASSTASRAAVSSGTDNPEDWEFISDESLNYISRMLMEEDIDEKVSMYQ

EESAALRAAAKPFYDILGHKFPPSPDRQLVAWPLDSPSESSTSSYPHSLASSVTSSNISGAVDSSQRRYVGHSEYRSLSGHSSQPPVGPSSDVRNAMETL

EDPLISNGRIPEYLFESFPTWDFRRGVDEAQKFLPGSDKVVIDLEAGGVAKRQEAGKAISLNVSKAEVLKVKKNRQSEDLDVMEGRNSKQSAFCSDEPDW

IEMFDVLLRQTEKKATDLRKMMRFEASKNSQVAQPKGPSGTRSRGRKPTKKDVVDLRTLLIHCAQAVAADDRRTANELLKQIRQHAKPNGDGSQRLAYCF

ADGLEARLAGTGSQLYHKLVAKRTTASDMLKAYHLYLAACPFKRLSHFLSNQTILSLTKNASKVHIIDFGIYFGFQWPCLIRRLFKREGGPPKLRITGID

VPQPGFRPTERIEETGQRLAEYAEKIGVPFEYQGIASKWETICVEDLNIKKDEVVIVNCLYRFRNLIDETVAIDSPRNRVLNTIRQVNPAIFIHGIVNGS

YSVPFFITRFREALFHFSALFDMLETTVPRDDAQRALIERDLFGREALNVIACEGSDRVERPETYKQWQVRNLRAGFVQSPLNQDIVLKAKDKVKDIYHK

DFVIDEDSEWLLQGWKGRIIYAISTWKPNNN

>LOC_Os02g10360

MLGSSPARDRGGGDDAEASEQPQPQPQPPLSPRAGGGEARGLVLACADLVHRGDLDGARRVAEAVLAAADPRGEAGDRLAHHFARALLALRGGGKGGHGG

GGGGVVPSSAAYLAYIKIAPFLRFAHLTANQAILEAAAADAGGAHRRVLHIVDLDAAHGVQWPPLLQAIADRADPAVGPPPEVRLTGAGTDRDVLLRTGD

RLRAFSSSLNLPFRFHPLILPCTAELAADPTAALELHPDETLAVNCVLFLHKLGGDGELAAFLRWVKSMNPAVVTIAEREGVLGGDVDDDNVPDELPRRV

AAAMDYYSSVFDALEATVPPASADRLAVEQEILSREIDAAVAAPGAGGGGRARDFDAWASAARAAGLAPRPLSAFAASQARLLLRLHYPSEGYKADDDGG

RGACFLRWQQRPLMSVSSWQPQP

>LOC_Os06g40780

MHESDELLLLVAFLTLGISLSPSCPLLALCEGWKQGISACCGCMTQMHMSLLVGNDGVQVEVMIDCLIWGLRHSIQGCPMPCPHIHSQLVELSPSPPLFA

IFHLFANVLYQDALDYMQCETLTQLDQVWGVCLFLLQGSYLEAIINEDPTKGQNMRWLETWEAAIASPDVGRARSPSLLNPPSSDAPSPPPPSPPPPPPS

PAAHRRCSSSGGGGGDDDGYGGGDGMLRSLHSSSSSDTDNNSGGCKNNGGGGGEAAAAVEGGGDQRAVAAAAPSTRDLLLACADLLQRGDLPAARRAAEI

VLAAAASPRGDAADRLAYHFARALALRVDAKAGHGHVVVGGGAARPASSGAYLAFNQIAPFLRFAHLTANQAILEAVDGARRVHILDLDAVHGVQWPPLL

QAIAERADPALGPPEVRVTGAGADRDTLLRTGNRLRAFARSIHLPFHFTPLLLSCATTAPHHVAGTSTGAAAAASTAAAATGLEFHPDETLAVNCVMFLH

NLAGHDELAAFLKWVKAMSPAVVTIAEREAGGGGGGGDHIDDLPRRVGVAMDHYSAVFEALEATVPPGSRERLAVEQEVLGREIEAAVGPSGGRWWRGIE

RWGGAARAAGFAARPLSAFAVSQARLLLRLHYPSEGYLVQEARGACFLGWQTRPLLSVSAWQPSSS

>LOC_Os04g35250

MNLKLSLAIDGGGGGDAAAAVAKKSKVVGGGAVVVDGVGSSAICGGDRGSRVRDRMVKKAEEFDHENGMAATSSDGGGGGGGGMELVRLLLSAVAAGEAG

DARAAAAALREVDRRASCRGGGDPAQRVAACYAAALAPRLAAGLRPARSSPAAPAAARAEQFLAYTMFYQASPFYQFAHFTANQAIVEAFESGGRRRLHV

VDFDVSYGFQWPSLIQSLSDAAAAATSSSSHDDDDNGGGCGDGPVSLRITGFGASADELRETEARLRRFAAGCPNLRFEFEGILNNGSNTRHDCTRIDDD

ATVVVNLVFPASSREACAATRMAYINSLNPSMVFLIEKHDGGGGLTGGDNTTTGRSASLLPRFAANLRYFAAVFDSLHECLPADSAERLAIERDHLGREI

ADAVASLDHQHRRRHGGGGGGGDHAAASWNWKAAMEGAGLDGVKLSSRTVSQAKLLLKMKSGCGGGGFRVVEGDGGMAMSLAWRDMALATATLWRRRRRR

RRCR

>LOC_Os12g38490

MGSSAADSFPACGDDAIRDVYGIGGGGEEDDPSLFLYLSDLAPVSPSAYLDLPPSPPPPTTTATTMVKEGEEAPEDLVLPFISRMLMEEDIDDKFFYDYP

DNPALLQAQQPFLEILSDPSSNSRSSNSDDPRLSPTSSSDTSAAINSYDAAATATAVAAAAVPVPQYESIELDPAAFFAAANSDLMSSAFLKGMEEANKF

LPTENKLIIDLEASSENNYLRGLEEAKRFLPSDDKLQVGFAAAAAPVVSVKKEAVDVVVATASGGGGRGRKNPYDDEELELEGGRSSKQTAVQGDDVAAR

AMFDKVMMPSHENCTEMMEKLRIAMKEEAAKNEASAGGKGGNGKVKGGRRGGRDVVDLRTLLIHCAQAVATDDRRSATELLKQIKQHAKPTGDATQRLAH

CFAEGLQARIAGTGSLVHQSLVAKRTSAVDILQAYQLYMAAICFKKVSFIFSNQTIYNASLGKKKIHIVDYGIQYGFQWPCFLRRISQREGGPPEVRMTG

IDLPQPGFRPTERIEETGHRLSKYAQEFGVPFKYNAIAAVKMESVRKEDLNIDPDEVLIVNCQYQFKNLMDESVVIDSPRDIVLSNIRKMQPHVFIHAIV

NGSFSAPFFVTRFREALFFYSALFDVLDATTPRESEQRLLIEQNIFGRAALNVIACEGIDRVERPETYKQWQVRNQRAGFKQLPLNPEIVQVVRNKVKDC

YHKDFVIDIDHQWLLQGWKGRILYAISTWTPNDALSYF

>LOC_Os11g47900

MATTPEAAESLRFRWPAAEEEFDNDMVLPYISRLLMEDDVHDHFFYQYPDHPALLRAQQPFAQILASSPSSAAGASSSSSSSDAPPSRPFFDDEAATAKT

FPTAAVHSVDHQYSGGLDMVNMAFLKGMEEANKFLPTNTLLLSTDSSTTLQLQVQGEVVVDGHGMLGGVGGAAAAHAHGAINSKKVNCRDDDLEAGTGRA

TKLMAPEPELEEEGARQMFDEMMLQEHEICMKGVKQLSLKSKSSSSKKARGRRTVIHTEPVDLHNLLLHCAQAVATDDRRSAHELLRQIKQHSSAWGDAG

QRLAHCFAQGLEARLAGTGSQVYQSLMSQRTSVVDFLKAYRLYMEACCCKKVAFVFSNKTIYDAVAGRRKLHIVDYGLSYGFQWPGLLRELAARRGGPPE

VRITGIDLPQPGFRPDQHIEETGRRLSRYADELGVPFKFHGIAATKKESVRREELGEAEEDEVVVVISLCHFRNVMDESLQEDSSRSPRDEVLGNIRRMR

PDVFIHGIMNGAYGATYFLTRFREALYYYAAQFDLLDATVGRESHERMLVERDIFGRAALNVIACEGAERVERPEMYKQWQARNQRAGLRQLPLNPQVVR

LVLDKVRDKYHKDFVVDEDQRWLLHRWKGRVLYALSTWVAQH

>LOC_Os03g15680

MEVTMEDVAGDFEFSGCGSTTTTSSASSLDDGTGMCYAWGELSPVADWANFCCSDDDGGHDLHGLIESMLCDDTLVGVDDDGQAGLHHADMFRDDLYCYG

NGSNPSSTTTTNPGSPVFDDPTQGCPEKGLRLLHLLMAAAEALSGPHKSRELARVILVRLKEMVSHTASANAAASNMERLAAHFTDALQGLLDGSHPVGG

SGRQAAAAASHHHAGDVLTAFQMLQDMSPYMKFGHFTANQAILEAVSGDRRVHIVDYDIAEGIQWASLMQAMTSRADGVPAPHLRITAVSRSGGGGARAV

QEAGRRLSAFAASIGQPFSFGQCRLDSDERFRPATVRMVKGEALVANCVLHQAAATTTIRRPTGSVASFLSGMAALGAKLVTVVEEEGEAEKDDDGDSAG

DAAAGGFVRQFMEELHRYSAVWDSLEAGFPTQSRVRGLVERVILAPNIAGAVSRAYRGVDGEGRCGWGQWMRGSGFTAVPLSCFNHSQARLLLGLFNDGY

TVEETGPNKIVLGWKARRLMSASVWAPPPLPVPSSPPEGVCQPVVGMAPVATGGFARTEFDYIDSFLVEPAYALV

>LOC_Os11g47890

MAAAPKPEELVVAIEQPFSPSLFLDLPPTPHHDDDPNNVNDDLLLPFISRILMEDDIDDKFFYQFPDHPALLQAQQSYAQILHAPATSSSSDDTTINNNT

TNSTSVPDTLAMPDHDADTQSAPDDMEMLYMAFLKGREEATKFLPTNNTLFSGFKAEPVLDIQPTFTFGPSGGGRGRKNRHAEEDDLETETSRSSKLMAP

EHDDAAAADEIFDEIILNGYQMIIKGIDELRVAMGSQSQADKNGRRASRAKTAVVDLHTLLIHCAQAVATGDWRSATELLKQIKQNSSARGDATQRMACC

FAEGLEARLAGTGSQMYQSLVAKRTSTVDFLKAYKLFTAACCIKKVSVIFSNKTIYNAVAGRRKLHIVDYGLSYGFQWPALFFLLGAREGGPPEVRMTGI

DVPQPGFRPADQIEETGRRLSICARQFGVPFKFRAIAAKWETVRREDLHLDPEEEEEEVLVVNCLHGLNTLQDESVVVDSPSPRDVVLDNIRDMRPHVFV

QCVVNGAYGAPFFVTRFREALFFYSAHFDMLDATIPRDNDDRLLIERDMLGRCALNVIACEGADRVDRPETYKQWQVRNHRAGLRQLPLEAEVVELVRGK

VKSLYHKDFVIDVDHNWLLQGWKGRILYAMSTWVAHHP

>LOC_Os12g04200

MGPAAPPSEAAAAADDVVLPYISRILMEEDIDDDMFFCLYPDHPALLEAQQPFAQILSSSSGIAGEVNSAPMEDSAALMMQGSGNGRGRKGSKHGGDELE

AEVGRASKLMATPEEEDDDDDGVGEMLEKMMLNGDEEMNAPRVPAEKNGGKAARRKRRQGKGEVVDLRELLMSCAQAVASGNRRSAGELLEQIKRHSSPT

GDATERLAHYFADGLEARLAGAASLEHRLLASAEERASAMELLEAYQVFMAACCFKWVAFTFANMAILRAAEGRSKVHIVDYGGQYHGLQWPSLLQRLAE

REGGPPEVRMTLVGHPQPGFRPARRLERTGRRLSNCARAFGLPFKFRAVAAARWETVTAEDVVGVDHDEAAVVVNDVLSLGTLMDESGVFDDPSPRDTVL

GSIRDMRPAVFVQAVVNGAHGAPFFPTRFREALFFFSALFDMLDATTPEEGSHLRAVLERDVLRRAAVGVIAGEGAERVERPETYRRWQARNRRAGLRQV

AVEADVVEAVRRRVRRRHHEEFVIEEDAGWLLQGWKGRILYAHSAWVVAEDATSVGELTVVTTSLVAIILSAASAQDELPLSTNE

>LOC_Os11g47870

MAAAAPQLEELVDLEPFSPSLFLDLPPTPHSDDPNDDDLILPFISRMLMEDDIDDKFFYQFPDHPALLHAQQPYAQILDAPSDDTTTNSSDDSASATTNN

TTNSAAAANASWPYDPIELSQLLQSPPHPVSDNHDADVGDTRSAPEDDKDLKLLFSAADNMEMLNMAFLKGREEANKLVPTNNTLFAGFDGASLLKTEPA

VDEPTLMFGRSGGSGRGRKNRHGEEDDLEAETGRSSKLMVPPQEATAAASEMFDEIMFNWYEVIMKGMEELRVAMDSEAEKKARNGGGAGRRAARAKAAV

VDLHTLLIHCAQAVATSDRRSATELLKQIKQNSSARGDATQRLACCFAEGLEARLAGTGSQVYKSLVAKCTSTVDFLKAYKLFAAACCIKKVSFIFSNKT

ILDAVAGKRKLHIVDYGLSYGFQWPGLFKCLSEREGGPPEVRITGIDFPQPGFRPADQIEETGRRLSNCARQFGVPFRFQAIAAKWETVRREDLHLDREE

EEEEEEEVLVVNCLHFLNALQDESVVVDSPSPRDMVLNNIRDMRPHVFVQCVVNGAYGAPFFLTRFRETLFFYSSQFDMLDATIPRDNDERLLIERDILG

RWALNVIACEGADRVDRPETYKQWLVRNHRAGLTQLPLQPQVVELVRDKVKKLYHKDFVIDVDHNWLLQGWKGRILYAMSTWVADRDHKSLF

>LOC_Os07g39820

MDTLFRLVSLQAASEQQQQQQQSASYNSRSTTSSGSRSSSHQTNASYSYYHHSSNSGGGGGGGGGYYYGGQQPPPSQYYYLEPYQEECGNAPHHQLYMDE

DFSSSSSSRHFHHGARVQQQQPPASSTPTGTAPTPPLSTSSTAAGAGHGLFEAADLSFPPDLNLDFSSPASSSGGGTASSGAVGGGGGGRWASQLLLECA

RSVAARDSQRVQQLMWMLNELASPYGDVEQKLASYFLQGLFARLTASGPRTLRTLAAASDRNTSFDSTRRTALRFQELSPWSSFGHVAANGAILESFLEV

AAAASSETQRFHILDLSNTFCTQWPTLLEALATRSADETPHLSITTVVSAAPSAPTAAVQRVMREIGQRMEKFARLMGVPFRFRAVHHSGDLAELDLDAL

DLREGGATTALAVNCVNSLRGVVPGRARRRDAFAASLRRLDPRVVTVVEEEADLVASDPDASSATEEGGDTEAAFLKVFGEGLRFFSAYMDSLEESFPKT

SNERLALERGAGRAIVDLVSCPASESMERRETAASWARRMRSAGFSPVAFSEDVADDVRSLLRRYREGWSMREAGTDDSAAGAGVFLAWKEQPLVWASAW

RP

>LOC_Os02g45760

MDPAGAPWCDPRRGYGGYGVGSAALQAAARQQSQQPRSDGAGGAGVTGGVLKRSLGEMERWQQQRQVAAQQAMYLRSVRQRMDIGAVLGGAASSPAYGIS

GLSSGFGGISQQQPSSTMSSLTTASRTVMPGMQQRRRMMAVPTAQNQAVARAPAARPATATELVLLQELEKQLLGDDEEADAAGSGCGSGITSSDWGDTI

QRLNSVTAASSPSLPLPTAVNSTALLARSPTNSSSSTASSSASSSPPISAASSRQLLSEAAAAVADGNHTAAASLLSALKLSANPRGDAEQRLVAMMVAA

LSSRVGTGPSQHLADLYSGEHRAACQLLQDVSPCFGLALHGANLAILDAVAGHRAIHLVDFDVSAAQHVALIKALADRRVPATSLKVTVVADPTSPFTPA

MTQSLAATCERLKKLAQQAGIDFRFRAVSCRAPEIEASKLGCEPGEALAVNLAFTLSRVPDESVSPANPRDELLRRVRALGPRVVTLVEQELNTNTAPMA

ARFSDASAHYGAVLESLDATLGRDSADRTRAEAALASKVANAVGREGPDRVERCEVFGKWRARFGMAGFRAVAIGEDIGGRVRARLGPALPAFDVKLDNG

RLGVGWMGRVVTVASAWR

>LOC_Os03g31880

MDTLFRLVSLHHHHHHQHAASPSPPDQPHKSYPSSRGSTSSPSSHHTHNHTYYHHSHSHYNNNSNTNYYYQGGGGGGGGYYYAEEQQPAAYLEECGNGHQ

FYMDEDFSSSSSSRQFHSGTGAPSSAPVPPPPSATTSSAGGHGLFEAADFSFPQVDISLDFGGSPAVPSSSGAGAGAGAAPSSSGRWAAQLLMECARAVA

GRDSQRVQQLMWMLNELASPYGDVDQKLASYFLQGLFARLTTSGPRTLRTLATASDRNASFDSTRRTALKFQELSPWTPFGHVAANGAILESFLEAAAAG

AAASSSSSSSSSTPPTRLHILDLSNTFCTQWPTLLEALATRSSDDTPHLSITTVVPTAAPSAAAQRVMREIGQRLEKFARLMGVPFSFRAVHHSGDLADL

DLAALDLREGGATAALAVNCVNALRGVARGRDAFVASLRRLEPRVVTVVEEEADLAAPEADASSEADTDAAFVKVFGEGLRFFSAYMDSLEESFPKTSNE

RLSLERAVGRAIVDLVSCPASQSAERRETAASWARRMRSAGFSPAAFSEDVADDVRSLLRRYKEGWSMRDAGGATDDAAGAAAAGAFLAWKEQPVVWASA

WKP

>LOC_Os05g31380

MAVEIPRLALGGGGGGAGGERLPAAGEDSAPAATNAGKRPVVGLGFGSSLAAMAAAAAAGIQPDAFALGGPAEYPAGDGERDVLMVSFLRSIAAFLADGT

CQMQVNDGLSCVVDLAGGDADGGGVGEGRSAQRLASAFAEALALRFILPCDGVCRSLHLTRAPPPPAVSAARQGFRAMCPFVRLAAAAANLSIAEVMEAE

RAVVHVVDLGGGVDANQWVELVRLVAARPGGPPGLLRLTVVNESEDFLSAVAAYVAAEAQRLDLSLQFHPVLSSIEELSATATGSIGSRLVVIPGQPLAV

VANLQIHRLLAFPDYVDGVASRRPAAEQSGSSQHTMTTATKTKADALLRAIRDLNPKLVVLTENEADHNVAELGARVWNALNYYAALFDALEASSTPPAA

VPPHERACVERWVLGEEIKDIVVREGTGRRERHETLGRWAERMVAAGFSPVTAARALASTETLAQQMVAAGGGGAGAGVLRAAHGGGCFPVICWCDVPVF

SVSTWTARRVLVPAPPLWPPAAAGGAGPSGSGYGGDGPSTASSSAAMWWVG

>LOC_Os11g47910

MANPEDFFWEALLKENEAPSPSPVFFELPPTPLANSDGSTDPSSLLDNQLLSYVTSMLMEDEMGSSAAVTNLQCVNRGSTEEANNMLPGSEVVRAFLKGM

EEASKLLPRNNSFRMLETVDQVSSHGHCRGGKKKNHDRDEQQLEEELGRSSKLAAMTNAGTEEAGARELLDELMLHSHETCIKDMEKLRIDMDNEADKTI

KKKGKKGSSSKVVDLRMLLIQCAQAMATDNQQSAGELLKKIKQHALATGDAMQRVAHYFAKGLEARLAGSGKHLYQNHVRMSLVEYLKVYKLYMAACCFK

KVALMFAAMTIMQAVQGKKRLHIVDYGIRCGLHWPDLFRRLGSREDGPPEVRITIVDIPQPGFRPFQRIEAAGHCLSSCANEFRVPFRFQAVVAAKWETV

GAEDLHIEPDEVLVVNDLWSFSALMDESIFCDGPNPRDVALRNISKMQPDVFIQGIINGGYGASFLSRFRGALLYYSALFDMLDATTPRESGLRLALEQN

VLGPYALNAIACEGADLVERPEKYRQWQARNHRAGMQQLKLRPDIVDTIREEVNKYHHKDFLLGEDGQWLLQGWMGRVLFAHSAWVPQQQDNSSG

>LOC_Os05g42130

MDMEHELRGGGVGAAAHGHGSICFSGGPVLVDGRRIQQLLLHCAAALESNDVTLAQQAMWVLNNIASSQGDPSQRLTSWLLRALVARACRLCAAAPAGAA

VEFLERGRAPPWGRAMSVTELADYVDLTPWHRFGFTASNAAILRAVAGASAVHVVDLSVTHCMQWPTLIDVLSKRPGGAPAIRITVPSVRPAVPPLLAVS

SSELGARLAIFAKSKGVQLEFNVVESATTTSPKKTSTTLCQELASVLSDPPSLGLRDGEAVVVNCQSWLRHVAPDTRDLFLDTVRALNPCLLTVTDEDAD

LGSPSLASRMAGCFDFHWILLDALDMSAPKDSPRRLEQEAAVGRKIESVIGEEDGAERSEPGARLAERMSRKGFAGVVFDEEAAAEVRRLLSEHATGWGV

KREDDMLVLTWKGHAAVFTGAWTPN

>LOC_Os12g04380

MEKATRCLARATGLAAAAAAGDGPQKRLAEAMVDCLARRLLRPVQAITDALIDPSVYLDRRSVRAARRGFFELSPFPKVAFVVGNRAIVEAVENESLVHV

VGMSGPFTQPCQWIQLLHELRRRPEGPPRVVRLTVVHDDGELLAKMAEVLSDEAEELDMEFQFHGVVGQLEDLDFSNLRDVLEIKSGEALVVSCTLQLHR

LLAADDDAMYSSRSAHLNQMASIAQLQQMAVSSCPPSTGGGGSVQYKDDDDDPYRSPATPLTFVSPPASTPHLQMPAALANFLSAVRALSPKIVVVAEQD

ADHNGVSFRKRFCEALHHYAAVFDSLDDAAAATTSAASHLWSPDERAQVERVVVGEEIKGVLLRDGAHRRERHDRLRQWAARMEMAGFTGVPLSYAAIRK

GNDMVRRCGLRRCENRECGGCLLLCWSSRPLYSISAWRPAASGGAGSGSERSEYIHVGAEADDR

>LOC_Os11g04590

MEKATRCLARATGLAAAAAAGDGPRKRLAVAMVDCLARRLLRPVQAITDALIDPSVYLDRRSVRAARRGFFELSPFPKVAFVVGNRAIVEAVENESLVHV

VGMSGPFTQPCQWIQLLHELRRRPEGPPRVVRLTVVHDDGELLAKMEELVSDEAEELGMEFQFHGVVGQLEDLDFSNLRNVLEIKSGEALVVSCTLQLHR

LLAADDDAMYSSRSAHLNQMASIAQLQHMAVNSCPSSSGGGSVQYKDDDPYRSPATPLTFVSPPVSTPHFQTPAALASFLSAVRALSPKILVVAEQDADH

NGVSFRKRFCEALHHYAAVFDSLDAAAATTSAASRLWSPDERAQVERVVVGEEIKGVLLRDGAHRREWHDRLRQWAARMEMAGFTGVPLSYAAIKKGNDM

VRRCGLRRCENKECGGCLLLCWSSRPLYSISAWRPAASRGSGSGSERSEYVHVGAEPDDR

>LOC_Os11g47920

MANPEDFFWEALLKENEAPSPPPVFFDLPATPLSNSDGTDPSSLDNQLLSYVSRMLMEDEMGSSAAITNLQCVNRGSTEEANNMLPGSEVVRVFSKGMGE

ASKLLPRNNSFRTLETVDQVSSDGHCRGRKKKNHDRDEQQLEEELGRTSKLAALTIAGTQEAGARELLDELMLHAHETCIKDMEKLRIDMDNEAEKKINK

KDKKGSSSKVVDLRLLLIQCAQATATDNQQSAGELLKKIKQHALATGDAMQRVAHYFAKGLEARLAGRGKHLYQNQMRMSLVEYLKVYKLYMAACCFTKV

ALMFAAMTIMQAVQGKKRLHIVDYGPRCGLHWPDLLRRLGSREDGPPEVRITIVDILQPAFRPFQRIEEAGHCLSSCANEFRVPFRFQAVAAAKWETVGA

EDLHIEPDEVLVVNDLWSFSALMDESVFCDGPNPRDVALRNISKMQPDVFIQGITNDSYGASFLSRFRAVLLYYSALFDILDATTPRDSGLRLALEQNVL

GPYALNAIACEGADLVERPEKYKQWQARNHRAGMQQLKLRPDIVDTIRDEVNKYHHKDFLLGEDGQWLLQGWMGRILFAHSAWVRQSQDTSSG

>LOC_Os06g01620

MSSSSCHPHNPTTLPLPEPDSSKSPEPTSVLYNRSSPSTSLGSCSSKPPEDPPPPIAADDDCDWDAVVDMHMHMLAPAPAPDSSFLRWIMDTGYADADTF

PDHPSFDSDLLQLPMPMPSDHPPQPLVDDLLDAARLLDAGDSTSAREILARLNHRLPSLPSPPGHAHPPLLRAAALLRDALLPPTALPVSSTPLDVPLKL

AAHKALADASPTVQFTTFTSTQAFLDALGSARRLHLLDFDVGFGAHWPPLMQELAHHWRRAAGPPPNLKVTALVSPGSSHPLELHLTNESLTRFAAELGI

PFEFTALVFDPLSSASPPLGLSAAPDEAVAVHLTAGSGAFSPAPAHLRVVKELRPAVVVCVDHGCERGALNLLQSCAALLESLDAAGASPDVVSKVEQFV

LRPRVERLAVGGGDKLPPPLQSMLASAGFAALQVSNAAEAQAECLLRRTASHGFHVEKRQAALALWWQRSELVSVSAWRC

>LOC_Os04g49110

MEPGAPWRDPRQGYLYGVGSAVQMPMQQRSDAAAAGGVLKRSLGDMERWQQHQHQQRQIAMQQQLYLRTVRQRTAAASAAVSPLTSADIAAVLGGPPSQP

LVLSGSSMGGAFGSPSSTLSSITTASRAVAMPLMQPQLQRQQQVTYMASSPQVQAFGTARALPPAPATSDLSILQELEKQLLGDDDEVEAAMSGTGSAVT

GSEWEEQLNSITAAPSPPLTAATTPNNNNNAVGMTRSPSNSSTSTASSSASCSPPTSATTSRQLLSEAAAAIADGHNETAATHLTALKRAANSRGDVEQR

LVAMMVAALSSRIGQTASVPDICGGETRAGSQLLHDISPCFRLALHAANVAIVDAVGDHRAIHLVDFDVSAPQHADLIRCLAARRLPGTSLKVTAVTDPA

SPFTQSVTATLHLQKLAERAGIDYRFKMVSCRAGEIEASKLGCEAGEALAVNLAFALSHVPDESVSPANPRDEILRRVRALGPQVVALVEQELNSNTAPL

TTRFTDACAHYGAILESLDATIPRESAERARAEAALGGRAANAVAREGADRLERCEVFGKWRSRFGMAGFRPVALGPGIADQVLARQGPVAAGFAVKAEN

GVLRLGWMGRVVTVASAWR

>LOC_Os07g40020

MMQFTHTAPPPPPLHPNGHGLGLGLYLDVGATRGGGGARPWSSSSSTTTLGGSGYFPSSAAASKISLGNLNSTGCMEQLLVHCANAIEANDATLTQQILW

VLNNIAPADGDSNQRLTAAFLCALVSRASRTGACKAVTAAVADAVESAALHVHRFTAVELASFIDLTPWHRFGYTAANAAIVEAVEGFPVVHIVDLSTTH

CMQIPTLIDMLAGRAEGPPILRLTVADVAPSAPPPALDMPYEELGAKLVNFARSRNMSMDFRVVPTSPADALTSLVDQLRVQQLVSDGGEALVVNCHMLL

HTVPDETAGSVSLTTAQPPVSLRTMLLKSLRALDPTLVVVVDEDADFTAGDVVGRLRAAFNFLWIPYDAVDTFLPKGSEQRRWYEAEVGWKVENVLAQEG

VERVERQEDRTRWGQRMRAAGFRAAAFGEEAAGEVKAMLNDHAAGWGMKREDDDLVLTWKGHNVVFASAWAPS

>LOC_Os10g40390

MAAAPAPKLPPFPAGGFVPALKPKAEAANDEAAAAVEQLAEAAKLAEAGDAFGAREILARLNYRLPAAPTAGTPLLRSAFYFKEALRLTLSPTGDAPAPS

ASTPYDVVVKLGAYKAFSEVSPVLQFAHLTCVQAVLDELGGAGCIHVLDFDIGMGEQWASLMQELAQLRPAAALKVTALVSPASHHPLELQLIHENLSGF

AAELGVFFHFTVFNIDTLDPAELLANATAGDAVAVHLPVGPAHAAATPAVLRLVKRLGAKVVVSVDRGCDRSDLPFAAHLFHSFHSAVYLLESIDAVGTD

PDTASKIERYLIHPAIEQCVVASHRAASAMDKAPPPPWRAAFAAAGFAPVQATTFAESQAESLLSKLGSASASACADAECHVAMVLYCPLIKERERKRWN

VHLVIVLVELNTPQAEKNQREVGGYGGEKNKILSEYNLLPLPVLEMQRKGGNHGVKRPCFTEMAAKHLRVRTGTSRTAAATLDGYTTLTWDSAPEWPWHR

MHKPDRLRWAHWRVTGEPQCAAGAVAIGQASGHTGVRDTAGANWQETCIVVTEMDVKLADKFRKKWYHSSKLQKRRCTVGLIKQDLKVQNQGGESEASRH

KGLQISFRQVQISLRKPNRKCKHSGQTVKLINENCYYLSTKRNNLKIKEVNYICYIYAEELHPLKVMFRNTQAKICQIDVQKS

>LOC_Os02g44370

MRAALFGAERSGVVDLGGIGGGNRGLFWPAGKGGLVVVEPRSVLDCTRSPSPRNSTSTLSSSQGGGGADSTGVAAVSESSAAAAEATKWGAPGEHGGGGG

GGGGGGKEDWSSGCELPPIPGTLDVGLVGGEGWDTMLGNAAAAAAGQDQSFLNWIIGAAGDLEQPGPPLLDNAGFGIPAVDPLGFSLDHSLSGVASDLSS

SGAHTATGGAGGGKASLGFGLFSPEATSLEQPPPPMLFHEGIDTKPPLLGAQPPGLLNHYHHQPPNPAATFFMPHPSFPEHNHQSPLLQPPPKRHHSMPD

DIYLARNQLPPAAAAAQGLPFSPLHASVPFQLQPSPPPIRGAMKTTAAEAAQQQLLDELAAAAKATEAGNSVGAREILARLNQQLPPLGKPFLRSASYLR

EALLLALADSHHGVSSVTTPLDVALKLAAYKSFSDLSPVLQFANFTATQALLDEIGGTATSCIHVIDFDLGVGGQWASFLQELAHRRAAGGVTLPLLKLT

AFVSTASHHPLELHLTQDNLSQFAADLGIPFEFNAVSLDAFNPGELISSTGDEVVAVSLPVGCSARAPPLPAILRLVKQLSPKIVVAIDHGADRADLSFS

QHFLNCFQSCVFLLDSLDAAGIDADSACKIERFLIQPRVHDMVLGRHKVHKAIAWRSVFAAAGFKPVPPSNLAEAQADCLLKRVQVRGFHVEKCGAALTL

YWQRGELVSISSWRC

>LOC_Os02g44360

MRAALFGAERSGVVDHGGGDKEELFWPAGKGGLVVVEPRSVLDCTRSPSPPYSTSTLSSSLGGGSADSTGVAAVSESSTAAAGATKWGAPGEHGGGGKEE

WGGGCELPPIPGALDVGLVGGEGWDATATLGNAAGPDQSFLNWIIGAAGDLEQPGPPLPVLQQPLIDNAGFGIPAVDTMGFSLDHPLSGVASDLSSSGAH

TATGGGGKASLGFGLFSPEATSLEQPPPSMLFHEGIDTKPPLLGAQPQFLLNHYQPQPPNPAAALFMPLPPFPEHNHQSPHLQPPLKRHHAIPDDLYLAR

NQQQSSAVAPGLAYSPPLHGPAPFQLHPSPPPIRGAMKSTAAEAAQQQLLDELAAAAKATEAGNSVGAREILARLNQQLPQLGKPFLRSASYLKEALLLA

LADSHHGSSGVTSPLDVALKLAAYKSFSDLSPVLQFTNFTATQALLDEIGGMATSCIHVIDFDLGVGGQWASFLQELAHRRGAGGMALPLLKLTAFMSTA

SHHPLELHLTQDNLSQFAAELRIPFEFNAVSLDAFNPAELISSSGDEVVAVSLPVGCSARAPPLPAILRLVKQLCPKVVVAIDHGGDRADLPFSQHFLNC

FQSCVFLLDSLDAAGIDADSACKIERFLIQPRVEDAVIGRHKAQKAIAWRSVFAATGFKPVQLSNLAEAQADCLLKRVQVRGFHVEKRGAALTLYWQRGE

LVSISSWRC

>LOC_Os05g31420

MVAQAATAAATTTAAATTAAVPMTNFQLFGSMVPVPVASMATATAPAAVAAADNGGHGSSSASQNASGSGEGQGGSMSLSLQLRPLGSTPTAAVAVSVPP

MAAAPMMAGPAAAAPAPAPPLATMAVAQNASLAAVASALAAHRRNQATHRSAALHGHLRRCAEALAASRPADADAELASIARMASSDGDAVQRVAAAFAE

AMARVVIRPWRGVSAALFPSDAGAAGDALTAWEAEFARQSFLNLCPLLHLAAVAVNEIILETTRNDKFIHIVDLGGIHHAHWVELLQGLATRRAAVRPCL

RLTIVHEHKHFLGQAAQVLAAESDRHGVPLDLHIVESSVEALKLDALGVRSDHAVVIVSTLQLHRLVGAGILSTTAPPSPAAAAAASMITSPLPPANMSS

KVDRLLRGFHLLSPRAIILTENEANHFVPSFTDRFASALPYYEQLFAAMEEAGAATVERKAAERYLLREEIKDVIACDHDGPRWARHETLGRWVVRMGAA

GFALAPAITVVTAAGRVRAVAARLPGGGDERRYGVTEGGGWLILNREEKPMFCVSAWRRQ

>LOC_Os04g46860

MRAAPFSADGNGAAELAGSIAALLWPEDKGGGGGGGGGSLLVEPRSVLDCRGSPSPPNSTSTLSSSHGSGAADSISTGVAAVSESSAAAAEATRWAAPGE

HGGGGGGELPPIPGALDVGFVAEESWDAMLGDAAAAAGQEQTFLNWIMAAPGDMEPQAPGLSQQQLLANAAGFGFPLQHHPGGVSSPAALASDLSSSGGR

SLTSSSGSNSKATSAFGLLSPEAALQPPPATTAPFHNGADMKPPLLGLPSPTLLLNQHQPTPASTLFMPFPSFSDHQQQPLLQPPPKRHHSVPDNLFLLH

NQPQPPPPAPAQCLPFPTLHSAVPFQLQPSMQHPRNAMKSTAAAAAAQQQHLLDELAAAAKATEVGNSIGAREILARLNQQLPPIGKPFLRSASYLKDAL

LLALADGHHAATRLTSPLDVALKLTAYKSFSDLSPVLQFANFTVTQALLDEIASTTASCIRVIDFDLGVGGQWASFLQELAHRCGSGGVSLPMLKLTAFV

SAASHHPLELHLTQDNLSQFAADLGIPFEFNAINLDAFDPMELIAPTADEVVAVSLPVGCSARTPLPAMLQLVKQLAPKIVVAIDYGSDRSDLPFSQHFL

NCLQSCLCLLESLDAAGTDADAVSKIERFLIQPRVEDAVLGRRRADKAIAWRTVLTSAGFAPQPLSNLAEAQADCLLKRVQVRGFHVEKRGAGLALYWQR

GELVSVSAWRC

>LOC_Os11g06180

MVLDSNELHQHDAPDVDVSINCDDRIFGQESVNLAAIQEELLEEDSLSDLLLAGAEAVEAGDSILASVAFSRLDDFLSGIPENGAASSFDRLAYHFDQGL

RSRMSSASTGCYQPEPLPSGNMLVHQIIQELSPFVKFAHFTTNQAILDAIIGDMDVHVVDLNIGEGIQWSSLMSDLARCGGKSFRLTAITTYADCHASTH

DTVVRLLSEFADSLELPFQYNSICVHNEDELHAFFEDCKGSVIVSCDTTSMYYKSLSTLQSLLLVCVKKLQPKLVVTIEEDLVRIGRGVSPSSASFVEFF

FEALHHFTTVFESMASCFIGSSYEPCLRLVEMELLGPRIQDFVVKYGSVRVEANASEVLEGFMACELSACNIAQARMLVGLFNRVFGVVFKKISLLMVYY

ISLGKNDLREPNKVIWSSLAAGCGSHGIVVLAFYAADKLLEFKPKGIETYIHPAVERAHFHKEDRMWQEYEN

>Bradi1g49630

MLAGFSLSSRHQMSSTAQRLPCGFSKRGGRGHGEGGAAAPRVAGDGRGGSSGACSFRAHPAPPVSQAVSWGATATAKPEPCVGDVGGGGWERRSRALKRA

HDEAAAVDEQEEGEYGAVARAKRTRMMGGAGDEVWFHKSIAGPMPMIQAAAGGEEEEEEEKVFLVPSAAAFPHGMIHAAAAGAGTSTLAPAKEEEYSKSP

SSHSSSSSGTDGGSSAAMPLVMASSSQPELEALELVRALMVCAESLGAGNHEAANYYLARLGESASPSGPTPLHRLAAYFAEALAIRAATTWPHLFHVSP

PRHLTDLTDDEEEDAVALRVLNSVTPIPRFLHFTLNERLLREFDGHDRVHVIDFDIKQGLQWPSLLQSLAARRPDPPAHVRITGVGPSKLELQETGARLS

AVAASLGLAFEFHAVVELRLEDVRLWMLHVKRGERVAVNCVLAAHRLLRDGGAMAAFLSLARSTGADLLLLGEHEAEGLNGGRWEPRFARALRHYAALFD

AVGAAGLDAASPARINAEEMFAREIRNAVAFEGADRCERHEGFPQWRRRMEDGGFRNAGFGDREAMQGRMIARMVAPPGNYGVRAQGDDGEGLTLQWLDN

PLYTVSAWTPAGDGAGSTVSASTTASHSLQS

>Bradi4g18390

MGTLNCSDTTSSVKLQQQQGPTSPTASFSESNIVASSTDPDAIDALAGLQALRFDGDIDGEIQSPDLAMWESLFADQIGASGADFLMSSPRRDFSPLRDF

MVSSPKRDYMVSSPKRDYMMSSPKRDYMMSSPKRDYMVSSPKREMGVSSPRRSTFSNLYSSTINQANQQSYMHGMEGSPQTQYSNLASQGNKGKSSPSPL

HKVYINNVNAHSNSGKSNGPSSLSCSSSYAHGENLPLPSMDPFLEEYKEGYLAYQLPEKAGGSESARTTAPTSSQLPTLSECLAMPEPGYGDGDDDTAAA

IVARAGIQVGGLQQTDHLYYASQFGAAEGSLSSLQHQMAKPEQWADSSSLHSMLGSVIQSEADQQQEQDSGLQLVHLLLACADLVSKGDQPSALRHLHLL

RRVASPLGDSMQRVASYFADALAARLALACPSSVVSPGGAPFPFPPSPDTLKIYQILYQACPYIKFAHFTANQAIFEAFQGEDRVHVVDLDILQGYQWPA

FLQALAARPGGPPTLRLTGVGHPAAAVRETGRHLASLAASLRVPFEFHAAVADKLERLRPAALQRRVGEALAVNAVNRLHRVPGAHLAPLLSMIRDQAPK

IMTLVEQEAGHNGPYFLGRFLEALHYYSAIFDSLDATFPADSAPRMKVEQCLLAPEIRNVVACEGAERVARHERLDRWRRIMEGRGFEAVPLSPAAVGQS

QVLLGLYGAGDGYRLNEDKGCLLLGWQDRAIIGASAWRC

>Bradi1g11090

MKREYQDGGGSRGGGDMGSSKDKMMVAAPAEEEDMDELLAALGYKVRASDMADVAQKLEQLEMAMGMGGVGAGAAPDDSFVAHLATDTVHSNPSDLSSWV

ESMLSELNAPPPPLPPAPQAPRLSSNSSTVTGGGGSGGGYFDGLPPSVDSSSSTYALRPIPSPVVTPAEPSADPAREPKRMRTGGGSTSSSSSSSSSLGG

GGTMSSVVEAAPPAAPASAAANAPALPVVVVDTQEAGIRLVHALLACAEAVQQENLSAAEALVKQIPLLAASQGGAMRKVAAYFGEALARRVFRFRPQPD

SSLLDAAFADLLHAHFYESCPYLKFAHFTANQAILEAFAGCRRVHVVDFGIKQGMQWPALLQALALRPGGPPSFRLTGVGPPQPDETDALQQVGWKLAQF

AHTIRVDFQYRGLVAATLADLEPFMLQPEGEEDPNEEPEVIAVNSVFEMHRLLSQPGALEKVLGTVRAVRPRIVTVVEQEANHNSGSFLDRFTESLHYYS

TMFDSLEGAGSGQSEISPGAAAGATDQVMSEVYLGRQICNVVACEGPERTERHETLGQWRGRLGQAGFETVHLGSNAYKQASTLLALFAGGDGYKVEEKD

GCLTLGWHTRPLIATSAWRMAAP

>Bradi1g23350

MANTPTSRMMHPFGSVPRQNLKQFQYSDNAQHTCHPYQSSPDTHVVPQHDYSLKSHSPDAGFENQVTHMKYTLDSSAEVGCMRHDSPSSHSFTPRSDSSS

PLSQEDSHSDSTNGSPVSASCVTVTEDPNHLRQKLKDLEAVMLGPDSEVVNSLESSIANQLSLEPEKWVQMMRFPRDNLKELLVACARAVEEKNGYAIDM

MIPELRKMVSVSGEPLQRLGAYMVEGLVARLASSGHSIYKALKCKEPKSSDLLSYMHFLYEACPYFKFGYMSANGAIAEAVKGEDRIHIIDFHISQGAQW

ISLLQALAARPGGPPTVKITGIDDSVSAYARDGGLDIVGRRLSHIAGLCKVPFEFHAVAISGDEVEERHLGVIPGEALAVNFTLELHHISDETVSTANHR

DRILRLVKSLSPNVLTLVEQESNTNTAPFVQRFAETLDYYTAIFESIDLTLPRDDKERINMEQHCLAREIVNLVACEGSERVERHEVFGKWKARLMMAGF

RPSPLSSLVNDTIRTLLQSYSVNYQLAERDGVLYLGWKNRPLVVSSAWH

>Bradi1g32070

MSLHERAAIRLVHMLVTCAAAIQAGDYGAAATYLIEARTILVNTIPTSSGIGRVTIHFATALAHRLNSASPNSSLSPSSSSPSANNHASEQYRQFYDQVP

HLKFAHFTANQAFLEAFRGHDRVHIIDLAIMCGLQWLSLIKAFSIWPGGPPSIRITGVGPTHTGSRDELREVGLMLTEHARVLNVPFSFHIVTGDNLEGL

KPWMFHLVQGEAIAVNSICQLHRLLVDPDAASTSLPPPIDTVLGWITSMRPKVFTVVEQEADHNKPTLVERFTNALFYYGAMFDSMEAVATRSRTGAAER

GAEVHLKREIFNIVCNEGSSRVERHETLPRWRARLRRAGLAQVPLRPNNLRHASMLLHDLSGDGYHVMERGDGLMLAWHNNPLFSVSVWHIMEEELEDNK

NKVVRRSSTHLATAGAMLSMQ

>Bradi1g24310

MIQGVLSRASAADAAAMKAKRVAPIPEDEEAAMVGARGKRLQLHSSGVQQQQEAPAEEGKVVVVEAAAETRGLRLLSLLLRCAEAVAMDSLPEARDLLHE

IAELASPFGTSPERVAAYFGDALCARVLSSYLGAYSPLALASAQQSRRVASAFQAYNALSPLVKFSHFTANKAILQALDGEDRVHVVDLDIMQGLQWPGL

FHMLASSRPSKPLLSLRITGLGASLEVLEATGRRLADFAGSLGLPFEFRPIEGKIGHVADTDVLLLGRDEGEATVVHWMHHCLYDVTGSDAGTVRVLRSL

RPKLVTIVEQDLGHGGDFLGRFVEALHYYSALFDALGDGAGPEEEEAQQRHAVERQLLGAEIRNIVAVGGPKRTGEVRVERWGDELRRAGFRPVSLSGGP

AAQARLLLGMCPWKGYTLVEEDGCLKLGWKDLSLLTASSWEPTTTDGDEEDDLGAAARQEECHGS

>Bradi1g47900

MSLHEHAAIRLVHILVTCAAAIQAGDYGVAVNNLAEAHTLLATTIPTSSGIGRVTSHFATALAYRLFSASPHSSMPPSSSSPSPNNQAGEQYRQFYDMVP

HLKFAHFAANQAILEAFQGHDQVHIIDLAIMRGLQWLPLIQAFSLQSGGPPSIRITGVGPTPTGPHDDIQEVGLLLTEHARVLNVPFSFHSVTCDSLEGL

KPWMFHLIHSEAVAINSIFQLHRLLGDPDAASTSLPPPIDTVLGWITAMRPKVFTIVEQEADHNKPELVERFTNALFYYGVAFDSMEAIVPRSQAGTAGL

AAEAHLQREIFDIVCNEGSGRVERHETLQCWRGRLRRAGLAQVPLGPNNLRHASMLLRIFSGAGYHVMERGDGLMLAWHGNPLFSVSVWHVMEEELEDNK

NNVVRHVTTGAMLSMQ

>Bradi3g24210

MWAKASDHTYRRSDDQRMAYYNNSVPSRENGRFYVAQSHIDLHYTSSDDGSQKIRSNPQVFEPQYCTLESSSGNGVYPTQSSTSSHSISPISGSPLSQHD

SHSDHIYGSPPSASCVTEVADLQVKLKELEDAILGPELDITSDSPESSLQAINPLKPDNWRQLLGIYTGDLKEVIIACGKAVAENDVFATELLISELGQL

VSVSGDPMQRLGAYMLEGLVARLSSSGSKIYKSLRCKQPTGSELMSYMSLLYEICPFYKFGYMSANGAIAEAIKGENFVHIIDFQIAQGSQWVTVIQALA

ARPGGPPCLRITGIDDSDSIYARGGGLDIVGTRLYKVSRACGLPFEFNAIPAASHEVHLEHLDIRPGEVIVVNFAYQLHHTPDESVSMENHRDRILRMIK

SLSPRVVTLVEQESNTNTAPFFPRYLETLDYYTAMFESIDAALPRDDKRRMSAEQHCVARDIVNLIACEGADRIERHEVFGKWKARFAMAGFRQYPLSSV

VNNTIKTLLDSYNNYYRLEERDGVLYLGWKNRVLVVSSAWW

>Bradi1g25370

MDLHQLLKYRLTGANVLYEIPTENNLTNSSWPASPLKLEYNNSPYTPLSTQLECDNLSALSNTPDNQSSTETISAQPISPLEADSSYIQPGSHLRENIQV

RADPLYARSRHNMQHALREIETVLMAPDADDATTSAKHEFEETKPAQLMRQRSRTWSHESRQPLPGVVRPQFASGGYPMASYEFRPEKRQRELRDDPQII

VKQLLTKCAEALSEDRTEEFLKLVQEARGIVSINGEPIQRLGAYLLEGLVARHGNSGRNIYRALRCRKPESKELLSYMKILYNICPYFKFGYMAANGAIA

EALRSEDNIHIIDFQIAQGTQWITLIQALAARPGGPPHVRITGIDDPVSEYARGEGLEIVGNMLKGMSKEFNIPLEFTPLPVYATQVTKEMLEIRPGEAL

AVNFTLQLHHTPDESVDVNNPRDGLLRMVKGLSPKVTTLVEQESHTNTTPFMMRFAETMDYYSAMFESIDANLPRDNKERISVEQHCLAKDIVNIIACEG

KDRVERHELLGKWKSRLTMAGFKPYPLSSYVNSVIKKLLACYSDKYTLEEKDGAMLLGWKKRKLISASAWH

>Bradi2g57940

MGMSPSPFTTSYCLTEQDASICTETQELDYHHSYYYGIEDVSLDEVELELAASSGRAHKATKVDYHSSPYHISWPPPPQQASADVESSRVRKKQFRDVLE

SCKQKVEAMEAMEQHSPPLSGGGVGFQDQQGDSGHGVAVGGEGSSNSGGGTDGMRLVQLLVACAEAVACRDRAQAASLLRELQAGAPVHGTAFQRVASCF

VQGLADRLALAHPPALGPASMAFCIPQSSSSASCAGRGEALAVAYEVCPYLRFAHFVANASILEAFEGESKVHVVDLGMTLGLDRAHQWRALLDGLAARG

VARPARVRVTGVGARVDAMRAVGLELEAYAEELGMCVEFRAIDRTLESLHVDDLGVEADEAVAINSVLELHCVVKESRGALNSVLQTIRKLAPKAFVLVE

QDAGHNGPFFLGRFMEALHYYAALFDALDAALPRYDARRARVEQFHFGAEIRNVVGCEGAARVERHERADQWRRRMSRAGFQSMPIKMAAKAREWLEENA

GGTGYTVAEEKGCLVLGWKGKPVIAASCWKC

>Bradi2g45117

MAMGSFPFQWSMDPAPLSSGVDGSLLPSFLPPPPAAAVPDDGTAYYAASDMHSVPCLPELAAPFQSRDSAAAELAMRRAEEEVAGIRLVHLLMSCAGAVE

AGDHEAAAALLADANASLAAVSTSSGIGRVAVHFTDALSRRLFLSPPAGATPTPPAAADPEHAFLYHHFYEACPYLKFAHFTANQAILEAFHGCDSVHVV

DFSLMQGLQWPALIQALALRPGGPPFLRITGIGPPSPPGGRDELRDVGLRLAELARSVRVRFSFRGVAANTLDEVHPWMLQIAPGEAVAVNSVLQLHRLL

ASPADLQAQAPIDAVLDCVASLRPKIFTVVEQEADHNKPGFLDRFTEALFYYSAVFDSLDATSAGASSNAAMAEAYLQREICDIVCHEGAARTERHEPLS

RWRDRLGRAGLRAVPLGPGALRQARMLVGLFSGEGHSVEEAEGCLTLGWHGRTLFSASAWRAAGDGGGGEENIDSNNSNIGGGSSGSDSNNSSCGVVGAA

NMFL

>Bradi1g10330

MAYMCADSGNLMAIAQQVIQQQQQQQQHHQQRHHHHLAPPMPSAPAPPHAQIPASLPYGGASAGWPQAEHFFSDVFGASAADAVFSDLATAADFDSDGWM

ESLIGDAPVFQDSDLDRLIFTTPPPPVPPPAEPAAAQAENAPASLPLAAATTPVACSPASSSDTSCSAPILQSLLACSRAAAANSGLAATELAKVRAVAT

DSGDPAERVAFYFSDALARRLACGGAASPVTAADARFAADELTLCYKTLNDACPYSKFAHLTANQAILEATGAATKIHIVDFGIVQGIQWAALLQALATR

PEGKPSRIRISGVPSPFLGPEPAASLAATSARLRDFAKLLGVDFEFVPLLRPVDELDQSDFLIEPDEVVAVNFMLQLYHLLGDSDEPVRRVLRLAKSLHP

AVVTLGEYEVSLNRAGFVDRFANALSYYRLVFESLDVAMARDSQERVMMERCMFGERIRRAVGPGEGADRTDRMAGSSEWQTLMEWCGFEPVRLSNYAMS

QADLLLWNYDSKYKYSLVELQPAFLSLAWEKRPLLTVSAWR

>Bradi2g56910

MSFVGRVDPSTTYADNIYVHKFGTPNSNFAARRFGSDTQLFHYGPEPFNSEDYEHMGFTEAPSSAFQNSYYNQQASLTPYHVMANGRSPSVSDNMANSCS

DTAKDSPVFSNVSQQNSQSISDNHSSGLEVEFDPEIRLKLQELEHALLDDGDDILFEISQTGCINDEWADPMKDVLLPNSPKESESSISCAGSNNGEART

PKQLLFDCATALSEYNIDEAQTIITDLRQMVSIQGDPSHRIAAYLVEGLAARIVASGKGIYKALTCKDPPTLYQLSAMQILFEICPCYRFGFMAANYAIL

EACKGEERMHIIDFDINQGSQYITLMQFMKNDANKPRHLRITGVDDHETVQRTVGGLKVIGQRLEKLAEDCGISFEFRAVAANIGDVTPAMLDCRPGEAL

VVNFAFQLHHLPDESVSIMNERDQLLRMVKGLQPKLVTLVEQDANTNTAPFVTRFREVYDYYSALFDSLDATLPRESPDRMNVERQCLAREIVNILACEG

PDRVERYEVAGKWRARMTMAGFVPCPFNNNVIGGIRSLLNSYCDRYKFEEDHGGLHFGWGEKTLIVSSAWQ

>Bradi5g10320

MNLTLSLGVDGGGAAAKKRKVRDGDVVGAAGDGGDYGRIMRLLQARDRVAKVKLGDQYGNDDDGSRGGGGLRLMRLLLSSAAAAEVGDAHAAAVALREVS

RHASFRGGDPVQRVAAHFADALASRLLLSPRAPAPIVAPRATPAEQSFLAFTMFYQASPFYQFAHFTANQAILEAFEAGARRSLHVVDFDVSFGFQWPSL

IQSLSDAAATTSPQSSDNSDAEPQPAAAFYSLRITGFGTSADELRDTEARLARFAAGCPNLRFEFEGIVNNNGSALHGLKNIKVDPDSTVVVNLVFPAAT

TTTTSSLACVRSLNPSLVFLIDKDVQVQDSASLLPRFAASLRYYAAVFESLHECLPADSAERLAIERDHLGAEIGHAMASLQGDRRQHGGDWTEVMEGAG

FEGARLSSRTVSQAKLLLKMKSGGCGGFRVIECGGEDKAMSLGWRDRALITATAWRPCRKQNDL

>Bradi1g36180

MPLLRLPHLRPLLTLLAAAAARRRQASGEDRRRRRSADVMLGSLHSSSSSSDTDTTTNNNNSGGVEAAAQGGIGHALQQAAAPPSARDLVLACADLLHRG

DLAAARRAAEILLSTSSPRGDAADRLAYHFARALALRVDAMAGHVVPFASSSGSPRQSSGGPGASAGPYLAFNQIAPFLRFAHLTANQAILDSIPAGARR

VHILDLDAAHGVQWPPLLQAIADRADPALGLGPPEVRITGAGADRDALLRTGNRLRAFARSIRLPFHFAPLLLSQSTKHQQEDSVAGSGSGSSHAGSGSS

QQLVELHPDETLAVNCVLFLHKLGGQEELAAFLKWVKAMAPAVVTVAERESSHPTAIEHPAMAMGIDGREQDELLPRRVGAAMDHYAAVFEALEATVPPG

SRERLAVEQDVLGREIEAAVSGVGGRWRGGLERWAAAARATGFAARPLSPFAVSQARLLLRLHYPSEGYLVQEARGACFLGWQTRPLLSVSSWH

>Bradi3g07160

MLGPPEPDDDDATASASPPHHGPDPTPRELVLACAGLLHRGDLAAARRLADQALSAADPRGEASDRLAHHFARALALRAPDNGGDSPSPAPVASTSSSSS

SSVSSAAAAAAYLAYNKIAPFLRFAHLTANQAILEAFFSSHADRPQHHSRVLHIVDLDAAHGVQWPPLLQALRDRAADDPPVEVRVTGAGPDRDALLRTG

NRLRAFAASIDLPFRFHPLLLPCPGAASAGLELELHPDEETLAVNCVFFLHRLSNDGELATFLRWVNSMNPAVLTIAEREGSSEPADELPRRVAAAMDLY

YAAVFDALEATVPPGSAERMAVEEEILGREIDAVVAGGSGSRTRGFEAWAAAARGAGLSPRPASAFAAAQARLLLRLHYPSEGYAAEEAHGACFLGWQRR

PLMSVSSWQ

>Bradi2g54670

MVMDATLHDWSAPLPGSNSKCDGHLPIYPQIPAANGFTVEELEALLVLPSDGVDAEGYLNAAAASTDSASPPREVSVSSAPPPAGPGQAPPDDSEGFSDI

VLGYINRMLMAEDIDEKFEHYPSLLSAEKPFLDILADHPPYSGGSTVDSPDGSSVTNSCNSLGSCHCAAPSSGLGQTPPFLEFPTAAFLQPPRLYQDPSP

ESCVVDAGGAWPYDPTEFYQLHTNPVPETLLSQSSSFASSNGSSVAFSEGFESWLSTAGVVPDVGLSNFVLQNQQAAQFSRGFEEASRFLPVESKLVIDV

ESLLSVASLQAGVKEEKFVEVKTEKAGAEVAVHRGKKHFYGDDLDVEEGRCCKHSAPAIIDSDHLVREMMDKVLLCNGETCSKGVKELREALRHDVANHP

SGAHGKGSGHGKGRGKKQSKQPKKEVVDLETLLIHCAQSVSIDDRRSATELLKQIRQHASANGDGDQRLAHCFANGLEARLAGNGSQIYKSFTISRLACT

DVLKAYQLYLAACPFKKISHYFANQTIMNAVEKAKKVHIIDYGIYYGFQWPCLIQRLSTRAGGPPKLRITGIDTPQPGFRPAERIQETGRYLSDYAQTFK

VPFEFQGIASQFEAVQIEDLHIEKDEVLIVNCMFKFKTLMDESVVAESPRNIVLNTIRKMKPHVFIHGITNGSYNAPFFVSRFREALFHYSAAFDMLEAN

IPRDNEQRLLIESALFNREAINVISCEGLERMERPETYKQWQTRNQRAGFKQLPLNQDIMKRAREKVKCYHKNFIIDEDNRWLLQGWKGRILFALSTWKP

NHHSS

>Bradi2g60750

MIQQQDTEGSSSSVTSSPLQNFSAMPLHPGTAAAHTPAAAAPPWMLRELRSDERGLCLIHLLLNCAAAAAAGRLDAANAALEHIAALAAPDGDAMQRVAA

AFAEALARRALRAWPGLCRALLLPRPSSPTPAESAAARRHFLDLCPFLRLAGAAANQAILEAMEGEKIVHVVDLGGCGADATQWLELLRLLAARPEGPPH

FRLTAVHEHKDLLSQTAMVLTKEAERLDVPFQFNPVITPRLESLDVESLRVKTGEALAISCSLQLHCLLATDEDASPDSSSAAAEKGSAAAERRSSPESS

GLLSPPTTRLESFLGALWGLSPKVMVVVEQEASHNTAGLTERFVEALNYYAALFDCLEVGASRASVERARVERWLLGEEIKDIVACDGAARRERHERAER

WAARMEAAGFAGVPLSYYALLQARRAAQGLGCEGFKVREEKGTFFLCWQDRALFSVSAWRGRRFD

>Bradi1g03620

MGSCEDVDYGDIFSVTNPAAAPNYMFNFSPLQQQQQHPHLRFPAAPHGFHPGSVGPSPPSGPPALGQTPSPSSSITTAELDAPEDVTGDAVLAYINQFLL

EDEDEDSCPGASSAATDPQQDSALLAVEKPFVDIIADAAPAGACRQNNNPWMDPCCGFAGNDAGVFVGRQTQTSSCQIVPCEEEPVKLEGDCAVHKGRKN

RHGDRGVELEEEEERRSKQSAVCEEETVREMFDKVLLCDGVNCILQSPLPAEAEVSAVYVKGSGNKRGRKKGKPGAAAAEEESVDLTTLLIHCAQATAID

DYRNSVELLKQIRQHSSALGDAGQRLAHYFANGLEARLAGTGSTIYRSLVAQRNTTADILKAFELYVTVCPFNKLSKFFSNEAILNASKDVTRLHIVDYG

IRWGFQWPIFMQRIARRLGGPPRLRITGIDLPQPGFRPAERVEATGQRLREYARTFKVPFEYHAIAAKWDTVRVEDLKIDKDELLIVNCTFLMRHMMDET

VTDDSPRMRVLNTIRKMNPHMFIHGVVNGTYNAPFFVTRFKEALFYFSSLFDMLETTSSRLDEHRLLIERDFFGREALNVVACEGTERVERPETYKQWQV

RNIRAGFRQLPLNQDTVKKARYKVTKSYHKDFLVDEDNKWMLQGWKGKVILAQSTWKPS

>Bradi4g43680

MASTPQPPRGDLAARLEAAAVAPPPAAAAQDLAALGLAAARSLRHRPPRHGGQMQSGPVEHQSISGSLIHSMIQFCGAPKYFMFNDPQPVIPPQMAAQPN

PSLPVATALSRATNNETDNPEDWEFISDESLNYISRMLMEEDIDEKVSMYQEESAMLRATAKPFYDILGHKFPPSPDRNLPWSLDSPGESSSSSHAQSLS

SVITSCSSGAVEDSNQSRNIGHCEQLEAYRGLHGQSFQPVVSPSSGVSDAAEALADPLITNGRIPDNLFESLPNWDFRRGVEEAQKFLPASDKLVIDLEA

ADVSTSQVGKDISLNGSKPEVLNAKKNRQSEDLDLIEGRSIKQSAFCSDEPDWIEMFDGLLRQTEQKATVLREKMRTEASKNSQVTQTKATTGVRTRGRK

PTKNDVVDLRTILIHCAQAVAADDRRTANELLKQIKQHSKVNGDGSQRLAFCFAQGLEARLAGTGSQQYHRLVAKRTTASDMLKAYHLYLAACPFKRLSH

FLSNQTILSMTKNASTVHIIDFGIYFGLQWPCLIRRLSKREGGPPKLRITGIDVPEPGFRPTERIEETGQRLAEYADRLGVPFEYHGIASKWETIRAEDL

KVGKDEVVIVNCLYRFRNLIDETVAVDSPRNRVLNTIRQVNPAIFIHGIVNGSYSVPFFITRFREALFHFSALFDMLEATVPRDDDQRRLIERDLFGREA

LNVIACEGSDRVERPETYKQWQVRNLRAGFVQSPLNQEIVAKAKVKVKDIYHKDFVIDEDSGWLLQGWKGRIIYAITTWKPNNN

>Bradi4g09235

MATTQEELLGVVDSAPLPPSVVLDLPPMTHGDSKRPQDDLSMAYISRMLMEDDIIDKFSYQYPEHLKLLQAEQPFAQILSADATTSSNVEESSGSDVLAS

ALLPSKVQDSAALSNSTGAAQSSSTLFPSESSTNMDMLSSMAFYKGMEEANMFLPRCNRMVDGRGRKNRFDMDGEMEGGMGRSSKQIVLVHTDSAEETTA

LKKLDRLVLYGSDTCSGEMQEILINKEKMAAEKSIRRRGRGSARQMVVTDLETLLIRCAEAMTSNDRRSASELLERIKRYSSPTGDARQRLAHYFAQGLE

ARLVGTGSQLYRSCMGRRTSIVELIKAYHLYNATCCFVKMAMLFSNKTIYNAVAGRRKLHIVHYGINSGLQWPKLIRWLAEREGGPPEIRITGINMPQPG

FNLAEQIKETGQRLSNYASKFGVSFKFHAIIAKLEAVHAEDLHIDPDEVLIVNSLFQFRILMDESLSFDNVSPRDMVLNNIRKMKPSMFIHGIANGSHSA

AFFMTRFRQALSHFTALFDMMETIMQGNYDKRLRVERAIFAWCAINMIACEGVDRVERPQNYREWQVRKNRAGLRQLPLDSDTVLMLKNEVKNQYHKHFM

IDEDHRWVLQGWKGRVLYALSTWAADDAGGSELS

>Bradi1g15123

MEEPFYKLLGQKYPVLPELSPLCRCGRLKNLNGNISKLTGQSCSTCSVNISSSNDHSNENTQDFQAPWTLSSIVGEAFTQGTERMEIGLNINGLAITKKP

SRDNHPLQISAQDTMRHASFEVRGRKGYPGIDGFDLLEERSNKKFAIFSDELIRNELFDKVLLCSENKLVGEGIVLQGTMPYKCAQKDQGRKSALQKTRG

RTQQKKEVVDLGTLLINCAEAVSLNNGSVASEILKKIRRHSSPCGDDSQRLAFYLADCLEIRLAGNVSQISQKTITAPIDVAHILKLLHLCYTVCPYLRA

SYYLSNKTILDVSKGKPRVHIIDFGICFGFQWPSLFEQFANREGGPPKLRITGVEIPQPGFRPDEMNKNTWLRLVEYANMFKVPLEYRLISSKWEAISIE

DFYIQKDEVLVVNCINRMKTLGDETISIDSARNRVLNTIRMMKPTVFIHGVVNGSYSTPFFLPRFKEAMYHYTALFDILDRTTPRDNKTRMMLEGHLYKF

AILNVIACEGSERTERPESYKKWKVRSLRARLEQLPLNPAIVKQTQCMVRQIYHKDYFVDEEDQWLVLGWKGRILYAISTWKPNESDDG

>Bradi2g22010

MQFHDPHQYMYSSSSSTAGGSFLPHHHAAFIKDVAPAEPLVEILEEAPGAAGAPAMQEERKVVDEAAEEEEGEEAAAESHGVRMIALLMECAVAMSVGNL

ADANGALLELSQMASPYAASCGERLVAYFARALAARLMSSWVGVCAPLSLQQHDDAGIVIHAAFRAFYNVAPFARAAYLACNQAVLDAFRGQRAVHIVDL

DMVPGGALQWLSLLPALAARPGGPPALLRVTGFGVSAALLHDAGNQLAGLAGKLGLPFEFYAVAKRPGDAAAAVSSGLLLPGKRPGEAVAVHWLRHALYD

AAGDEAAAIRLARWLEPRVMTVVDQERSLSSSSSSGAAADDGGSFLDRFVSALHHYSAAFDSLGAARPAGDDASRHLAENGMLGREIGNVLAIAGPSRSG

RERLLPGSWQAELARHGFLRARWGSGGARAQMLAGACPAGLGYTVADDAHDGTVRPNLGPAAGIAVAFRQEISLTICENCYFFVWPENSYSTLTL

>Bradi4g09180

MAGGATPEEEHVRQSFLARLEPPSPSLFLDLPPTPRSSSSFDDMALPYISRLLMEEEDMADHFFYLYPQHPALLRAQLPFAQILVNNSSAGSGSGSVPSP

SSSDSSSASPHDAVQIAGPPPCAPDGLLPGGDQDMLNLAFLKGMEEARKFLPRNTGLPPVVAASAEDGLLKTKRANSEPEEVGRASKLMAPELEEEEDGA

RELLDEMMLGENEICMKGVPNLQVAEPVKKTRKGTGQRGRPRKAAVDGEMVDLHTLLLQCAQAVSTDNQRGASELLKKIKQNSSPTGDAAQRLAHYFSVG

LEARLAGRGSRLYESLMARRTSVVDVLKADQLYMAACCCKKVAFVFANKTICNAVAGKSRLHIVDYGINLGLQWPGLLRMLAAREGGPPEVRITGIDLPQ

PGFRGASHVEDTGRRLSNFARVFSVPFKFCAIAAKRETVRPEDLNIDPDEVLVVISLCHFRLLMDENLGFDSPSPRDQVLNNIRKMRPNVFIHGILNGSY

GATYFLTRFREALFHYSAQFDLLDATVPRDNSGRLLLERDIFGRSALNVIACEGADRVERPETYKQWQLRNHRAGLSQLPLNPEVVKLVLDKVRGNYHKD

FVVDEDQRWLVHRWKGRVLYALSAWVADDAT

>Bradi2g52227

MDATPEEFLRTGGYLVDPEPLSPSLFLDLPPTPRADGPPAAAASAAADLDLDFISRMLMEEDIDDKFFYQYPDQPALLAAQRPYAQIISDTTSSGSPTGT

TATSSDDSVINSNTATSSSDDTTNYTWPYDPIELSQLLRSPPYDGDMGFGGLVTDSPADEDTRSSFFPGDGAPTAEFQQSAALFKGAAEGGTTSFGQNGG

SPGTQNSAILNDPAVEEEAKPAVFYDGDGDQGALVSAFFSGQTGSDMEMLNMAFLKGMEEANKFLPTNNTLPEVISDKPALRGFTVKKEELVDGTLTSGN

GRGRRYRHDVDDLEAETGRNSKLMMPEHEETGAREMFDEIMLEGYEMCMKGMEDLRVAMDSEAKKNNTKGTGKAARAKRGTSEVVDLHTMLIHCAQAVAA

GDRRSATELLKQIRQHSGPRGDATQRLAHCFAEGLEARLAGTGSQVYQSLVAKRTSVVEFLKAYKLFMAACCFKKVSFGFANLTILDAVVGKSRLHIVDF

GVQYGLQWPGLMRLLAERDGGPPEVRITGIDLPQPGFRPACQIEETGRRLSNCAREFGVPFKFHSIAAKWETVRAEDLGIDRNEVLVVLCQCGLSNLMDE

SLVTDGLSPRDLVLRNIRNMRPDVFIQCVANGTYGAPFFVTRFREALFFYSAHFDMLDATIPRDNDERLLIERDIIGRAALNVIACEGADRVDRPETYKQ

WQVRNHRAGLRQLPLNPEIVKLAKEKVKNHYHKDFIIDVDHQWLLRGWKGRVLYAVSAWIAEDDS

>Bradi4g09155

MTHGDSKQPQDGPSLAYISRMLMEDDIVDKFSYQYPDHPKLLQAEQPFAQILTATASTSPNAEESSASNTISSALMLSKVQDPSFFSNGTDAVGPSSTFF

SIESSTNMNKMSSMAFFKGMEQAKMFLPSDNLMVDGRGQKKRFDMDGETEAGMDRSSKQIAMTHTDLEDTALKKMDRLILNEYDGYRGEMHEELITLDNE

NKAAQQSIRMRGRRSAKKTMVTDFETLLIRCAEAVSSNDRGSASELLMRIKRHSSPSGDARQRLAHYFAQGLEARMAGTGSQLYHSLIGTRTSTLELIKA

YHLHMATCSFLKVALIFSNYTIYNAVAGRRKLHIVHYGINTGYQWARLIRRLADREGGPPEVRITGINRPQPGFRPAELIEEAGHRLSKYARKCGVPFKF

HAVAAQPEAVRAEDLHIDPDEVLVVDSLFDFRTLMDESLTFDRVNPRDVVLNTIRMMKPSVFVHAIVNGSYSAAFFMTRFRQAMYFFTALFDVMETTFPR

DNAKRLLLERDIFARSAVNMIACEGTERVERPQNYREWQARNQRAGMRQLPLDPDILLMLKEKVKNQYHKHFMINEDQGWLLQGWKGRVLYALATWTADD

TSGPV

>Bradi4g03867

MDSPGSDDPSLYLYLSDLVPGSPPSSLSAYLDLPPTPHHEQPQQQQQRSDYDDLGSGGGGGGGGGGGDDKSSSTPEDLVLPFISRMLMEEDIDDKFFYDF

PDNPALLQAQQPFLDILSDPSSNTTSSDSNNNNNPCSPSDASFSPAGPLPPTPAAVDSYDSQPFQYDIDPAAFFSAGANSDLMSSAFLKGMEEANKFLPS

QGNLVIDLEASSGQFLKGVEEGNKFLPKEDQLVVGFNGRAAPVSAPTAVASVKKEEPVDAVSANSHGGSRGRKNPYRDDELERELGRSNKQSALQGEDIS

ARELFDRVLMPSPEMCVEQMQSLRIAMQEAVAKNDTGSGKVGNGKGRGRRGGSDVVDLRTLLIHCAQAVATDDRRSATELLKQIKLHARHDGDGTQRLAH

CFAEGLQARLAGTGGLVHQSLMATRISAVDMLKAYQLYMAAICFKKVCFIFSNFTIYNASLGKKKIHIIDYGIQYGFQWPCFLRRISEREGGPPEVRITG

IDLPQPGFRPAGRIEETGRRLSKYASEFKVPFKYNAIAVTNMESLRKEDLNIDPEEVLIVNCLFQFKNLMDESVVIESPRDIVLNNIRKMQPHAFIHAIV

NGSFSAPFFVTRFREVLFYYSALFDVLDTTTPRDNEQRMLIEQNIFGRAALNVIACEGADRVERPETYKQWQVRNQRAGLKQLPLNPDIIETVRDKVKDC

YHKDFVIDVDHNWLLEGWKGRILYAISSWVANDASSHF

>Bradi4g09190

MAGGATPEEEFLRQSFLARLEPPSPSLFLDLPPTPGAAAAGEAGPSSSSFDDMVLPYISQLLMEEEDMVDHFFYLYPQHPALLRAQLPFAQILQDNSSAG

SAPTPSSSDSSSPADCRPELRAVQSSALPDGLLTGGDQDMLNLAFLKGMEEARKFLPPNTGLPPVAAKAEDGLLKKKRGNSEPEEAGRASKLMAPELEEE

EDGARELLDEMMFDQNEICMKGVQNLQVSEPVKKTRNRKGTGQRGRPKNASADDEMVDLHTLLLQCAQAVSTDDQRGAGELLKKIKQNSSPTGDAAQRLA

HYFSIGLEARLAGRGSELYESLMTRRTSVVDVLKANQLYMAACCCRKVSFLFSDKTIYNAVAGRSRLHIVDYGINLGLQWPALLRMLAAREGGPPEVRIT

GIDLPQPGFRGAYHIEDTGRRLSNFARVFGVPFKFHGIAAKRETVRPEDLNIDPDEVLVVISLCHFRHLMDENLGFDSPSPRDQVLNNIKKMRPNVFIHG

ILSGAYGATYFLTRFREALFHYSAHFDQLDVTVPRDNHGRLLLERDIFGPSALNVIACEGADRVERPETYKQWQLRHHRAGLSQLPLNPEVVKLVLDKVK

DNYHKDFVVDEDQRWLVQRWKGRVLYALSAWVADDAA

>Bradi4g09170

MPVTSEELLGLTESVPPSPSLYLDIPPTPYDASVGDLVLPYIARILMEEDIDDRFFYQYPDHPALLRAQQPFAQILDEAKNLLSNEDGDMERMNSDVSLQ

GAHGGSMFLAGKDIRNWAILDGAEVDNNGDQRKLSLCNEDMLNLAFLKGMEEANKFLPRDNNLQVSAFSIGQAREMLVRSTSGRRRCDVEEVVGRASKLM

VLDLEEDGAHELMQKLMLNSCEFRGEAMQKLRITMEKTEAERRSMKAGRGRQQRKRGDTVDLRGMLLCCSQEVATGNHQGASILLKQIRQHASARGDAAQ

RLAHCFAQGLEARLAGTGSQVYRSLVEKHTSAMEFLKGYELFMAACCFKRVAFTFSSMTIFNAVEGKSRLHIVDYGLHYGCQWPGLLCWLANRGGRPPKV

RITGIDLPQPGFRPSKRIEETGKQLSNCARQFGLPFKFHAITAKWETICAADLNIDPDEVLVVNELFNFNTLMDESLVTDRPSPRDVVLSTIRGMRPDVF

IQGVVNGSSGPFFLARFRESLFFHSSVFDMLDATTPRDSEHRLVLERDMFGQCALNAVACEGADRVERPETYKQWQLRNQRAGLRQLPLRPSVVEVATDK

VKSLYHKDFLVDVDQGWLLQGWKGRILYAHSAWVADDTSSDD

>Bradi4g09160

MPITPEELLETVPPSPSIYLDIPPSPYGGNVGDMVLPYISRILMEEDIDERFFYQYPDHPALLRAQQPFAEILNEARNLLSSEGSDIERMNSDSSLQGGC

GSSMFLAGKDDIQSRAIFDAAEVDNNGYQSKLGLSNEDMLNMSFLKGMEEASKFLPRDNNLQVSAFSVGQPKEMFDRSASGRERCDGEEVVGRASKLMVI

ELEEDGAHEMFEKAMLNSCDLSGETMEKLHIDMENAEANRRNKKAVRGRQQGKRGDTVDLRALLLSCAQEVAISNHQGAGNLLKQIRQHASATGDATQRL

AHCFAMGLEARMAGTGSKVYKTLVAKQTSAIEFLRGYELFMAACSFRRVALTFSSMTIFHAMRGKKRLHIVDYGVHYGCQWPGLLCWLASRDGGPPEVRI

TGIDLPQPGFRPAKRIEETGQRLSNCARQFGLPFKFHAIAAKWETIRAEDLNIEPDEVLVVNDLFNFNTLMDESLVTDRPSPRDVVLSTIRGMRPDVFIQ

GVVNGSSGPFFLARFREALFFHSSVFDMLDATTPRESEHRLVLERDMFGQCALNAIACEGADRVERPETFKQWHLRNQRAGLRQLPLRPIVIEVATGKVK

SLYHKDFVVDVSQGWLLQGWKGRILYAHSAWVADDTSSDD

>Bradi1g67340

MEDVVGDLEISGCSSSTATSSPSSSSLVDDDMGLYAWNALSPVADWGPFCSDDGGQDLHGLIESMLSDDALVGADPASSLFHQAGPCYSNGSDPSSTTTT

NPGTPVYDDPAQAQAECSPEKGLRLLHLLMAAAEALSGPHKSRELARVILVRLKDMVSSTSDAAASNMERLAAHFTDALQGLLDGSHPLSGAGKQAAAMA

AAASSSSLHHHHYNASDVLTAFQMLQDMSPYMKFGHFTANQAILEAVAGDRRVHVVDYDLAEGIQWASLMQAMTSRPDGVSPPHLRITAVTRGGGGGARA

VQEAGRRLAAFAGSIGQPFSFGHCRLDSDERFRPATVRMVKGETLVANCILHQAAATTTVRRPTGSVASFLTGMAALGAKVVTVVEEEGEASSEKESDEK

EEGAAGGFVARFMEELHRYSAVWDSLEAGFPTQSRVRGLVERVILGPNIAGAVSRAYRGGMDGGRGGWGEWMRGSGFKAVPLSCFNHSQARLLLGLFNDG

YTVEETRPNKIVLGWKARRLLSASVWAPPPLSVPSSPAEAVFQPIGMAMAPAASGFGRMEFDFIDSFLVEPAYALV

>Bradi5g19190

MEQPGGPWRDPRQGYLYGVGSAMQTPLQQQQQQRRRPDTAAVGGVLKRSLGEIERWQHQRQVAMQQELYMRAVRQRTAVASAISPLSSADIAKVLGRGSS

QPLVLSGSSLGGNLASPSSTLSSLTTASRAAIPLIQPQLHRQVPFVPSSEHALGVTRAPAPSATGSELSILQELEKQLLFDDDDEAEPAMSGTGSAVTSS

EWEETMMIQQLNPITAAPSPFPGVAAPNNNNINKGMTRSPSNSSTSTASSSASCSPPTSATTSRQLLSEAAVAMADGNLETAAAHLATLKGAVNPRGDAE

HRLLAMMVAALSSRIAPTASATSKHIADLCGSEHRTGSQLLHDISPCFRFALHAANIAIVEAVADRRAVHLVDFDISVPQHVALIQCLADRRVPGTSLKV

TAVTDPTSPFTESLTATLDAVGARLKKLAERAGIEYRFKIVNCRAAEIDSSKLGCEPGEALAVNLAFALSRVPDESVSPANPRDELLRRVRGLGPRVVTL

VEQELNSNTAPLATRFTDACAHYGAILESLDATAGRDSADRARAEEAVANKAANAVGREGADRLERCEVFGKWRARFGMAGFRPVALAAGIADQVKARVG

NSQPGFAVKPENGVLRLGWMGRVITVASAWR

>Bradi2g20760

MEQELRSGGPALPRPHSGSGGSICFSGAAALVDGPRIQQLLLHCAAALESKDVTLAQQAMWVLNNIVGSSQGDTPNSRLTSALLRGLVARACRTCVSPGS

SAEAAAGPGRSRAGGNGISATELAEYVDLTPWHRFGFTASNGAILRAAAGRPALHVVDLSVTHCMQWPTLIDALSKRAGGPPALRISVPRARPAVPPLLA

ASDAALLGPRLANFAKSRGVRLDFHVIDVKNAELASVLSDREALELRDGEALVVNCHSWLRHVAPGRRDGLLDAVRALEPCLVTVTDEDADLDSPSLASR

IAGCLEFHWILFDALDTCAPRDSTRRAEQEAALAQKIESVVAGDDGGGAAAERSECGARLSERMRRRGFDGVGFGEEVAAEVRRLLGEHANGWGVKTEED

MMVLTWKGHGAVFTTAWAPS

>Bradi4g43200

MFQQQLLQQQQEDVMSSSATSSPASTLYSPCPPGAGASWVQELSSDQCSVRLIGLLYQCAAEVAAGALDRANLCLEHIMQLASLDAPHALQRLAAVFADA

LARKLLNLVPGLSRALLTSSSSSSSPSPAGTSSGDAARRHMFDMLPFLKLAYLTTNHAILEAMEGERFVHVVDLSGPAANPAQWIALFHAFRGRSRDGQG

VFPHLRVTAVHESKEFLGAMAASLAREAEAFDIPFQFAGVESRLEDLDSAALRHCLRVRSGEALAVSVALQLHRLLAADDDGRCRRHPGGGLTPLQSILA

ARSSPRSPSSGGSSSFGELLERELNTRLQLSPDASSYNSPQYSSPTMSAFSSSGSGKPKLESFLSAIKALSPKVMVITEQEANHNGAAFHERFDEALNYY

ASLFDCLERASSGQGQHRSNSAAERARVVERAVLGEEIRSIVASEGRERKERHERAAQWAGRMEASGMERVGLSYGGAMEARKLLQSCGLGGGGYQVRHD

VQEQRFFFCWHDRPLYAVSAWRPAGAGPASAYHHRGPR

>Bradi1g22907

MQFTHAAVTAPPLHHHHHHHGHHVPNNAAHGLGLGLLLDVGTPRPWPGTTTPTTASSKISLGNLNSAGCMEQLLVHCASAIEANDATLTQQILWVLNNIA

PPTATPPSGSRRPSSARSCPAPPGRAPARPVTAAVAAAVAVEDVHRFTAVELAGFVDLTPWHRFGYMAANAAILEAAEGFPVVHVVDLSTTHCMQIPTLI

DLLASRAEGPPILRLTVADVSSNSSSSSSSSPPPVLDTSYEELGAKLVSFARSRNVSMDFRVVRTSPADAFASLLDTLRMQQLLVSDSSSCSEALVINCH

MLLHAVPDETAGSVMSTLTAGLLPQQQQPVSLRTMLLKSLRSLDPALVVIAEEDADFTACDVVGRLRAAFNFLWIPYDAVDTFLPKQGSAQRRWYEAEVG

WKVENVLAQEGVARVERQEGRARWGQRMRGAGFRAVGIGEEAAGEVRTMLNEHAAGWGMKREDDDLLLTWKGHNVVFASAWAPS

>Bradi1g78230

MPLGDLLLHGNQDQLASCAAATDLWDPASVLDHRPSPPASASSTLSSSSPLRAPAAGVAALAKNVHPPPAQPAAASPWPPPPGEDDWVHHLPPLDMGMGM

GMTWGDPHQSAAMPMPPVAGDHQNPPSQQDSTFLRWIIGGDDASSAAAMDHPDLDLDHMLPFPLHPGNDDTKPAATAPPFRSPLLHQQPHTHAAFHGGFP

SSASFDTQQQAAKRQHPMAGASSPKLLPPFVSGPGGFVSALKPKAEAGDDAAAVDQLAEAARLAEAGDGFGAREILARLNHRLPAAPSAGTPLLRSAFYF

KEALRVALDAATGEAASSSAAAASTPVDVLLKLGAYKAFSEVSPVLQFAHFTCVQAVLDELAGAACIHVLDFDIGVGEQWASLMQELAQRRPGAAALKVT

ALVSPASHHPLELQLIHENLSNFAAELGVPFQFTFFSLDAVDPAELLAIAGGDAIAVHLPVGSVHAAAVPSVLHLVRRLGAKLVISVDRSCDRGGGELPF

ATHLFQALQSCMFLLESLDAMGTDPDVASKIERFLIQPKIESCVTRRHRAVTAGDKMLPWRTMFASAGFVPVQISNFAEAQADSLLKKVPVRGFRVEKRG

GSLLLHWQRAELVSVSAWRC

>Bradi4g41880

MEALLSYPCSPLISFPAQHEESSSLLWPNQLVFPHENAHIYDANPPGDDRWQDHRFSTMDVVQEADDLLQDYFPSAKIIEGDNLAVAVQDDSLVMEDSSS

LDDLLMASATAVEAGDMVNASAILEKIDSIVLDGITCGRYGAVGSSSFDHLACYFASSLRTGIARARTECHPLALASRLPAYQMLQELSPFIKFAHFTAN

QAILEATLDEGGVHVVDLNVGDGVQWSSLMSDLLRHGGSKPFRLTAVGSTTVTATPGRWLSEFAESLGLPFRYDEVEDLHKLTEIICGGSSVILSCDATG

MSHTLAMDTSQTLPLLTGVIKVLQPKLVILIEDELSRLGRTPPPPLAGGASFVELFSEAWCHFAAVQESLASCFSGAGYKARLRLVEEEILGPSIEGAIT

ATWPPHGSMTGGTDAGPVASNGFPGSGYRTCEISGFNIVQAKMLAGLFSRGFGVLHDKKGRLALCWKDRPLTSVSVWSPV

>Bradi4g24867

METMSYPCSPLLSFPTHEENNFVLWSPQVALHEDATIHVDPSTHQLHDCGFLDTMALDYSNDWHRQDASDVGMFIDCDERILGQENGDLMAIQEELMEEN

SLTDLLLTGAEAVEAGDSRLATAVFSRLDGLLLGIPENAAVGSFDRLAYHFAQGLRSRLSSANTRCPPPEPLPSDRMSVQQIIQELSPFAKFAHFTANQA

ILDATKGDSDVHVVDLNIGEGVQWPSLMSDLASHGGRSFHLTAIITDADYSYDVHQASARRLSEFADSLKLPFQYNSLRIHSDEDLHDFSKSCNGPVIFS

CDTTSMTYKLLGKLRTILPGCVKMLRPKLMVIVEEELVGIGKEASVCNTSFVDFFFEALHHFTTVSESLASCFSGGNHGLCLRLVERDMVGPRIQDFVEH

YGPVTLEPNAPGVLERYGACEMSASNVAQARMLVGLFNRGFGVVHEKGRLVLCWKSRALTSVSVWAPI

>Bradi3g50930

MRAALLGAERNGGVDHHLAGRGSGKDLFWAAAGGLEPRSVLDCSRRLSPPNSTSTLSSSLGGGAADSTGVAAVSESSAGDAEATKWGAPGDHGGGGGGGG

GRKEDWGGGGCELPPIPGALDVGLVGGEGWDAMLGNAAAAAGQDQTFLNWIMGAAGDLELPVPPLPMHQQPLVDNAGFGFSAVDPLGFSLDHPLGGVSSD

LSSSGAMSHTTTGGGGSGGGSKASSAFGLFSPEAASLQPPPPPMLFHEGIDTKPPLLASQPPGLLNQYQHQPPPPAAAFFMPLSSFPDHNQRSQLHQPPP

KRHHSVPDDLYLTRNHLGTSAAGQGLAFSPLHGSVPFQLQPSPPPPLRGAMKTTAAEAAQQQLLDELAAAAKAAEAGNSTGAREILARLNHQLPPVGKPF

LRSASYLKEALLLALADGQHHGASRLTSPLDVALKLAAYKSFSDLSPMLQFANFTATQALLDEIACSAASCIHVIDFDLGVGGQWASFLQELAHRRGAGG

VALPLLKLTAFVSAASHHPLELHLTQDNLSQFAADLGIPFEFNAVSIDAFSPAELISPTGDEIVAVSLPVGCSARAPPLPVILRLVKQLGPKIVVAMDYG

ADRADLPFSQHFLHCFQSCMFLLDSLDAAGIDSDSACKIERFLIQPRVEDAVLGRRKADKSMAWRNVFAAAGFTPVPLSNLAEAQADCLLKRVQVRGFHV

EKRGVGLTLFWQRGELVSVSAWRC

>Bradi1g52240

MSSSSRGNLNNQDLDGGAAAKRLRGSSSSSKSPPEPTSVLYNRSPSPPTSSSHSSSAAPEPPPISAEDWDAVFLSAGPAMAVPASAAPHHSQAQEDSSFL

RWIMDTGYADGDADAFGFKSSTGAAFFDPSFLNPQSPAPQQQQEELFALPQQLPLPAVAQREEILEPQGAVDELLEAARLADAGDSTGAREILARLNHHG

LLPPSPPPPGHPPLLRAAALLRDALLLRLLPPGSGSGSVRPQPSPLDVALKLAAHKAMADASPAVQFASFTSTQAFLDAAGAGGVHLVDFDLGFGAHWPP

LMQELAHSSRRASPSAPAAALKLTALVSASGSPMELRLSQESLTRFAADLGIPFEFAALTFDPSSPMPGLSLSADETVAVHVTVGGVTSAVSPATLRLIK

QLRPAIVVCVDHGGGCDMPLPSHALNVLRSSAALLESLAAGGASPDVVTKVEQFVLRPRVERGLVPAGGDKLPPWRSLFASAGFAPLQLSNAAEAQAECL

LRRTAASHGFHAEKRQPGELALCWRRSELVSVSAWRC

>Bradi3g32890

MPFAGESHAGIQLLLLQQQQQGVLAGGGKVSSGGALWEPTSVLDLRHSPSPSPPVSSASTLSSSEVAALAGGDAKNVSPTPQQAWPGGGGGEDAAGVKEE

WAHQLAPLDNMGLAPCDDWDDAAAAPPSGPDSSFLRWIIAGGEDGGVLMDPPVLELDHATSMMSSPAAAAFGPPNLSSSFAPAMEDTKPQQPFGHGPGFL

LHHPQPHAAFFGSHPSFDSPPAATKRHHPMAAAPPPKLPPYPSHHLAPASGGGGFVLKQPKAAAASQDEAAAMAAVEQLAEAAKLAEAGDAHGAREILAR

LNYRLPAAPAAGNTPPLLRSAFYFKEALRLALSTTPAGGEAYYSPTPPASTPYDVVLKLGAYKAFSEASPVLPFAHLTCVQAVLDELGDRGCVHVLDFDI

GMGEQWASLMQELASSSQRRTSAAAALKVTALVSPATHHPLELQLIHENLSSFAADLGVLFQFSAFNVETLDPADLVSIVAGAGDEALAVHLPVGAAHAA

STPAVLRLVKRLGAKVVVAVDRGGDRAELPFAAHLFQAFQSAVFLLESIDAVLGSADPDTAGRVERFLVQPAVEQCVVARHRDRASPGMMPWRAVFAAAG

FAPAQASSFAESQAESLLHKVPVRGFRVERRAPGSLCLYWQRAELVSVSAWRC

>Bradi1g23060

MDTLFRLVSLQATTEQQQQQQQQSASYNSRSTTSSGSRSSSHHTTASYSYYNSGSGSGGQPQQQYYYSQPQQSYYLEPYHEECGGGNQLYMDEDFSSSSS

SRHFGGHHGSHGGQQQQPSSTPPLSTTSSTAAGHALFEPAELSFPPDLNLDFSSPASSSGGGIAAASASSPAVIGGAGGGRWASQLLMECARAVASRDST

RVQQLMWMLNELASPYGDVEQKLASYFLQALFARLTASGPRTLRTLAAATDRNTSFDSTRRVALKFQELSPWSSFGHVAANGAILESFLEAAAAAPSSEP

QRFHILDLSNTFCTQWPTLLEALATRSPDDTPHLSITTVHVSSSSAASSPAVQRVMREIGQRMEKFARLMGVPFRFRAVHHSGDLAELDLDALDVREGGA

TTGIAVNCVNSLRGVGARRRGEFAALLRRLGPRVVTVVEEEADFVADSDHHRSADQDAETDQAAFLKVFGEGLRFFSAYMDSLEESFPKTSNERLALERG

AGRAIVDLVSCPASESMERRETAAAWARRLRCAGFSPVAFSDDVADDVRSLLRRYREGWSMRDAGAEDSAAAGAGVFLQWKEQPLVWASAWRP

>Bradi4g09197

MAATDGFMRLIRERDNRGAPAFHLAAHDAEDEDEPPMSPSLFLDLPPTPPRLLAADAPGDIDLDFISRMLMEEDIDDRFFYQYPDHPAILSAHRAFAQII

SDDRSAAATTSSDYCSSATTGAKSTPTTSSSDSPDALSASPTAACWPRESPVDLSVDSHAALPKGSGDDSFGAVDRNGTTSTAEKEKENGSKPATTATFL

AGGSDDGAFTASAFFNGGGAMDDMLSLAFRKGMEEASKFLPSATNNSLLVDPDSFSGGDQARKEEADGLGTLMFGSSNNGRSRKHRHSDDEAEAEAEAGR

SRKVMAPEPEEVGTDARQMFDEIMLHGYDSCMQGIEDLRIAMGSEAERNIRNGTGGKAAAVARRGKRRGAARSEPEVDLRTMLIHCAQAVATGDHRSSAD

LLSQVKQHCSPKGDATQRLAYCFAEGLEARLAGTGSQVYQSLMAKRTSVVEYLRAYKLYMAASSFKKVNMAFVGKTIVDAMAPGKGRDRLHIVDYNVQYG

FQWPGLLQWLSIREGGPPEVRITGIDLPQPGFRPAFQIEETGRRLTDCAREFGVPFKFHGIAAKWETVRAEDINIDPDEFLVVTSQAGFGNLLDESVVMD

RQDIPSPRDMVLNNIGKMRPDVFIDCVVNGTYGAPFFVTRFREALYYYSAQFDMLDATIPRDNDERLLIERDIFGRCALNVVACEGADRVERPETYKQWQ

VRGHRAGMRQLPLCPEVVKVVRDKVKNYYHKDFVIDVDNRWLLQGWKGRVLYAMSTWVAADDDKSRF

>Bradi1g60140

MDYHEPDEQELTTAVTAIADGGGLDWLEDSISLLSADGDLSGSYGWWCDPPLLPAQQDDIGIVVAQTLPPPAALTTTGSPPTSIASPAPASSKKRKSSPA

SGHSGGGSQRRRAEQERERPAGGGGKKKGGGSDCREARWAEQLLSPCAVAVEARNLSRVQHLFYVLGELASFSSGDANHRLGRLRPARALPPPPRRGWPR

RRSRRARPGLGRRVPVVVRGRGARVFRASLIRFHEVSPWFALPNALANAAIAQQLLQAPGRPLSLVDVGVSHGVQWPTLLESLTRLPAGRAPPSVRLTVV

SAAAATRAAPFSASPPGYDFAPHLLRYAKSINLDLHIARAGDLSAQLGEEEALVVVCLQFRLGHVAPEERTEVLRKVRDLKPDLVVLSELDVVVGGGSAG

AAGEFSARLELLWRFLESTSAAFKGRDGEDRRLMEAEAGAPAGMITAPASGEGREAWRDRMRAAGFQEAAFGGEAVESARSLLRKYDSGWEMAGAASGAA

AVALRWKGQPVSFCSLWRPAPPP

>Glyma05g03490

MLAGCSSSTLLSPRHRLRSEASAQFQACHFQLPSSMSTQRLDLPCTTTTFTRNNKDTTSSRSVLSVDQKPIEAKTSTCSLKQHIRLPPLAITAAPSPLVE

DSIIKDNTNNNKSLKRLAAEHQDDSFTNNIAKRKKKSSSTECDWFQPDVVGTTLGGFNNNNNNNNTSLASFSSEEDRVCFVPSEVVSHSAPFPLNPWLES

CVTKITNFGEGSHRPHHHHHSDHASGSVSNASSESQSLRLNDNVSEHEVGNGSGNPYYHHEVDTGEEDNHHGFELVSLLTGCVDAIGSRNVTAINHFIAK

LGDLASPKGTTSISRICAYFTEALAIRVTRLWPHVFHITTTTTSRDMVEDDESATAMRLLNQVTPIPRFLHFTSNEMLLRAFEGKDRVHIIDFDIKQGLQ

WSGLFQSLASRSNPPTHVRITGIGESKQDLNETGERLAGFAEALNLPFEFHPVVDRLEDVRLWMLHVKEHETVAVNCVLQLHKTLYDGSGGALRDFLGLI

RSTNPSVVVVAEQEAEHNENRLEGRVCNSLKYYSALFDSIDESGLPQESAVRVKIEEMYAKEIRNIVACEGRERVERHESFGNWRRMMVEQGGFRCMGVT

ERELSQSQMLLKMYSCESYSVKKQEKEGATGVTLSWLEQPLYTVSAWGPVDAAAGTSSSFSQPS

>Glyma17g14030

MLAGCSSSTLLSPRHRLRSEAPAQFQACHFQLPSSMSTQRLDLPSCTTATFTRNNKDHHHHQPLRPVGLSVDQKHIEAKTSTCSLKQHIRLPPLAITASA

TPLVEESSIINDNNNKSLKKRLAAEHHDDSFAKRKKSSSTTECDWFQPDVVETTTLGGFNNNNTSLVSFSSEEQERVCFLPSEVVSHSAPFPLNPWLESC

VTKITNFGEGSHRHPHHHHHPHHHNDHASGSVSNASSESQSLRLNDNVSEHEVGNGSGNPYYHHRKVEAGEEDDHHGFELVSLLTGCVDAIGSRNVTAIN

HFIAKLGDLASPKGTTSISRICAYFTEALAIRVTRLWPHVFHIAAATTSRDMVEDDESATALRLLNQVTPIPKFLHFTSNEMLLRAFEGKDRVHIIDFDI

KQGLQWPSLFQSLASRSNPPIHVRITGIGESKQDLNETGERLAGFAEVLNLPFEFHPVVDRLEDVRLWMLHVKEHETVAVNCVSQLHKTLHDGSGGALRD

FLGLIRSTKPSVVVVAEQEAEHNHTRLEARVCNSLKYYSALFDSIEESGLPIESAVRVKIEEMYGKEIRNIIACEGRERVERHESFGNWRRMMVEQGGFR

CMSVTERELSQSQMLLKMYSCESYSVKKQEKEGATGVTLSWLEQPLYTVSAWGPVDAAAGTSSSFSHPS

>Glyma16g05751

MMNSLCGSSVSLKSENNNSRTKVQQPTTPSNDSVMQSKKNNATQSSGDLDQTSLTPPSLNLPSLKFDLDGDVEVQSPDSSMWESFFADHNLDGDFMISSP

VRTPQASSYNCNYNYAQGMQGQSLSGCSPPRFSSSQIGAFNSNKGKGLSPLHRVFNSPNNQYMQHVENLSLPAIEFLEDYQLGYSSSTTNKMCCSDHNNI

ASSSECFDLSMSHQIPSSMLDGLALHNSSSRYHRGSVVVDEESSVHGGGAGGFSQLSEESDIYHQMGSMASASLSQALQQEHYQEKQQAQQLQQQQQQQQ

QHQQENLMVPIPIGVEQEQDSGLQLVHLLLACAEAVAKEEYMLARRYLHHLNRVVTPLGDSMQRVAACFTDSLSVRLNSTLTPKPTTPSKPLTPSNSLEV

LKIYQIVYQACPYVKFAHFTANQAIFEAFETEERVHVIDLDILQGYQWPAFMQALAARPAGAPFLRITGVGPSIDTVRETGRCLTELAHSLRIPFEFHAV

GEQLEDLKPHMLNRRVGEALAVNAVNRLHRVPGNHLGNLLTMLRDQAPSIVTLVEQEASHNGPYFLGRFLEALHYYSAIFDSLDATFPAESAQRAKVEQY

IFAPEIRNIVACEGPERFERHERLEKWRKMMEGKGFKGVVLSPNAVTQSKILLGLYSCEGYRLTEDKGCLLLGWQDRAIVAASAWRC

>Glyma19g26735

MMNSLCGSSLSLKSENNNSRTKLQQPTSSNDSVLQSKKNNATQSSGDLDQTSLTPPSLNLPSLKFDLDGDVEVQSPDSSMWESFFADHIDGDFMISSPVR

TPQAASSYNCNYNYAQGMQGQSLSGCSPPRFSSSQIGAFNSSSSLNKGKGLSPLHRVFNSPNNQYMQHVENLSLPAIEEFLEDYQTKVSSSDHNITSSSE

CFDLSSHQIPSSILDGLALHNSSRYHRGSVVDEESTVHGGGGGGGGSSQLSQESDIYHQMGSMASASLSQALQQERYQEKQQKQQAQQLQQQQRQHQQEN

LMVPIPIEIEQEQDSGLQLVHLLLACAEAVAKEEYMLARRYLHHLNRVVTPLGDSMQRVAVCFTDSLSARLNSTLTPKPATPSKPLTPSNSLEVLKIYQI

VYQACPYVKFAHFTANQAIFEAVEIEERVHVIDLDILQGYQWPAFMQALAARPAGAPFLRITGVGPLLDAVRETGRCLTELAHSLRIPFEFHAVGEQLED

LKPHMLNRRVGEALAVNAVNHLHRVPGNHLGNLLTMLRDQAPSIVTLVEQEASHNGPYFLGRFLEALHYYSAIFDSLDATFPAESAQRAKVEQYIFAPEI

RNIVACEGAERFERHERLEKWRKIMEGKGFKGVALSPNAVTQSKILLGLYSCEGYRLTEDKGCLLLGWQDRAIIAASAWRC

>Glyma10g33380

MKMISSASNENKTVVDMDDHLAGLGYKVRSSELCQVAANMERLENVISSTDLSQLASDTTLYDPSNIGLGSWVDTLLSEFDQTASLPLQYDFATDPNHNK

QLALVTTVEEDSGIRLVHMLMTCADSVQRGDFSFAGSLIENMQGLLAHVNTNCGIGKVAGYFIDALRRRISNTLPTSSSTYENDVLYHNYYEACPYLKFA

HFTANQAILEAFNGHDCVHVIDFNLMQGLQWPALIQALALRPGGPPLLRLTGVGPPSAENRDNLREIGLRLAELARSVNVRFAFRGVAAWRLEDVKPWML

QVSLNEAVAVNSIMQLHRVTAVDAAVEEVLSWIRSLNPKIVTVVEQEANHNGEGFLERFTEALHYYSTVFDSLDACPVEPDKAALAEMYLQREICNVVCC

EGPARLERHEPLAKWRDRLGKAGFRPLHLGFNAYKQASMLLTLFSAEGFCVQENQGSLTLGWHSRPLIAASAWQAAPLRDDETLRFGH

>Glyma08g10140

MKREREQLGSIAGTSSCGYSSGKSNLWEEEGGMDELLAVVGYKVRSSDMAEVAQKLERLEEAMGNVQDDLPEISNDVVHYNPSDISNWLETMLSNFDPLP

SEEPEKDSASSDYDLKAIPGKAIYGASDALPNPKRVKADESRRAVVVVDSQENGIRLVHSLMACAEAVENNNLAVAEALVKQIGFLAVSQVGAMRKVAIY

FAEALARRIYRVFPLQHSLSDSLQIHFYETCPYLKFAHFTANQVILEAFQGKNRVHVIDFGINQGMQWPALMQALAVRTGGPPVFRLTGIGPPAADNSDH

LQEVGWKLAQLAEEINVQFEYRGFVANSLADLDASMLDLREGEAVAVNSVFEFHKLLARPGAVEKVLSVVRQIRPEIVTVVEQEANHNRLSFVDRFTESL

HYYSTLFDSLEGSPVNPNDKAMSEVYLGKQICNVVACEGMDRVERHETLNQWRNRFVSTGFSSVHLGSNAYKQASMLLALFAGGDGYRVEENNGCLMLGW

HTRPLIATSAWQLAATR

>Glyma06g23940

MASSSSNGSSSGSKSWDIDGDLAGFGYKVRSSELQHVAENMERLENVMDIVNSSTNNNISQLASDTVFYNPSDIGSWVDTLLSEFDQTASLPYDFSDFLD

LDTDQNQNHKPTLVTMEEDSGIRLVHTLMTCADSVQRGDLAFAGSLIENMQGLLAHVNTNIGIGKVAGYFIDALRRRILGQGVFQTLSSSSYPYEDNVLY

HHYYEACPYLKFAHFTANQAILEAFNGHDCVHVIDFNLMQGLQWPALIQALALRPGGPPLLRLTGIGPPSSDNRDTLREIGLRLAELARSVNVRFAFRGV

AAWRLEDVKPWMLQVNPNEAVAVNSIMQLHRLLASDSDPIGSGIETVLGWIRSLNPKIISVVEQEANHNQDRFLERFTEALHYYSTVFDSLEACPVEPDK

ALAEMYLQREICNVVSSEGPARVERHEPLAKWRERLEKAGFKPLHLGSNAYKQASMLLTLFSAEGYSVEENQGCLTLGWHSRPLIAASAWQAAPMQDRET

LRFEQ

>Glyma04g21340

MASSSSNGSSSGSKSWDIDGDLAGFGYKVRSSELQHVAENMERLENVMDIVNSSTNNNISQLASDTIFYNPSDIGSWIDTLLSEFDQTASLPYDFSELPD

LDTDQIQNLKPTLVTMEEDSGIRLVHTLMTCADSVQHGDLPFAGSLIENMQGLLAHVNTNIGIGKVAGYFIDALRRRIFAQGVFLTSCSYPIEDDVLYHH

YYEACPYLKFAHFTANQAILEAFNGHDCVHVIDFNLMQGLQWPALIQALALRPGGPPLLRLTGIGLPSSDNRDTLREIGLRLAELARSVNVRFAFRGVAA

WRLEDVKPWMLQVNPNEAVAVNSIMQLHRLLASDSDPAGSGIETVLGWIRSLNPKIISVVEQEANHNEDMFLERFTEALHYYSTVFDSLEACPVEPDKAL

AEMYLQREICNVVCCEGPARVERHEPLDKWRKRLGKAGFKPLHLGSNAYKQASMLLTLFSAEGYCVEENQGCLTLGWHSRPLIAASAWHAAPVQDRETLR

FEQ

>Glyma05g27190

MKRERQQLGSNAGTSSCGYSSGKSNLWEEEGGMDELLAVVGYKVRSSDMAEVAQKLERLEEAMGNVQDDLTDLSNDAVHYNPSDISNWLQTMLSNFDPLP

SEEPEKDSASSDYDLKAIPGKAIYGGGSDALPNPKRVRTDESTRAVVVVDLQENGIRLVHSLMACAEAVENNNLAVAEALVKQIGFLALSQVGAMRKVAT

YFAEALARRIYRVFPQQHSLSDSLQIHFYETCPYLKFAHFTANQAILEAFQGKNRVHVIDFGINQGMQWPALMQALALRNDGPPVFRLTGIGPPAADNSD

HLQEVGWKLAQLAERIHVQFEYRGFVANSLADLDASMLDLREDESVAVNSVFEFHKLLARPGAVEKVLSVVRQIRPEILTVVEQEANHNGLSFVDRFTES

LHYYSTLFDSLEGSPVNPNDKAMSEVYLGKQICNVVACEGMDRVERHETLNQWRNRFGSTGFSPVHLGSNAYKQASMLLSLFGGGDGYRVEENNGCLMLG

WHTRPLIATSVWQLATKSVVAAH

>Glyma20g34260

MERLENVISDTILYDPSNIGLGSLVDTLLSDLDQTMSLPSHYHLSSDLPDFATTVEEHSGIRLIHTLMTCADSLQRGHFSFAASLIQNMQGLLAHVNTNC

GIGKVAACFIDALRRRISNKFPASSAYENDVLYHNYYEACPYLKFAHFTANQAILEAFNGHDCVHVIDFNLMQGLQWPALIQALALRPGGPPLLRLTGIG

PPSAENRDNLREIGLRLAELARSVNVRFAFRGVAAWRLEDVKPWMLQVSPNEAVAVNSIMQLHRLTAVKSAVEEVLGWIRILNPKIVTVVEQEANHNGEG

FLERFTEALHYYSSVFDSLDACPVEPDKAALAEMYLQREICNVVCCEGPARLERHEPLAKWRDRLGKAGFRALHLGFNAYKQASMLLTLFSAEGFCVQEN

QGSLTLGWHSRPLIAASAWQAAPLGDDETLRFEH

>Glyma11g10220

MLQSLVPRSPRTSNPNAMKTKRAVDAGGDSPADEPSFKRTNFSGEKTTAEAEEEQAFDPEPHGGDSTGLKLLGLLLQCAECVAMDNLDFANDLLPEIAEL

SSPYGTSPERVGAYFAQALQARVVSSCIGSYSPLTAKSVTLTQSQKIFNAFQSYNSVSPLVKFSHFTANQAIFQALDGEDRVHIIDLDIMQGLQWPGLFH

ILASRSKKIRSVRITGFGSSSELLDSTGRRLADFASSLGLPFEFFPVEGKIGSVTELSQLGVRPNEAIVVHWMHHCLYDITGSDLGTLRLLTQLRPKLIT

TVEQDLSHAGSFLARFVEALHYYSALFDALGDGLGADSLERHTVEQHLLGCEIRNIVAVGGPKRTGEVKLERWGDELKRAGFGPVSLRGNPAAQASLLLG

MFPWRGYTLVEENGSLKLGWKDLSLLIASAWQPSDLMITYPD

>Glyma18g45220

MLLPSSTTTTINSSGVAPNYITHHQHHHFQGLVESQDHQQNPVPAVCGFSGLPLFPSQSQRNRDSNNTPTTNNIRNSGGNIVDVVASSSSSSSMDDTSAA

AATSGWIDGILKDLIHSSNSVSIPQLISNVREIIYPCNPNLAMVLEYRLRLLLTESTTQNKRGTEGVPLPPSVSSVKLTSNRVVDGIVPNLHFTDASGGA

VVVNQHMLSNWGVPQITHHHDNNTNTNTNNSNNPSVSLVTLPSPTPPLPPPHYSPPQEKHPQEEDLAATSTAEVALSRKKKEELREQKKKDEEGLHLLTL

LLQCAEAVSSENLEDANKMLLEISQLSTPFGTSAQRVAAYFSEAISARLVSSCLGIYATLPHTHQSHKVASAFQVFNGISPFVKFSHFTANQAIQEAFER

EERVHIIDLDIMQGLQWPGLFHILASRPGGAPYVRLTGLGTSMEALEATGKRLSDFANKLGLPFEFFPVAEKVGNLDPERLNVCKTEAVAVHWLQHSLYD

VTGSDTNTLWLLQRLAPKVVTVVEQDLSNTGSFLGRFVEAIHYYSALFDSLGSSYGEESEERHVVEQQLLSREIRNVLAVGGPSRTGEPKFHNWREKLQQ

CGFRGISLAGNAATQASLLLGMFPSEGYTLVEDNGILKLGWKDLCLLTASAWRPPFHSAITHHN

>Glyma09g40620

MAACALFSGGGNITEDGNVNGSGSANSTPLTSASNSSNMSNEEQQQLHAGGIMPQPYCERKMMRKRMASEMEVNVHATPPHNSSSTTSDYPRFPRRSNTN

TNMLEKGSPTTTSSTTTLAAGTCNNNNNNNNNNNSHHYNNSNNSNSGNISSRDNVAIPNYPTVTVTTNYSTMLLPSSLNSSGVAPNYNSTHQHHHFQGLV

ESQDQQNSVPAVCGFSGLPLFPSQSQRNRDNIRNSGGNIVDVVASSSPSPSMDDTSGAATTSGWIDGILKDLIHSSNSVSIPQLISNVREIIYPCNPNLA

MVLEYRLRLLLTESTTPQHKRGTEGVPLLPSVSSVKLMNNRVVDGIAPNLHFTDASGGAVVVNQHMLSNWGVPQITPHHDNTNTNTNTNNNNNPSVSLVT

LPSPAPPPHYSPPEEKNPQEEDLAAATTTAHEVALSRKKKEELREQKKKDEEGLHLLTLLLQCAEAVSAENLEDANKMLLEISQLSTPFGTSAQRVAAYF

SEAISARLVSSCLGIYATLPHTHQSHKVASAFQVFNGISPFVKFSHFTANQAIQEAFEREERVHIIDLDIMQGLQWPGLFHILASRPGGAPYVRLTGLGT

SMEALEATGKRLSDFANKLCLPFEFFPVAEKVGNLDPERLNVSKTEAVAVHWLQHSLYDVTGSDTNTLWLLQRLAPKVVTVVEQDLSNTGSFLGRFVEAI

HYYSALFDSLGSSYGEESEERHVVEQQLLSREIRNVLAVGGPSRTGEPKFHNWREKLQQCGFRGISLAGNAATQASLLLGMFPSEGYTLVEDNGILKLGW

KDLCLLTASAWRPPFHGAITHHN

>Glyma13g18680

MMNVGFEVIHANYNPNMIHPHMHETWDHYSNTITSLPFSAPTPSNPYPKPATENNHCLNLGQNELCDWMEEHISDITKHFVEDLPETTTSDNLLSNNPTG

VVSHHNLGVPSLLSPNFTQRKPSCFRPQFESFTNDPPNFNLHIQTNTSTLDQNKHNVYDQGLNLITLLMECAVAISVDNLGEAHRMLLELTQMASPYKAS

CAERVVAYFAKAMTSRVMNSWLGVCSPLVDHKSINSAFQVFNNISPFIKFAHFTSNQAILEAVSHCDSIHIIDLDIMQGLQWPAFFHILATRMEGKPKVT

MTGLGASMELLVETGKQLTNFARRLGLSLKFHPIATKFGEVIDVSMLHVKPGEAVAVHWLQHSLYDATGPDWKTLRLLEELEPRIITLVEQDVNHGGSFL

DRFVASLHYYSTLFDSLGAYLHNDDSNRHRVEHGLLSREINNVLAIGGPKRSGEDNFRQWRSELARHCFVKQVPLSDNSMAQAQLILNMFSPAYGYSLAQ

VEGTLRLGWKDTSLYTASAWTCCNSS

>Glyma12g02530

MLQSLVPRSPRTSNPNAMKTKRPVDASDLSPADEPSFKRTNFSADRTAAEAVEEQAFDPEPHGGESTGLKLLGLLLQCAECIAMDNLDFANDLLPEIAEL

SSPYGTSPERVGAYFAQALQARVLSSCIGSYSPLTAKSVALTQSQRIFNAFQSYNSVSPLVKFSHFTANQAIFQSLDGEDSVHIIDLDIMQGLQWPGLFH

ILASRSKKIRSVRITGFGSSSELLDSTGRRLADFASSLGLPFEFFPVEGKIGSVTELSQLGVRPNEAIVVHWMHHCLYDITGSDLGTLRLLTQLRPKLIT

TVEQDLSHAGSFLARFVEALHYYSALFDALGDGLGEDSLERHTVEQHLLGCEIRNIVAVGGPKRTGEVKVERWGEELKRAGFGPVWLRGNPAAQANLLLG

MFPWRGYTLLQENASLKLAWKDFSLLIASAWQPQYKNICFIHSSH

>Glyma11g33720

MKRDHKDSCGGGGAAGGTVKGECSSMQSNGKAKMWEEEQQQQQQQQQQQQQQGMDELLAALGYKVRASDMADVAQKLEQLEMVMGCAQEDGISHLASDTV

HYDPTDLYSWVQSMLTELNPEPNNNLDPSSFLIDNNNNIINSTAPVFNDDSEYDLRAIPGIAAYPPPLPQDNHLDEIETANNINKRLKPSPAESADSAAS

EPTRHVVLVDHQEAGVRLVHTLLACAEAVQQENLKLADALVKHVGILAASQAGAMRKVASYFAQALARRIYGIFPEETLDSSFSDVLHMHFYESCPYLKF

AHFTANQAILEAFATAGKVHVIDFGLKQGMQWPALMQALALRPGGPPTFRLTGIGPPQPDNTDALQQVGLKLAQLAQIIGVQFEFRGFVCNSLADLDPNM

LEIRPGEAVAVNSVFELHRMLARSGSVDKVLDTVKKINPQIVTIVEQEANHNGPGFLDRFTEALHYYSSLFDSLEGSSSSSTGLGSPSQDLLMSELYLGR

QICNVVAYEGPDRVERHETLTQWRGRLDSAGFDPVHLGSNAFKQASMLLALFAGGDGYRVEENNGCLMLGWHTRPLIATSAWKLPSSSESSGLTQ

>Glyma18g04500

MKRDHRDSCGGGGGGSVKGECSSMPSNGKANMWEEQQQQQQGMDELLAALGYKVRASDMADVAQKLEQLEMVMGCAQEEGISHLASDTVHYDPTDLYSWV

QTMLTELNPEPNNNNNSLLGPSSLLIDNNTAPVFNDDSEYDLRAIPGIAAYPPPPPQDNNNNNNNLDEIETANNINKRLKPSPVESADSASEPTRTVLLV

DHQEAGVRLVHTLLACAEAVQQENLKLADALVKHVGILAASQAGAMRKVASYFAQALARRIYGIFPEETLDSSFSDVLHMHFYESCPYLKFAHFTANQAI

LEAFATAGRVHVIDFGLRQGMQWPALMQALALRPGGPPTFRLTGIGPPQPDNTDALQQVGWKLAQLAQNIGVQFEFRGFVCNSLADLDPKMLEIRPGEAV

AVNSVFELHRMLARPGSVDKVLDTVKKIKPKIVTIVEQEANHNGPGFLDRFTEALHYYSSLFDSLEGSSSSTGLGSPNQDLLMSELYLGRQICNVVANEG

ADRVERHETLSQWRGRLDSAGFDPVHLGSNAFKQASMLLALFAGGDGYRVEENNGCLMLGWHTRPLIATSAWKLPSPNDLHCKL

>Glyma12g34420

MDSQQLFSFGGVTSAGLPYMSSFPTVPSLPNSLLRSLKYDTGNSPNSPFSTYFDSDTLSALSDGQEQYSPGEILSGVSSSLETNHYMYNRSISTLDSFPL

YSDRNSLLQNASSNQKIQHALLELETALMAPDDDQVTTPNTLAERHRSWNNENHVSQHNTQAQPSYATGNRQSSEVVHVEKRQKLMEEEATLEAFPPNNL

KQLLIACAKALSENNMNDFDQLVGRAKDAVSINGEPIQRLGAYMVEGLVARTQASGNSIYHALRCKEPEGDELLTYMQLLFEICPYLKFGYMAANGAIAE

ACRNEDRIHIIDFQIAQGTQWMTLLQALAARPGGAPHVRITGIDDPVSKYARGDGPEVVGKRLALMSEKFGIPVEFHGVPVFAPDVTREMLDIRPGEALA

VNFPLQLHHTADESVHVSNPRDGLLRLVRSLSPKVTTLVEQESNTNTTPFFNRFIETLDYYLAIFESIDVTLPRDSKERINVEQHCLARDIVNIIACEGK

ERVERHELFGKWKSRLKMAGFQQCPLSSYVNSVIRSLLRCYSEHYTLVEKDGAMLLGWKDRNLISASAWHC

>Glyma10g04421

MMKVGFEVVHANYNSNMIRPHMHETWDDHYSNNITTSLPFSSPTTPSNPYPKPATENNHCLNMGQNNELCDWMEEQVSDFTKHLVEDFPETTTTSDNLLS

NNPTRVNIASHHNLNVPSLLSPKFSQRKPSCLFRPQFESFTNNDPPNFNLHIQTNTSTLDQSQHNVVYDQGLSLITLLMECAVAISVDNLGEAHRMLLEL

TQVSSPYKASCAERVVAYFAKAMTSRVMNSWLGVCSPLVDHKSINSSFQVFNNISPFIKFAHFTSNQAILEAVSHCDSIHIIDLDIMQGLQWPAFFHILA

TRMEGKPQVTMTGFGASMELLVETGKQLTNFARRLGMSLKFLPIATKIGEVIDVSTLHVKPGEAVAVHWLQHSLYDATGPDWKTLRLLEELEPRIITLVE

QDVNHGGGGSFLDRFVASLHYYSTLFDSLGAYLHNDDENRHRVEHGLLSREINNVLGIGGPKRSEDKFRQWRNELARHCFVKQVPMSANSMAQAQLILNM

FSPAYGYSLAQVEGTLRLGWKDTSLYTASAWTCSNSS

>Glyma13g36120

MPSSFSMDSQQLFSFELPYMSSLPTVPSLLGSLKYDTGNSPNSPFSTYFDSDTLSALSDGQEQYSPGEILSGVSPSCNSSLETNHYMYRSVSTLDSFPLY

SDRNSLLQTMSSNQKIQHALLELETALMAPDDDQVNTPNTLAESSRPMASGQRSRSWSNENHVSQYTQTQPSYATANMQSSEVVHVEKRQKLMEEATLQD

FPPNNLKQLLIACAKALSENNTKDFDQLVGKAKDAVSINGEPIQRLGAYMVEGLVARMQASGNSIYHALRCREPEGEELLTYMQLLFEICPYLKFGYMAA

NGAIAQACRNEDHIHIIDFQIAQGTQWMTLLQALAARPGGAPHVRITGIDDPVSKYARGDGLEVVGKRLALMSEKFGIPVEFHGVPVFAPNVTREMLDIR

PGEALAVNFPLQLHHTADESVHVSNPRDGLLRLVRSLSPKVTTLVEQESNTNTTPFFNRFIETLDYYLAIFESIDVTLPRDSKERINVEQHCLARDIVNI

IACEGKERVERHELFGKWKSRLTMAGFRQCPLSSYVNSVIRSLLMCYSEHYTLVEKDGAMLLGWKDRNLISASAWHC

>Glyma01g43620

MFQEEGLSSVTSSPLQFFSMMSLSVSPSLGSPYQMKCEQRGLVLIHLLLAGANFVATGDLQNANLTLEQISQHASLDGDTMQRIASYFSEALADRILKTW

PGIHRALNSSRITMVSDEILVQKLFFELLPFLKFSYILTNQAIVEAMEGEKMVHIVDLYGAGPAQWISLLQVLSARPEGPPHLRITGVHHKKEVLDQMAH

KLTEEAEKLDIPFQFNPVLSKLENLDFDKLRVKTGEALAISSILQLHSLLALDEDASRRKSPLLSKNSNAIHLQKGLLMNHNTLGDLLDGYSPSPDSASS

SPAASSSALMNSESFLNALWGLSPKVMVVTEQDFNHNCLTMMERLAEALFSYAAYFDCLESTVSRASMDRLKLEKMLFGEEIKNIIACEGCERKERHEKM

DRWIQRLDLSGFANVPISYYGMLQGRRFLQTYGCEGYKMREECGRVMICWQERSLFSITAWRPRK

>Glyma06g41500

MDLNQFSSFNVISSAGFPCASSCTPISPISNRLLGSLKLDIGNSPNSALSTHFDSDTVSSALSDSQEQHNCGEIHSGVNSCNSLQESNHYLHRPVSSVDH

LEDGLHLSTRSFFPQDASYDHETRHALLELETSLMAPDDEDQVTTSSTSLGDSSRPTASDQRNRSWSHEGQSSDVAYVEKRHKSMEEALLQGFPSSNLKQ

LLIVCAKALSENNMKGFDQLIEKARSAVSITGEPIQRLGAYLVEGLVARKEASGNNIYHALRCREPEGKDLLSYMQLLYEICPYLKFGYMAANGAIAEAC

RNEDLIHIIDFQIGQGTQWMTLLQALAARPGGAPHVRITGIDDQLSKYVRGDGLEAVGKRLAAISQTFNIPVEFHGVPVLAPDVTKDMLDVRPGEALAVN

FPLQLHHTADESVDMSNPRDGLLRLVKSLSPKVTTLVEQESNTNTTPFFNRFIETLDYYLAIFESIDVSLPRKSKERVNVEQHCLARDIVNIIACEGKER

VERHELLGKWKSRLTMAGFRQYPLSSYVNSVIRSLLRCYSEHYNLVEKDGAMLLGWKDRNLISASAWH

>Glyma12g16750

MDLNQFLNFNVISSEGFPCTSSCTPISPISNQLLGSLKLDIGNSPNSALSSHFDSDTLSSALSDSQELHTSGEIHSGVNSCKSLQESNHYLHRPVSFVDH

LQDGLHLSTRSFLPQDASYDHETRHALLELETALMAPDGEDQITTSSTSLGVGSRPTTSCQRNRSWSSEGQSSDVAHVEKHHKSVEEESLQGFPSCNLKQ

LLIVCAKALSENNMQHFDQLIEKARSAVSITGEPIQRLGAYLVEGLVARKEASGNNIYHALRCREPEGKDLLSYMQLLYEICPYLKFGYMAANGAIAEAC

RNEDQIHIIDFQIGQGTQWVTLLQALAARPGGAPHVRITGIDDPLSKYVRGDGLEAVGKRLAAISQTFNIRVEFHGVPVLAPDVTKDVLDVRPGEALAVN

FPLQLHHTADESVDMSNPRDGLLRLVKSLSPKVTTLVEQESNTNTTPFFNRFIETLDYYLAMFESIDVSLPRKSKVQINMEQHCLARDIVNIIACEGKER

VERHELLGKWKSRLTMAGFRQYPLSSYMNSVIRSLLRCYSKHYNLVEKDGAMLLGWKDRNLISTSAWY

>Glyma15g28410

MKGINRWLSFYMDESVNHDFAIRRYCPARMEQEQGGDKCQDQEALVFYDTELCSSDATSSTPCLASSEVDDFVDSFINMDQYEYVNEDQGFQEKHRSFDH

FVVNDEDEADAYSIVNGVFEYVPTTLEDSELEIYEDVTTAMLEEEVAMNGSFCAIPEFVVPCTQEANLGVDQGLDLVHMLLACAEAVGCRDNQQAELLLS

RIWALASPSGDSLQRVSYCFAKGLKCRLSLLPHNVIANATLSSMDVPFITRENKLEAFQLLYQTTPYIAFGFMAANEAICQASQGKSSIHIVDLGMEHTL

QWSSLIRALSSRPEGPPTLRITGLTGNEENSKLQASMNVLVEEASSLGMHLEFHIISEHLTPCLLTMEKLNLRKEEALCVNSILQLHKYVKESRGYLKEI

LLSIKKLGPTALTVVEQDTNHNGPFFLGRFLESLHYYSAIFDSLEASMTRNSQHRMKIERLHFAEEIQNVVAYEGPDRIERHERVDQWRRQLGRAGFQVM

PLKCTSQVRMMLSVYDCDGYTLSYEKGNLLLGWKGRPVMMASAWQVASV

>Glyma04g28490

MDGLGSPSQWLRELRWDSQGLNPISLLIDCAKCVASGSIKNADIGLEYIYQISSPDGNAVQRMVTYFSEALGYRIIKNLPGVYKSLNPSKTSLSSEDILV

QKYFYELCPFLKFSYLITNHAIAEAMECEKVVHIIDLHCCEPTQWIDLLLTFKNRQGGPPHLKITGIHEKKEVLDQMNFHLTTEAGKLDFPLQFYPVVSK

LEDVDFEKLPVKIGDALAITSVLQLHSLLATDDDMAGRISPAAAASMNVQRALHMGQRTFAEWLERDMINAYTLSPDSALSPLSLGASPKMGIFLNAIRK

LQPKLVVITEQESNLNGSNLMERVDRALYFYSALFDCLDSTVMKTSVERQKLESKLLGEQIKNIIACEGVDRKERHEKLEKWIRRLEMAGFEKVPLSYNG

RLEAKNLLQRYSNKYKFREENDCLLVCWSDRPLFSVSAWSFRR

>Glyma10g35920

MEDSEEDELLNLSLSVAANRERKKKGKTTIREHHVSMTTTTTTRNSYESYHEGKIFRLLQMREQMLRQDHRRKGVVEDGNGLPLIHLLLSTATSVDDNNM

DSSLENLTDLYQTVSVTGDSVQRVVAYFVDGLAARLLTKKSPFYDMLMEEPTTEEEFLAFTDLYRVSPYFQFAHFTANQAILEAFEKEEERNNRALHVID

FDVSYGFQWPSLIQSLSEKATSGNRISLRITGFGKSLKELQETESRLVSFSKGFGSLVFEFQGLLRGSRVINLRKKKNETVAVNLVSYLNTLSCFMKISD

TLGFVHSLNPSIVVVVEQEGSRSPRSFLSRFTDSLHYFAAMFDSLDDCLPLESAERLRIEKKLLGKEIKSMLNNDVDGGVDCPKYERMEAWKARMENHGF

VATKISSKSMIQAKLLLKMRTHFCPLQFEEEGGGGFRVSERDEGRAISLGWQNRFLLTVSAWQSV

>Glyma02g47640

MQASEQHRNSSMYYQPLQQIEAYCLPQYRTLNPQLYYHDGGHGTQFSTPSSSELYCTLESSSVALYNSPSTVSFSPNGSPISQQDSQSYPPDQYHSPENT

YGSPMSGSCITDDLSSFNLKHKLRELESVMLGPDSDNLDSYDSAISNGNNFVPLEMDGWKQTMVAISSKNLKHILIACAKAISDDDLLMAQWLMDELRQM

VSVSGDPFQRLGAYMLEGLVARLAASGSSIYKSLRCKEPESAELLSYMHILYEVCPYFKFGYMSANGAIAEAMKDEDRVHIIDFQIGQGSQWITLIQAFA

ARPGGPPHIRITGIDDSTSAYARGGGLHIVGRRLSKLAEHFKVPFEFHAAAISGCDVQLHNLGVRPGEALAVNFAFMLHHMPDESVSTQNHRDRLLRLVR

SLSPKVVTLVEQESNTNTAAFFPRFLETLDYYTAMFESIDVTLSREHKERINVEQHCLARDLVNIIACEGVERVERHEVLGKWRSRFAMAGFTPYPLSSL

VNGTIKKLLENYSDRYRLQERDGALYLGWMNRDLVASCAWK

>Glyma14g01020

MQASEQHRSSSMYYQPLQQIEAYCLPQYRSRNQQLYYHDGGHGTHFSTPSSSELYCTLESSSVAGSFTLYNSPSTVSFSPNGSPISQQDSQSYPPDQYHS

PENTYGSPMSGSCITDDLSSLNFKHKLRELESVMLGPDSDNLDSYESAISNGNNSVPLEMDSWRQTMVAISSKNLKHILIACAKAISDNDLLTAQWLMDE

LRQMVSVSGDPVQRLGAYMLEGLVARLAASGSSIYKSLRCKEPESAELLSYMHILYEVCPYFKFGYMSANGAIADAMKDEDRVHIIDFQIGQGSQWITLI

QAFAARPGGPPHIRITGIDDSTSAYARGGGLHIVGRRLSKLAEHFKVPFEFHAAAISGFDVQLHNLGVRPGEALAVNFAFMLHHMPDESVSTQNHRDRLL

RLVRSLSPKVVTLVEQESNTNTAAFFPRFLETLNYYTAMFESIDVTLPREHKERINVEQHCLARDLVNIIACEGVERVERHEVLGKWRSRFAMAGFTPYP

LSSLVNGTIKKLLENYSDRYRLEERDGALYLGWMNRDLVASCAWK

>Glyma20g31680

MEDSEEDELLNLSLSVAADRERKKKGKTIREHNVSMSSTTTTTRNSYERKIFRLLQMREQMLRQDHHRRKGVVEDGNGLPLIHLLLSTATAVDDNNMDSS

LENLADLYQTVSITGDSVQRVVAYFVDGLSARLLTRKSPFYDMLMEEPTTEEEFLSFTDLYRVSPYFQFAHFTANQAILEAFEKEEERNNRALHVIDFDV

SYGFQWPSLIQSLSEKATSGNRISLRITGFGKNLKELQETESRLVNFSKGFGSLVFEFQGLLRGSRVINLRKKKNETVAVNLVSYLNTLSCFMKISDTLG

FVHSLNPSIVVVVEQEGSRSPRSFLSRFTDSLHYFAAMFDSLDDCLPLESAERLRIEKKLLGKEIKSMLNNDVDGGVDCPKYERMETWKARMENHGFVAT

KISSKSMIQAKLLLKMRTHYCPLQFEEEGGGGFRVSERDEGRAISLGWQNRFLLTVSAWQSV

>Glyma05g03020

MAPPPYSAYNGEIDGENLPLAFDIWENLWAYGYYPHQPISEINSTSTLVDFPFCDGTIVRDNKRVKRTVCFPIYNSINCHSFFNTNNSSSRNSIPKLHFR

DHIRTYTQRYLAAEPVEDTNSSESSGGEEDGCADGVRLVQLLIACAEAVACRDKSHASILLSELKANALVFGSSFQRVASCFVQGLIERLNLIQPIGPAG

PMMPSMMNIMDVASDEMEEAFRLVYELCPHIQFGHYLANSTILEAFEGESFVHVVDLGMSLGLRHGHQWRGLIQNLAGRVGGERVRRLRITGVGLCERLQ

TIGEELSVYANNLGVNLEFSVVEKNLENLKPEDIKVREEEVLVVNSILQLHCVVKESRGALNSVLQMIHGLGPKVLVMVEQDSSHNGPFFLGRFMESLHY

YSSIFDSLDVMLPKYDTKRAKMEQFYFAEEIKNIVSCEGPLRMERHERVDQWRRRMSRAGFQAAPIKMVAQAKQWLLKNKVCEGYTVVEEKGCLVLGWKS

RPIVAVSCWKC

>Glyma11g20980

MDSGSPYQWLRELRWESQGLNPMILLLDCAKCVASGSIKNADIGLEYISQISSPDGSAVQRMVTYFSEALSYRIIKRLPGVYKSLNPPKTSLSSEDILVQ

KYFYDLCPFLKFSYLITNQAIVEAMEFEKVVHIIDLHCCEPAQWIDLLLTFKNRQGGPPHLKITGIHEKKEVLDQMNFHLTTEAGKLDFPLQFYPVISKL

EDVDFEKLPVKIGDALAISSVLQLHSLLATDDDMAGRISPAAAATMNLQRAVHMGQRTFAEWLERDMINAYILSPDSALSPLSLGASPKMGIFLNAMQKL

QPKLVVITEQESNLNGSNLMERVDRALYFYSALFDCLESTVLRTSVERQKLESMLLGEQIKNIIACEGVDRKERHEKLEKWIRRLEMAGFVKVPLSYNGR

IEAKNLLQRYSNKYKFREENDCLLVCWSDTPMFSVSAWSFSR

>Glyma02g46730

MQMSQKHKMSYDSSRFTSEPVQNLGSCCFLQSGNLDYYSSSDNSSHATYPSVCTFEQYCTLESSTNNNLPSLNSSSTVSFSPNNSPVSKLQSKSNVLSSQ

NSLELVNDSLENESCLTLNNDELRHKIRELESALLGHDTYILDTYDTIIPEESDSFMLEAERWKRMMEMISRGDLKEMLCTCAKTVAVNDMETTEWLMSE

LRKMVSVSGDPIQRLGAYMLEALVARLASSGSTIYKVLKCKEPTGSELLSHMHLLYEICPYLKFGYMSANGAIAEAMKEESEVHIIDFQINQGIQWVSLI

QALAGRPGGPPKIRITGFDDSTSAYAREGGLEIVGARLSTLAQSYNVPFEFHAIRASPTEVELKDLALQPGEAIAVNFAMMLHHVPDESVDSGNHRDRLV

RLAKCLSPKIVTLVEQESHTNNLPFFPRFVETMNYYLAIFESIDVALPREHKERINVEQHCLAREVVNLIACEGEERVERHELLKKWRSRFTMAGFAPYP

LNSFITCSIKNLQRSYRGHYTLEERDGALCLGWMNQVLITSCAWR

>Glyma14g01960

MQMSQKHKMSYDSSRFSIEPAQNLGSCCFLQSGNLDYYSSSDNGSHATYPSVCIFEQYCTLESSTNNNFPSLNSPSTVSFSPNNSPVSKLQSKPNVLSSQ

NSLEIVDESLENKSFLTLNDDELRHKIRELESAMLGHDTDILDTYDTIIPEESDSFLKEAERWKRMMEMISRGDLKEMLCTCAKAVAGNDMETTEWLMSE

LRKMVSVSGNPIQRLGAYMLEALVARLASSGSTIYKVLKCKEPTGSELLSHMHLLYEICPYLKFGYMSANGAIAEVMKEESEVHIIDFQINQGIQWVSLI

QAVAGRPGAPPKIRITGFDDSTSAYAREGGLEIVGARLSRLAQSYNVPFEFHAIRAAPTEVELKDLALQPGEAIAVNFAMMLHHVPDECVDSRNHRDRLV

RLAKCLSPKIVTLVEQESHTNNLPFFPRFVETMNYYLAIFESIDVALPREHKERINVEQHCLAREVVNLIACEGAERVERHELLKKWRSRFTMAGFTPYP

LNSFVTCSIKNLQQSYQGHYTLEERDGALCLGWMNQVLITSCAWR

>Glyma13g09220

MSLVISTELADTSYGNAKLYTLKGTDVTPDLSSHNFAPDKHRNMYMTKSYSCESYEKYFHDSPTEELIEPSSSSISGNSVHPDGASSYLLRASSGASVIV

NNPFDTSIWSTRHRDAYQSNSVSDFVENGSPDGLDFDGEMRLKLQELERALLSDEEEEEQGIFETVQSMEIDPDMVEWANPLQDMLLHDSPKESSSSDSS

NLSSISSTTKDTSQNSPQTPKQLLYDCARILSEGNEQEATSMINKLRQMVSIQGDPSQRIAAYMVEGLAARVATSGKCIYQALRCKEPPSNDRLAAMQIL

FEVCPCFKFGYIAANGAIAEAVRDEKKVHIIDFDISQGTQYITLIQTLASMPGRPPHVRLTGVDDPESVQRSIGGINIIGQRLEKLAEELGLPFEFRAVA

SGTSNVTQSMLDCRPGEALVVNFAFQLHHMRDETVSTVNERDQLLRMVKSLNPKLVTVVEQDMNTNTSPFLPRFVEAYNYYSAVFNTLDATLPRESQDRM

NVERQCLAKDIVNIVACEGEERIERYEVAGKWRARLSMAGFTPSPMSTNVREAIRKLIIKQYCDKFKIKEEMGGLHFGWEDKNLIVASAWK

>Glyma04g42090

MSLVRSADLAPTSYENAKLFSLKGTDVRPGLSSQIFGPDKHRSTYMTDTYPSESYEKYFHDSQTEEIIEPSSSSISGSSIHPDVASSYQIRAISGASMVA

NNPFDSSFMSTRHRDAYQSNSGSDLMENGSLDSRNDDGLMRLKLQALERALLDDSDAEEEEEEEEDIFEAAQSMEIDPDIAEWADSMQNMLLHDSPKETS

SSDSNISSISSTKEISQTSQNPRKLLYECAIALSEGNEVEGSSMINNLRQMVSIQGEPSQRIAAYMVEGLAARLAESGKSIYKALRCKEPPTSDRLAAMQ

ILFEVCPCFKFGFIAANNTITEAVKDDMKIHIIDFDINQGSQYINLIQTLASRSSKPPHVRLTGVDDPESVQRSVGGLQNIGQRLEKLAEALGLPFEFRA

VASRTSIVTPSMLDCSPDEALVVNFAFQLHHMPDESVSTANERDQLLRLVKSLNPKLVTVVEQDVNTNTTPFLPRFVEAYNYYSAVFESLDATLPRESQD

RMNVERQCLARDIVNVVACEGEDRIERYEVAGKWRARMTMAGFTSSPMSTNVTDEIRQLIKVVYCDRYKIKEEMGALHFGWEDKSLIVASAWKLPR

>Glyma06g12701

MSLVRSADLAPTSYENAKLFSLKGTDVRPGLSSQIFDPDKHRSMYMTDAYSGEGYEKYFHDSQTEELIEPSSSSISGSSIHPDVASSYQLRASSGASMVA

NNPSDSSFMSTRHHDAYQSNSGSDLMENGSLDSRDDEGLMRLRLKALERALLDDSDAGEDEEEEEEEEEDIFEAAQSMEIDPDIAEWADSMHNMLLHGSP

KESSSSDSNTSSISSTKEISQTSQTPKKLLYECAIALSEGNEVEGSSMINNLRQMVSIQGEPSQRIAAYMVEGLAARLAESGKSIYKALRCKEPPTSDRL

AAMQILFEVCPCFKFGFIAANNAITEAVKDDMKIHIIDFDINQGSQYINLIQTLASRSSKPPHVRLTGVDDPESVQRSVGGLRNIGQRLEKLAEALGLPF

EFRAVASRTSIVTPSMLNCSPDEALVVNFAFQLHHMPDESVSTVNERDQLLRLVKSLNPKLVTVVEQDVNTNTTPFLPRFVEAYNYYSAVFESLDATLPR

ESQDRMNVERQCLARDIVNVVACEGEDRIERYEVAGKWRARMTMAGFTSSPMSTNVTDEIRKLIKTVYCDRYKIKEEMGALHFGWEDKNLIVASAWKLPR

>Glyma12g02060

MAYMCADSGNLMAIAQQVIKQKQQQEQQKQQQQQQTNPIPWNSTPPLPPTDFPDPFQSDPPFHFQPFRFSDFDSDDWMDSLIATPSSYTTDFPFPSSDLN

RLIFPPQSPDSDSPQQPLLKALSECASLSETEPDQAAESLSRLRKSVSQHGNPTERVGFYFWQALSRKMWGDKEKMEPSSWEELTLSYKALNDACPYSKF

AHLTANQAILEATENASNIHILDFGIVQGIQWAALLQAFATRASGKPNKITISGIPAVSLGPSPGPSLSATGNRLSDFARLLDLNFVFTPILTPIHQLDH

NSFCIDPNEVLAVNFMLQLYNLLDEPPSAVDTALRLAKSLNPRIVTLGEYEASVTRVGFVNRFRTAFKYFSAVFESLEPNLAADSPERFQVESLLLGRRI

AAVIGPGPVRESMEDKEQWRVLMERAGFESVSLSHYAISQAKILLWNYSYSSLFSLVESKPPGFLSLAWKDVPLLTVSSWR

>Glyma17g13680

MAPLYSAFNGEIDGENLPLAFDIWATNLWAYGYYPHQPAISENSSSKLVDFPFCDGTIIRDNKRVKRTVCFPIYNSVSCHSFFNTNSSSRNSIPKLHFRD

HIRTYTQRYLAAEPVEEASEDTNSSESSGGEEDGCADGVRLVQLLIACAEAVACRDKSHASILLSELKANALVFGSSFQRVASCFVQGLTERLNLIQPIG

SAGPMMAPAMNIMDAASDEMEEAYRLVYELCPHIQFGHYLANSTVLEAFEGESFVHVVDLGMSLGLRHGHQWRALIQSLANRASGERVRRLRITGVGLCV

RLQTIGEELSVYANNLGINLEFSVVNKNLENLKPEDIEVREEEVLVVNSILQLHCVVKESRGALNSVLQMIHGLGPKVLVMVEQDSSHNGPFFLGRFMES

LHYYSSIFDSLDVMLPKYDTKRAKMEQFYFAEEIKNIVSCEGPLRMERHERVDQWRRRMSRAGFQAAPIKMVAQSKQWLLKNKVCEGYTVVEEKGCLVFG

WKSRPIVAVSCWKC

>Glyma17g01150

MQTSQKHPSSAGAHFYHQPVQGIYQMLQSNLCQDSSSQGTSVSFETCKEQYFTLESCPAPTNDFMDCDDSPSYASVSSKRTPFSPQGSQSCYSDHHQSSD

NTYGSPISGLSSVDDRHQLKHKLRELEISLLAPEESDITDSCGCCVVKGGLHGSSQLAKHNWDQIAENIAQFDLKGALKVCAQAVSDDDVPTARGWIDNV

LGKLVSVSGDPIQRLGAYLLEGLRARLESSGNLIYKSLKCEQPTSKELMSYMHILYQICPYWKFAYISANAVIQETMANESRIHIIDFQIAQGTQWHLLI

QALAHRPGGPPSLRVTGVDDSQSTHARGGGLWIVGERLSDFARSCGVPFEFHSAAISGCEVVRGNIEIRAGEALAVNFPYVLHHMPDESVSTENHRDRLL

RLVKSLSPKVVTFVEQESNTNTSPFFQRFVETLDYYTAMFESIDVACPRDDKKRISAEQHCVARDMVNMIACEGVERVERHELFGKWRSRLSMAGFKQCQ

LSSSVMVATQNLLKEFSQNYRLEHRDGALYLGWMNRHMATSSAWR

>Glyma11g14700

MEPNIPRGAEEQSTAYLMEDSDFSETAKFISQILMEENIDQRPLYDTLTLQVTEKSFYDALTGNIPLSPNPNQHPLLLSPQAQTTITEHGLSDLDSSLQQ

NLFNDADSVSHFKRGLEEATKFLPPVSNLVTGQYPNGEQPINTFEGNSYGFQSRKNHEREEIDTREEEHEGRGHKQSALSLVDETDLSDAIDRVFLSVEN

VCIEHSSLQNGALKPKAPEVGKGRSKKQGRKKETVDLRNLLLMCSQSVYANDIRTANELLKQIRQHSSPVGDASQRLAHYFANGLEARLIGAGSGAIGTF

SFVSSKRITAAEFLKAYQVFLSATPFKKFTYFFANQMIVKAAAKAEIIHIIDYGILYGFQWPILIKFLSNREGGPPKLRITGIEFPQSGFRPTERIEETG

HRLANYCKRYNVPFEYHAIASRNWETIKLEALKIERNELVAVNCHMRFEHLLDESTIEVNSPRNAFLHLIRKINPDIFTQIIINGSYDAPFFATRFREAL

FHYSAIYDMFDTVITSENEWRMTIESELLGREVMNVIACEGSERVQRPETYKQWQVRNTRAGFKQLPLNEELMAKFRSKLKEYHRDFVLDENNNWMLQGW

KGRIFNASTCWFPA

>Glyma14g27290

MSLVISAELADTSYGNAKLYTLKGTDVTPDLSSHNFAPDKHRNMYMTKSHSCESYAKYFRDSPTEELIEPSSSSISGNSVHPDGASSYLLRASSGASVIV

NNPFDTSIWSTRHRDAYQSNSGSDFVENGSPDGLDFDGEMRLKLQELERALLSDEEEEEEGMFETVQSMEIDPDMVEWANPLQDMLLHDSPKESSSSDSS

NLTSISSTTKDTSQNSPQTPKQLLYDCARVLSEGNEEEATSMINKLRQMVSIQGDPSQRIAAYMVEGLAARVATSGKCIYQALRCKEPPSNDRLAAMQIL

FEVCPCFKFGYIAANGAIAEVVRDEKKVHIIDFDISQGTQYITLIQTLASMPGRPPRVRLTAVDDPESVQRSIGGINIIGQRLEKLAEELRLPFEFRAVA

SRTSIVSPSMLNCRPGEALVVNFAFQLHHMRDETVSTVNERDQLLRMVKSLNPKIVTVVEQDMNTNTSPFLPRFIETYNYYSAVFDTLDATLPRESQDRM

NVERQCLAKDIVNIVACEGEERIERYEVAGKWRARLSMAGFTPSPMSTNVREAIRNLIIKQYCDKFKIKEEMGGLHFGWEDKNLIVASAWK

>Glyma16g27310

MEDSEEEELLNLTLSVDAGRKRRKKGRTRDSNNNNSNTNSVIIPIKSNEGMIFRLLQMRELMLRQDHRIRKGVVEDGINNNKNGLPLIHLLLSTATAVDD

QRNYCAALENLIDLYQTVSLTGDSVQRVVAYFADGLAARLLTKKSPFYDMLMEEPTSEEEFLAFTDLYRVSPYYQFAHFTANQAILEAYEEEEERNNKAL

HVIDFDVSYGFQWPSLIQSLSEKATSGNRISLRITGFGNNLKELQETEARLVSFSKGFGNHLVFEFQGLLRGSSRVFNLRKKKNETVAVNLVSYLNTSSC

FMKASDTLGFVHSLSPSIVVLVKQEGSRSLKTFLSRFTESLHYFAAMFDSLDDCLPLESTERLKIEKKVLGKEIKSMLNYDMDGVDYCPKYERMETWKGR

MENHGFVGRKISSKCVIQAKLLLKMRTHYYPLQFEEEGGGGFRVSERDEGRVISLGWQNRFLLTVSSWQPV

>Glyma09g01440

MQTSKKHPTSAGIHLYLQPAQDIDPYTHYQILQSNSCHDNSSSQGTTISFETSKEQYFTLESSPAINDLIGCDSPSYASVSSNRSPFSPQASHSDQHQSS

DNTYGSPTSAHSRYDDDGYELKNKLRELEISLLGPDSDIVDSWHCSYKGGRHRASSPTAKHNWDQIVEMIPKLDLKEVLIRCAQAVADDDIETAVGFMNN

VLAKMVSVGGDPIQRLGAYMLEGLRARLESSGSIIYKALKCEQPTSNDLMTYMHILYQICPYWKFAYTSANAVIGEAMLNESRIHIIDFQVAQGTQWLLL

IQALASRPGGAPFIRVTGVDDSQSFHARGGGLHIVGKRLSDYAKSCGVPFEFHSAAMCGSELELENLVIQPGEALVVNFPFVLHHMPDESVSTENHRDRL

LRLVKSLSPKVVTLVEQESNTNTSPFFQRFVETLSYYTAMFESIDVALPRDDKQRINAEQHCVARDIVNMVACEGDERLERHELLGKWRSRFSMAGFAPC

PLSSSVTAAVRNMLNEFNENYRLQHRDGALYLGWKSRAMCTSSAWRCY

>Glyma12g06670

MLMEEDLEAKPCMFHDSLALQAAEKSFYEVIGETYPSSSSSSSIQNYHNVDSPDESSFSGTTTSTGNSFGSQWNNVDLADYKPSILQTTFPTDFVFQASS

IQSSMNTTSKFAVTNSEFLASSAAGFLGPGSTNLFSKSESVLQFERGVEEANKFLPKGNPLVIDLENPSFRMVPLQQEEIKAERDIDEISAESRGRKNHE

REDEETDLQDGRSNKQSAVYIDDSEISELLDKVLLGTWCRNEPAPSCIGYTDLPSGPSLGKLEETNKSGGGKSRVKKQGNKKGVVDLRTLLILCAQAVSS

DDHVSANELLKQIKQHASPLGDGTQRLAHCFANALEARLAGTGTQIYTALSHKRTSAADMVKAYQMYISACPFKKLSMIFANHTILQLAKEVETLHIIDF

GIRYGFQWPAFIYRLSKQPGGPPKLRITGIELPQPGFRPAERVQETGLRLARYCDRFNVPFEFNAIAQKWETIKIEDLKIKENELLVANAMFRFQNLLDE

TVVVNSPRDAVLKLIRKANPAIFLHATVNGSYNAPFFVTRFREALFHYSTLFDVLDTNVAREDPMRLMFEREFFGRQVMNIVACEGSERVERPETYKQWQ

VRNMRAGFKQLPLDKHLINKLRCKLKGVYHSDFMLLEDGNYMLQGWKGRVVYASSCWVPA

>Glyma15g12320

MQTSQKHPTSAGIHLYHQPAQDIDPYTHYQILQSNSCHEIHDNSSSQGTTISFETSKDQYFTLESSPVINDLIGCDSPSYASVSSNRSPFSPQASHSYHS

DQHQSSDNTYGSPTSSHSTADDSYELKNKLRELEISLLGPDSDIVDSCHCSYKGGCHGASPMAKYNWDQIVEMIPKLNLKEVLIRCAQAVADDDIETAVG

FMNNVLAKMVSVGGDPIQRLGAYMLEGLRARLESSGSIIYKALKCEQPTSNDLMTYMHILYQICPYWKFAYTSANAVIGEAMLNESRIRIIDFQIAQGTQ

WLLLIQALASRPGGPPFVHVTGVDDSQSFHARGGGLHIVGKRLSDYAKSCGVPFEFHSAAMCGSEVELENLVIQPGEALVVNFPFVLHHMPDESVSTENH

RDRLLRLVKSLSPKVVTLVEQESNTNTSPFFQRFAETLSYYTAMFESIDVALPRDDKQRINAEQHCVARDIVNMVACEGDERVERHELLGKWRSRFSMAG

FAPCPLSSLVTDAVRNMLNEFNENYRLEYRDGALYLGWKNRAMCTSSAWRCY

>Glyma11g14720

MDSNFPGASEDQGTPYLSDSLGFATMEDNDFSETAKFISQILMEENVDQKPFYDSLTLQVTEKSFYDALAGNLPLSPDQHPVLLSPEAETTTTTTTSSSS

SSSNNNFSDEYSRELKLRSPDSISVSAFQFKSQPPPSVTVSDAVSDLDSSIAKLLAHNIFNHVDSVSQFRRGFEEASKFLPPGPNLVTALHSKREEPINS

FGDNSYGLLKGRKNHQRQEIETREEGEGERSNKQSALSLVDESDLSDAFDRLLLHEGNLCDEHIRLTSGSVNVEERDGGKGRSKKQGRRKKETVDLRNLL

LMCSQSVYANDNRTANELLKQIRQHSSPVGDASQRLAHYFTNGLEARLVGDGTSAQGMYTFLSSKNITVAEFLKAYQVFTSSSPFKKFIHFFANKMIMKA

AAKAETVHIIDFGILYGFQWPILIKFFSNREGGPPKLRITGIEFPQPGFRPAERIEETGHRLANYCKRYNVPFEYNAIASKNWENIQVEALKIQSNELVA

VNCHLRFENLLDESIEVNSPRNGVLHLIRKINPDIFTQSITNGSYNAPFFATRFREALFHYSAIYDLIDTVIPRENEWRLMLERELLGREIMNVIACEGS

ERIERPETYKQWYVRNTRAGFKQLPLNEELMAKFRTKLKEWYHRDFVFDEDNKWMLQGWKGRILYASTCWVPA

>Glyma08g43780

MQTPQNHKISYGSGGFYVEPVQNLDSYCIPSSENIDNYSSSDNSSQTTYPSVQTLEQYCTLESASTGNSFPSQNSPPALSFSSNNSLLSKLESNSYVLRP

QHSLEIASGSPEDDSYLTHDLDGLTHKIRELETAMLGPNADMLDIYGTVIPEPDSFLLEAEKWKKMMEISCRGDLKEMLYMCAKAMAVNDMETTDWLVSE

LRKMVSISGNPIQRLGAYILESFVARIGASGSTIYKSLKCSEPTGNELLSYMNVLYEICPYFKFGYMSANGAIAEALREESEVHIVDFQIGQGTQWVSLI

QALARRPVGPPKIRISGVDDSYSAYARRGGLDIVGKRLSALAQSCHVPFEFNAVRVPVTEVQLEDLELRPYEAVAVNFAISLHHVPDESVNSHNHRDRLL

RLAKQLSPKVVTLVEQEFSTNNAPFLQRFVETMNYYLAVFESIDTVLPREHKERINVEQHCLAREVVNLIACEGEERVERHELLNKWRMRFTKAGFTPYP

LSSVINSSIKDLLQSYHGHYTLEERDGALFLGWMNQVLVASCAWR

>Glyma07g39650

MQTSQKHPSSAGVHFYHQPVQGIYQMLQSNLCHDSSSQGTSVSFETCKEQYFTLESCPAPTTCFVDCDDSPSYASVSSKRTPFSPQGSQSCYSDHQQSSD

NTYGSPISGLSSVDDGHELKHKLRELEISLLGPEQSDSCGCCVVKGGLQGSSQLAKHNWDQIAENVAQFDLKGVLRVCAQAVSDDDVPTARGWMDNVLGK

MVSVSGDPIQRLGAYLLEGLRARLESSGNLIYKSLNCEQPTSKELMSYMHILYQICPYWKFAYISANAVIEEAMANESRIHIIDFQIAQGTQWHLLIQAL

AHRPGGPPSLRVTGVDDSQSIHARGGGLQIVGERLSDFARSCGVPFEFRSAAISGCEVVRGNIEVLPGEALAVSFPYVLHHMPDESVSTENHRDRLLRLV

KRLSPKVVTIVEQESNTNTSPFFHRFVETLDYYTAMFESIDVACPRDDKKRISAEQHCVARDIVNMIACEGVERVERHELLGKWRSRLSMAGFKQCQLSS

SVMVAIQNLLKEFSQNYRLEHRDGALYLGWMNRHMATSSAWR

>Glyma18g09030

MQTSQNHKISYGSGGFYVEPVQNLESYCMPSSENIDNYSSSDNSSQTTYPSVQTLEQYCTLESASTGNSFPNQNSPPALSFSSNNSPLSKLESNSYVLRP

QHSLEIASGSPEDDSYLTHDLDDLTHKIRELETAMLGPNADMLDIYGTVIPEPDSFLLEAEKWKKLMEMSSRGDLKEMLYTCAEAMARNDMETTDWLVSE

LRKMVSISGNPIQRLGAYILESFVARMAASGSTIYKSLKCSEPTGNELLSYMHVLYEICPYFKFGYMSANGAIAEALKEESEVHIVDFQIGQGTQWVSLI

QALAHRPGGPPKIRISGVDDSYSAYARGGGLDIVGKRLSAHAQSCHVPFEFNAVRVPASQVQLEDLELLPYEAVAVNFAISLHHVPDESVNSHNHRDRLL

RLAKRLSPKVVTLVEQEFNTNNAPFLQRFDETMKYYLAVFESIDTVLPREHKERINVEQHCLAREVVNLIACEGEERVERHELLNKWKMRFTKAGFTPYP

LSSVINSSIKDLLQSYHGHYTLEERDGALFLGWMNQVLIASCAWS

>Glyma15g04190

MDRRAVSSINTDQECPLDDTDSSSALFSYIKQMLMEDDTQESYSIFHDSLALQHTERSFYQVITHNYPPSSSSPHHQSPEQSLSVSSSDSSSSCSTNSTS

SSADSPFRSLPPLLPHSTFPIPHNFFFHSNSTQSSISTTLGFFDNPLAEISDSAFLQQFERGVDQGTRFLPLHTTPFNINVDPNNTAFSSSFTKTKTPPP

QMLIKTEAEGEPFLAGRKQRQREEYEADGRSRKQSAPYMDDSELSELFDKVLLGTGLGKGVPPDTTHETILTNMFGGDASKSDEEVVDLGTLLMLCAQAV

ASGSSPSFAKQLVMQIKQHSSPIGDETQRLAHYFGNALEARLDGTGYQVYSVLLSSKRTSAKDMVKAYHVYLSICPFEKLAVIFANNSICNLSEDAKTIH

IIDFGIRYGFKWPALISRLSRRPGGPPKLRITGIDVPQPGLRPQERVLETGRRLANYCKRFNLPFEFHAIAQRWDTIRVEDLKIETDEFVAVNCLFQFEH

LLDETVVLNNPRDAVLKLIKKANPDIFVHGIVNGSYDVPFFVSRFREALYHYSALFNMLDTNVGREDPIRLMYEKELFGREIMNIIACEGCERVERPQTY

KQWQLRNMRNGFRPLPLDQRIIDKLKGRLRDDAYNNNFLLEVDGNWVLQGWKGRILYASSCWVPA

>Glyma11g14670

MLSTDSLLENFPFVNGPISVFSNQNPESGFKVDDSCSPSESVTDSGPSSGTSSNGEHAESTKHSNPILRYISDILMDEEVDLERKPCMLQDCLRLQAAEK

SFYDALVRSYPSSTGQFNDNPDPDDNFGGTTSSESFSSYTTDNSCESDWFNGASDLDSSFLQRSLIDSLEHTYVAPDLFRETQAGVHFSNGAWNLIHLQN

KPRAIEDGVMRGSVTATGLREKRSYQMNDISHEEEEKSNKLSAVYLDDSEPSSMFDDVLLCKDGKSPSIFYAGREPSPSQIADSGGSNGKKTRSKKGSNK

RTSASATVDLWTLLTQCAQAVASFDQRTANETLKQIRQHSSPYGDGLQRLAHYFADGLEKRLAAGTPKFISFQSASAADMLKAYRVYISASPFLRMSNFL

ANSTILKLAQNESSIHIIDFGISYGFQWPCLIQRLSERPGGPPKLRMMGIDLPQPGFRPAERVEETGRWLEKYCKRFGVPFEYNCLAQKWETIRLEDLKI

DRSEVTVVNCLYRLKNLSDETVTANCPRDALLRLIRRINPNIFMHGIVNGTYNAPFFVTRFREALFHFSSLFDMFEANVPREDPSRLMIEKGLFGRDAIN

VIACEGAERVERPETYKQWQVRNQRAGFKQLPLAQEHVNRVKEMVKKEYHKDFVVGEDGKWVLQGWKGRILFAVSSWTPA

>Glyma02g08241

MEDSEEEELLNLTLSVDAGRERRIKKGRTRDNNNNTNSVIIPMKSNDGYEGKIFRLLQMRELMLRQDHRRKGVLLEDVNNNNNNGLPLIHLLLSTATAVD

DDQRNYCAALENLIDLYQTVSLTGDSVQRVVAYFADGLAARLLTKKSPFYDMLMEEPTSEEEFLAFTDLYRVSPYYQFAHFTANQAILEAYEEEEERNNK

ALHVIDFDISYGFQWPSLIQSLSQKATSGKRIFLRITGFGNNLKELQETEARLVSFSKGFGNHLVFEFQGILRGSSRAFNLRKRKNEIVAVNLVSYLNTL

SSFMKVSHTLGFVHSLSPSIVVLVKQEGSCRSLKTFLSRFTESLHYFAAMFDSLDDCLPLESTERLRIEKQLLGKEIKSMLNYDMDDGVEYYCPKYERME

TWKGRMENHGFVGRKISSKCVIQAKLLLKMRTHYYPLQFEEEGGGGFRVSERDEGRVISLGWQNRFLLTVSAWQPV

>Glyma11g14750

MDPHLKSNFINGVKPSQVPNTVDVEDHSFDPVNGMMKPFDFGFMDNRFLISPDPNAAQNPFSSSTDEDYPLDEIDFSATVLRYINQMLMEEDLEAKPCMF

HDTLALQAAEKSFYEVIGETYHSSSIQNYHNMDSPDESSFSGATTSTSNSFESQWNNVDLADYKPSILQTTFPADFVFQASSIQSSMNTTSNFAVTNSQF

LASSVAGFLDPGSTNLFSKSESVLQFERGVEEANKFLPKWNPLVFDLENPSFRMVPPQQVKIKAETERDEISAESRGRKNHEREDEEADLQDGRSNKQSA

VYIDDSEISELLDKVLLGTGCRNEQAPSCIGHADLPNGPSLGKLEETNKSGGGKSHVKKQGSKKEIVDLRTLLILCAQAVSSDDRMSANELLKQIKQHAS

PLGDGTQRLAQCFASALEARLVGTGTQIYTALSHKRTSAADMVKAYQMYISACPFKKLSMIFANHTILHLAKEVETLHIIDFGIRYGFQWPALIYRLSKQ

PGGPPKLRITGIELPQPGFRPAERVQETGLRLTRYCDRFNVPFEFNAIAQKWETIKIEDLKIKENELLVANAMFRFQNLLDETVVVNSPRDAVLKLIRKA

NPAIFLHANVNGSYNAPFFVTRFREALFHYSTLFDVLDTNVACEDPMRLMFEREFFGRQVMNIVACEGCERVERPETYKQWQVRNMRAGFKQLPLDKHLI

NKLRCKLKDAYHSDFMLLEDDNYMLQGWKGRVVYASSCWVPA

>Glyma15g04166

MEFTKYSNPILRYISDILMDEEDDLECKPCMLQECLRLQAAEKSFHDALLHQNPSPFRDENTCITDSDEIFGRIPSFVSSGSCTTDNSCESDLLNGVSEF

NSSFLQLQTPLVDSPEDGYFHDGTWNLFQSHTKPLMVEEGTPASALREKRSHGMDGHASHEKEGRRGSKVSAIFSDDSEPREILDEVLLYQTGGSQPLYS

APTEPSQRVDLGRSNGKATRLRSKKVSTNMETTVDLWTLLTQCAQAVANYDQRNANELLKQIRQHSSPFGDGLQRLAHYFANGLETRLAAGTPSYMPLEV

ATAADMLKAYKLFVTSSPLQRLTNYLTTKTIISLVKNESSVHIMDFGICYGFQWPCLIKKLSDRHGGPPRLRITGIDLPQPGFRPAERVEETGRRLANFC

KKFNVPFEYNCLAQKWETIRLADLKIDRNELTVVSCFYRLKNLPDETVDVKCPRDAVLKLIRKINPNVFIHGVVNGAYSAPFFLTRFREALYHFSSLFDV

YEANVPREDPQRVMLEKGLFGRDAINVVACEGAERVERPETYKQWQVRNLRAGFKQLPLDPQLVNDAKDIVKREYHKDFVVAENDKWVLLGWKGRILNAI

SAWTLA

>Glyma11g14710

MSDIDVYECLKRKKTLLKLLLNVCCITISNHISFFLNRVKTYFSVVGFGEDDGIAYLSDSLGFATMEDNDFSETAKFISQILMEENVELEQSPFYDSLTL

QVTEKSFYDALAGNLLLSPQASNTNFSVENSRELNLPSPDSLSVSALQFNPHALSQPPPLVNVSEGVSDLDSSIARLLAHNIFNDVDSVSHFRRGFEEAS

RFLPPGPNLVTALHSNAQEPINSFRENSYGLLKGRKNLERQEINTREEERGGRSNKQSAFSFVDESDLSDAIDRVFLSVENVCSEHSSLQSGPLRAEEQD

RGKGLSKKQERRKQETVDLRNLLLMCSQSVYANDNRTANELLKQIRQHSSPVGDASQRLAHYFANGLEARLVGDGTSSQGMYTFLSSKNITAAEFLKTHQ

DFMSASPFKKFTYFFANKMIMKAAAKVETVHIIDFGILYGFQWPILIKFLSNREGGPPKLRITGIEFPQPGFRPTEKIDETGRRLANYCKRYSVPFEYNA

IASKNWETIRIEALKIESNELVAVNCHQRFENLLDDSIEVNSPRNAVLHLIRKINPNIFTQSITNGSYNAPFFAPRFREALFHYSAIYDLIDTIIHRENE

RRLMIERELLGREIMNVIACEGSERIERPETYKQWQVRNMKAGFKQLPLDEELMAKFRTELRKWYHRDFVSDEDSNWMLLGWKGRILFASTCWVPA

>Glyma12g06655

MESNFPGAYHLFGEDQGIPYLSDSLGFPTMDPSSPEDNDFSETVKFISQILMEENVDQRPFYDSLTLRVTEKSFYDALTGNQPPFVLCSEAETNTITSNN

SGSNFLNENSRELNIPSPLSVSVSAIHFNPNPLSQPLPSVTVSDRVSVLDSSIAKLLAQNISIEVDSVSKFRRGLEEATKFLPPEPKLVTGLDLYREQAI

NSSGDTSYRLNSRKNHGCEVRDTREEEEEEGGRSNKQSALSLVDETDLSDAFDQVLLHEENLWNEHTCLQSEAEKVEGPNGGKGGSDKKVRKKKKTVDLR

NLLLMCSQAVYASDIRAANELLKQIRQHSSPIGDASQRLAHYFANGLEARLVGDGTSTQGMYTFLSSKNNTFSELLKAYQVFSSSSPFKKFAYLFENTMI

MKAAASAETVHIIDFGILHGFQWPMLIRLLSNREGGPPKLRITGIEFPQPGFRPTEKIEETGRHLANYCKRYNVPFEYNAISSRNWETIQLEALKIASNE

LVAVYCHQRFENLLDECTIEVNSPRNAVLHLIRKINPDIFTHSITNGSYNAPFFTTRFREALFHYSAISDKNDTVISRENERRLMVERELYGREIMNVIA

CEGSDRIERPETYKRWQVRNMKAGFKQLPLNEELMAKFRSKLKEYHRDFVLDENNNWMLQGWKGRILFASSCWVPA

>Glyma12g06640

MEANFPRRAHQFGEEEESILYVSDSLGFAATDPSLRDPSLEDNDFSETAKFINQILMEDNVEQMPFYDSLNLQVTEKSFYNALTGNIPLSPNQHPLVLSP

QAETTPTTSNSSNNSNHNFLDENSRELNPSPDSVSVLAFQFNPNSLSQPPSVTVNHGLSNLDSSTAKLLAHNIFNDADSLSQFRRGLEEATRFLPPGPKL

VAGLDSKGEEPINTLGENSYGSKGRKNHEREDIDTREEEERRSNKQSALSLVDESDLSDAFDRVVLLSVENVCNEHCSLQSETVKAVEPGGVKGRPKNQA

TNKETVDLRNLLMMCSQSVYANDKRAANELLEQIRQHSSPSGDALQRLAHYFANGLEARLVGEGMFSFLKSKRSTAAEFLKAHQDFLSVSPFKKFTYFFA

NKMIMKAAVKAETVHIIDFGIQYGFQWPMLIKFLSNREGGPPKLRITGIDFPQPGFRPTEKIEETGCRLANYSKRYSIPFEYNAIASRNWETIQVEALNI

ETNELVAVNSLMKFENLMDETIEVDSPRNAVLHLIRKINPHIFTQCIVNGTYNAPFFTTRFREALFHFSTIYDLCDTVIPRENEWRMLIEREVLGREAMN

VIACEGSERVERPETYKQWQARNMKAGFKQLPLNEELLAKFRNELRKSYHRDFVLDEDKNWMLQGWKGRILYASTCWVPA

>Glyma15g04173

MDPNFIISGNDFDESKQWDLNGHYTNLLNDPLSDVGPLGFSHNSILNSEPFVSSSASAAAASSSTATVADPSPEEDTDFSETFKFISQILLEENFEQKPC

MCYDPLTLQHTEKSFYEALELEPSLLLSPNQHPLESPDGNSSNSTTDSANSHDLKPSSPNTPASDALHSSSHAPSLVVPPHALTKINDGTVDLDSSVTKL

LAENIFSDADSMLQFKRGLEEASKFLPQGPQLFTGLESPTVSSEPKGRGVALKMENSFGVKNRKNHARQDDEEERSNKQSAVSAVCVEEESEISEIFDRV

LLSVENVPLCAENNGSVAVGDSNTKLIEKSSLSDGGKVRSKRQGRKKETVDLRTLLVLCAQAVSSSDNRTANELLKQIRQHSSALGDASQRLAHYVANAL

EARLVGDGTATQIFYMSYKKFTTTDFLKAYQVLISACPFKKFAHFFANKMIMKTADGAETLHIIDFGILYGFQWPILIKFLSGRRGGPPKLRITGIEYPQ

PGFRPTERIEETGCRLAKYCKRFNVPFEYKAIASRNWETIQIEDLKIERNEVLAVNCLVRFKNLLDESIEVNSPRKAVMNLIRKMKPDIFVHCVVNGTYN

APFFLTRFREALFHYSSMYDMFDTLVSRENEWRLMLEREFLGREIMNVVACEALERVERPETYKQWQARNTRAGFKQLPLDKEIMTKFRGKLREWYHRDF

VFDEDGNWMLQGWKGRILYASTCWVPA

>Glyma12g06630

MLSTDSLLENFPGSVNGFIFENGPVSVFSNQNPASGFEVDDSVSPSESATDSGPSSGASSNREHVESTKHSNPILRYISDILMDEEDDLERKPCMLQDCL

RLQAAEKSFYDALVRSYPSSPRQFDDNPDQDDNFGGTTSSESFSSYTTDNSCESDWFNGASDFDSSFIQRSLIYSPEHAYVAPDPFRETQAGVHFSNGAW

NLIHPQNKPRVIEDGVMQGSVTATGLREKRSYLMNDMSHEEERSNKLSSVYSDDSEPSSMFDEVLLCKDGKSPSIFYAGREPSPSQIADSGGSNGKKTRS

KRGSNKGTRASVTTVDLWTLLIQCAQAVASFDQRTANETLKQIRQHSSPFGDGLQRLAHYFADGLEKRLAAGTPKFISFQSASAADMLKAYRVYISASPF

LRMSNFLANRTILKLAQNESSLHIIDFGISYGFQWPCLIQRLSERPGGPPKLLMTGIDLPQPGFRPAERVEETGRWLEKYCKRFGVPFEYNCLAQKWETI

RLEDLKIDRSEVTVVNCLYRLKNLSDETVTANCPRDALLRLIRRINPNIFMHGVVNGTYNAPFFVTRFREALFHFSSLFDMFEVNVPREDPSRLMIEKGV

FGRDAINVIACEGAERVERPETYKQWQVRNQRAGFKQLPLAPEHVNRVKEMVKKEHHKDFVVDEDGKWVLQGWKGRILFAVSSWVPA

>Glyma15g15110

MDDLYYAGFSTDDNSSSSNDFFWDNEGLNDVRKFQFSGVEDHEEYGGIDSFYSNFGFFPDDPSEEGYLLSTNHQKYHQIFDDYGLLDDNLQFDMVSPPLQ

FDEQYRTMVPLCNSTKDMPHSTTPLASLEILKSYGKGFKRFWNEGNNTMQPIDDETLATDEVIAGRNLSTEDLMRIAGTRFIQSSSSSSDSESLPFLENH

PFGFSFSGFSDEEKEDLELAESLLACAEKVGNKQFERASKLLSHCESLSSKTGNPVKRIVHYFAEALRQRIDTETGRVSSKDLQKGQPFDPEEAAKELTP

AILAFVEDLPFCKVAQFTAAQAIIEDVAEAKRIHIIDLEIRKGGQWTIVMQALQLRHECPIELLKITAVESGTTRHIAEDTGQRLKDYAQGLNIPFSFNI

VMVSGMLHLREDLFEIDPEETIAVYSPYCLRTKLQQSDQLETIMRVIRTISPDVMVVAEIEANHNSKSFVNRFVEALFSFSAFFDCFEACMKGDEKNRMI

IESMYFSPGIRNIVAAEGAERRSRSVKIDVWRAFFSRFGMEEKELSTLSLYQAELVAKRFPCGNFCTFERNGHCLLIGWKGTPINSVSVWKFL

>Glyma01g40180

MDTTLFRVVSSFQHQHQPDHDQSLNNSTTSSSSRSSRQQQNYPYPQEDEECFNFFMDEEDLSSSSSKHYCPYQPHPPSTTTIHHSFSPTPCDFEFSGKWA

QDILLETARAVADKNTTRLQQLMWMLNELSSPYGDTDQKLASYFLQAFFSRISQAGDRTYRTLASASEKTCSFESTRKTVLKFQEVSPWTTFGHVASNGA

ILEALEGEPKLHIIDISNTYCTQWPTLFEALATRNDDTPHLRLTSVVTADATAQKLMKEIGARMEKFARLMGVPFKFNVVHHVGQLSDLDFSMLDIKEDE

ALAINCVNTLHSIAAVGNHRDAVISSLRRLKPRIVTLVEEEADLDVGLEGFEFVKGFEECLRWFRVYFEALDESFPRTSNERLLLERAAGRAVVDLVACS

AAESVERRETAARWARRMHGGGLNTVAFSEEVCDDVRALLRRYREGWAMTQCSDAGIFLTWKEQPVVWASAWRALT

>Glyma13g41240

MLVFSFPPYALVLFHISLASMDPNFIISGYDFDESNHWDLNGHYANLLNDAFSDVGPLGLSQNAILNSEPFVSSSGSAATDPAATTATLADPSPEEDTDF

SETFKFISQILLEENFEQKPCMCYDPLTLQHTEKSFYEALELEPSLPLSPNQHPLESPDGNSSNSISDSANSHDLKPSSPNTPVSDALHSSSHAPSFVVP

PHALNKINDGTVDLDSSVTKLLAENIFSDTDSMLQFKRGLEEASKFLPRRPQLFTGLESTAVSAEPKGKGVALKMENSIGVRSRKNHARQDEEEEEERSN

KQSAVSAVCVEEESEISEIFDRVLLSVENVPLCAEKNGSVAQAEKSNLSDGGKVRSKRQGRKKETVDLRTLLILCAQAVSSSDNRTANELLKQIRQHSSA

LGDASQRLAHYVANALEARLVGDGTATQIFYMSYKKFTTTDFLRAYQVFISACPFKKFAHFFANKMIMKTADGAETLHIIDFGILYGFQWPILIKFLSRR

PGGPPKLRITGIEYPQPGFRPTERIEETGRRLAKYCKRFNVPFEYKAIASRNWETIQIEDLKIERNELLAVNCLVRFKNLLDESIEVNSPRNAVLNLIRK

MKPDIFVHSVVNGSYNAPFFLTRFREALFHYSSIYDMFDTLISRENEWRLMLEREFLGREIMNVVACEALERVERPETYKQWQARNTRAGFKQLPLDKEI

MTKFRGKLREWYHRDFVFDEDGNWMLQGWKGRILYASTCWVPA

>Glyma15g04160

MSCNFLVEFNMNSLVQNFSGPITEFSNQNQVEKGFDFEFDDSSSSLSSGASSGGESTEVTKYSNQILRYISDILMDEEDDLERKPCMLQECLRLQAAEKS

FHDALLHQPSSRFRDESVSITDSDENYGRNASFESNGSCTTDNSCESVLVNGVGEFDSSFLQLQTPLVDSPHDPSGESPGVGFFHDPFVKSQAAGYFHDG

TWNLFQSQSQTKKPLMVEEGFSASAPREKRSHGMDDYAFHEQEGRRGSKVSAVFSDESESPEILDEVLLCQSGRSQSLLCAAAEPSQSVNLGGSNGKGTR

SRSKKVSTKAGTAVDLWTLLTQCAQAVASFDQRNANDLLSQIRQHSSAFGDGLQRLAHYFANGLETRLAAGTPSYMPLEGTTSADMLKAYKLYVTSSPLQ

RLTNYLATQTFLVENEGSVHIIDFGICYGFQWPCLIKKLSERHGGPPRLRITGIELPQPGFRPAERVEETGRRLANYCKKFNVPFEYNCLAQKWETIKLA

DLKIDRNEVTVVSCFYRLKNLPDETVEVKSPRDAVLKLIRMINPNMFIHGVVNGTYSAPFFLTRFREALYHFSSLFDMFEANVPREDPERVMLEKGLFGR

DAINVIACEGAERVERPETYKQWQVRNQRAGFKQVRFDPQLVNHEKEMVKKEYHKDFVVAEDGKWVLLGWKGRILNAISAWTPA

>Glyma13g41220

MMNATNDEESCLDKSVLSYIKQMLMEDDTEERYSMFHDSLALQHTERSFLEAINHNYPSPSYSSSTHYHLDNYPSVDSPEPCLSACSADNITFSASSSCA

SNNTTSSSEFPLRSLYPLLPDTTDEFVFHSNSTQSTINTPFGFFDNPLAEIFERRVDLGTLFLPANTPFSSSFTKVPHVVIKTEAEEGDHFLTGRKQRER

EEYEAADGRSRKQSAAHMDESELSELFDKVVLGTDLRKRVPPNTTHKTTILTNMLYGGDVWENDDQVVDLRTLLMLCAQAIASDNPSSAKQLVKQIMQHS

SPTCNETQRLAHYFGNALEARLDGTGYKVCSALSSKRTSAKDMIKAYHVYASVCPFEKLAIIFANNSIWNPSVDAKAIHIIDFGIRYGFKWPALISRLSR

RSGGPPKLRITGIDVPQPGLRPQERVLETGRRLANFCKRFNVPFEFNAIAQRWDTIRVEDLKIEPNEFVAVNCLFQFEHLLDETVVLNNSRDAVLRLIKN

ANPDIFVHGIVNGSYDVPFFVSRFREALFHYTALFDMLDTNVARQDPMRLMFEKELFGREIVNIIACEGFERVERPQTYKQWQLRNMRNGFRLLPLDHRI

IGKLKDRLRDDAHNNNFLLEVDGDWVLQGWKGRILYASSCWVPA

>Glyma06g11610

MDIDAIHNLDFSGYSTITNTTPSSDDDYGCNWNHWSPVVNWDAFTGAHDDFHQIIDSIMCDGAAPEEDNLSPDDHVASNSPSVSITEEDDDAGDDSKGLR

LVHLLMAAAEALSGAPKSRDLARVILVRLKELVSSHAAPHGSTMERLAAYFTDALQGLLEGAGGAHNNNNKHHHHYITSCGPHHHHRDDHHHQNDTLAAF

QLLQDMSPYVKFGHFTANQAILEAVAHDRRVHIVDYDIMEGVQWASLIQALASNKTGPPGPHLRITALSRTGSGRRSIATVQETGRRLAAFAASLGQPFS

FHHCRLEPDETFKPSSLKLVRGEALVFNCMLNLPHLSYRAPESVASFLSGAKALKPRLVTLVEEEVASIVGGFVARFMDSLHHYSAVFDSLEAGFPMQGR

ARALVERVFLGPRIVGSLARMGEEEERGSWGEWLGAAGFRGVPMSFANHCQAKLLIGLFNDGYRVEELGSNKLVLDWKSRRLLSASLWTSSSHSD

>Glyma03g10320

MTMNPHLTGFSGSTNQSFPILQNQRFDNGPRFENLFFDQSRNFDLQCDPNLIPANTPSSSTVTHEEHSPEDCDFSDAVLSYISQILMEEDLEDNTCMVQD

SLDIQAAEKSFYEVLGEKYPPSPRNTSLMNDGVGGYDFSGDYGNCPDTNGDLMSIFTNQFLPPNSGSFPAHSLHGDGISHSSYNPSNSVEGLVNSSKSII

QVPDLNSESESIWQFQKGVEEASKFLPSANGLFANLSEPEPKEGKDELSFKVEKEEGEYVNGGSKGRKHPQIDEADDEENRSSKQAAIYSEPTLRSDMAD

IILLHSTGDGKDHFVARREALQNKTQKSVLPKGQSKASSSGKGRGKKQGGRKEVVDLRTLLFLCAQAVAADDHRNANELLKHIRQHSTPFGDGNQRLAHI

FADGLEARLAGTGSQIYKGLVGKRTSAANYLKAYHLYLAACPFRKISKFTSNITIRESSAQSMKVHVIDFGIFYGFQWPTFIQRLSWRAGGPPKLRITGI

DFPQPGFRPAERILETGRRLAAYAEAFNVPFEYKAIAKKWDTIQLEELEIDRDEFLVVTCFYRGKNLLDESVVVDSPRNNFLTLIRRINPKLFIHGIMNG

AFDAPFFVTRFREALFHYSSLFDMLETIVPREDWERMLIEKEIFGREALNVIACEGPERVERPESYKQWQARILRAGFVQQSFDRRTVKMAMEKVRGSYH

KDFVIDEDSQWLLQGWKGRIIYALSCWRPA

>Glyma11g14740

MESNFTGADDLFAEDKGMPYLSDSLGFATMDPSPEDNNDFLETANFISHILTEENVEQRPFYDSLTLNRNPSTMLSPATYLFHPFNTLSPEDETTTSSSN

DSNNNFSDENSRELRLPSPDSLFVSAFQFNPNTLSQPPPSVTVSDGVSDLDSSIANLLAHNIFNHVDSVSQFRRGLEEASKFLPPGPNLVTDLDSYGEQI

IKFFWREFLWVEGRTANELLKQIRQHSSAIGDASQRLVHYFANGLKTCLIGDGTGAQGMYFFLTSKKITAAEFLTTYLVFLSASPFKKFIHFFANKMIMK

AAAKAETVHVIDFGILYGFQCPSLIKFLSNRESGPPKLRITGIEFPQPGFRPTERIEETGHCLANYCKHYNVPFEYNAIASKNRESIQVEALKIQSNELV

AVNCHLRFENLLNESIEVNSPRNAVLHLIRKINQDIFTQSITNGSYNAPFFATRFREALFHYSATYELIDTVIPRENEWRLMIERELLGREIMNVIACEG

SQRIERPETYKQWQVRNTRAGFKKLPLNEELMAKLRTANGTTDFVLDEDNNWLLQGWKGHILYASTCWVPA

>Glyma13g41261

MSSNFLVEFNMNSLVQNFGGPISVFSNQDQVEKGFDFEFDDSSSPSSGTSSGGESTEVTKYSNPILRYISDILMDEEDDLERKPCMLQECLRLQAAEKSF

HDALLHQNPSSCFSDENYGRTVSFESCTTDNSCESELVNGVGEFDSSFLQLQTPLVHDPFGESQAAGYFHDGTWNLFQSQSQTKPLMVEEGSSASAPREK

RSHGMDDYASHEQEGRRGSKVSAVFSDESESPEILDEVLLCQSGRSQALLCAATEPSQSVDLGGSNGKATRSRSKKVSAKAGTAVDLWTLLTQCAQAVAS

FDQRNANDLLSQIRQHSSAFGDGLQRLAHYFANGLQIRLAAGTPSYTPLEGTTSADMLKAYKLYVTSSPLQRLTNYLATKTIVSLVGNEGSVHIIDFGIC

YGFQWPCLIKKLSERHGGPPRLRITGIELPQPGFRPAERVEETGRRLANYCKKFKVPFEYNCLAQKWETIKLADLKIDRNEVTVVSCFYRLKNLPDETVD

VKSPRDAVLKLIRRINPNMFIHGVVNGTYNAPFFLTRFREALYHFSSLFDMFEANVPREDPERVMLENGLFGRDAINVIACEGAERVERPETYKQWQVRN

QRAGFKQVRFDPLLVNDEKEMVKKEYQKDFVVAEDGKWVWLGWKGRILNAISAWTPA

>Glyma04g43090

MEIDMDMDFSGYSTITNTIPSSDDDYGCNWNHWSPVVNWDAFTGAHDDFHHLIDSIMCDSAAAEEDNLSPDDHAASNSPSASVTEEEDDDADEETGPVDD

SKGLRVVHLLMAAAEALTGAPKSRDLARVILVRLKELVSHAAPPHGSNMERLAAYFTDALQGLLEGASGGAHNNKRHHHYNIITSSCGPHHRDDHHNHQS

NTLAAFQLLQDMSPYVKFGHFTANQAILESVAHERRVHIVDYDIMEGVQWASLMQALASNKTGPPGPHLRITALSRTGSGRRSIATVQETGRRLTAFAAS

LGQPFSFHHCRLDPDETFKPSSLKLVRGEALVFNCMLNLPHLSYRAPDSVASFLSGAKALKPRLVTLVEEEVGSSAGGFVGRFMESLHHYSAVFDSLEAG

FPMQGRARALVERVFFGPRIVGSLGRLYRTGEEERGSWGEWLGAAGFRGVPMSFANHCQAKLLIGLFNDGYRVEELGTNKLVLDWKSRRLLSASLWTQIN

SF

>Glyma11g05110

MDTTLFRVVSSFQHHQADQDHQSLNNSTTSSSSRSSRQEQNYPYPQEHDEECFNFFMDDEDLSSSSSKHYYPYQPHPPSTTDHSFSPTPGVDVVFPFEFS

SGKWAQDILLETARAVADKNTTRLQQLMWMLNELSSPYGDTDQKLASYFLQAFFSRITQAGDRTYKTLASASEKTCSFESTRKTVLKFQELSPWTTFGHV

ASNGAILEALEGEPKLHIVDISNTYCTQWPTLFEALATRNDDTPHLRLTSVVTAGATAQKVMKEIGARMEKFARLMGVPFKFNVVHHVGQLSDLDFSVLD

IKEDEALAINCVNTLHSIAAVGNHRDAVISSLRRLKPRIVTVVEEEADLDIGLEGFEFVKGFEECLRWFRVYFEALDESFPRTSNERLMLERAAGRAVVD

LVACSPADSVERREKAARWARRMHGGGGFNTVAFSEEVCDDVRALLRRYREGWAMTQCSDAGIFLTWKEQPVVWASAWRALT

>Glyma07g15950

MQRLMIMDPRLCGPPNEIQLGNQRFENGFFDQSREFGYLQSNLVPTDTPSSSSVWTHEEPSPEDCEFSDGILSYISQILMEEDMEDKTCMRQDSLDLQIA

ERSFYEVIGEKYPSTPLGHPSSVDPDDGSGEHNLSENYGTCSYNDGDLSSIFTNNFLRRNLGELPNQNFRGNSISQSSYSSSNSVKSSVEGPVDSPSSIL

QVPDLNSETQSILLFQKGVEEASKFLPSGNGLFANLDVANFSKLKPRVGSDELPVKVEKDEGESFPAGSKIRKHHHMEEEDVEENRSSKQAAIFSEPTLR

SSMIDIILLHSLGDGKKHFMARREALQTKNDQIVVSNGKSKASNGGKGRSKKQNGKKEVVDLRTLLVLCAQAVAADDYKSAHELLKRIRQHSNPFGDGNQ

RLAHIFADGLEARLAGTGSQIYKGLVSKRTSAADFLKAYHLYLAACPFRKMTAFISNVTIRKSSANSPRLHIIDFGILYGFQWPTLIQRLSLAGGAPKLR

ITGIDFPQPGFRPAERIVETGCRLAAYAESFKVEFEYNAIAKKWETIQLEELKIDRDEYLVVTCFYRCKNVLDESVVVDSPRNKFLSLIRKVNPNIFIHG

ITNGAFNAPFFVTRFREALFHYSSLFDMLETIVPREEWERMLIEKEIFGREALNVIACEGCERVERPETYRQWQARILRAGFLQQPFEREIVKRAIEKVT

TSYHKDFVIDEDSQWLLQGWKGRIIYALSCWKPA

>Glyma13g02840

MDMLIMDDPDFLDFSDHSITTTLSSHEDCNKNNNNLWSPLIDWESLNACENDFQTLIDSVMIDNNDDVLTPEESDQSAAEESESDSTGGDERGLRLLHLL

MAAAEALSSGTESHDLARAILVRLNELVSPTQGTNIERLAAHFSHALHSLLNGTASAHTPPIDTLTAFQLLQDMSPYIKFAHFTANQAILEAVAHEKRVH

IIDYDITEGAQWASLIQALSSAGPPGPHLRITALSRGGGGGGNSSSASGQRSTASVQETGRRLTAFAASVGQPFSFHHSRLDPDETFRPSNLKLVRGEAL

VFNCMLHLPHLNFRASGSVGSFLRGAKELNSRLVVLVEEEMGCVAADSGFVGFFMDSLHHYSAVFDSLEVGFPMQTWARALVEKVFLGPRITGSVARMYG

SGTEEEKVSWGEWLGAAGFRGVPLSFANHCQANLLLGLFNDGYRVEELENNRLVLGWKSRRLLSASVWSSNS

>Glyma18g39920

MIMDPRLLGFSGSPNEIQLGNQRFENGFFDQSREFGYLQSNLLPIDTPSSSSVWTHEEPSPEDCEFSDGILSYISQILMEEDMEDKTCMLQDSLDLQIAE

RSFYEVIGEKYPSSPLGHPSSVDPDDGGGVDNFSENYGTCSYNDGDLSSIFTNNSLRRNLGELPNQNFRGNSISRSSYSSPNSVISSVEGPVDSPSSILQ

VPDLNSETQSILQFQKGVEEASKFLPSGNGLFANLGVANFSKLEPRVGSDELPVKVEKDEGESFPAGSKIRKHHHREEGGVEENRSSKQAAIFSEPTLRS

SMIDIILLHSLGDGKKHFMARREALQTKNEKIVVSNGKSKASNGGKGRSKKQNGKKEVVDLRTLLVLCAQAVAADDYKGANELLKQIRQHSNPFGDGNQR

LAHIFADGLEARLSGTGSQIYKGLVSKRTSAADFLKAYHLYLAACPFRKMTAFISNVTIRKSSANSPRLHIIDFGILYGFQWPTLIQRLSLAGGAPKLRI

TGIDSPQPGFRPAERIVETGRRLAAYAESFKVEFEYNAIAKKWETIQLEELKIDRDEYLVVTCFYRGKNVLDESVVVDSPRNKFLSLIRKINPNIFIHGI

TNGAFNAPFFVTRFREALFHYSSLFDMLEAIVSREEWERMLIEKEIFGREALNVIACEGCERVERPETYRQWQARILRAGFLQQPFEREIVKRAIEKVTT

SYHKDFVIDEDSQWLLQGWKGRIIYALSCWKPA

>Glyma13g42100

MDISPYTPPKKLSSHSTIHSNNHNNIIADEATTLDHNNIMHRSNHSHTSTSRSSDSGELSEDGKWAPKLLRECAKAISERDSTKTHHLLWMLNELASPYG

DCDQKLASYFLQALFCRATESGERCYKTLSSVAEKNHSFDSARRLILKFQEVSPWTTFGHVASNGALLEALEGEPKLHIIDLSSTLCTQWPTLLEALATR

NDETPHLKLTVVAIAGSVMKEVGQRMEKFARLMGVPFEFNVISGLSQITKEGLGVQEDEAIAVNCVGALRRVQVEERENLIRVFKSLGPKVVTVVEEEAD

FCSSRGDFFKCFEECLKFYTLYFEMLKESFPPTSNERLMLERECSRSIVRVLACCGTGHEFEDDHGEFDCCERRERGIQWCERLRNAFSPSGFSDDVVDD

VKALLKRYQSGWSLVVTQGDEHISGIYLTWKEEPVVWASAWKP

>Glyma09g04110

MEDLYHVSFTTDDNSSSSNDILWCTEELNELRKVQFSGAEDHGEYGGIDPLYSNFGFFPDDPSEQEGYLLSTDQQKYHQQVQPSYHDYGQLDDNLQFDMV

SPPLQFDAQYPTMVPLCHTTKDKPNSNSNTPLASLDILNNYGKGFKRLRNEDKTLKQVDDVAMATTNEGIMRKLSTEDVMRIAGTRFIQSSSSESESLPF

LETHPFGIYFSGLSNEEKEDVELAESLLACAEKVGHQQFERASKLLSRCESLSCKTGSPVRRIVHYFAEALRQRIDRATGRVSYKDLQKGPSFDPLEATK

VLNPTVVAFYEELPFCQISVFTEVQVIIEDVAEAKKIHVIDLEIRKGVQWTILMQALESRHECPIELLKITAVESGTTRHIAEDTGERLKDYAQGLNIPF

SYNIVMVSDMLHLGEDVFEIDPEETIVVYSHFALRTKIQESGQLEIMMRVIRILNPSVMVVAEIEANHNSTSFVNRFIEALFFFSTFFDCLETCMKGDEG

NRMIVESLYFSHGIRNIVAAEGAERDSRSVKIDVWRAFFSRFGMVEKELSKLCLFQADLVAKRFPSYSTFDKNGHCLLIGWKGTPINSVSVWKFL

>Glyma11g10170

MMSLSPSLGSPNNLLFREMKSEERGLYLIHLLLTCANHVAAGNLENANTTLEQISLLASPDGDTMQRIATYFMESLADRILKTWPGIHRALNSTRITLLS

DEILVQKLFFELFPFLKVAFVLTNQAIIEAMEGEKVIHIIDLNAAEAAQWIALLQVLSGRPEGPPHLRITGVHQKKEILDQVAHRLTEEAEKLDIPFQFN

PVVSKLENLDFDKLRVKTGEALAISSILQLHTLLAWDDETMQRKSPLLLKTSNGIHLQRVLPMGQSTLGDLVEKDMVNGYTLSPDSTSSSPASLTTSNSM

NMESFLNALWGLSPKVMVVTEQDCNHNGPTLMDRLLEALYSFAALFDCLESTVSRTSLERLRVEKMLFGEEIKNIIACEGSERKERHEKLEKWFQRFDLA

GFGNVPLSYFGMLQARRFLQSYGCEGYRMRDENGCVLICWEDRPMYSISAWRSRK

>Glyma15g03290

MDISPYTTPKKLSPHSNNHNNIIADEAAALLDHNNMHRSNHSHTSTSRSSDSSEPSEDGKWAPKLLRECAKAISERDSSKTHHHLWMLNELASPYGDCDQ

KLASYFLQALFCRATESGERCYKTLSSVAEKNHSFDSAMRLILKFQEVSPWTTFGHVASNGAILEALEGEPKLHIIDLSNTLCTQWPTLLEALATRNDET

PHLKLTVVAIAGSVMKEIGQRMEKFARLMGVPFEFNVISGLSQITKEGLGVQEDEAIAVNCVGTLRRVEIEERENLIRVFKSLGPKVVTVVEEEADFCSS

RENFVKCFEECLKFYTLYFEMLEESFPPTSNERLMLERECSRTIVRVLACCGSGEFEDDGEFDCCERRERGIQWCERLRSAFSPSGFSDDVVDDVKALLK

RYQPGWSLVVSQGDEHLSGIYLTWKEEPVVWASAWKP

>Glyma05g22460

MDTLFRLVSFHQQQQPDPSLNSTTSRTSSSSRSSRQNYYPYSSYQEEEECFNFYMDEEDLSSSSSKYYYPYQPHHQQQQQQQHNVSNISTNTFSTTPNTD

YSYSFSPTPQVQDFNFEFSPNWSHNLLLESARAVADNNSTRLHHLLWMLNELSSPYGDTDQKLAAYFLQALFSRVTEAGDRTYGTLASASEKTCSFESTR

KTVLKFQEVSPWTTFGHVASNGAILEALEGNPKLHILDISNTYCTQWPTLLEALATRSDETPHLRLTTVVTGRTSNSVQRVMKEIGTRMEKFARLMGVPF

KFNVIHHYGDLSEFNFNELDIKEDEALAVNCVNRLHSVSAVGNNRDALISSLQALQPRIVTVVEEEADLDVGIDGYEFVKGFEECLRWFRVYFDALDESF

VKTSNERLMLERAAGRAVVDLVACSTAESVERRETAARWVARLHNGGLKAAPFSEEVCDDVRALLRRYREGWSMAACSDAGIFLSWKDTPVVWASAWRP

>Glyma19g40440

MGYGSTQEDVFQGKESLSGMELLGEIDPLPAENGKGVKLFKYQDQKQLQEPKADNLMLEEVNFDPDVQMQPTQLFQETEGLMKHGVASTEPVEYNKQVAP

TPSLASLELLRNYGSRFKRLSEQNISTLSNIETSFDRQKMSTEEIIRVAGARYIQYSAHWNDSFYIPMHPYGLDLGGLSEEENRDIELAQFLLAAAERVG

CQQFERANGLLLHCEWSSNASANPVQRVIFHFARALRERIYKETGRMTVKGSGKNEERELLQKMDTNIALKCHLKVPFNQVMQFTGIQAIVEHVACETKI

HLIDLEIRSGVQYTALMQALAERRDRIVQLLKITAIGLSSLKTMIEETGKRLASFAESLNLPFSYKTVFVTDIAEIREDHFEIGEDEAVAVYSPYFLRSM

VSRPDCMENLMRVIRNIKPVIMIVLEVEANHNSPSFVNRFIEALFFYSAYFDCLETCIKHEIECRMTIEAVLSEGIRDIVAMEGRERTVRNVKIDVWRRF

FARYRMVETGFSESSLYHAHLVAKGFSFGKFCTIEKNGKCLIVGWKGTPMHSISAWRFL

>Glyma12g02490

MMSLSPSLGSPNNLLFREMKSEERGLYLIHLLLSCANHVAAGNLENANTTLEQISMLASPDGDTMQRIATYFMESLADRILKTWPGIHRALNSTKMTLIS

DEILVQKLFFELFPFLKVAFVLTNQAIIEAMEGEKVIHIIDLNAAEAAQWIALLRVLSAHPEGPPHLRITGVHQKKEILDEVAHRLTEEAEKLDIPFQFN

PVASKLENLDFDKLRVKTGEALAISSILQLHTLLAWDDEAMQRKSPLLLKSSNGIHLQRVLPMGQSTLGDLLEKDMVNGYTPSPDSTSSSPSSLTTSNSM

NMESFLNALWGLSPKVMVVTEQDCNHNGPTLMDRLLEALYSYAALFDCLESTVSRTSLERLRVEKMLFGEEIKNIIACEGSERKERHEKLEKWFQRFDLA

GFGNVPLSYFGMVQARRFLQSYGCEGYRMRDENGCVLICWEDRPMYSISAWRSRK

>Glyma13g38080

MQVTSSQSHMKAELKGTTSISFQNPNTALFNTPHNNPLSGALKGCLGSLDGACIEKLLLHCASALESNDITLAQQVMWVLNNVASPVGDTNQRLTSWFLR

ALISRASRICPTAMSFKGSNTIQRRLMSVTELAGYVDLIPWHRFGYCASNNEIYKAITGFQRVHIVDFSITHCMQWPTFIDGLAKRPEGPPSLRITVPSC

RPHVPPLVNISIHEVGLRLGNFAKFRDVPFEFNVIGNTEGPLTPAELSDESTSFHFEAMLSLLNPTMLNLREDEALVINCQNWLRYLSDDRKGISCQSFS

LRDAFLNLIKGLNPRIVLLVDEDCDLSASSLTSRITTCFNHLWIPFDALETFLPKDSCQRSEFESDIGQKIENIIGYEGHQRIERLESGVQMSQRMKNVG

YLSVPFCDETVREVKGLLDEHASGWGMKREEGMLVLTWKGNSCVFATAWVPCEILRDHHIGMDASLS

>Glyma12g32350

MHVTSSQSHMKAELKGTTSISFQNPTTTLFNNPLSGALKGCLGSLDGACIEKLLLHCASALESNDVTLAQQVMWVLNNVASPVGDTNQRLTSWFLRALIS

RASRICPTAMSFKGSNTIQRRLMSVTELAGYVDLIPWHRFGYCASNNEIYKAITGFQRVHIVDFSITHCMQWPTFIDALAKRPEGPPSLRITVPSCRPHV

PPLVNISIHEVGLRLGNFAKFRDVPFEFNVIGNTGPLTTAELSDESTNFHFEAMLSLLNPTMLNLREDEALVINCQNWLRYLSDDRKGISRQSLSLRDAF

LNIIKGLNPRIVLLVDEDCDLSASSLTSRITTCFNHMWIPFDALETFLPKDSCQRSEFESDIGQKIENIISYEGHQRIERSESGVQMSQRMKNVGYLSVP

FCDETVREIKGLLDEHASGWGMKREEGMLVLTWKGNSCVFATAWVPCEMRDHIGIDAGLS

>Glyma09g35876

MNWDDDERIHLINLLNDCVRLTELGNFNDADIALYHLSQLASSDGDSMQRVATYFIEALAYCQVVKNLRGVPKVLHLVKTLSTPEQQLVKKLFFDFYPFI

KIAHTITNQAIIEAMKGETSINVLDLSPSYNALQWTNLMKCLLKPNTPTCLKITITAIHEKKEVLEQMGLHLGVEAERLHFLISNSILFLENLDPEKLPI

KKGEPLAISSVLQLHSLLATDDDNEMVKMRRGTGQRMFPEMLAKPKKKKVVNPSPDSALSPFSPCPSHKMESFLYGLWKLQPKVMVITEQESNVNNGSSL

TKRVRSALKFYSTLFDCLEASTSRTSERRSLMEKMLLGEEIRNIVAGEGVERKERHEKLVTWIPRLELAGFRREPISSNGIRLATKLLQTYVPGYHIHQK

NKCLFIYRHNVPLFSVSAWKF

>Glyma03g37851

MEFLGEIDPLPTEDGKGAKLFNDQDQKQLQEPKIDNFMLEEVNFNPDMQMQPTQLFQETTGLMKHVRVNSELVEYNKQVPPTPSLASLELLRNYGSRFKR

LSEQNISTLSNIETSFDRQKMSTEEIIRVAGARYLQYSAHWNDSFYIPMHPYGLDLVGFSEEENRDIELAQFLLAAAERVGCQQFERANGLLLHCEWSSS

GSASPVQRVIFHFARALRERIYKETGRMTVKGSGKNEERELIQKMDTNISIKCHLKIPFNQVMQFAGVQAIVEHVASETKIHLIDLEIRSGVQCTALMQA

LSERRDCIVQLLKITAIGLNSLKIKIEETGKSLTSFAESLNLPFSYNAVFVADIAEIRKDHFEIGEDEAVAVYSPYFLRSMVSRPDCMENLMRIIRNIKP

VIMIVLEVEANHNSPSLVNRFIEALFFYSAYFDCLETCIKHEIECKMTIEAVLSEGIRDIVAMEGRERTVRNVKIDVWRRFFARYRMVETGFSESSLYHA

HLVAKGFAFGKFCTIEKNGKGLVVGWKGTPMHSISAWRFL

>Glyma08g15530

MKTMDFEQFYYSFGPPYMNQLHECISENAFPFQTENLLSPNTFLDEMFDQEYSMEGLLQQHANNQEDFGFLKHDDPLETEFCHGFSPSAEENMHVSMEEG

DSCLKGIQAELMEETSLADLLLTGAEAVEAQNWPLASDIIEKLNNASSLENGDGLLNRLALFFTQSLYYKSTNAPELLQCGAVSTHTNAFCVFQVLQELS

PYVKFAHFTANQAILEATEGAEDLHIIDFDIMEGIQWPPLMVDLAMKKSVNSLRVTAITVNQRGADSVQQTGRRLKEFAASINFPFMFDQLMMEREEDFQ

GIELGQTLIVNCMIHQWMPNRSFSLVKTFLDGVTKLSPRLVVLVEEELFNFPRLKSMSFVEFFCEALHHYTALCDSLASNLWGSHKMELSLIEKEVIGLR

ILDSVRQFPCERKERMVWEEGFYSLKGFKRVPMSTCNISQAKFLVSLFGGGYWVQYEKGRLALCWKSRPLTVASIWEPMAYLDDKVK

>Glyma11g01850

MESMFQEEGSSSVTSSSSLQFFSMMSLSLSPSLGSSPYQMKCEERGLVLIHLLLAGANFVATGDLQNANLTLEQISQHASLDGDTMQRIASYFSEALADR

ILRTWPGIHRALNSNRIPMVSDEILVQKLFFELLPFLKFSYILTNQAIVEAMEGEKMVHVIDLNAAGPAQWIALLQVLSARSEGPPHLKITGVHHQKEVL

DQMAHKLTEEAEKLDIPFQFNPVLSKLENLDFEKLGVKTGEALAISSIMQLHSLLALDDDASRRKLPLLSKNSNAIHLQKGLLTNQNTLGDLLDGYSPSP

DSASASASSSPASSSASMNSESFLNALWGLSPKVMVVTEQDFNHNCLTMMERLAEALFSYAAYFDCLESTVSRASLDRIKLEKMLFGEEIKNIIACEGCE

RKKRHERMDRWIQRLDFSGFANVPISYYGMLQGRRFLQTYGCEGYKMKEECGRVMMCWQERPLFFITAWTPRK

>Glyma17g17400

MDTLFRLVSFHQQQQPQQQPDPSLNSTTSRTSSSSRSSRQNYYPYSHQEEEECFNFYMDEEDLSSSSSKHYYHPYQPHHHQQHNVSNISTNTFSTTPNTD

YSYSYSFSPTQPVQDFNFEFSSPNWSHNLLLESARAVADNNSTRLHHLLWMLNELSSPYGDTEQKLAAYFLRALFSRVTEAGDRTYRSLASASEKTCSFE

STRKTVLKFQEVSPWTTFGHVASNGAILEALEGNSKLHILDISNTYCTQWPMLLEALATRSEETPHLCLTTIVTGSRIGNNVQRVMKEIGTRMEKFARLM

GVPFKFNVVHHYGDLSEFNFSELDIKDDEALAVNCVNSLHSVSALGNNRDALISALQALQPRIVTVVEEEADLDVGIDGYEFVKGFEESLRWFRVYFEAL

DESFVKTSNERLMLERAAGRAVVDLVACSPADSVERRETAARWAARLHNGGLNAAPFSDEVCDDVRALLRRYKEGWSMAACSDAGIFLSWKDTPVVWASA

WRP

>Glyma02g01530

MESLRGRNNLPTTSEQNQKQQHDLNFDPLMIEEFNFEPIQQPKQPLQETKEPKKNKQVLPSSLASLELLSNYGSSDSSVQTCVGPQKLSTEEIMRLAGAR

YIQHSTQLCDDVCIPVHPYGFGLGVLSQEENRDIELAQFLLAAAERVGCQQFERASILLSSHFQWNSSGDGAVQRVVFHFAQALLERIRRETGGKVTLNK

CEKNCEREMFEKLRSDTNMAVTCHQKIPFNQEMQFSGVQAIVENVTSKTKVHLINFDIGCGVQCTALMQALAERQEKQVELLKVTAIGLQGKTELEETGK

GLVVFVTSIIEIKVEQFGIEDNEAVAVYSPYMLRTMVSDSDSLEHLMRVMRKIRPSIMVVLEVEAMHNSPSCVNRFIEALFFYAAFFDCIGTCMKQDHEC

RIRIEGILSEGIRNIVAMEDGERKVRNVKIDVWRRFFARYRMVETTFSESSLYQANLVAKKFACGNFCTVDRNGKCLIVGWKGTPIHSISVWKFL

>Glyma20g30150

MSSPRFPGGGASDFYGGAGGFPSQFTAVQPTMNNHSATTPRPLYRSQPSILLNPSSHIAQHQPSPLIGKRTLAEFQTHNLNSSNNNNNNPLLSNYLLRSV

KPRTFQHTELSTFPSNRYGLPLLHHLRPNAVNAQQQQPVTNSILPNTNYFPPVRSRLTAPHELEKNSIDRRLQELEKQLLEDNEDEQGDAVSVITNTTTT

SEWSHTIQNLITPQKPTSSSPTSSTTSSNSSVESTSSKQSLTEAAIAISEGRFDTATEILTRLLQNSDQRFVNCMVSALKSRMNHVECPPPVAELFSIEH

AESTQLLFEHSLFFKVARMVANIAILESALTENGKLCVLDFDIGDGNQYVSLLHELSARRKGAPSAVKIVAVAENGADERLNSVGLLLGRHAEKLGIGFE

FKVLIRRIAELTRESLDCDADEALAVNFAYKLYRMPDESVSTENPRDELLRRVKALAPRVVTLVEQEANANTAPFVARVSELCAYYGALFDSLESTMARE

NSARVRIEEGLSRKVGNSVACEGRNRVERCEVFGKWRARMSMAGFRLKPLSQRVAESIKARLGGAGNRVAVKVENGGICFGWMGRTLTVASAWC

>Glyma03g03760

MKVMPLPFEEFQGKGVLDFSSASDSFSVLLHNPQPKWTIDKEDYCYVGSITEPTSVLGSRRSLSPPTSSSTMSSSLGSSNNSTSKGGGGTSANTTNNPTP

PSDYNNNNPPQESSPEKCGIRMEDWECQDQSILRLIMGDVEDPSAGLSKLLQSTACGSQSADFNAGFGVVDQQGLNMNMNMNMVGGNIDPNYPAGFPFIA

ENMDGQNAKAGSGTGQVSESVVFSANNPLMVSSSVSPGVFTSQQQQQEFGVVDEKPQVQEQQVFSQHQAKHHLFDTIGHNFQAPRLSLLDSGQEVFGRRQ

QTQLPLFPHHMQQQQQQQSMVMPSTKQQKVSSTGDDASHQLQQAIFDQLYKTAELIEAGNPVHAQGILARLNHQLSPIGRPFQRAAFYMKEALMSLLHSN

AHSFMAFSPISFIFKIGAYKSFSEISPVLQFANFTCNQALIEAVERSDRIHVIDFDIGFGVQWSSFMQEIALRSSGAPSLKVTAIVSPSTCDEVELNFTR

ENLIQYAKDINVSFEFNVLSIESLNSPSCPLLGKFFDNEAIVVNMPVSSFTNYPSLFPSVLHFVKQLRPKVVVTLDRICDQMDVPLPTNVVHVLQCYSAL

LESLDAVNVNLDVLQKIERHFIQPAIKKIILGHHHFQEKLPPWRNLFMQSGFSPFTFSNFTEAQAECLVQRAPVRGFHVERKPSSLVLCWQKKELISVST

WRC

>Glyma01g33270

MKAMPLPFEEFQGKGVLDFSSASDLFSVLLHHPQPKWTTDKADYCYVGSSTEPTSVLDSRRSPSPPTSSSTMSSSLGSSSNNSTSKGGGGSGTSANTTTN

PTPPSGNNNNNPPQESSPEKCGIRMEDWEGQDQSILRLIMGDVEDPSAGLSKLLQSTACGSQSVDFNAGFGVVDQQGLNMNMNMVSSGNIDQNYPAGFPF

IAENIDGQNAKAGSGTGQVSESVVFSGNNPLMVSTSVSPGVFTSQQQQEFGVVDEKPQVINPQFMLNQNQVQFSENPSFFVPLMYPQVQVQEQQVFSPPH

QSKRPLFDTIGHNFQAPRLPLLDSGQEVFGRRQQTQLPLFPHHMQQQQQQQQSMGLPSAKQQKVSSTGDDASHQFQQAIFDQLYKTAELIEAGNPVHAQG

ILARLNHQLSPIGKPFQRAAFYMKEALMSLLHSNAHSFMAFSPISFIFKIGAYKSFSEISPVLQFANFTCNQALIEAVERFDRIHVIDFDIGFGVQWSSF

MQELALRSSGAPSLKVTAIVSPSTCDEVELNFTRENLIQYAKDINVSFELNVFSIESLNSASCPLLGQFFDNEAIAVNMPVSSFTNYPSLFPSVLHFVKQ

LRPKVVVTLDRICDRIDVPLPTNVVHVLQCYSALLESLDAVNVNLDALQKIERHFIQPAIKKIILGHHHSQEKLPPWRNLFIQSGFSPFTFSNFTEAQAE

CLVQRAPVRGFHVERKPSSLVLCWQRKELISVSTWRC

>Glyma10g37640

MSSPRFPGGGASDFYGGAGGFPSQSTALQPTMNNHSATTHHPLYRSQPSIFLNPSSHIAQHQTPTLIGKRTLAEFQTHNHSLNLINNNDNLLLSNHQLRS

VKPRTFQHTELSTFPSRRYGLPLLHHLRPNAVNAQQQPVTNSILANTNYLSPVHSRLTATHEPEKNLIALRLQELEKQLLEDNEDDAVSVITTTTSEWSH

TIQNLITPQKPASSSPTSSTTSSNTSVESSSYKQSLTEAATAISEGKFDAATEILTRLSLNSDQRFVNCMVSALKSRMNHVEYPPPVAELFGTEHAESTQ

LLFEYSLFFKVARMVANIAILESALTESGKLCVVDFDICDENQYVSLLHELSARRKGAPAAVKIVVVTENCADDERLNIVGVLLGRHAEKLGIGFEFKVL

TRRIAELTRESLGCDADEPLAVNFAYKLYRMPDESVSTENPRDKLLRRVKTLAPRVVTLVEQDANANTAPFVARVTELCAYYGALFDSLESTMARENLKR

VRIEEGLSRKVVNSVACEGRDRVERCEVFGKWRARMSMAGFRLKPLSQRVADSIKARLGGAGNRVAVKVENGGICFGWMGRTLTVASAWC

>Glyma05g22140

MTQFTPSLPPLHHQITPLLQNPNIMNESQFPRPGPWASACFPTTSKALSNFGDANCMEQLLVHCANAIETNDVTLAQQILWVLNNIAPPDGDSNQRLASG

FLRALTARAAKTGTCKMLVPAGGTNLSIDTHRFNVIELANFVDLTPWHRFGFTAANAAILEATEGFSVIHIVDLSLTHCMQIPTLVDAIASRNYHEVPPP

IIKLTVAADASFRDNIPPMLDLSYDELGAKLVNFARSRNMVMEFRVVSSSYRDGFAGLIEHLRVQQQHFVYAAESRTTPSEALVINCHMMLHYIPDETLS

DTTGLTSFLYDSSSLAASSAVSVTPTSSLRSLFLKSLRGLDPTVVILVDEDADLTSNNLVCRLRSAFNYLWIPYDTVDTFLPRGSKQRQWYEADICWKIE

NVIAHEGVQRVERVEPKNRWEQRMKNASFQGVAFSEDSVAEVKAMLDEHAAGWGLKKEDEHIVLTWKGHNVVFASAWLPA

>Glyma01g18040

MKAVPLPFQEFRGNGVLDFASVAAVSDSLLLPQQEQFLQRWNPQRENFCYVGIEPAVSGLDLKRKTSSPPTSSSTLSSSRASSGGCGGWGSADSTTGAAT

ATVAEKENNPPQGGLEVVGQARCGLGMEDWESVLSESPGEDHSILKLIMGDIEDPSVGLTKLLQGGSASQDVELNGLGVGFGLVNQSSVLDPIPSVNFVS

SSIDPSGPGNCADFPFNSHSNVSPNVPSVGSGLNPNPTGFPTSASNLSQVSLPQGVFQPQQQQHQPIEPLDEKLQVLNPQFILYQNQSQFMPNPGLVLPL

TYAQLQEHHQLLPQPPAKRLNCGPNYQVPKTPFLDSGQELLLRRQQQQLQLLPHHLLQRPSMVVAPKQKMVNSGSEDLATHQLQQAITEQLFKAAELIDA

GNLELAHGILARLNHQLSPIGKPFQRAAFYFKEALQLLLHPNANNSSFTFSPTGLLLKIGAYKSFSEISPVLQFANFTCNQALLEAVEGFDRIHIIDFDI

GLGGQWSSFMQELALRNGSAPELKITAFVSPSHHDEIELSFSQESLKQYAGELHMSFELEILSLESLNSASWPQPLRDCEAVVVNMPIGSFSNYPSYLPL

VLRFVKQLMPKIVVTLDRSCDRTDAPFPQHLIFALQSYSGLLESLDAVNVHPDVLQMIEKYYLQPSMEKLVLGRHGLQERALPWKNLLLSSGFSPLTFSN

FTESQAECLVQRTPSKGFHVEKRQSSLVLCWQRKDLISVSTWRC

>Glyma11g17490

MNEMKAVPLPYQEFRGNGVLDFASGAAAAVSDSLLLPQQEQFLQRWNPQRENFCYVGIEPSSGLDRKRKTSSPPTSSSTLSSSRASSGSTDSTTGTATAT

AAEKENNPPQAGLEVGQARCGLGMEDWESVLSESPGQDHSILKLIMGDIEDPSVGLTKLLQGGSGSQDVEFNGVGVGFGLVDQSSVLFPIPSVNFVSSSS

SIDPSGTGNCSDFPFNSQTNVSPNVPRVGSGVNPNTTGFPASASNLSPVSLPQGVFLPQQQQQHHPPIEPLDEKLQVLNPQFILNQNQSQFMPNAGLVLP

LTYGQLQENHQLLPQPPAKRLNCGPNYQVPKTPFLDSGQELLLRRQQQQLQLLPHHLLQRPSMVVAPKQKMVNSGGQDLATHQLQQAITEQLFKAAELID

AGNLELAHGILARLNHQLSPIGKPFQRAAFYFKEALQLLLHSNANNSSFTFSPTGLLLKIGAYKSFSEISPVLQFANFTCNQALLEAVKGFDRIHIIDFD

IGLGGQWSSFMQELALRNGGAPELKITAFVSPSHHDEIELSFTQESLKQYAGELRMPFELEILSLESLNSASWPQPLRDCKAVVVNMPIGSFSNYPSYLP

LVLRFVKQLMPKIVVTLDRSCDRTDAPFPQHLIFALQSYSGLLESLDAVNVHPDVLQMIEKYYLQPSMEKLVLGRHGLQERALPWKNLLLSSGFSPLTFS

NFTESQAECLVQRTPSKGFHVEKRQSSLVLCWQRKDLISVSTWRC

>Glyma16g29900

MSSSGFPGGGGASEFFAGVGGFGGRSIPATSMNNPNAAASATNNNLHPLYRTQQQQQQQQQQNLPAMFLDPSSQIAQRQTPTLIGKRTLTEFQAYNQTNN

NPNHVLSNLLLRTVKPRTTFSHNHMDFPVPELQSQNLYANQTQRFGVPLLHQLRPQPINLPNNGPVPMTGPNFGYRNSNLGFPPNQNRVRVSPPVSVPVQ

VQSSEPEKKIMDHRLLELEKQLLEDNDEEGEADAASVITTSEWSETYQNLISPGPVQKPVLTTTSPTSSTTSSTSSSSSVASPASGCSKQTLMEAASAIV

EGKHDVAAEILNRLNGVNRSDRLTDCMVSALKSRMNPGEHPPPVAELFRKEHAESSQLLLDNSVCFKVGFMAANYAILEAAFEEKTENNRFCVVDFEIGK

GKQYLHLLNALSARDQNAVVKIAAVAENGGEERVRAVGDMLSLLAEKLRIRFEFKIVATQKITELTRESLGCEVDEVLMVNFAFNLNKIPDESVSTENPR

DELLRRVKRLAPRVVTIVEQEINANTAPFLARVAETLSYYSALLESIEATTAGRENNNNNLDRVRLEEGLSRKLHNSVACEGRDRVERCEVFGKWRARMS

MAGFELKPLSQSMAESIKSRLTTANNRVNSGLTVKEENGGICFGWMGRTLTVASAWR

>Glyma02g06530

MRVPSSPQASNNHVLHTNNNPNTFTYEPTSVLDLCRSPSPEKKLTVPKPEPQHNNQLDLDDHVLPNSDWWESIMKDLTLPEDSPTPLLKTNPSCIPDFPP

SSQDPSFDHPTPDFTSLSEIYNQNIPFNYPSNTLEPSFHDLNHSLHPHNNNWDFIEEFIRAADCYDSSHFQLAQAILERLNNRLLRSPMGKPLHRAAFHF

KDALQSILSGSNRNGNGSNLLSSMAEIVQTIKTYKAFSGISPIPMFSIFTTNQALLETLNGSSFVHVIDFEIGLGIQYASLMKEIAEKAGPGTAPLLRIT

AVVPEEYAVESRLVRQNLNQFAQDLGISAQVDFVPLRTFETVSFKAVRFIDGEKIAVLLSPTIFSRLGGNGGSVGAFLADVRRMAPGVVVFVDGEGWTEA

AAAASFRRGVVSSLEFYSMMLESLDASVASGGGGEWVRRIEMLLLRPKIFAAVEGARRRTPPWREAFYGAGMRPVQLSQFADYQAECLLAKVQIRGFHVD

KRHAELVLCWHERAMVSTSAWRC

>Glyma07g04430

MIMEPNPTPDHSLDWFEGSVSYFPPFLDYPYNSSDIQEYQLWEQDIASNYQTDTNTSSPNATNIVTITTPLETCDSNNLPLSDLPKKRSATDESSPKPPQ

NHKHKKIKSKPRNNEADNGDAEATTTTTATVRKSGGNKKGGAKANGNNCNNKDGRWAEQLLNPCAAAITGGNLNRVQHLLYVLHELASPTGDANHRLAAH

GLKALTQHLSSSPTSTSSGSITFASAEPRFFQKTLLKFYEVSPWFSFPNNIANASILQVLGEDTDNSRTLHILDIGVSHGMQWPTFLEALSRRAGGPPPL

VRLTVVTASSSTENDTPFCIGPPGDNFSSRLLGFAQSMNVNLQINKLDNCPLHSLNAQSVDASPDEIFVVCAQFRLHQLNHNAPDERSKFLTVLRNMEPK

GVILSDNNLGCCCNCCGDFATGFSRRVEYLWRFLDSTSSAFKGRESEERRVMEGEAAKALTNQRETNEGKEKWCERMKEAGFVEEVFGEDAIDGGRALLR

KYESNWEMKVEDDNRSVGLWWKGQSVSFCSLWKLDGNDQSST

>Glyma17g17710

MTQFTPPLPPLHHQITSLLPNPNMMNKNQIPRPRPWPASGFPTTSKALSNLGNANCMEQLLVHCANAIETNDVTLAQQILWVLNNIAPHDGDSNQRLASG

FLRALTARAAKTGTCKMLVSAGTNLSIDTHRFNIIELANFVDLTPWHRFGFTAANAAVLEATEGFSVVHIVDLSLTHCMQIPTLVDAIASRQHHDAPPPI

IKLTVADACCRDHIPPMLDLSYEELGAKLVSFARSRNVIMEFRVVSSSYQDGFASLIEHLRVQQEQQQQQQQQHFVYAAEPSTPSEALVINCHMMLHYIP

DETLSDTTDLTSYVYDSSSSAAVSVTPTSSLRSLFLKSLRGLDPTVVILVDEDADLTSNNLVCRLRSAFNFLWIPYDTVDTFLPRGSKQRQWYEADICWK

IENVIAHEGLQRVERVEPKNKWEERMKNASFQGVGFSEDSVAEVKAMLDEHAAGWGLKKEDEHIVLTWKGHNVVFASAWLPA

>Glyma16g01020

MIMEPNPTSDHSLDWFEGSVSYFPPLLDYPYDSGDIQDYQLWEQDITSHYQTDTNTSSSPNATNISTTTTPLETCDSNNNLPLSDLPKKRNATDESSPKP

PQNHKHKKIKSKPINNEAYTGHAEGTTTTTVRKSGGNKKGGAKANGNNCNNKDGRWAEQLLNPCAAAITGGNLNRVQHLSYVLHELASPTGDANHRLAAH

GLKALTQHLSSSPSSGSITFASSEPRFFQKTLLKFYEVSPWFSFPNNIANASILQVLGEDTDNNSRTLHILDIGVSHGMQWPTFLEALSRRPGGPPPLVR

LTVVTASSSTENDTPFCIGPPGDNFSSRLLGFAQSMNVNLQINKLDNCPLHTLNAQSVDTSPDEIFVVCAQFRLHQLNHNAPDERSEFLKVLRNMEPKGV

ILSDNNMGCCCNCCGDFTTGFSRRVEYLWRFLDSTSSAFKGRESDERRVMEGEAAKALTNQRETNEGKEKWCERMKEAGFVGEVFGEDAIDGGRALLRKY

DGNWEMKVEDDNTSVGLWWKGQSVSFCSLWKLDGNYKTSTFTS

>Glyma16g25570

MRVPSSPQPNNNHSPTSNLVLHTNNNPTTTFTYEPTSVLDLCRSPSPEKKLTVPKPEPQHNNNNNNLNLDLDDHVLPNSDWWESIMKDLALPEDSPTPLL

KTNINPSCIPDFPPSSQDPPFDHPQDFTSLSEIYNQNLPYNYPTNTLEHSFYDLSHNHHHNNNNNVNNNNWDFIEELIRAADCFDSSHFQLAQAILERLN

NRLLRSPMGKPLHRAAFHFKDALQSILAGSNRTSSNRLSSMAEIVQTIKTYKAFSGISPIPMFSVFTTNQALLETLNGSSFVHVIDFEIGLGIQYASLMK

EIAEKAGAGASPLLRITAVVPEEYAVESRLVRENLNQFAQDLGISAQVDFVPLRTFETVSFKAVRFVDGEKIAVLLSPAIFSRLGSNGGSVGAFLADVRR

VSPGVVVFVDGEGWTEAAAAASFRRGVVSSLEFYSMMLESLDASVAAGGGGEWVRRIEMMLLRPKIFAAVEGARRRTPPWREAFYDAAMRPVQLSQFADY

QAECLLAKVQIRGFHVDKRHAELVLCWHERVMVATSAWRC

>Glyma01g38360

MRVPVPSSPQANPKPTNNVVRTIALPNLNNSTTPPTGLCYEPTSVLDLCRSPSPGTEKPTTDHSVLVTNSQDYLDLEDHALHNLDWDSIMKDLGLHDDSA

TPVLKTFLHPDDDDDDDNNNNPSCDDFTLFDHALEFTTLSDIYSNQNFAFDFNHLPHDFNHLNGFDFIEELIRAADCFDTKQLHVAQLILERLNQRLRSP

VGKPLHRAAFYLKEALQSLLSGSNRTPRISSLVEIVHSIRTFKAFSGISPIPMFSIFTTNQIVLDHAASSFMHVIDFDIGLGIQYASLMKEIAEKAADSP

VLRITAVVPEEYAVESTLVRDNLAQFALDLRIRVQVEFVPLRTFENLSFKAVKFVNGENTAVLLSPAIFRHLGNAAAFLADVRRISPSVVVFVDGEGWAE

TATASAASFRRGVVSSLEYYSMMLESLDASTVGGGGEWVRRIEMMQLRPKILAAVESAWRRVPPWREAFYGAGMRPVQLSQFADFQAECLLAKSQIRGFH

VAKRQNELVLFWHDRAIVATSAWRC

>Glyma11g06980

MRVPVPSSPQANPKPTNNVVRTIALPNLNTTTTTTTTSLCYEPTSVLDLCRSPSPSPGTEKPTNDHAVLDLDDHALHNLDWDSIMKDLGLHDDSATPVLK

TFLHPDDDNNNPSCDDFTPFDHALEFTSLSDIYSNQNLAFDFNHLPHDFNHHLNGFDFIEELIRAADCFDTKQLHVAQVILERLNQRLRSPVGKPLQRAA

FYFKEALQSLLSGSNRTPRISSLVEIVHSIRTFKAFSGISPIPMFSIFTTNQIVLDHAACSFMHVIDFDIGLGIQYASLMKEIAEKAAESPVLRITAVVP

EEYAVESTLVHDNLAQFALELRIRVQVEFVALRTFENLSFKSVKFVDGENTTVLLSPAIFGHLGNAAAFLADVRRISPSMVVFVDGEGWAETATASAASF

RRGVVSSLEYYSMMLESLDASTVGGGGEWVRRIEMMQLGPKILAAVESAWRKLPPWREAFYGAGMRPVQLSQFADFQAECLLAKSQIRGFHVARRQNELV

LFWHDRAMVATSAWRC

>Glyma18g43580

MMQSEDLQFQWPFFEDMNSTFDQGVESYGFTTMYGHVGDCGPYDFTMNDHVGDCGLNTLLSTPEDSTSEICSIPFPSSPIFSIDHIHIQYPINEETMHLP

SLMELDDFDSILDTQIISIQGHGESEGSFFPSQNFSSEVENAWSPTPSVRSELSTNQTSPLTLPLENMEVENQVSLPHLLKAYGEALEQGQKALEEVILR

CISQKASPLGESLERLAFYLSQGMTNHGDYLKGEALKNFEAALRALYQGFPIGKIAHFAAVSAILEALPQDCDVHIVDFYIGHGVQWPPMIEAIAHMNKT

LTLTSIKWGGEVPECVSSPCNFEETRRQLYEHAKSCGLKLKVEEKGVEELVSDIKKMNKKGEKGEFLAFNCTIDLPHMGKVRSRKHALQFLRVADELIST

SDNRGIITFADGDAFEKVKNNLNFRSFFDGHLVHYQALLESMESHFPTSFSEARIAMEKLFLQPCISSLDWLQTWEEMKRGGHLEEETSLEGCQLSKNIL

MEIREVLRGSDGSYQARIEGQHDNELVLEYKGTQLLRFSTWKN

>Glyma07g18934

MMQSEDLQFQWPFFEDMNSTFDQGVESYGFTTMDDHVGNCEPYGFTMDDHVGNCEPYGFTMDDHVGDCGLSTLLSTPGDSTSEICSIPFPSSPMFSNDHI

HIQYPINEETMDLPSLMELDDFYSILDTQIISIQGHGESEGSFFPSQNLSSEVENAWSPTPSVMSELSTNQTSPLTLPLENMEIEKQVSLPHLLKAYGEA

LEQGQKALEEVTLRRISQKASPLGESLERFAFYLSQGMTNHGDYLKGEAFKNFGITLRALYQGFPIGKIAHFAAVSTILEAMPQDYDVVHIVDFCIGHGV

QWPPMIEAIAHMHKTLKLTTIKWGGEGSECVSSPCNFDETRRQLYEHAKSCGLKLKVEEKGVEELVTEIKKMNKKGGRREFLAFNCMIDLPHMGKVRSRK

NALQFLRVAEELINTSGNRGIITFGDGGAFEKVKNNLNFWSFFYGHLVHYQILLESMESHFPTRFSEVRIAMEQLFLQPCISSLDWLQTWEEMKRGDHLE

AETSLEGCQLSKNILMEIREVLRESEGSYQAKIEGQHDNELVLEYKGTQLLRFSTWKN

>Glyma03g06530

MMHPEILESSSWSSIYEVNSSTIGHQFEHYGFEVDAHVGVSVFNSLFNTPENTSSETSINTLPSIMFTNEYDHFLHYQIEKDTLQLPKLMDVYGLSMDCD

DFDPILRDHQESEGDWSPTPSLDSDLSSNQKALTLPQQGMEIENQVSLPHMLEALGEAIYQGKKALKEVILRCMRQKVSPLYEPLERVAFYLCQDMETRQ

DDFYLKQEASKNFEAAFKAFYQGLPHGKVAHFVANLAILEALPHDSEVIHIVDFDMGEGSQWPPLIESIATLRKTLKLTAIKRGEEGSEVVSSPWKFEEI

KRVLHEHARSRDLKLMVEEKEMEEVVSELKKINKSVGSGKRDFYAFNCMVGLPHMGRGSSRRHATEFLNLIKSCGSRGIVTFGDARVCEKLENDLEFVSF

FERHLLHYKALLESIESHFPNHFTDARSAMECLFVAPNISSLAWLQKWEEIKEESESYFQADIGLEGLRLSNAILMEVGEMLSGSEQGSYQARIEGQNDN

QLTLEWKGTPLVRVSTWRKIKGQ

>139506

MGMDELLAHAGYNVRASDLTHVAQRIEELDSLLGAAAPADILAQDTVHYNPSDLVSWIEGMLDELVPQQPTATSSSDMESVNEVGVVASHSQIAASTTPR

PASGSSSSTSPHGIPPHAAGGMTSAAAMPTIQESDELSGVRLVHLLLACANAVQRGDLAAAGDMVAQLRILVAHPSSSSSAMARVATQFVEALSRRIQNS

CYNESSDPGNTNNGAMDEILHFHFYETCPYLKFAHFTANQAILEALEGHKSVHVVDLDLQYGLQWPALIQALALRPGGPPTLRLTGIGPPQPHRHDLLHE

IGLKLAQLADSVNVDFAFHGVVAARLNDVQPWMLTVRRGEAVAVNSVFQMHKALVEEPPIDEVLRLVRNLKPKIVTLVEQDADHNSPVFMERFMAALHYY

STMFDSLEACNLAPGSVEQMVAETYLGQEIGNIVACEGAARTERHETLTQWRIRMARSGFQPLYLGSNAFKQANMLLTLFSGDGYRVEEKDGCLTLGWHS

RPLVAASAWECC

>122441

MERLESLGVERFDQDAVHSNPSDISGWIDSLIEQQTRGSPLDSQQDNTSSSPSPPSLLISCPHDSSRIATTTTTTNALPLHMIHTQPDDEQQDDQEANGL

KLIHLLFACGACLGREDDKSAKAEEFLDQIRMLLLSMGDSAGAIGRVAAYFVEGLSRRILFGSLPAAQAEEADPAFLESFYRTCPFLKFGHFTANQAMYE

ELEEERSVHIIDFEFGLGVQWPPLIQMLAIRPGGPPSLRLTAIAPDHLQFQVHHTGNRLARFAASIGVDLQFQTVNSIASVLVYPGEALAVNSMLHLHRL

VDDSLDSVLASVRRLSPKIFTLLEQDASHNSPDFDNRFNECLHYYSAIFDSIYQQFGQVEQAVLESEAHLGREIVNILACEGRARVERHERLEQWTRRMS

GMGFKPRHLGSNAYNQAATFLTIFPGGGHTIQETAGCLTLGWQSRTLFAASAWRC

>83811

MCSNDVFNPVQREDVLQDKIELKESISALESDGAVGLEFWRGLQHQQEQQEQQEQQQQRHAQDQSLFEQEQQQGSRAQPAAAQDHHESGDANVGIRLIQL

LLACAEAVACRDVNQAATLLSQLQQMASPRGDSMQRVTSCFVEGLTARLAGLQSISLSGAAYKPAVAPPAARRSQIPEALRDEGFNLVYEFCPYFSFGHF

AANAAILDAFEGESRVHIVDLGMSSALQWPALLQGLASRPGGPPESIRITGVSCDRSDKLFLAGEELSRLAESLELQFEFRAVTQAVESLQRGMLDVRDG

EAMAINSAFQLHCVVKESRRSLKSVLQSIHELSPKILTLVEQDACHNGPFFLGRFIEALHYYSAIFDAVDAILPSDSEERLKIEQYHYAEEIKNIVACEG

PDRVERHERADQWRRRMSRAGFQPKPLKFLGEVKTWLGMYYPSEGYTLVEEKGCIVLGWKGKPIVAASTWRC

>102726

MVALGGGGVSRAGGGDYNSNHHHHLGQQQQQQQQQRHHSNNFPKFEPVDQQLRLVRMLLSCAGAVAIDNLDLARAILVQLRALVVPHGSPMQRLASYVTE

ALVARLSRNTRSSHFQGLIADHSLQQLSSATRSDMLEAFWVFYEYIPIGKFTHLTMNQILLEAADRERAIHVVDFQVWYGAQWPSFLQSLAMRPGGPPVV

RMTAVGSSLRDLQEAGSKLLDCARSLGVPFEYCILRVELEDFHAGMVELRDGEAVLVNSLCQFHRFLKRDLDQFLQGLRSLRPRLVVMAENDADHNSPDF

MHRFMACLHYYSAVFDAFDASLHMPGTLPGRKKLEELIAAQKLRNMIACEGSERVERHESMRAWNARMEGVGFRAVSMSHKAINQASLLLKLYYSDGYTL

TNQEGFLILGWRGMPLNGVGAWI

>113858

MPPPAPILPFRSHQDSFGTIRSFDFQQPIQPAFASWTHGTWHVHPTVPPPLPPAVRGPAWQHRGGIPHGTVPVPPAPASTVAVPGGIPHDGSQDPQVLLV

QLLVMCAHAVAEDNESIAQMILARLRQHTGPEGTPMERLASYFTEALAARIDHSTGSALFKGLLSDKLLESDGSTQASMLEAFSTFYDYLPIGKFDHLTM

NQVILDAVERERAIHILDLQLWFGTQWPALLQALATRPGGPPRVRITAVGSSADDLAATGDKLHECAKTLRVHLVYKALLLPKADKFHAGLVNLHPGEAF

IVNSLSQFHYLLQPSTSDSDTSFGGFMAHIRALRPKVLVMAENDASHNSSDFLKRFGECLKYYSAVFDAMATCASSPSGRLKMERLFAAPKIRNIIACEG

PNRVERHESMADWSKRLEVAGFRPSPLSQRAVNQAKLLLRLYYTNGYTLHSERGSLVLGWRNLPLNTVSAWRVA

>88625

MERSGSPASDDAFLIAAGSFDRHHSAFDREQGLLRDDGDEQQQWEALVEGVLDCSADAQSESIGGQGYLDAQHQDDDDHRAAENATSDSNKEEKIELVDL

LVACAQAISAKSTSLIHCLLARLGELASPHGSTAMERLAAYFTEGLACRLASQRPDLYKPLSLETDPSPGSACSSEAEEESIAAYHILNHVSPIVKFAHF

SANDAILEAFQGRKKVHVIDLDVGQGLQWPALFQALANRSEGPPSLVRISGIGPFKDSVQETGDRLAEFAQALGLCFEFHAVVERLEEIRLWMLHVKDGE

AVAVNCIGQLHRSLLDRQQIQGVMELIRSTKPEVVAIVEHEAEHNVECFEARFAGSLRYYAAMFDALDSSVVVVDGESSLSARTRVEKTIFAREIRNIVG

CEGEDRIERHERFEGWKRMLEEEGFRNRGMSQRAIVQAKLLLEMFLCPEYRIDKLEGKDENGSRECCEGITLGWLDQPLVTVSAWSLIR

>77165

MGDPRQLLLLCAESIANGDFALAEVVISRLNQVVCIYGQPMERLAAYMVEGLVARIQSSGTGLCRALRCKEPVGNEILSAMQVMYEVCPYIKFGYMAANG

AIAEALKDEPRVHIIDFEIAQGTQYIALIQALARRPGGPPTVRITGVGDPAAGVAAPGGVAAVGRRLAVLAADHGVPLEFHAVPLSGAGVTDAAALQRRP

GEALAVNFAMQLHHMPDESVSVSNPRDRLLRMAKSLGPKIVTLVEQEANTNTAPFLARFKESLSYYGAVFESLDVTLPRQSKERISVEQHCLARDLVNLI

ACEGAERIERHEVMGKWRARMSMAGFKQYPLSRYVNQTISCLLKTYCDKYKLSEEDGVIYLGWLDRSLVSASAWN

>85562

MLTSSSSSVGAAHIQTTDALRPDVLKLSLDSSSSSPSENTNFSAAAAANQFELHRNLQDQGNVIKPPAPTVAAPSSSTAAATSDDEGLQLLALLLQCAEA

VSADNFEEANALLPQLSELTSPYGNSVERMAAYFSEAMNARMVNSCLGVYAPLIPEMHKVSSKNTIAAFQVFNSLCPLVKFSHFTANQAILEALDGEDSV

HILDLDVMQGLQWPALFHILASRPRGPPRVRLTGLGACSDTLEQTGKRLSEFAASLGLPFEFHGVADKIGNLDPLKLGVRRNEALAVHCLHHSLYDITGS

DVKALALLRQLRPKIITTVEQDLSHSGSFLHRFVEALHYYSALFDSLGASLPEDNTERHVVEQQLLSCEIKNILAVGGPARTGEEKFGSWREEFQGAGFR

AVALGGNASAQASLLLGMFPCEGFALVEDGELLKLAWKDMCLLTASAWSSS

>84991

MATSKLILDHLAASSRDHPPHLSSPIERVSTHICKALSERITKTSIFDATTSDDLAFARRAFYQHFPFLKFAHFTANQAILESLRGCSKLHIVDLDIDQG

MQWPSLIQALSQIENAPSLRITGVGSSLAELQSTGRRLTEFATSIGYHKLDYHPVRLDSPDQLDPSAFSLGDDDDQDLGLAVNCSMFLHRLLGNHPALER

TLCMIRAWNPRIVTVSEMEANHNTPSFVDRFVEALHFYSAVFDCLESALARTDPDRIYIEGAMFAGEIRSILACEGADRIVRHARSESWRDFMRWSGFKD

VGLSDHSLYQAHVFLTLYSQAYRLTREEQALILGWHDTPVVSISTWSC

>84762

MNEIRSMDEGTVAVWLETMVSDISHSFPSMPAEHIWQSLLENLSPCNPHVAPVIESRIISLRHHHEMLHGHHSLKQQQQNQLKHHGHSVNPAAAAQQHKN

HAKQQLPSPTNSSKDKSSESQDQPSGKDDQAGSGGGAATATTTATSAATIAGTSDDAGLQLLALLLQCAEAISTDNFEEANLIQPQLTELASPYGSSVQR

VAAYFAEAMAARMVNSCLGICSALPGIHHVYNHSIAAAFQIFNGMCPLVKFSHFTANQAILEAFEGEQSVHIVDIDIMQGLQWPALFHILASRPGGPPNV

RITGLGTSAEALEATGKRLSDFASSLGLPFEFFAVADKIGHCDAATLKVRQGDALAVHWLHHSLYDVTGSDSKTLKLLGSLEPKVVTMVEQDLSHAGSFL

NRFVEALHYYSALFDSLGASFPEDSPDRHMVEQQLLSCEIKNILAVGGPARTGEVKFEQWRDQLKQSGFRPISLAGNAATQATLLLGMFPLQGYTLVEDN

GTLKLGWKDLCLLTASAWRHP

>96442

MLDDHERSFPDPQQVAVNPVSPPQYQQQQEIERSLVDFRHHSHDERVDFVYPELGVQVHPWDEDMDSIRLVHLLLGAAEATVCGETDLAIAIIDRLKSCC

STQSGTTMQRIAAYFRDALNCRLHGLKFFSRTESQFDTVGAFHVLHEICPYIKFGHFSANQAILESVAGEQRVHIFDFDITDGVQWPSLMQSLALRAGGP

PQLKITALYRPNSKGALSTTQETGKRLAACARQFNVPFVFNQVRVDGESEEFLSSSLKLIQGEALVVNCMLHLPHMSCHSRDAVRFFLGKMAALRPRVLA

IVEEDLSCTSTTFTGRFHEALYHYSTLFDSLEATLASEDEMRSLVERVFLGPRIKNTVTSAVSRSPLEKEANRWSGLAEAVGFQQRSFSSYNRCQARLLV

GLFQDGHQIQEDEDTMLLCWKSRPLIAASVWSSSSDPA

>84560

MQQQPPSSHQHHHQEQPQFLGHTGGIGTGAGAGTGFGFGVGGAGSEGSSGGAGTHHNDGGLRELLVECAASVTSSDWHRAIRCLVHLSRAASPHGDAVER

LAFYFSAALARCLCSLSTPCASEIRSLLRLNNLHFLLEEDQPPPSFEDDLFYYSGGAEEAYLALNQVTPFIRFSHLSANQAILEAVDNERAVHIVDLGIM

QGLQWPPLMQALPPTLSSSSSSSSTTLTLRITGTGPSISLLEQTGARLRDFARTLHLDFEFDAVCTTSRHVVASLQQHLELRRGEALVVNCMTQLHKLLP

AAHRAALPHALEFMRSLCPRILTVAEKESEHDLSQSFLERFLVTLDHYVAVFDSLEATLPPRSPQRLMIERLVLAKEISGIVLEDGGGDDENLAVVRHQS

FGNWRRDMEAAGFQLVPPSDFAIAQAKLLLRLHYPADGYRLLVENQHGSLFLSWHDKPLVALSTWSC

>74492

MIDSPVGLQWPKPSPKYQHEEDLQQQPPSLLATGKGTYYQVLESVGLPSLDLVEQLVECARAVSSHDVMRANLLVEEIRSKVSPLGTSTQRIVYYFVEAL

VARVSATGNGLFTAMCHARPTAGAMLKSVEYIMERSPFLSVRYFFPNQVILNACRGHQRIHIVDYGACFGFQWPALMQELANTPGGPPYLRITGIDSPLP

GGGSASDVGCMLREYAQSIGLPFKFRAVSKKWENIDAATLLLSDDEVLAVNCMFRQTNLLDESVLAESPRKMWLNRVRSLNPRVFIQGMNNASYNVPFFM

TRFLEALTHFALLFDAIDCCSQPESKERHLLEQEKYGREIVNIVACEGLERVERAETYKQWHSRTQRAKFELLNISDQVFHDTESLMGMYHQSFELHRDQ

GWLLLGWKGQILHAFSGWRPSSS

>83927

MNFDDFQLPGDPELDLQHQEFFGCRLFHLLLAGAEAMFSQALDLAKVILFRLRELTSSCSSSGPVFQRLALYFTEALQSLLDGARITKVASSCSMSYLDS

ITAFQALHEASPYIKFGHYVANQAILEAIGDDKRVHILDYDVTLGIQWPSLMQALALREGGTPHLRITAVYRPHSRHQLANFQETKERLMECAAAFKIPF

SFHQAKVEDDEDSKLVGLKLIKGETLIVNCMLHLLHVPHKSPSSVLSFLKSVQKFSPRLVTFVEEEVVSCLSAPNTVDKFFQALHHYSAMLDSLEASLCE

TTAHILVERAFLATRIKTALIAHHHAHSKVEWSSLLHSAGFHRVSLSRRNICQARLLLGLFKDGYQLKEHHSDEEIEKLLLSWKSRPLIAASAWTCKNKS

SDGSENEQQ

>232175

MGLRSHLLDKLVLCGEAVWSDDFGSALAIMEELREQAGPEGDATQRVVHYFLHALNARMSNTGSRFYSVMCKARPSIAETLKAVQMILKHTPFLGLPHFF

TNQIILEAIKGERKVHIVDFGIMYGLQWPALLQLLAERKEGPPQLRITGVDLPPRALNNHSGRIRETGSRLKRCAQDWGIPFKFRSLSCAWESMEPGLLQ

LKDDEVLIISCSFKQTNLFDGSVIAESPKLQWLTRIRNLHPKVFIQSLASSNFAGPIFLQRFQEALVHHAAVFAAMDACISRMLPERRVIEQDKYGREIM

NIIACEGLDRVERSETHQQWHHLAVKAGLEVIPLSPALFEESKAFARFYNRDLTVNRDGEWMWLGWRDQIIHAYSAWRAAT

>122435

MSSSCCSSDQQQWDISSFTTGPATTTTTTTTGGDEHLAANPQHVSTTTVNLRSQHHQEQQQDRGGALEQLVQLLVSCLDDMESGQASSAMDKLATLKAMA

SSSGNAVEKCAWYFSSGLEARLHRRGGNDHSDGDDDEEEESPSSSPNKAEAIAMAYKTLTDACPYLKFAHLTANQALLEATDGAPKIHIVDYGTMQGVQW

AAFLQAFATWPAKNPSPRSLRITGIPSPHLGSNPAPAMLATQRRLTDFAKLLGVDFQFCPILEPIRDFQPSQSLRTDPDEVVAVNFVLQLAQLPAPALKR

AFSLVQRLNPRIVTVAEYEANNGASLRDQLASNARFYSSVFESLDVALPGDDAQRITAERLFFGREITKSLVEGTNCECPEKQREWQRCIDGAGLWSAAL

SHYTVSQARLLLWLYNKSENFTLLQGPGSLSLGWLGTSIVTVSAWHC

>80549

MRYLKASIVKCCEAIAANATTQAYELVSELRDKSSPTGTTVERLAFYFSEALVARSTGTGSLLYNGLIKSKRPIDEILQLFATVAETSPGFGLPIFFTNQ

TILDETSSAARVHVVDFGIGPGYRWLCLIKDFSERSGGPPHFRVTAVDRPSNSLLYPREDVGAKLGRYASSLGVPFEFHSVVTADWDSIGPSQLMIQPDD

VLIVTSFHKLRELSDDPKRRFLRNIHAMEPKLFLNAAFPPVGFNSPSLVARAREAFEFYAGMFEAIAASLAESRFAGERRFLEQLRGLELLNTLACEGEE

RVERPEGYKQWQELMRGAGFEGYEIKRHVYAGAKKMLATYSNAREYSVGRSGNWILLRRNRQVLIAISNWTC

>142207

MLLEDEGEGEADSAQQQKPSFQGLFDELSAIVSPSESSDGSTEGSDQSSGLEVNLGRTTDIYSSLMVCARAVAANNVAGFYDLARDIRDAVALQSTPLQK

VARFLIDALAARLAGTGPQAYRAISAGISSRMLVADARLPLFAVAVNFANNVILRACAGANKVHIIDYGVHCGRQWPSLIKAFSVRPEGPPQLKITGIDL

VTVPEAFVAGQRLAAFARSNGVQLEYCSIQSNSWESVQPVTLANELLVVNSNMSLKRMRDEWISVNNPRRLLFESIYKMRPKVFVMCVSNASFSSPFFIP

KFDETLKHFTAKMECLDAWLGWDSIGDRDLIEKVFQRAIMSVVACDGLEQLERPDKYRTWDSRAKRAGFQPFLIGEEVYERMKSQWGGYACKKNFGCGKD

ENWMLLGWKDVILCGMSAWQTKAQSLIKF

>113376

MDQSDRIELTQLPALQRGDGRWAASLLIECAAAVVQKDAARVQHFMWMLNELASPYGDFDQRLASCFLQGLFCRITGTGSRQHRVLCSAAERQCLFDPMR

KMMLKFQEMSPWTTFGHVAANGALMEAVEGEFRVHILDVSSTMCTQWPTLLEALATRSDGAPHLRLTSILVSSEEAVVKVMTEVGARLRKFARLMGVPFE

FRLLQQPELELLDVATIQPRAGEALIVNCIHSLHNVSERPPPSSSSSAASPRDLVLNTFRSLNPKLVIIADDEADLISRGDFMSRFVEAVRYYSLFFESV

EESFPRTSNERLMLERIVSRKIVNLLACDEASISERQEKSSQWVMRMRRAGFALAKFSDDVADDARALLKRYKEGWGYTNTDVGLFLTWKEQPTVFATSW

KPF

>444260

MYGMLSGQHHGTSFPVFHQQQQQQQELYETCQREEQICLNGLLSSTATTTTSSSSSGSSHSHSHAAVFLSSSKSLKAALSSNNITGSNSNSSWEVATATA

TATGAAARTGQQLPEREHFHHQQQHYNHNQQLLAIDSTGVISSTTAGGGFAGEPTSVLDLRSPSPNNSRALGSASSSSAIQNNTKKAGASSSSSSDCNNS

TTDAAKVKSRGNHHQEQQQQRQQQQISSSFAQSSSSADLQDELEQAVAAASTGYGLSLFYSGQGIRQMENYGGYEQQQQQQQQQQQQQPPQELLHQQHQG

RSDQWLLQDVVGAQQDELLVHHLHQQQHLLPEKQKLEELESELLGSCPSPGVPPAAPAPDPSIIRWLFEEPFSGSAAAAAGAADVNVIPPPPPWQQQVMM

MVKQEQPQQLSSSSSTSSLPPHLAHLESFTRRTLPLVSSGAPLFHHPHPMQFQDPLALSGSMNPLWQQQQQQHNLQIGLEHQQQHREPFLLGMLQDEQHQ

QHMPNLFGQAPMSFMRASSPRALHPGLFVENPGPSPLQLLQQQQERELQQAGKRQASRIPYVDEHVLLRPQPLPPSLKREQQELQHPSSKVARQSPPNAQ

PGHPLQQHVLSSNSGAFPSRLLLHAAEACESGQDELAQSILARLNDHHHHQVFLQQQEHHQNQQFHKLQPPAYRIGLLFRDILAERLSKRKARSSTPAPA

STDATEVVERIAACKSLYDTSTLLKCAHFIANQAIAEAIESEECVHILDLSEAANSPSMNSSFGGQWSSFVASTLEQKLPRPIKIRITLTSTRQHALYFA

LESLRELARDHPKNAGCAEFEFCGVLTRSGSPLSLSDMGLKVHEVLVVNFVFGLHKLVPVAEALDSIRASHPKIVLLVEQEMEPQPSFIERFREGLDYYT

ALAECVAASEECSLKEADKVEQLVWKPEVEELLAGNLEHQRLEKWNLWMSHAGLCCSRLSDMCEKLALGLFNFEKAGNNKTRGFDLVKDDGSLLLGFQGK

VLASASAWKCC

>424843

MEFLGRDLSDPVSSGENYGYGIDGSSPAAVESPGSSGFTLEELNSLMMDENGGGESAMAGSSMIKELQQLILYDSPSPSSSSSQQQQAVTVTTSASDWSS

LDGLSIKSWTSEMIVGEDQEVSSSSCNTMERSSNQELSSSDSSGSANLRDLTPTHVADRQELRGFSSDRADLVGILQEVIASHLATDNHKRNSQEKPAAT

AINRKKRNSAATSEEEDAVEGLKTVIKIRRQGSETADKSNGHNPRLYSVSPDIMTLLHDCAHAVAVRDVTNANSLAVSIRRQHGASSTAADPARRVAYYF

VESLLARVLGTGTALAAATASNSGGPDVMERVYEFRLHATPSPLVPHFVSTSTIVAAAQGKRFLHIVEIGMARNRFQWSLLLQALAHRRDVPLRRLRVTA

VDSPQHKALLAQLAQRIIFESSRLGLEAEVELEIADAHAWEAVLPRIVPRAAPGEVLVVNALFALDFLEDETAVQARSPRRAVLSALRRMDPAVLVLGIN

HGYRAVPQFSFLERFVQAVTRFNAKFDAALECGSGGAQAAGRAAYEGQFLGPEILNLVACDGSDRLVRTAPCEEWERAMVGAGFVALPLEESTVLRAQAM

MGNYNRNFELDYSDGWIKLAWKKVTTQCVSVWQPRRIILPRY

>Pp1s12_244V6

MRLCSESTLTRAWLSRGTRHEHNEGVFDYLDANHLNDDSMQPEKRLAVCPLLLENNAMAYQYYPGSTRYEATGGALVDGQFRHANFMQPSDLVQHLEQLH

SVLGTVSQDSPNIPAHHTLDAGAQTSNNRTSDLAGWIDGMIDELSFNNAGTMAAPQQRSLTEDSLHQNELEASSSHDSSLDTGSSRLPTLHYQNTPAVGN

NFLATPQNDASQLNANRATGAVLEQQPSPMGEDEDNGVRLVHSLLACAESIQRGNLNLAEQTLRRIQLLSLPPGPMGKVATHFIDALTCRIYGVAFSSGN

NVGSNQSDSLSELLHFHFYETCPYLKFAHFTANQAILEAFAGQKQVHVIDFNLMHGLQWPALIQALALRPGGPPRLRLTGIGPPQSGGSDVLQEIGMKLA

QLAETVKVEFEFRGVVAVKLDDIKPWMLQICHGEAVAVNSVFQLHKLLYSAGSVIPIDEVLRSARALKPKIFTIVEHEANHNQPSFLGRFTEALHYYSTM

FDSLEACSLPSDSSEQVLAEMYLGREINNIVACEDAARVERHENLVQWQMRMLKAGYRPIQLGLNAFKQASMLLTMFSGDGYRVEEKLGCLTLGWHTRPL

ISASAWQCLLHKAHTAVTGPNLFEVVLRWSMNNDGVGVSSQPSVDLSPAGFPERLPHDLRMELVYCWKENSILSFTVKASNLVKNEQLGWKSPLAAEIPL

PVTKSEQDQHFR

>Pp1s175_16V6

MCPGHELTLTYVCLAPWAYDHSHRHLLHKAIPLKLDSQQELHMAYQYSPGGSRWKPTGGTLVDGRLRHDKFTQASDAVQQLEELHTSLGSVSQDSLNIPA

YYTLGSSSQAVSNCSTDLAGWIDCMIEELSSNTACPIMAPQQQHGLLEGSFLKNDHDASSCRDSLLETGSHRLSNVQFQDTSAARNKSSTAPHNGTSQVN

AIRTTAAGLEQQLNKMGEDENNGIQLVHSLLACAESIQRGNLSFAEETLRRIELLSLPPGPMGKVATHFIGALTRRIYGVASSSGNNSSSNQSDSLLGLL

HFYFYESCPFLRFAHFTANQAILEAVTGLKEVHVIDFNLMQGLQWPALIQALSLRQGGPPRLRLTGIGPPQPSGSDTLQEIGTKLAELAKTVRVDFEFRG

VIAVKLDDIKPWMLQIRHGEAVAVNSVLQLHKLLYSAGPEAPIDAVLLLVRELKPKIFTIVEHEANHNQPSFLGRFIEALHYYSTMFDALEACNLPSENN

EQVLIEMYLGREIYNIVACEDGARTERHENLFQWRLRLLKAGYRPIQLGLNAFKQASMLLTMFSGEGYRVEEKLGCLTLGWHSRPLIAASAWKCA

>Pp1s63_181V6

MPPFGSLLEPPTNLRGGQSSFYVKQEPGVDSVRKLRMDTPQSAFSSYRDTHPTLRKPLQHELSAFGPFSAQGQSSMDASTSNAAPHPPSGDTHTSLRKSA

SRQPIQVHQYLRPAVLQPTTSVSPSKKQCTEKSRQFNDVFLSAHTNAMPSKSSDVGSSGLATGIRDTTSDGFVVVSCKQMQCKQECDIASTSFSAPPLSR

KRSHSELAEESCDSELVLGVGGRSSVSREGSEYRNSRASSPRKSQKSSDTRLDLVSADLCLAPPIVAPEYSSSSSQQEAPSSSMLETQPLGESSKVSHSR

GTYVSPTQSHLSFQKIKSKDVSRAEGVTCISQAESHPTGSRCQQPVRPLLNLEVIKRECDAVERERDNEESLAQSRNHHEGFELGLFPGVFAADQGKKSE

ASGLQLVHLLLACADAISKNKIEIATQKLEELYSHASLFGDSMQRIAAFFTEALAARIVGKDNPAYKNLMLQSHLDDYLSAFTTLYKICPYFQFGHFTAN

QAILEAVEGYSVVHIIDMDLMQGFQWPGFIQSLSEREGGPPKLKITGVGTSCTSLQDTGRRLAAFAETYGVPFEFHAVVGELEDLSPMELGAKPGEAVAV

NCVMQLHRLLNNGDKLQNFISGLRSIHPVMLTLVEQEANHNTSSFMGRFVEALHYYAAVFDSLDSSLPLASEERAKIEQLYFAQQIKNIVACEGADRIER

HETLELWQKRMKLAGFRQWPLSSHSVTQAKLLLSLSPCDGYCLSQQPGGSISLNWQDRSLLTASTWVL

>Pp1s165_99V6

MVGEKVESPGGTPRGGGDGDVDDTHNSPAVVGHELVTLLIACAEAVSTQSLSLVNHLLPKLGELASPQGTAMQRVAAYFTEGLACRVAHLWPHIYQPLPI

ESSLNEEELQTAFHLLNHVVPYTKFAHFTANDIILQGFEGADRVHVIDFDVKQGLQWPALFQSLAVRECGPPSHIRITGIGECKEDLLETGDRLAEFAEE

FNIPFTFHAVIDRLEDVRLWMLHVKENEAVAVNCISQLHRLLYDSGETIEGFLNLIGSTKPKVVAVVEQEGSHNSPQFEGRFLESLQYYSAVFDSLEANI

SRESSARVQVEQLFAREIRNILSCEGTDRMERHENISRWRSIMSRSGFVKVPLEDSAYTQALILLRMFDSDGYTLAEENGAVTLGWMEQPLLTASAWKPD

KDFVPTGTSLVTNEKQRAV

>Pp1s165_77V6

MMPPFGSLLEPSTDSRGGPGSFYVKPELGVDAVKKIGMDTPQSAFSSYRDAHPPLRKPLQHELSAFGPFAAQGQSSVDASAVNVAPILPSVDAHLQLWRP

TSRQPVQVKHYLQPTVLQSTTNNFLSKKQSTEVSQQFNGMFRSTNANAMHSKSSDVGSSGLVTGIRDTTSDGFVVVSCKQVKCKQECDIASSSFSAPPLS

RKRSHSDLAEEESCDSELVLGIGGRSSVSRGGSEFRNSRASSPRKSLKSSDTRLDLVSADLCLAPPVLAPEYSSSSSQQEVPSSSMLEVQPFGESSKVSH

SRGPYASPTESHLSFQKIKSQDASRMEGVTCVSQAESHPTGSRCCQPVRPLLNLEAIKRECDAVERERDNEESLAQSRTHHEGVELGLFPGVFAADTGKK

SETSGLQLVNLLHDCAEAVSKGKIETATQKLEELYSHASLFGDFMQRVAAFFTEGLAARMVGKDKPMYKNLMVQSRLDDYLSAFTTLYKVCPYFQFGHFA

ANQAILEAVEGRSVVHIIDMDLMQGLQWPGFIQSLSEREDGPPKLKITGIGTSCNSLQDTGRRLASFAETYGVPFEFHAVVGELEDLTPMELGAKPGEAV

AVNCVMQLHRLLNNGDKLHNFIAGLRSLHPVMLTLVEQEANHNTSSFLGRFVEAVHYYAAVFDSLDSSLPLASEERAKIEQLYFAQQIKNIVACEGVDRI

ERHETLDLWQKRMVTAGFRQLPLSSHAVTQAKLLLSLSPCGGYRLSQQPGGSISLNWQDQCLLSASSWVL

>Pp1s63_198V6

MREIGVLEYGEMLYTGELDELFRLPAVKTGRSSSPSLPCSNASSQYGLHSQRSELGLSGSPSHRGGMSGNNRVLLPGKFVLKKGLEPWSTVERRGEQLSS

TDPAGSPSFRRAGTTMWPDKTSTACEAETSTGQWTSPRCGEDPRNEGDLIHFHQHQNQSTSNSPLCPGTPIRHHYSQMAPSSPLQIISSPDVKKCIAPMS

EPRSVLDLTISPQQHQLPTIRATLPRRFRETFLEHNVAPVTAPSLSPSPRVLNFESCSNGSTKSGTFADQYGPSPTNASTWAPDLATQGYPTSIPTHKVE

ELPVLPTAQSFIQETSSLDEWEAATITSPQHKLRAKQWMVSLMEDLRNAEDANSAQETEECTTPFTPGVDSCAPYFLTSQNGLGSPCPSDYTGDDTPRGS

RLEHLQVESPCGTPRGGGDGEDAQNSPVMVGHELVTLLIACAEAVSTQSLSLVNHLLQKLGEHASPQGTAMQRVAAYFTEGLACRVAHLWPHVYQPLPTH

SNLNDEQLQTAFHLLNHVVPYTKFAHFTVNDIILQAFNGADRVHVIDFDIKQGLQWPALFQSLAERECGPPSHIRITGIGECKDDLLETGDRLAEFAEEF

NIPFSFHAVIDRLEDVRLWMLHVKENEAVAVNCISQFHRLLYDSGETIKDFLNLIGSTKPRVVAIVEQEGSHNSPHFEGRFLESLKYYSAIFDSLEANLS

RESCVRVQVEQLFALEIRNILSCEGAERVERHEDTARWSVLLSQSDFVNVPLEDSANTQAQILLRMFDSDGYTLTAENGSLTLGWVEQPLLTVSAWKPDK

DFVLTGKSLVTNEKQQAV

>Pp1s346_13V6

MSVQYRPELGTMVLTPGYPPGKEREYLSDTANSQQTPSYYGAQKSYADGQRQSAYGMKNKSHSSPVSPLSPQDSSQAASDNGQRMSAGWSSASYQSESSS

HSDGSLEGPGKLEEADYYGRQHRHGEQLTGSVAYHNTPSSVLRPMGYPAETAQAYQMPNYQQAVRYIPEEQYAQSQSNYAQRNPEMAHMLQVLESALLDD

DDGADLPGSLGNGHDPASEGNWADTIEEFMAADASPADSSTVTSATTPPEYGKQCRNGSTNNYTGAVTARVEEPPPQKLVVGTRSRSEQLLVACAEALSN

NDMPLANVLIAQLNQVVSIYGDPMQRLAAYMVEGLVARVAASGKGIYRSLKCKDPPTRDLLSAMQILYEVCPYFKFGYMAANGSIAEAFQNESRVHIIDF

QIAQGTQWTTLIQALAARPGGPPHLRITGIDDPMPGPNSNAGVEMVGKRLAKLAEAVGVPFDFHPVAKKGPEVEAWMLERQPGEALAVNFALHLHHMPDE

SVCTSNPRDRILHMVKALNPKVVTLVEQESNTNTAPFFPRFLEAMNYYAAIFESLDITLARESKERVNVEQQCLARDIVNIIACEGIDRVERHEMMGKWR

ARLTMAGFRPYPLSQTVNNTIKTLLESYSDKYRLKDEGGALYLGWKNRSLIVSSAWQ

>Pp1s456_3V6

MSVQYEPELGSMVLTPGFPPGKEREYFSDTANSQQAPNYFGARTSYANDHRQNAYGLKIKSHGSPISPLSPQDSSHAASDNGQRMSGGWNSASYQSESSS

HSDTSLEGHVKQEEADYYGRQYGHIEQSEGSLAYHNSPSSVLKPMPYPGAIAQGYSMPNYQQDGRHVPDGQYAQSQSNCAQRNPEMVHMLQVLENALLDD

DDDDDHRAPEGNWADTIEKFLAADNSAIKPSPVTSRTTQPDYGKQQCNENAINFTGAVTARVEELALQKLVVATRSRSEQLLVACAEAVSNNDMPLANVL

IAQLNQEVSIHGDPMQRLAAYMVEGLVARVAASGKSIYTSLKCKEPPTRDLLSAMQILYEVCPYFKFGYMAANGAIAEAFQNESRVHIIDFQIAQGTQWT

TLIRALAARPGGPPHVRITGIDDPMPGPTPNVGVEMVGKRLANLAEAVGVPFVFHPVAKKGTEIEAWMLERQQGEALAVNFALQLHHMPDESVCTSNPRD

RMLHMIKGLNPKVMTLVEQESNTNTAPFFPRFLEALSYYSAIFESLDITLARESKERVNVEQQCLARDIVNIIACEGIDRVERHEMMGKWRARLTMAGFR

PYPLSQTVNNTIKTLLESYSDKYRLKEEGGALFLGWKNRPLIVSSAWH

>Pp1s130_58V6

MAATHVVSKKRSPTIFSEVATATCKRWKSEAAWHHVEEMAPEPPPIQDFDLKVKTSCLKMVQPEPTSVLDLQASPGRSCSSSTSLSSGTDSPHSISTDSP

NFSTTVQQVELRPPLEEVLKSDIDFASTDLDFGFELTSLDHCSVVTEHGYSVSLLSEFLGDPDENLLLPESFHDAKRLQELDDSLSSMLSEVRSSGSDSG

SSVPTTVELARLVESLPCSDLRRHGGVDTKHHHHHSRSESWGSTSKLQTLQHPEDSGLQLVHMLLACAEAIEKSDFNKAKPILDQLLRSSDPYGDPMQRI

ALYFGEALTDHLAGVVSPSETHLLSDSKLAYQAFYKVLPFAKFSHVTANQTIYEAVVRSQNVHVVDLDIQLGLQWPCFIQSLAMRPGGAPHLRISAIGTN

AENLQTTKRRLSEFAEALKVPFEFTPVLSSLENLTAAMLDIRSEEDLAINCSQVLHTLSGEEAVLDKLLSMFHNLKPNVVTLLEAEANHNGASFIARFVE

ALHYYCALFDSLEGALGRDSADRYHIESTALAAEIKEIVAFKGNRRRVRHVRSETWRGLFAKAGFLSMAFSSYTVQQAQMLLEVLTSKPMQQANATMPYK

LSQESTSLILGWQETPVIGVSAWTC

>Pp1s31_35V6

MADGDLTSIFSQRTQPISDPWGVAVPQQQLLHYRQFPQLLQDLDNARRDYSSCQPPQSPCDIQEQNSNLSNRMLQAKVGSYNPGFSSLRPSALPSQNHLN

FPAVSPQIVQYSLPQALVGTKGDYQGSDFHLQGSQPTIAAGSNAFTEVISRISPVGRDTLAGADQQHQALFVESGKNIEHPQLSTWPSITTLYKVDERAT

ELQTDTLGELRSGPEYVADLYTGGLLLCGQVYNEGRCMEVQSNSLSSGAESMPILQQNFLGDGGIIKVLDTASNRQSQPTSATGLQIVQLLLSCAEAISN

QQIDVAYVFLRRLNGMLGHCTTTMQRLGTVLVDALYARITNSIDSGRYKGLEKDGDVAILDMLHSFSVIYDYTPFIKFPNLTLNQIILDAVEGAQHVHVI

DLNTGWRGMQWPAVIQSLALRPGGPPHLRITSIGKLDDLEQSREKLQDFARNLQVPFEFCPLVVDMKSFDVRLLDLRDWEVLCINSANQFHQLLTWGDER

FHRFLCDLRSLNPRVVAFSENDADHNSPKFLNRFFECLRYYSAVYDALDAALPSGSPALQQVEHLFTGQKIRNIVACEGEDRITRHEPMKNWSRRMELAG

FRPMPLSTRAISQARALLEIYFSLSGYNLRTENGILVLGWDNTPLVGVSAWRA

>Pp1s31_40V6

MTATHVASNKRSSTSFPEFVTAPCKRWKSEAAWHHVEEIVPEPPPAQDFDLKVKTCYSNMVHPEPTSVLDLQTSPGRSCSSTSLSSGTDSPHSVSTDSSN

FSTTLQQVEQDDIANWVDCMGLPEFEECGRPLEEVLKSDIDFNTTDIDFGFEMTSLEHCSAVAEHDFSVSLLSDFLGEPDENLLLPESFHYAKRLQELDD

SLSSMLNEVRSSGSDSESSIPTTAELVRLVEAQSCEDLGRQRACGARNHHHSRAGSLGSTSEPQTLQQPENSGLQLVHLLLACAEAIDKSHFHKANPILD

QLGRFSNAYGGPMQRIALYFGNALSNHLAGVVSPTDPHSPSDSKFAYQAFYKILPFAKFSHVTANQTIYEAVLRSQNVHVVDLDIQQGLQWPCFIQSLAM

RPGGAPHLRISAVGMNMESLQTTKRWLTEFAEDLKVPFEFTPVLSTLENLTPAMLNIRADEDLAINCSQVLHTLSGDEAVLEKLLCMFRNLRPNVVTLLE

AEANYNAASFITRFIEALHYYCALFDSLEGALGRDSADRFHIESTAFAAEINDILASKDSSRRVRHVRSETWRALFKKAGFRSMAFSSYTVRQAQMLLEI

LTSKHLMQANSPIPYKLSEESTSLILGWQETPVIGVSAWSC

>Pp1s17_52V6

MTLQILSKRSCPVEVGPSAKRWKSQSSWAIEGVAPVAATSAAAEAAEGSACVKQEDPITLQEPTSVLDLQTSPGRSSSSGSLSSHSSLSGDETSFPSLAG

TDICSEDQTSSELLPVSTVESVHNDHDIASWMECMEDPNGPLALTDFSKCEEGGEVRQAEVKLDGGIETGIDHFVNADIDFGFELSLDDNDHEAFANYML

SDPGLCLMDEPKFLNFPEGFQDAHRLLELGDSLSSMIDSAPGIGVGEVQSSDASSTPGSDVSYEPGYSGSQNWESNPLEVQQPLDSGLQLVHLLLACAEA

IEESNFDTARPMLSRLKAISNPYGDPMQRISLYFADALSDRLTKESETPVSAAPISSPVELDTDLAYQSFYEVLPFAKFTHFTANQAIFEAVGYHNKIHV

VDLDIQQGLQWPSFLQTLALRPGGPPSLKITAVGTNAASLQLTKRRLSEFAQALEVPFELIVLVEDLDNLDKEKFQIEPDEALAVNCSQVLHRLSGSEAV

LQKLLLLLRSLNPEVVTLLEVEANHNGANLISRFVEALHYYCALFDALEASVSSDSPDRFRIENITLASEIRGIVALEGSGRGARHVKSETWQSHFTKCG

FRNRPLSSYAVQQAQLLLGYFVTGETPTYKLSEEFGVLIMGWQDTPVMAVSSWSC

>Pp1s116_166V6

MSVAQVLRSANGHQEFFPSADADYVRVLASHSRLDVHSLVLHGEGDSIANQTLKLLNERRCTPCGHWNGFSGGLMQLRDLLLETAQLISQCDWDRARPLL

QLLSRRVSTTGDSSERVASCFFEALATRFSRVSGIQINELLPSRIQGPSNQEMISAYLALNQVTPFMRFAHLTANQALLEALTGENFVHIVDLEIGHGIQ

WPLFMQALADLRGEEGYTIQHLRITGVGQDRDVLNRTGIRLAEFAQSINLPFEFSPLVQISEHLVPRMLGLRVGEAVAINCMLQLHRLLAKGPEKLISFL

CMLESLTPKVVTLAELEASHNQPHFLDRFAEALNHYSTLFDSLDATLPPTSADRIRVEQTWCKMEIVNIVACDGAERIVRHQRFELWRRYFHRAGFQLLS

TSRFATSQARLLLRLHYPCDDYQLLENVDDGCLLLGWQDHPLFCVSSWNTNNIASSGRVWNKDSTTKSDMKPQVLFLAGPSKPATDLYLIPSPQW

>Pp1s281_32V6

MAEITHYRPHSDTNTVKFATVPDLQTYLGISSWLELNQHWRLGDATKFFKVAEHEGQNSMSSAVDSKRLDSGKSGNQHSTALAMSKEPTSVLDLPSSCSV

SLSSQHSMNSVSDGISKALQTVADQLLSDEQDSAHPMQSATPETSNMLMDQDVMTWMSLTRGSDHSASDMDPVPDWFHKQPERLDLNAQQYYCSKTGLDF

HKNPGTMERICNNLPSSHHHYVAQPFKLISQAQLTMSETAGNQQTGFFDTSTSSNQRAFQQCRNVKAQGATLMSMIDGTGAITGSQLLNSTVVPTPELWK

LLSSQHDASPQCGNQGPPNKQAPITQARDQQNRSTSLAIPTQQPQPQLPYHFGQTLYPGVTPQDQETGIHMVHLLLACAEAVDMCQSATAGPMLARLRSI

YDPEGEPMRRIALYFAEALFERLTIEMNRKQSSHHGSCVRFPEPEAYYQILPFKKFTHLTANQALLEGVANYPRVHIIDFNIRQGLQWPSFIQSLAMLPR

GPPQLKFTAVQTDAATVQKTGNRLAEFARTMHVPFEFYILEESVESFHQGMISPRAEEALAVNCSDMLHRLLRKEGKLTELLGKIRSLQPVVVTVLEVDA

NHNEPSFMPRFVHALHYYCAVFDSLEAALLRNSLDRLRIENHCFSTQIRSIIALEDVDREIRHVRAETWQSHFLQAGFRAVTVSRYAADQAQLLLGLYKP

SDRMPFTLSSGFGGLSLGWRETPVVAVSSWTFSPPPY

>Pp1s130_63V6

MADGDLASIFRQHTQPVSDPWGVAVPQHQLSHFQQMPQTLQDTDNVRWDYPNSLSPQSPFDIQDHNSNFSTRMLQSKVGSYNHSYSSLRPSTIPPQSHLQ

FSTISPQLVEPVQSLQSQTLSGNKRDFQGSSFQVQNKQPTRSAGRNIYSEVVTSTSPVGQENLVTVSHHQSQFSGNNYNISHGQPSTWPTAPTGCKVGEL

ATELQPEILGDLRPGPGYLADHYPGRLLQSGQAYNQNQNLGPQGNPNDLPSGSGTLSSSLQQNFPGDDGMIRVLDTACSRRNQQTSATGLQLVHLLLLCA

EAISNQQMDLAHVVLTRLNAMLVPCTSTMQRLAAVFVDALHARITNSATTGRYKGLERDNDVAILDMLQSFSVIYDHTPFIKLPHLTLNQIILDAVEGEP

HVHVIDLNTGWRGMQWPGFIQALALRPGGPPKLRITAIGKADDLEHSREKLQDYARHLQVPFEFCPLVVDMKSFDVRLLDMRDWEVVCINSANQFHQLLI

WGDECFHKFLCDLKSLNPRVLAFTENDADHNSPKFLNRFFECLRYYSAVYDALDSSLPNGSAALQQVEHLFTGQKIRNIVAMEGEDRITRHESLTSWSRR

MEMAGFRPVPVSSRAISQAGLLLRMYFAQSGYTLRTENGNVSLGWDNMSLVGASAWRA

>Pp1s324_56V6

MNCSGYSPGYRDHLHMTEYLPSPCHSESEVVGREHRDAGRSSKLSDQRAHVPDLDFGVAAKWSSTGQMSSCSPQAMRCDAKEPYQSPVPIMNAAPRDDWG

KIADALSHHQQLPSHNDKAQQGSNGCGAQTREEARDQSAEMDEDSIAAWVDGTITEMMEAMPGVAIEQLFTNLPEFLSPCNLHLKGLIGCRLQLLLGGAG

YPSKNFPPPGPNRSGKRTREVQVEWTNSKTFQNESQDLHETLPRHHVRPENFISQKKVAASLSRVDLRCPKPLKRSIDNLQLSLEPADERNLRHLHIGLQ

ATQKDQSSHQVRHSRAQSTEPVQQHYHKLHTHNVQDKADGFLQRSPAPSSASQFSTLNTMGSEDPPSQPQKLPQTTAQNAQREDTVAPDEGLQLMSLLLQ

CAEAISADDNNQATAILPQLSELATPFGTSVQRVVAYFAESMGSRLVTSSLGICRPLPCKQPASNQSIVSAMQVFNEICPFVKFSHFTANQAIAEAFEGK

FNVHIIDVDIMQGLQWPSLFQVLASRAGGPPHVHITGLGTSAESLDATGKRLKDFAGSFGISFEFTAIADKMSNVDISTLKVAFSDALAVHWMHHSLYDV

TGSDLDTLSLIQKLNPKVITLVEQDFRHSGTFLSRFLEALHYYSAMFDSLGATCKDDSPERYMVEQQLLSCEIKNIVAFDGPGRKINHKFDQWRDELSKA

GFKPVSLSGKASHQAALLLQSLFPCDGYTLLEHSGSLKLGWKDLYLFTASAWTRV

>Pp1s240_118V6

MQLFVPPLRMRVHSHNFSRPQLSLRFPGGLHEMLGSVGSNASASSEPVPEVSSPHRTYGYVDCSNSLGGLMLLRDILVDTAQYISQCDWERARPLLQVLR

RQVSSTGDSSERVASCFFEALATRFSRVSGTEINELLSSPTQEPSSEEILSAFLALNQVTPFMRFAHLTANQALLEALTGEDFVHIVDLDIGHGVQWPPF

MQALADIRGEEGHTIQHLRITGVGKDREMLDRTGTRLAEFAQSIQLPFEFTPLVQAPENLIPSMFGLRIGEAVAFNCMLQLHQLLAKGSEKLTSFLYMLE

SLTPRVVTLAELEASHNQPHFLDRFAEALNHYSTLFDSLDATLPPTSPERIRVEQTWYKMEIINIVACDGTERTVRHQRCEQWRRFFERAGFQLLPTSRF

ATSQARLLLRLHYPCDGYRLVEDVEDGCLLLGWQDRPLFCVSSWHPSNM

>Pp1s362_33V6

MAGRDVGAAICMPRSDPSIVPHPQQILHFSHYNSKQDFSSPSMIPQYGSPRYRQSTEQASLQDLFSSPLAGSMTMQPLHPGIELDRVGSPLQRGQSLQTC

SSPSDPVNLQHSPHVGDIEKANSPFSRRTNVALMQDGSPSLKLEQGLHCVAPTTPSTVLPPLKMDDFNMGFGGGPTYGSASGNEWMESFIGDLQPCESTE

SSALPPVMVDSWENDFTLPRELRVRINSESEDASAMAELQRTGLQQEDYELQHRLRTLQQLDQAAADPSLQEELSFGASDHTQYANRKDVSEAEERSTES

DYSGGLDKDHSVHLVHLLLECATQIEKNQHLAVSTLCRLRDLSSPLGDPMQRVAAYFCDALTKRIARGKGEADPGVLEAPHNSPKACQVLNEACPYMKFA

HLTANQAILEAVKGCESVHILDFGITHGIQWAALLQAFASLPKKQPPPKVRITGISVNNPASESASLSVLATGKRLQSFAEHLNVEFEFCPVILVSMEDF

TPESIQLNPDEKTVANFMLQLHEMLDEEGSPSILRLLRSVISLSPALVTLTEHDAALNRPEFRPRFMDALHFYCALFDSLDSTMPRDCHDRLNVENNYFA

KQIENIVANEGVDRTERYECTETWIRIMETVGFTLVPLSHYAYSQAQQLLWQFCDSFRLQRPSGCIALAWQDRSLITVSAWKCS

>Pp1s882_1V6

MALVCPNPRERLGLVTKEPTGVGSRTHYNVGFPGYRDQLRMTDNLRDPFHSGPETGGRDPQDAERRGELQEHRVHLPDLDFGVGAKWNCTGQISSWSSYD

STSQSFPSTSAAGWVVSPLGSNNSPFPSDGSFSDHLSSGLSNTSFPGALPASLQVQSGGSAGGFFSGSTQTASCNGNQLYQNPPSLMNAAPRDSWGKIED

APSPHHQLSCQYDMEQHGNYGYGVQPAREAVKDHFSGMDEDSVAAWVDGMIMEMMEAMPGVPIEQILTNLSEVLAPSNSQLERIIGSRVQSLYGGAGLRV

QTFLHTGPSRFGKRTREGQIGWRNAKSSQNEARAYQDTPSHHHVRPDNFMEQNAVASLARDELHYPRPQKRTIDNLQLSLEPADERSLRHLHTAQQPMPE

DQYSHQVLRSRDQTTKPVQHQHYFQPHLQHQQQKSGEIQNDFSPPSNAAQYPILNTRGFNDAVSLPQNVPQSTPRNAQPEPPNATDEEGLQLLALLLQCA

EAVSSDDFDQANSILPQLSELATPYGTSVQRVVAYFAEGMASRLVTYCLGICPPLSSKQLVSNQSFLSAMQVFNEICPFVKFSHFTANQAIFDAFEGMFN

VHVIDIDIMHGLQWPPLFQLLASRPGGPPHVHITGLGTSIETLEATGKRLTDFAASFNISFEFTAVADKIGNVDLSTLKVEFSDAVAVHWMHHSLYDVTG

SDLNTLNLIEKLNPKVITLVEQDLRHGGTFLSRFVEALHYYSALFDSLGASYKADSPERHMVEQQLLSCEIKNILAFGGPARTGEAKFDQWRDELGKRFK

PVSLSGKAAHQAALLLQGLFPCEGYTLLEHRGTLKLGWKDLYLFTASAWTNE

>Pp1s85_139V6

MAIVCPNPSKRLRSVSTELLPGVITGTCTQYADLPGYRSLVTAEDRDQLRMTDHYTLRNSGQISSDAGGSCGVHWDVHRRADSLDHGSDHLVDLDFTVGS

NWPNSTLPPVAPVSSWSNYTFASNSFPTSSSGWEVPPLEDNITVTFPSNVTFPNHLSSGISAPNNFTNTLPQSLQLETCGSASIRDGFFSSGLAQARSGN

VQHLYQTQPLLINAEPGNSLGKIADAVPSHQHQHVPQFDMPQHRTHSFSAHNASENVKDQFAGLDEDAVSQWVDGMIRGMMEVMPGMPIEHLFANLSEAL

APGYVNAERVIRSRLHPLYASARGRVQNTLPIDLARAGKRPRVEYNDGAKGQVYRENANSSQNTSQTFKNAISGLHDRQENVMQIQAAVSHPSPELREDQ

QLPFSSSQKRTDDNLQLSLEPADERSLRHLHTTRQPSLSKFGHQGVTSRDSPGPSHLKQYPQIQPHYHQKPKLGNTILDFDVRSSVSQCSAQNARDCQGL

PSLPLDVPQTSSQKRQSHEPSTSQEGLQLLALLLQCAEAVSSGNHDEANTILPQLREQVTPYGSSVQRVVAYFAEGMASRLVTSCLGINSPLPRNDLVNN

PSFTSAIQVFNEICPFVKFSHFTAIQAISEAFEGMNNVHVIDMDIMHGLQWHLLLQNLAKRPGGPPHVHITGLGTSVETLDATGKRLIDFAATLGVSFQF

TAVAEKFGKLDPSALKVEFSDALAVHWMHHSLYDVSGCDSATLGLMHKLSPKIITIVEQDLRHGGPFLNRFVEALHYYSALFDSLGASYNRKSLKRHMVE

QQLLSCEIKNILAIGGPGRSGTTKFDHWRDKLSEAGFNPVALSAQAVHQAALLLSQGFYPGEGYTLLEDLGALKLGWEDLCLFTASAWTST

>Pp1s84_299V6

MAGSKHERSGDSPVGTGEVSPLAAQLQQAWLQTQMPKAQKRSFTSVANAQNQLPRLSPPGPGSEMSASSKPPSSSVLKSLSTLFPELANVQEKKEAKISS

VFEEGEQPIPIHPELRQKKAECLEVMFGQDVVEQTQAQGLRDYDRHASTERSLSDSLIQQSDDSLDFSDLGPLSVSNSFSRPSAQPGRGTFEDDLSYICS

AYGSRPSASEYETSAEVEQEKTPLFEYLTDILMDENVEEKKCMFIEMSAYQAMAKELGDLISYDPPPMPIPETRRSDPHFEEDVRFVDSWIDEILSGPLP

ADRTDSPGAEAKLDIKHGSSPEELYSHTDADRGSSVWNDTASDTASYLHPDSTLSPVDFGNSHALENGSGGSLQVGTRHLSSISSSNGNGVHAPPVDLTD

LLIRCAQAVEQADYRHANELIHELRHHSSAYGNGSQRMAHYFMEALVAKISGTGGQLYSALSNYRPSEAQMLKAQMLFCEHCPFIQVPHIYANHAIMVAF

KGAPRVHIIDYGILYGIQWPCLIHQLSQRPEGPPHLRITGIDRPQPGFRPSARIQDTGRRLAKLAKQMGVPFEFHAIAEKWEAITPAHLLLRDDEVLAVN

SMFRFRHLLDESVTAASPRNLVLSRIRSLNPKIFVQGVLNAGYNAPFFMSRFREALAYFSTIFDSMECSFPAEHPDRQIIDHEIVGREILNVVACEGPER

VERSETYRQWQARTMRAGFQQKPNSPDVMAKIRMAMRSYHRDYGIGEDGAWFLLGWKERITHAMTVWEPLPDSP

>Pp1s205_1V6

MVITAGSSGGAGSGSESTRINQMRAAEEQWWVDVLAPVSVIGSSAVSQQESVAPNANVEFGVPRLYVEEPDNMLFAQPADPIVMKLLMGDGQFCGQGDIQ

SVQRSRDIDTVMFEQDSWSSNQHQMCHHLGDKPPSRLTLPDHFTGGTFSSFLNSSVSLAPSIPNSDSHASFTSLPRALSLPSQLHDIPHNYPKGTGVSVL

STTTTPVASMSRPIGHPYLASSSFLDAMSVPPNASARGDCVAQSPLLGNSIYEDQLSQRNALTVDQTSLVMEERSVSMQGSLLSSPSSLYAVHLNFPSTT

PAYHHQEPEEGFEHRGILQRSFSSLPSTASQNQRIKAPAMMSGDYLHQLSWNAGRMSPQWQKLAPLNRSLRATGERKRETINDRTLGALSPLSTVPGFCL

GRPQDPGDSCEQVGVELKQEDVNLDTESIDGGVIPEGGLAIVNLLLRAAEAVDNGDAEMAKAILARLNQHISPSREQSIQRVAHYFREALETRIMGWENF

VVQLSQDRVLHPLEEFHKVNAYVRFCEVSPYHKFAHFTANQAILETLEGEESIHIIDFQMGAGAQWASFLQDIACLRAAGKAVPTVRLTVVGTGADQIHA

TGANLCNFARLMSIALEFQAVVTRPECLEVSMFRLRDHEAVAVNFIFSLHELLDGDTSNGLATVLKAVLEARPKVVTTVEQEAYHSGPSFQQRFSEALQY

YMFLFDSLTNPLEAGVDSSVNLSIESYLLAPEIMNIVACDGVARVKRHERLEHWRKRMLAARFHSRPLSEVSLLQSEILVTQLSSRSGFQVICDQGSLLL

SWRGRPLLAASSWIC

>Pp1s98_14V6

MIIAMAGSKHERTEGSGGAGEGPSLAAQLQQVWLQTQMPKAQKRSFTPDSSTQNQIPRLSTSNLGADTSALSKPSALSALKSLSNLFPELANVQEEKKAI

ISSVFKEGEHPVALNPAMLQKKNECLEMMLTDNFGNEAQSERSQVSHSRTSSQTMRSNSYGIQPSSDSLDFSSMCPLTSFNSFSGSTQRSAVDTPEEDFF

CPFGRSEPSDSPSVDNTEATAEVEVAVELEKTPLFLYLTDMLMDEKVEEKKCMFVEMSAYQAMAKELGDLISYDPSYSSMPGAENTNSEFHFEDNSGLFE

KEYLEFQGEEDVVEDVGHVDSWINEILSGPLPPELRDSPDSETKHGHKHAESPEESCSNADSEFCSCNTLKDEDFEVAQHHWAYVQANGSYLDSSSSLLD

SVNSAHSLQNKNGRMLPVGFTNPISSSNGNEVHVTPVDLTNLLIRCAHAVEQGNFGYANELINELREHSSAYGNGRQRMAHYFVEALVAKMSGTGGQLYS

ALSNNRPSEAQMLKALMLFCEHCPFIQVPHIFANHSIVEAFKGASRVHIIDYGILYGVQWPCLLYQLSTRPEGPPHLRITGIDRPQPGFRPSARIQDTGR

RLAKLAKKMGVPFKFHAIAEKWEAITPAHLLLREDEVLAVNCMFRFRHLLDESVTAASPRNLVLSRIKSLNPKVFVQGVFNAGYNAPFFMSRFREALSHF

STIFDAMESSFPPDHVDRQLIDHEIVGREILNVVACEGLERVERTETYRQWQARTTRAGFQQIPSSGETMAKIKMAMRVYHRDYGVGHDGHWFLIGWKNH

ITHAMTIWEPIRDGSP

>Pp1s20_86V6

MLGTRQSRSGALARETSPLASQLQQAWLQSQIQSQAPKHQKRGFTTLGEDDVSVQNRVVQQFHQVASDLSSIVADHANHTGFPNVVEKEELYFHKTRLEM

PEFPRRPETPQLVSYSRKADKPQKPRSGNFLERRSSSYSSLSSASSGSLSPTSTVSFESVQLHQEFDAPLDVADYAISGTLDLACQSEPLVRFLDGMLAE

DNMDGKKFTLQEGFDYHAIAKEFDDIARPNLASEYGSSDFADGDYDMSWASMGILPTEEAVADKLIHAYTSNLNKHVTDVSENVDSSTWVNEGDACTDHS

DQSWDWYLERLRPNPTGRSLSSSIFQGDSLGQDMSPDALSAFPAVCVDLKTLLLKCAFVVSKDDVRTANDLIRELRMHSSVHGTALQRMAYYYMEALVAK

MSGTGPQLYMAITSNTPSTATMLKAHRLFVDYSPYIKVTHFFSTKTILDAFEGADRVHLVDYGVAYGAQWPCLIQRLSQRKGGPPHLRITCIDLPQPGGK

VSARVKEVGCRLAEFAQLWEVPFEFNALADKWESITSAHLNLNQDEVLAVNCQYRLRNLLDESIMAASPRKLLLEKIRFMNPKVFIMLTVNANYNAPFFM

TRFRESMKYYFTMFDAMEVSMPANDPDRVILEREFYGREILNIVACEGVERVERAEPYRQWQTLTQRAGFTQKPLSPIIASKIKAMMGSYHKDYGVGEDG

SWFLMGWKNQIVRAMTVWEPTSATTVT

>Pp1s359_32V6

MRMAAKLWPKYGRVHGQMRLNLSGLRFLQPAHLPYFPSRRESVYAMPHMLTSMEYFGFLEEVGNKEELKAGNQRTASVLVLIDSGMVGTRRVEGSSQATH

TEISPLESLLQRAWLQTHKSKGSRRLSSAPLPDVADEKRGFSRIASLPAFSGTRFPRSDDQEESDKVYFHKSRLDASTVLVSRFSLPELTSYSSKTYVPL

SVSLVQWTHSEPVAGHGLSQALKPLTKSSTESSETLSGQSRHSLGATESEVSSTSSVDFERQAQPMASFLGGILTDENLEEENLHETRAYQAIAKEIADL

IGLNSSVETETSDIVDYESCWADAEPYRTEAEMHILEQSGAYQAMVKEIAELISPHPAVIDTDETDLSDGKASDSADFRPTEELAARGMMEAYSHILTRH

LEDVAINSDSDAASTIKYRTTETDTIVTYFNEDWKLYLQNLNQNFTPTSNFDFNLLLCGAVEGSKVSADNAVPSSSSGSLTELLYRCALAVSQGNVREAT

DLLSDLRQISSPNGNATQRMAHYFMEALVAKLSGTGEELYRVIINNGPSAAIVFKAIRLYLENCPYLIFAHFFTVKSIVDVFEGAARVHLICYGIQYGVE

LPSLIQYLSQRPEGAPHLRITGIDSPHPGNNPCLKINETGRRLAMFAKKWGVPFEYVALAGSWESFTARDMNLREDEVLAVSSQDSLHTLPDESVMATSP

RELVFRRIRSMNPKLFVMVGMHGGHNAPFFMTRFRESVKHYSAIYEGLDISMPRDDPDRVIVEREIFGSQILNIVACEGQARVERAEPYRQWQNRFQRAG

FTQLPILDTVFNKMKAMMGAFHKDYGVGRDDGWFLMGIRNQIVKFCSAWEPKCP

>Pp1s213_67V6

MTEKATSDSSNPVRRGSGGSSLESLLQQACLQLQKPKLQRRSESDPRLDVSVEKRVNLDSACLGDAADGLGETYFHKIRPEANSNFLERTAPPKLETISK

KTNVPRSNSGCVLGGRGSPLGTSDNSSQFSPSPTRSLSNDTQASVSGGNCDAVDFEGSSSVSADLAGQMESMIRYIDDILMNENMEDEKSLMQQTGAIHA

MAKDLAGLIDPDSSVDETNNLEVVGGDYSNDWAKNEILSAEETNAVENSSVYRDLRKEIADLISSEANVLDAGGFGGDNDQSNTSTTFLPTEDNTTLKLM

EGYTRMLSKHIADVSGASNSDNVDYRSFITESDDLVVHFDDRLKYYLDNLNKNPPPNSALDFKLLLRTPGERSSSSSSRVVYSSITDLLHRCAFAVSQGK

TGSAADYLAELRSLSSPYGDYMQRMAHYFMEALVAKLSGTGEQLYTVITNNHPSAATMLKAYRQYVDCCPYIKLSHFFETKMTLDAFEGATRVHVVHYGI

QYGVEWPSLIQHLSKRPEGPPYFRITGVDVPYPGDDPCWKIHQTGRRLAEFAKMWNVPFEFHALAGKWESFTAKDFNLRSDEVLAVTSHKMHNILDESVL

GSSPRELLLRRIRSLNPKLFFIIVDNAACNGPFFMTRFRESVKHYSAIFNGMELSFPEDDPDRVVLEREIFGREILNIVACEGQARVDRQEPYRQWQNRL

QRAGFKQVQPKKIILSKMKAMMATFHKDYGVGIDEGWFLLGIKNQIVKANSCWESKPVLNFMS

>Pp1s359_34V6

MTEKGKTGGSLTPGRENSSSSLESLLQQALLQSQRPKLQRRSLSAPVVDVSAEKRGRSLSPHRASHVDGLRGSYFHKSRLEVSSSNFLERFAPPKLETIS

KKTDVPWSSSSGVLGSGRAALGVNYYASQDSRSSTFSTGDSLSSDTQPSATEGIFDAVHFEGNSSGSGDLAGQTESMTRFIDDILSNEDLDEEKSLMQQS

GAFHAMVKEIAGVLAPDSGVDGIDDPVVVEADYLNDWAYAAFFSTDELRMAEKSRSCQAMAREIAGVLSPEANIPDIGEVEGFNDQNNNISEFIPTEDNT

TMKLMEGYTCMLSKHITDVSGARNSDNVDYRSFITESDSLMVHFDDVFRYHLDNSNRKPTMKSDFDFNPLLRGLGGSNSSPPCATLTELLYRCAFAVSQG

KTHSATEYLAELRSLSSPFGDYMQRVAHYFMEALVAKMSGTGEQLYTVITNNHPSAATMLKAFRQYVDRCPYIKVGHFFETKMTLDAFEGATRVHIIHYG

IQYGVEWPTLIQHLSKRPEGPPHFRITGVDVPYPGEDPCWKIEQTGRRLAEFAKMWNVPFEFHALAGKWESFTARDFNLRSDEVLAVITHRLHNILDVSV

LGASPRELLLRRIRSLNPKVFFMFVDNAACNGPFFMTRFRESVKHYSAIFNGMELSFPIDDPERVILEREIFGREILNIVACEGQARVERQEPYRQWQNR

LQRAGFTRVHPKQILLSKMKAMMATFHKDYGVGVDDGWILLGIKNQVVRANSFWEPKPVMSFIS

>Pp1s1_711V6

MTNIMARPDGNPYFFAQHQHSPGFDPESRQHQSSPREWYSSNLQTHSPVSVGARDEICNVYASSSQNPADSTQSWAKALLLDCARAIAEKDTSRVQSIMW

ILNESASPYGDSDQRLMSYFVQALVCKITDTGSRCHRSLTSAAEKTYSFESMRNMILNFQNASPWTTFGHVAANGALLETMEGESKIHIIDISSTLCTQW

PTFLEALATRTDITPHLRLTCIVISPEEAALRVMKQVMNRIDRFARLMGVPFESTVIHKPHLETLDLDELNLREGEALAVNCVQTLHHISECVAAEEQYS

PRDRILSTFRSAKPKILSIVEDEANMISPDFLGCFREALRFYSLLFESLEESFPRASNERLMLERNCARKLVNMLSFDATESTERQEKGIHWDYRLRKVG

FEPVSFSHDVVDDVQALLKRYKKGWGLDITDARLYLTWKEQAVICSTTWKLITTPSY

>Pp1s97_39V6

MIFVKVKDEVMDKLLVKYQEYPQTDQIADLNTSDEAGPTRSSEDSIFEEPRHSARSQLGSGVVSTSLAARRTPSSEGHQYPNLQSEESRQSFPSFRLEQH

PHEFDSEPRQLHDQSPRPEWQYYASPVKPSSGKGDLDGNSMIAGFTGLPSEPYRSSTSQTTESSNSSSHQWAPNLLVECARAITANDSARVKNLMWVLNE

LGSPYGDADQRVAAYFLQALFCKITNTGSSCYRALTAAAERTYSFDTLRKMILDYQEASPWTTFGHTAGNGAMMEAFEGETKIHIVDMSSTYCTQWPILF

EALATRAEGTPHLRLSTIVISPEESALQVMKQIMTRLERFARLMGVPFEYVVKHEPQLEKLELAALDLRQDEVLAITCNHTLHHVSEIVPRGEQYSPRDV

LLCTFRNANPKIMILVEEEVDLTSPDFIVCFCEALKFYSLLFESLEENFPRTSNERLILERICARNLVNLIGCDPPENVERQETGIQWDLRLKRIGFVPC

PFSDDVVDDVRALLKRYKEGWSLSMNENRLYLAWKEQVVLCATAWKPIASPSPEP

>Pp1s181_36V6

MLGSRKLEGPSQENSAHISPLESLLHRAWLRTHKPKLRKSITSAPPTDVSVGKRESSQASLPVLSELVSGQVSNEDGQDQLYFHKSRLDASASFISRFVL

PELKCYSQKTYIPKSVSTEQWIYRLPGVRYGLSQGLKSLSGSSTGLSRNRSSTVSQVSGVTQSEVSGSDSVDSEEHTQPLASYLEGILTEENLEEENTML

CKTSAYQGIAKEISDLIGLEVPTDPDGSDVNSDRHWADVESHYTEEELRIVEKSSAYEAMTKEIAELISPPHSDVHDALSAESSNEVYVSKSDFKPTMEL

AARKMIEAYTCMLSKHMADMSINTDNEDSKNRVANTDGLNSSLLIYFDEDWRIYLHNLNQNFTPSSNFDFNMLLCGASLGSKTLEDNAVATTSGGSLTEL

LYKCALAVSQGDARNATDLLAELRLKSSNQGNPTQRMAHYCMEALVARMSKTGEQLYNVIMNSGPSDARLFKAIRLYLENCPYIKLAHFFAIKALLDACE

GATRIHLVCYGICYGVEYPSFIQQLSLRGGKLPHLRMTVIDSASPGDDPASKLHETGRRLTAFAKDVNLPFEFVGLAGNWESFTARDMNLRDDDVLLVYS

VGLHRLLDASVVASSPREVVLRRIRSINPKVFVMVTLNGGYNAPFFMTRVRECVKFFSAMYEGMEMCMPRDDPDRIIIEREIFGLEIMNIVACEGRTRVE

RAEPYRQWHNRLQRIGFTQLPLNPIVYSKITSMMSAYHKDYGVGEDNGWFLMGIRNQIIKCCSAWEAKTSVSLRPIL

>Pp1s36_131V6

MEHQKGLSNDTDSKTSNASSKAEHESTGFSATPDKRLSSSVFDASLDASQLDLDSHLDALRSQNCRMDNNPLGSDCDILWNQMQLQSHKMMPLSCLASDQ

RPRSIMSRDRFGISSSIENYTDSSLFRLSSNLGGAPQSIVKPELLWNKCEEFAHDAFTESTTSPGEMASKFLNMQTSRQGYMPVQIASSSGTHAEAEVNP

PAEKKMRSMMRMPIVGKQEYSLDPIQCPQEPGQVPIFQGEALESFMLSNTTKYILPPQSAELEKQQRRRSEEKAKRIQVLGVRGVGSVMGTRELNTMEQL

LVQCATALEVSDITYAQQTIFVINNIAAADGDPNQRLLAHFLRALILRASKFTPHLLPGNDNPHTKSRKLKTVLELTNYIDVMPWYRFGFIAANGAILEA

FEGKEKVHILDLNISHCMQWPTLIESLAERNEGPPQLRLTVCVSKAPIPPLLDVPYDELIIRLAKFARSKNVPFEYQLLFEDIEKLDVSKIGIREGEVLA

VNCLFRLHYVTDECTELSTLSPREEVLYFIRKLNPAIVTLTEDDASLTSPKLVTRLKAAFNYFWIPFDALHTLLPKECQQRLHCEDEVANKIENLIACEG

KHRIERVEAKDRWVQRMKRARFHMVSFSEDVVTENKLMLGEHSGCWGLRKDEDEDVLFLTWKGHNVSFSTAWLPANPLLPLR

>Pp1s130_153V6

MPGMIKGETSNSISHSPSLSVSTTSFSSLSHVSGHQSSPSTASTPAQLTWLQDLRSEERGLYLIHLLLACAHAVANNNMEYTNAYLEQISVLASLTGDPM

QRVATYFMEGLAARITKSWPGLYKALHSTHLPSVMDIISARQVFFSVCPYVKFAFLMGNQAILDAMEGEKVVHIVDLEASDPVQWLALLEELSVRKEGPP

HLRITGVSLKKDVLEQTGQRLSEEAEKLDIPFQFHPLVASLEKLDVDSLKVKSGEAVAISSMMRLHPLLAKETDAVLRTENDNHNHPVIRHLRSSSGGYE

RITYVSRDEAINTAGEVGSDRGLKRSREMYEAAPEVGAETLKNHVDASEAGVRQQLPRSMSLSDGNGTNVESRGKPSMVHLPRDESSCSESGFGSGVVQR

ILQKLQCLSPKVMVLVEQDSNHNSGSMPERFVEALHYYSAVFDSLNLTLPQHCLERVTLEKFLLGQEIKNIVACEGAERVERHEKIDRWRMRMRSAGFVS

RPLSSTAALQAKRLLHGYPCDGYRVKDDQGCLTLCWQDTTLYTASAWTV

>Pp1s144_114V6

MPGVSAARAERLLVLCATAIQNQNAPVVQQAVQALQSISSIDGEPNERVTAYFLRALSIRAGGLLQGQLDEDPTGHLDHPHQVQWGDRRLGFNELTNLVD

MTPYYRFGYMAANGAILEALEGVDRVHIIDFSTSHCMQWPTLIDALADRMGGPPHVRLTVASGSLPTPPRLQPTYEEVGHRLALWAGEKKVPFEFRILSR

PLERLRTKDIDLRDGESLAVNCSLRLHYLADESAGFVSEASSETIFSPRDKFLQLIRGLNPTVVTLYEEDCNTTSVDLVTRLKEAYNHEWISFDYLATYS

QNGSHGRLELERAVGQKIENIIACENFHRIERLESKSQWAQRMQRLNFRALPVSEDVVAALREMVGDYAVGWGMKLDEDDVQVLSWKGHSLAFASSWVPY

ETPSKLA

>Pp1s84_112V6

MATMEKEESANIYIDHEDLFRVPPSSNCDQSILSMTNNWGDERLTQWFDGAIDVEGTPGLGLDLNLAVEDEDVQDMAYSDRLWSGIDEPIVLSEGRTRVN

YYLAASTVELDVPEPNDSISAADMLRLPIIHGSYCAPGANSLNKYTAWSEMPSDAALESVSDMHYGSNSDGSTRPTQGLEDLFDAEEDHQLEIMSNDADS

IELTMEILSDGVMSVSLEEESDDDSLQELIAYRRSSSSSRHEFEYELMQSPIRPGTGFTARKKMMHEWVNQGESTFIVPDNIKGADPEEKITIGNGPHDI

LQPREMLSQPGDSRWAEQLLNLCAGAIASKNIGRTQHLMWVLNELASFTGDGNQRLAAYGLKALFCRITGGKEASATYIRPFHHQEKTLGPKAVHRALVT

FHEFSAWHQVAYTVTNETLLEVFAGKSHLHIVDVGIIKGLQWPILIDALSNRPGGPPTKLRITTIRHQNATAKTTGSKQVDAESADFMSRLVTFAKVLGL

HCELNMYVGPLENIKKEDLKLEDGEVLAVCCQFRLHRLSNLVPKSSRHSPTPHLSPRDAFLDFLSSLKPSVLVVSENDADMLSENFLTRFKEIINFWWTF

FDSTHIAFNGREPEAQQIVEYEGSMIMLNGIACEGVERIERNDRQDNWMSRIRRAGFVPMCISEDTKKTVQVLLQNTASVHWSVRYSQNTNCVNMSWKDQ

PVNFTSLWKAPSCSRKLCKCNMLHD

>Pp1s80_27V6

MIKGETSNSISHSPSLSVSTTSFSSLSHVSAHQSSSSSTSTPAQLTWLQELRSEERGLYLIHLLLACANAVANNNMEYTNVYLEQLSVLASLTGDPMQRV

ATYFMEGLAARITKSWPGLHKALHSTHLPFVMDIISARQLFFSVCPYVKFAFLMGNQAILDAMEGEMVVHIVDLEASDPVQWLALLQELSNRQAGPPHLR

ITGVSLKRDVLEQTGQRLSEEAEKLDIPFQFHPLVASLENLDVDSLKVKSGEAVAISSMMRLHPLLAKESDTVIRTDTDYHSHPEIHHFRSSSGGYEKIT

SAGRDEAINSTGEIGPERGLKRSRETYEAAAEVAGENLKVPVDGSEAVDRQRTRSTSQSDGNGSCVEPRGNSVPRPLRDESSFSDSGFGSGVVQRVLQKL

QSLSPKVMVLVEQDSNHNSGSMPDRFVEALHYYSAMFDSLDLTLPQHCLERVTLEKFLLGQEIKNIVACEGAERVERHEKLDRWRIRMRSAGFVARPLSS

TAALQAKRLLHGYPCDGYRVKDDQGCLTLCWQDTPLYTASAWTV

>Pp1s197_153V6

MKRRYKTGALRVDAHSYFSVQTTQGFTSLIRLMKPEAPVPLTSQGHRAGGNEHRHLASNSMTMFGSNAWDRATQHPTDLVGDNLHQGGATRHWVSPYSDP

QLEYVYLGTPHQGSPQTSQVASPQRWPMPDATLNPSLAPPASNNVQYFQPTSGHPTSGQPTSGHAYIDHNGYNAAPTGTAQPSYTMPSSHFQLSQFQTVQ

RFGGVLSQETYEGPAVSELQYDNIWSISESNFLAQRSPQFNVAPGDIDMVEGYGLNSSANMQTPMASQLQQGGSVGNSTGNWTMLSQSPVLDDVGSSSSG

QGAYNSTITGQTSMGGDNQGFLHDHHHSEGSRDCDRALFQLGGMPLPNVGYGETVMNPRLDYMNIVMEDTVSDGVSSTSTVDNRSSRDARAQTPYGHDQS

GQGSSPLDSSVREQFQHNHQQRLEHREAYNTSARSDYNGQATAMDNDFTQLIASGFSGIRHLGEPRWAMQLLNLCAAAIASRNISRTQHLMWVLNDLASV

VGDANQRFAAYGLRALFCRITGRMEAASTFLRPRHYDQEISFGPKTVHRALVKFHEYVPWHQICYTAACQILMEVCAGKARLHLIDIGAGKGIEWPIFID

ALVSRPGGPPAILKITMIRDQRREELNMRTAKSVNSEAADLMTRLVKFAGLVGLHVEVNVVTKALECVTREDLKIRDGETLAAVCQFRIHRLSEEVPDRA

TKSNPTSRPLLSPRDDFFDFLFSLKPDVFIMSDNDSDHCSHDFLTRFQNAISFWWRCYESMDIGYNGRDSEERQIIEYEGGMMVLNMVACEGFARIERNE

SYPQWQRRITRAGFVPQNLSDETKKVCQTLISNHSEFWELSFTDSNVVNLLWRKQPTTFTSVWKMPQSYSSSSSCQSTLIHN

>Pp1s72_74V6

MKPWLFLPCSRGVGASMVHAFDACARFLCASTDASRVQVVRLIPHDIIGSIMFHTSSTRCSFSTIAQSAVGGLVAGCREGRRKNITLSLQRYRISCGSNR

HSKLVSKLFSKLADITPSGWPSAGSVSIQHNAGVDAGPLPIANMGYSSSSILLSSDESDGRFAVGHSSHPGDGSVYGSASSNLAMFAKDMGGAGQHIMDM

SRGNLDRRGAAQTWVNPYSNPEFEYVYLGSPQTSQDSQTPLILDPGMSSVSSGSQVLQPPTGNVHYNASYGRTSQSKYAVQPSSNFQGHLHPAQQFSAPP

MHRHKAPEMHTPFSQEFYNVQDEIDMQSYEDIWRNLESDFLAQTSAQYDATPGDINMVDAVYNVSQSTPGIPFPVPNQTQPVSRPIADQKTWNMLSNSVQ

NEAESSSNVRTRYGNASSSTRHPFGFNPPRHFAEQAQPHQRSEVAQNRDLSFHQSVMETRPDFMDMVMDDSVSDGISITGSSDNQLRHAQSPNYGFGSHG

YAGTASNFGERQHSAVQQSFQPENNSREQMQTNHQYNRFPEHREASFSSGGDLYNETKSGVESDFSTLMAPGQSGLRSLGESRWAEQLLNLCAAAIASKN

ISRMQHLMWVLNDLASVVGDANQRYAAYGLRALFCRITGRMEAAKTYLRPRHYDQEQSYGPKMVHRALVKFHEHVPWHQNCYSVASQTLLEVCAGKSRLH

IIDIGAGKGIEWPIFIDALVSRSGGPPSILRMTMIRDLQREEPNLRTRGGSEAADFMTRLVKFASLLGLHVEVNMVRKPLECVTREDLKLRDGEILAVVC

QFRLHRLSEELDYQSSPPDLSPRDKFLDFLFTLDPHIFIQSDNDSDHCSQDFLTRFQNAVSFWWRCFESIDVGYNGRDPDERQIIEYEGAMMLLNMVACE

GIARIERNESYPQWARRIKRAGFMPRELSEETKKVSQNLVANHSEFWETAFIESNMVSLLWRKQPTTFTSVWKSSSCTNPKHSCKCSKLHN

>Pp1s117_143V6

MFGNDVIGGGQQVMDLSGGNPIQCGAAQPWVNPYNNPEPEYVYLVSPQNSHTLSENGYSNLSCGPVPQPNYMVRPDSNFLPQLNPVQQFSAPHMDYRYLP

PAQPPVSHEPYGTLSDNNLQQYDNVWQSLESDFLAQTSVQYNDVPGDNNMIEVVHGFHDPPAHISISVSNEVPLTSRPVADHRAWTMPSHPIPGVAESSG

SVPAEYGNTSSPTSTRQSQEFNPSHGFAGQGQVPSDTVSTTGTGGNHAAAQRSSRPKSNFEDQMQTSHQQYRKVLDDGEVACASGGSSYNESDPTVDNDL

STLMAPGRSGLQSLGESSWAVQLLNLCAAAIESQNVSRTQHLMWVLNDFASVVGDANQRIAAYGHRALFCRITGSLEAAKTYLRPKHYDQELSLGPKMVH

RALVKFHEHVPWHQNCYSIMSQTLLEVCAGKSRLHIIDIGAGKGIEWPIFIDALVSRSGGPPSILRMTMIRDLQREEHNLRSQLNSESSDFMTRLVKFPG

LLGLHVEVNMVRKALECVTQELLAVICQFRLHRLSEEPDTNCLPPRLSPRDKFLDFLFQLNPHVFIQSDNNCDHCSQDFLTRFRNASAFWWNCFESIVFD

YNGRDPDGRQINMASLQWRNPPTTFTSVWKTPSTCLKTSCKYSKLHS
