# Supplementary material for: Unusual tandem expansion and positive selection in subgroups of the plant GRAS transcription factor superfamily
Source: BMC Plant Biol. 2014 Dec 19;14:373. doi: 10.1186/s12870-014-0373-5 (PMC4279901; doi:10.1186/s12870-014-0373-5)
Supplement: Additional file 3: — Predicted AtGRAS genes and related information. a.aa = amino acids; b. pI = isoelectric point of the deduced polypeptide; c.Mw = molecular weight; d. the relative position of introns are indicated by the red square. [file 12870_2014_373_MOESM3_ESM.doc]

**Additional file 3. Predicted AtGRAS genes and related information.**

| Group | Gene ID | Other  name | Chromosome | ORF(aa)^a^ | pI^b^ | Mw(KD)^c^ | Gene structure^d^ |
| --- | --- | --- | --- | --- | --- | --- | --- |
| 1 | AT1G21450 | SCL1 | 1 | 593 | 5.01 | 66.7 | 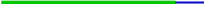 |
| 1 | AT1G50600 | SCL5 | 1 | 597 | 5.77 | 66.6 | 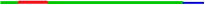 |
| 1 | AT2G04890 | SCL21 | 2 | 413 | 5.53 | 46.5 | 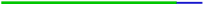 |
| 1 | AT4G17230 | SCL13 | 4 | 529 | 5.58 | 58.5 | 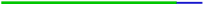 |
| 1 | AT5G48150 | PAT1 | 5 | 490 | 6.17 | 55.3 | 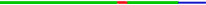 |
| 1 | AT5G52510 | SCL8 | 5 | 640 | 5.12 | 69.2 | 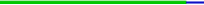 |
| 2 | AT1G07520 | SCL31 | 1 | 695 | 5.44 | 79.3 | 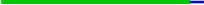 |
| 2 | AT1G07530 | SCL14 | 1 | 769 | 5.56 | 85.9 | 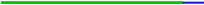 |
| 2 | AT2G29060 | SCL33 | 2 | 694 | 5.75 | 78.1 | 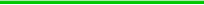 |
| 2 | AT2G29065 | SCL34 | 2 | 630 | 5.43 | 72.5 | 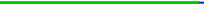 |
| 2 | AT2G37650 | SCL9 | 2 | 718 | 5.99 | 80.9 | 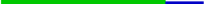 |
| 2 | AT3G46600 | SCL30 | 3 | 583 | 5.55 | 66.6 | 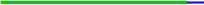 |
| 2 | AT5G59450 | SCL11 | 5 | 610 | 5.82 | 69.8 | 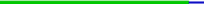 |
| 3 | AT3G13840 | SCL29 | 3 | 510 | 5.28 | 57.2 | 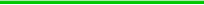 |
| 3 | AT3G49950 | SCL22 | 3 | 410 | 5.75 | 46.4 | 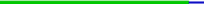 |
| 3 | AT4G37650 | SHR | 4 | 531 | 5.44 | 59.5 | 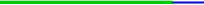 |
| 4 | AT1G14920 | GAI | 1 | 533 | 5.37 | 58.9 | 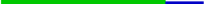 |
| 4 | AT1G66350 | RGL1 | 1 | 511 | 5.45 | 56.8 | 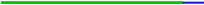 |
| 4 | AT2G01570 | RGA | 2 | 587 | 5.51 | 64.0 | 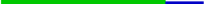 |
| 4 | AT3G03450 | RGL2 | 3 | 547 | 4.82 | 60.5 | 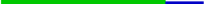 |
| 4 | AT5G17490 | RGL3 | 5 | 523 | 4.76 | 57.3 | 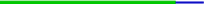 |
| 5a | AT3G54220 | SCR | 3 | 653 | 5.82 | 71.5 | 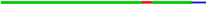 |
| 5a | AT5G41920 | SCL23 | 5 | 405 | 5.68 | 44.9 | 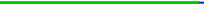 |
| 5a | AT1G50420 | SCL3 | 1 | 482 | 6.00 | 54.2 | 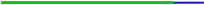 |
| 5b | AT1G55580 | LAS | 1 | 445 | 6.11 | 50.0 | 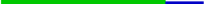 |
| 6 | AT1G63100 | SCL28 | 1 | 658 | 6.49 | 73.5 | 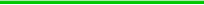 |
| 6 | AT3G50650 | SCL7 | 3 | 542 | 5.08 | 60.8 | 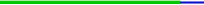 |
| 6 | AT5G66770 | SCL4 | 5 | 584 | 4.68 | 64.6 | 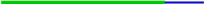 |
| 7 | AT2G45160 | SCL27 | 2 | 640 | 5.41 | 70.2 | 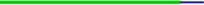 |
| 7 | AT3G60630 | SCL22 | 3 | 623 | 4.78 | 68.1 | 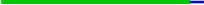 |
| 7 | AT4G00150 | SCL6 | 4 | 558 | 5.45 | 61.2 | 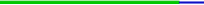 |
| 7 | AT4G08250 | SCL26 | 4 | 483 | 5.18 | 53.5 | 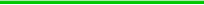 |
| 7 | AT4G36710 | SCL15 | 4 | 486 | 5.20 | 54.1 | 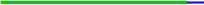 |
