# Supplementary material for: Unusual tandem expansion and positive selection in subgroups of the plant GRAS transcription factor superfamily
Source: BMC Plant Biol. 2014 Dec 19;14:373. doi: 10.1186/s12870-014-0373-5 (PMC4279901; doi:10.1186/s12870-014-0373-5)
Supplement: Additional file 4: — Predicted BdGRAS genes and related information. a.aa = amino acids; b. pI = isoelectric point of the deduced polypeptide; c.Mw = molecular weight; d. the relative position of introns are indicated by the red square. [file 12870_2014_373_MOESM4_ESM.doc]

**Additional file 4. Predicted BdGRAS genes and related information.**

| Group | Gene ID | Chromosome | ORF(aa)^a^ | pI^b^ | Mw(KD)^c^ | Gene structure^d^ |
| --- | --- | --- | --- | --- | --- | --- |
| 1 | Bradi1g23350 | 1 | 549 | 5.89 | 61.2 | 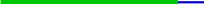 |
| 1 | Bradi1g25370 | 1 | 571 | 6.35 | 64.6 | 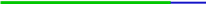 |
| 1 | Bradi2g56910 | 2 | 571 | 4.90 | 63.9 | 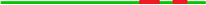 |
| 1 | Bradi3g24210 | 3 | 541 | 5.62 | 60.4 | 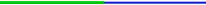 |
| 1 | Bradi5g19190 | 5 | 631 | 8.60 | 66.8 | 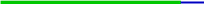 |
| 2 | Bradi1g03620 | 1 | 659 | 5.89 | 73.7 | 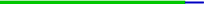 |
| 2 | Bradi1g15123 | 1 | 589 | 8.89 | 67.2 | 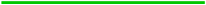 |
| 2 | Bradi2g52227 | 2 | 765 | 4.95 | 84.2 | 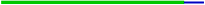 |
| 2 | Bradi2g54670 | 2 | 805 | 5.84 | 88.9 | 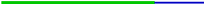 |
| 4 | Bradi2g57940 | 2 | 531 | 5.80 | 57.4 | 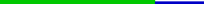 |
| 2 | Bradi4g03867 | 4 | 738 | 5.20 | 81.5 | 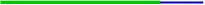 |
| 2 | Bradi4g09155 | 4 | 605 | 8.16 | 68.6 | 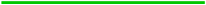 |
| 2 | Bradi4g09160 | 4 | 640 | 5.59 | 71.6 | 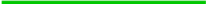 |
| 2 | Bradi4g09170 | 4 | 642 | 5.87 | 72.2 | 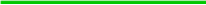 |
| 2 | Bradi4g09180 | 4 | 631 | 6.31 | 69.6 | 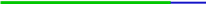 |
| 2 | Bradi4g09190 | 4 | 637 | 5.75 | 70.7 | 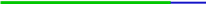 |
| 2 | Bradi4g09197 | 4 | 767 | 5.18 | 84.4 | 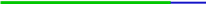 |
| 2 | Bradi4g09235 | 4 | 634 | 7.26 | 71.5 | 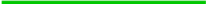 |
| 2 | Bradi4g43680 | 4 | 784 | 6.56 | 87.4 | 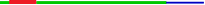 |
| 3 | Bradi1g22907 | 1 | 484 | 6.12 | 59.9 | 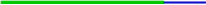 |
| 3 | Bradi1g23060 | 1 | 593 | 5.7 | 64.2 | 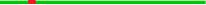 |
| 3 | Bradi1g60140 | 1 | 523 | 9.57 | 55.7 | 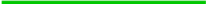 |
| 3 | Bradi2g20760 | 2 | 420 | 6.15 | 44.3 | 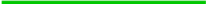 |
| 4 | Bradi1g11090 | 1 | 623 | 5.03 | 65.6 | 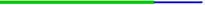 |
| 4 | Bradi1g32070 | 1 | 421 | 7.41 | 46.7 | 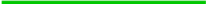 |
| 4 | Bradi1g47900 | 1 | 416 | 6.09 | 45.6 | 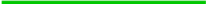 |
| 4 | Bradi2g45117 | 2 | 504 | 5.18 | 53.1 | 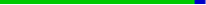 |
| 4 | Bradi4g18390 | 4 | 739 | 5.94 | 80.0 | 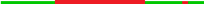 |
| 5a | Bradi1g24310 | 1 | 465 | 5.29 | 50.0 | 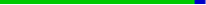 |
| 5a | Bradi2g22010 | 2 | 495 | 5.49 | 51.9 | 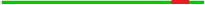 |
| 5b | Bradi2g60750 | 2 | 465 | 5.9 | 50.1 | 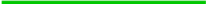 |
| 5b | Bradi4g43200 | 4 | 538 | 7.04 | 58.1 | 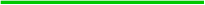 |
| 5b | Bradi1g36180 | 1 | 494 | 9.17 | 52.3 | 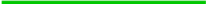 |
| 5b | Bradi3g07160 | 3 | 409 | 5.49 | 43.4 | 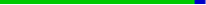 |
| 6 | Bradi1g10330 | 1 | 541 | 5.01 | 58.4 | 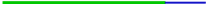 |
| 6 | Bradi1g49630 | 1 | 631 | 5.96 | 67.0 | 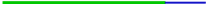 |
| 6 | Bradi5g10320 | 5 | 404 | 6.57 | 49.5 | 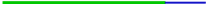 |
| 7 | Bradi1g52240 | 1 | 537 | 5.76 | 55.9 | 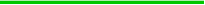 |
| 7 | Bradi1g67340 | 1 | 576 | 5.29 | 60.8 | 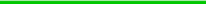 |
| 7 | Bradi1g78230 | 1 | 620 | 5.84 | 65.3 | 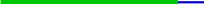 |
| 7 | Bradi3g32890 | 3 | 653 | 5.59 | 67.9 | 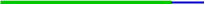 |
| 7 | Bradi3g50930 | 3 | 724 | 5.89 | 61.2 | 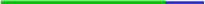 |
| 7 | Bradi4g24867 | 4 | 468 | 6.35 | 64.6 | 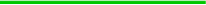 |
| 7 | Bradi4g41880 | 4 | 480 | 4.90 | 63.9 | 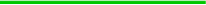 |
