# Supplementary material for: Unusual tandem expansion and positive selection in subgroups of the plant GRAS transcription factor superfamily
Source: BMC Plant Biol. 2014 Dec 19;14:373. doi: 10.1186/s12870-014-0373-5 (PMC4279901; doi:10.1186/s12870-014-0373-5)
Supplement: Additional file 5: — Predicted OsGRAS genes and related information. a.aa = amino acids; b. pI = isoelectric point of the deduced polypeptide; c.Mw = molecular weight; d. the relative position of introns are indicated by the red square. [file 12870_2014_373_MOESM5_ESM.doc]

**Additional file 5. Predicted OsGRAS genes and related information.**

| Group | Gene ID | Chromosome | ORF(aa)^a^ | pI^b^ | Mw(KD)^c^ | Gene structure^d^ |
| --- | --- | --- | --- | --- | --- | --- |
| 1 | LOC_Os01g65900 | 1 | 553 | 4.80 | 61.8 | 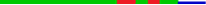 |
| 1 | LOC_Os02g45760 | 2 | 618 | 9.27 | 64.2 | 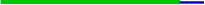 |
| 1 | LOC_Os03g09280 | 3 | 535 | 5.86 | 59.6 | 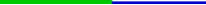 |
| 1 | LOC_Os04g49110 | 4 | 619 | 7.20 | 64.9 | 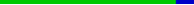 |
| 1 | LOC_Os07g36170 | 7 | 571 | 5.87 | 64.6 | 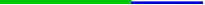 |
| 1 | LOC_Os07g39470 | 7 | 544 | 6.00 | 60.1 | 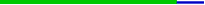 |
| 1 | LOC_Os10g22430 | 10 | 541 | 5.79 | 59.9 | 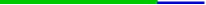 |
| 2 | LOC_Os01g62460 | 1 | 705 | 6.10 | 79.0 | 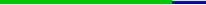 |
| 4 | LOC_Os01g67650 | 1 | 532 | 6.09 | 57.4 | 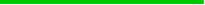 |
| 2 | LOC_Os03g48450 | 3 | 731 | 6.44 | 82.2 | 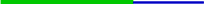 |
| 2 | LOC_Os04g50060 | 4 | 636 | 5.51 | 71.7 | 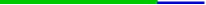 |
| 2 | LOC_Os11g47870 | 11 | 692 | 5.01 | 77.4 | 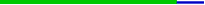 |
| 2 | LOC_Os11g47890 | 11 | 638 | 5.27 | 71.5 | 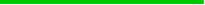 |
| 2 | LOC_Os11g47900 | 11 | 642 | 6.26 | 72.1 | 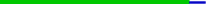 |
| 2 | LOC_Os11g47910 | 11 | 595 | 5.99 | 66.7 | 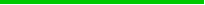 |
| 2 | LOC_Os11g47920 | 11 | 593 | 5.98 | 66.6 | 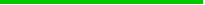 |
| 2 | LOC_Os12g04200 | 12 | 585 | 5.23 | 64.0 | 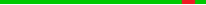 |
| 2 | LOC_Os12g38490 | 12 | 738 | 5.17 | 81.4 | 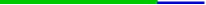 |
| 3 | LOC_Os03g31880 | 3 | 603 | 5.93 | 64.2 | 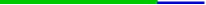 |
| 3 | LOC_Os05g42130 | 5 | 425 | 5.81 | 45.4 | 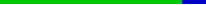 |
| 3 | LOC_Os07g39820 | 7 | 602 | 5.61 | 64.7 | 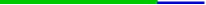 |
| 3 | LOC_Os07g40020 | 7 | 473 | 5.31 | 50.6 | 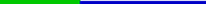 |
| 4 | LOC_Os01g45860 | 1 | 495 | 5.06 | 52.1 | 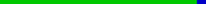 |
| 4 | LOC_Os03g49990 | 3 | 625 | 5.14 | 65.4 | 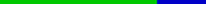 |
| 4 | LOC_Os05g49930 | 5 | 500 | 5.91 | 53.0 | 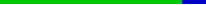 |
| 4 | LOC_Os11g31100 | 11 | 772 | 6.18 | 81.1 | 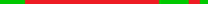 |
| 5a | LOC_Os05g40710 | 5 | 493 | 5.75 | 51.6 | 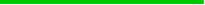 |
| 5a | LOC_Os07g38030 | 7 | 457 | 5.51 | 49.1 | 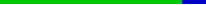 |
| 5a | LOC_Os11g03110 | 11 | 651 | 5.91 | 69.9 | 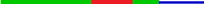 |
| 5a | LOC_Os12g02870 | 12 | 660 | 5.91 | 70.4 | 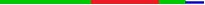 |
| 5a | LOC_Os01g71970 | 1 | 442 | 6.15 | 48.0 | 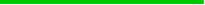 |
| 5a | LOC_Os05g31380 | 5 | 551 | 5.41 | 56.3 | 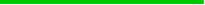 |
| 5a | LOC_Os05g31420 | 5 | 560 | 7.85 | 58.2 | 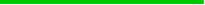 |
| 5a | LOC_Os11g04590 | 11 | 460 | 6.92 | 50.4 | 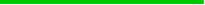 |
| 5a | LOC_Os12g04380 | 12 | 464 | 5.99 | 50.7 | 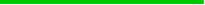 |
| 5b | LOC_Os02g10360 | 2 | 423 | 5.56 | 44.1 | 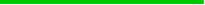 |
| 5b | LOC_Os06g40780 | 6 | 666 | 5.76 | 69.8 | 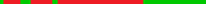 |
| 6 | LOC_Os03g51330 | 3 | 578 | 5.63 | 62.5 | 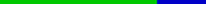 |
| 6 | LOC_Os04g35250 | 4 | 504 | 8.67 | 52.6 | 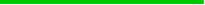 |
| 6 | LOC_Os06g03710 | 6 | 617 | 6.02 | 65.8 | 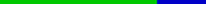 |
| 7 | LOC_Os02g44360 | 2 | 709 | 5.65 | 74.2 | 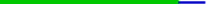 |
| 7 | LOC_Os02g44370 | 2 | 715 | 5.82 | 74.1 | 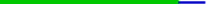 |
| 7 | LOC_Os03g15680 | 3 | 575 | 5.04 | 60.8 | 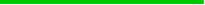 |
| 7 | LOC_Os04g46860 | 4 | 711 | 5.57 | 74.0 | 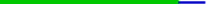 |
| 7 | LOC_Os06g01620 | 6 | 480 | 5.46 | 51.0 | 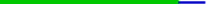 |
| 7 | LOC_Os10g40390 | 10 | 683 | 8.85 | 74.3 | 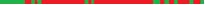 |
| 7 | LOC_Os11g06180 | 11 | 472 | 4.78 | 52.3 | 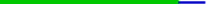 |
