# Supplementary material for: Unusual tandem expansion and positive selection in subgroups of the plant GRAS transcription factor superfamily
Source: BMC Plant Biol. 2014 Dec 19;14:373. doi: 10.1186/s12870-014-0373-5 (PMC4279901; doi:10.1186/s12870-014-0373-5)
Supplement: Additional file 6: — Predicted GmGRAS genes and related information. a.aa = amino acids; b. pI = isoelectric point of the deduced polypeptide; c.Mw = molecular weight; d. the relative position of introns are indicated by the red square. [file 12870_2014_373_MOESM6_ESM.doc]

**Additional file 6. Predicted GmGRAS genes and related information**

| Group | Gene ID | Chromosome | ORF(aa)^a^ | pI^b^ | Mw(KD)^c^ | Gene structure^d^ |  |
| --- | --- | --- | --- | --- | --- | --- | --- |
| 1 | Glyma02g46730 | 2 | 545 | 5.42 | 61.5 | 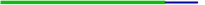 |  |
| 1 | Glyma02g47640 | 2 | 541 | 5.75 | 60.7 | 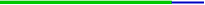 |  |
| 1 | Glyma04g42090 | 4 | 596 | 4.78 | 66.5 | 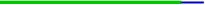 |  |
| 1 | Glyma06g12701 | 6 | 600 | 4.83 | 66.8 | 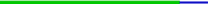 |  |
| 1 | Glyma06g41500 | 6 | 568 | 5.67 | 63.1 | 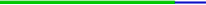 |  |
| 1 | Glyma07g39650 | 7 | 542 | 6.02 | 60.3 | 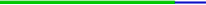 |  |
| 1 | Glyma08g43780 | 8 | 545 | 5.26 | 61.3 | 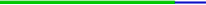 |  |
| 1 | Glyma09g01440 | 9 | 548 | 5.83 | 61.3 | 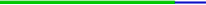 |  |
| 1 | Glyma10g37640 | 10 | 590 | 8.26 | 65.2 | 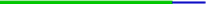 |  |
| 1 | Glyma12g16750 | 12 | 568 | 5.88 | 63.1 | 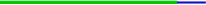 |  |
| 1 | Glyma12g34420 | 12 | 571 | 5.5 | 63.8 | 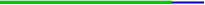 |  |
| 1 | Glyma13g09220 | 13 | 591 | 4.97 | 66.0 | 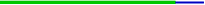 |  |
| 1 | Glyma13g36120 | 13 | 577 | 5.45 | 64.5 | 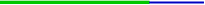 |  |
| 1 | Glyma14g01020 | 14 | 545 | 5.93 | 61.1 | 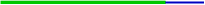 |  |
| 1 | Glyma14g01960 | 14 | 545 | 5.54 | 61.5 | 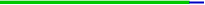 |  |
| 1 | Glyma14g27290 | 14 | 591 | 5.02 | 66.1 | 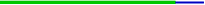 |  |
| 1 | Glyma15g12320 | 15 | 552 | 5.57 | 61.7 | 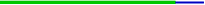 |  |
| 1 | Glyma16g29900 | 16 | 657 | 6.64 | 72.4 | 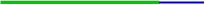 |  |
| 1 | Glyma17g01150 | 17 | 545 | 6.05 | 60.9 | 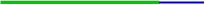 |  |
| 1 | Glyma18g09030 | 18 | 545 | 5.2 | 61.2 | 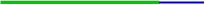 |  |
| 1 | Glyma20g30150 | 20 | 594 | 8.31 | 65.4 | 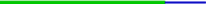 |  |
| 2 | Glyma02g01530 | 2 | 495 | 6.31 | 56.2 | 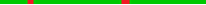 |  |
| 2 | Glyma03g10320 | 3 | 730 | 5.79 | 82.4 | 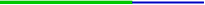 |  |
| 2 | Glyma03g37851 | 3 | 540 | 6.03 | 61.8 | 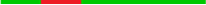 |  |
| 4 | Glyma05g03020 | 5 | 511 | 6.05 | 57.7 | 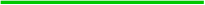 |  |
| 2 | Glyma07g15950 | 7 | 734 | 6.06 | 83.0 | 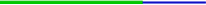 |  |
| 2 | Glyma09g04110 | 9 | 595 | 4.99 | 67.7 | 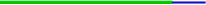 |  |
| 2 | Glyma11g14670 | 11 | 680 | 5.84 | 76.5 | 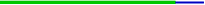 |  |
| 2 | Glyma11g14700 | 11 | 614 | 6.00 | 70.2 | 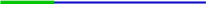 |  |
| 2 | Glyma11g14710 | 11 | 696 | 6.01 | 79.5 | 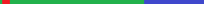 |  |
| 2 | Glyma11g14720 | 11 | 673 | 5.90 | 76.6 | 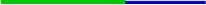 | |
| 2 | Glyma11g14740 | 11 | 571 | 5.32 | 64.8 | 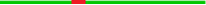 | |
| 2 | Glyma11g14750 | 11 | 742 | 5.53 | 83.9 | 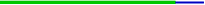 | |
| 2 | Glyma12g06630 | 12 | 687 | 5.74 | 77.4 | 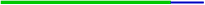 | |
| 2 | Glyma12g06640 | 12 | 680 | 5.42 | 77.3 | 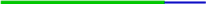 | |
| 2 | Glyma12g06655 | 12 | 676 | 5.68 | 76.8 | 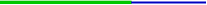 | |
| 2 | Glyma12g06670 | 12 | 660 | 6.18 | 74.2 | 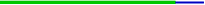 | |
| 2 | Glyma13g41220 | 13 | 644 | 5.86 | 73.0 | 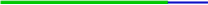 | |
| 2 | Glyma13g41240 | 13 | 743 | 5.71 | 84.0 | 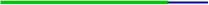 | |
| 2 | Glyma13g41261 | 13 | 657 | 5.46 | 73.7 | 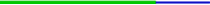 | |
| 2 | Glyma15g04160 | 15 | 684 | 5.5 | 76.8 | 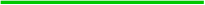 | |
| 2 | Glyma15g04166 | 15 | 606 | 6.21 | 68.8 | 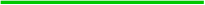 | |
| 2 | Glyma15g04173 | 15 | 727 | 5.72 | 81.9 | 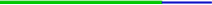 | |
| 2 | Glyma15g04190 | 15 | 665 | 5.88 | 75.1 | 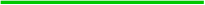 | |
| 2 | Glyma15g15110 | 15 | 593 | 4.82 | 67.4 | 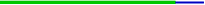 | |
| 4 | Glyma15g28410 | 15 | 549 | 4.88 | 62.1 | 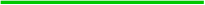 | |
| 4 | Glyma17g13680 | 17 | 514 | 6.05 | 57.8 | 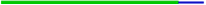 | |
| 2 | Glyma18g39920 | 18 | 733 | 6.06 | 82.3 | 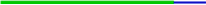 | |
| 2 | Glyma19g40440 | 19 | 559 | 5.88 | 63.9 | 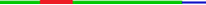 | |
| 3 | Glyma01g40180 | 1 | 476 | 5.45 | 54.0 | 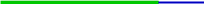 | |
| 3 | Glyma05g22140 | 5 | 480 | 5.65 | 53.4 | 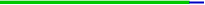 | |
| 3 | Glyma05g22460 | 5 | 499 | 5.28 | 56.8 | 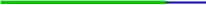 | |
| 3 | Glyma07g04430 | 7 | 542 | 5.62 | 60.0 | 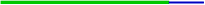 | |
| 3 | Glyma11g05110 | 11 | 482 | 5.45 | 54.5 | 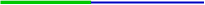 | |
| 3 | Glyma12g32350 | 12 | 460 | 6.13 | 51.5 | 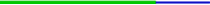 | |
| 3 | Glyma13g38080 | 13 | 467 | 5.98 | 52.2 | 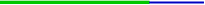 | |
| 3 | Glyma13g42100 | 13 | 443 | 5.58 | 50.0 | 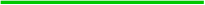 | |
| 3 | Glyma15g03290 | 15 | 437 | 5.27 | 49.4 | 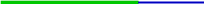 | |
| 3 | Glyma16g01020 | 16 | 543 | 5.79 | 60.2 | 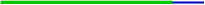 | |
| 3 | Glyma17g17400 | 17 | 503 | 5.41 | 57.1 | 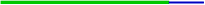 | |
| 3 | Glyma17g17710 | 17 | 482 | 5.73 | 53.8 | 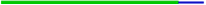 | |
| 4 | Glyma04g21340 | 4 | 503 | 5.00 | 56.0 | 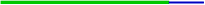 | |
| 4 | Glyma05g27190 | 5 | 523 | 5.28 | 57.9 | 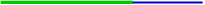 | |
| 4 | Glyma06g23940 | 6 | 505 | 5.05 | 56.3 | 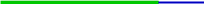 | |
| 4 | Glyma08g10140 | 8 | 517 | 5.21 | 57.3 | 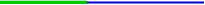 | |
| 4 | Glyma10g33380 | 10 | 488 | 5.28 | 54.2 | 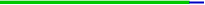 | |
| 4 | Glyma11g33720 | 11 | 595 | 5.00 | 65.0 | 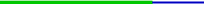 | |
| 4 | Glyma16g05751 | 16 | 687 | 5.73 | 76.3 | 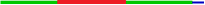 | |
| 4 | Glyma18g04500 | 18 | 584 | 5.16 | 63.9 | 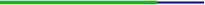 | |
| 4 | Glyma19g26735 | 19 | 681 | 5.79 | 75.4 | 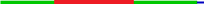 | |
| 4 | Glyma20g34260 | 20 | 434 | 5.71 | 48.3 | 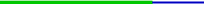 | |
| 5 | Glyma09g40620 | 9 | 823 | 6.05 | 89.6 | 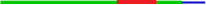 | |
| 5 | Glyma10g04421 | 10 | 537 | 6.01 | 60.4 | 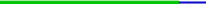 | |
| 5 | Glyma11g10220 | 11 | 442 | 5.52 | 48.2 | 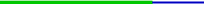 | |
| 5 | Glyma12g02530 | 12 | 445 | 5.92 | 49.0 | 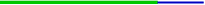 | |
| 5 | Glyma13g18680 | 13 | 526 | 5.98 | 59.1 | 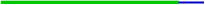 | |
| 5 | Glyma18g45220 | 18 | 664 | 6.02 | 72.6 | 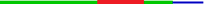 | |
| 5a | Glyma01g43620 | 1 | 465 | 6.04 | 52.3 | 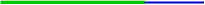 | |
| 5a | Glyma04g28490 | 4 | 443 | 6.53 | 50.3 | 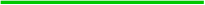 | |
| 5a | Glyma09g35876 | 9 | 421 | 8.97 | 48.0 | 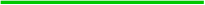 | |
| 5a | Glyma11g01850 | 11 | 473 | 5.71 | 53.0 | 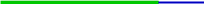 | |
| 5a | Glyma11g10170 | 11 | 455 | 5.66 | 51.5 | 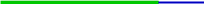 | |
| 5a | Glyma11g20980 | 11 | 442 | 6.13 | 50.3 | 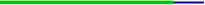 | |
| 5a | Glyma12g02490 | 12 | 455 | 5.64 | 51.4 | 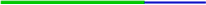 | |
| 6 | Glyma02g08241 | 2 | 476 | 6.27 | 54.8 | 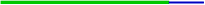 | |
| 6 | Glyma05g03490 | 5 | 664 | 5.99 | 73.8 | 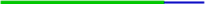 | |
| 6 | Glyma10g35920 | 10 | 465 | 6.18 | 53.0 | 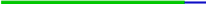 | |
| 6 | Glyma12g02060 | 12 | 481 | 5.11 | 53.8 | 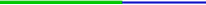 | |
| 6 | Glyma16g27310 | 16 | 471 | 6.79 | 53.9 | 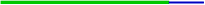 | |
| 6 | Glyma17g14030 | 17 | 669 | 6.31 | 74.7 | 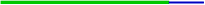 | |
| 6 | Glyma20g31680 | 20 | 462 | 6.26 | 52.8 | 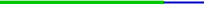 | |
| 7 | Glyma01g18040 | 1 | 744 | 5.48 | 81.6 | 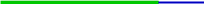 | |
| 7 | Glyma01g33270 | 1 | 737 | 5.76 | 81.4 | 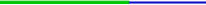 | |
| 7 | Glyma01g38360 | 1 | 525 | 5.45 | 58.7 | 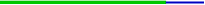 | |
| 7 | Glyma02g06530 | 2 | 523 | 5.91 | 58.2 | 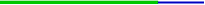 | |
| 7 | Glyma03g03760 | 3 | 703 | 5.74 | 77.9 | 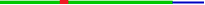 | |
| 7 | Glyma03g06530 | 3 | 523 | 5.13 | 59.6 | 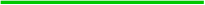 | |
| 7 | Glyma04g43090 | 4 | 502 | 5.75 | 55.2 | 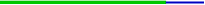 | |
| 7 | Glyma06g11610 | 6 | 495 | 5.86 | 54.3 | 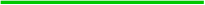 | |
| 7 | Glyma07g18934 | 7 | 558 | 4.90 | 63.3 | 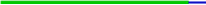 | |
| 7 | Glyma08g15530 | 8 | 487 | 4.76 | 55.7 |  | |
| 7 | Glyma11g06980 | 11 | 516 | 5.56 | 57.5 |  | |
| 7 | Glyma11g17490 | 11 | 745 | 5.89 | 81.8 |  | |
| 7 | Glyma13g02840 | 13 | 472 | 5.05 | 51.4 |  | |
| 7 | Glyma16g25570 | 16 | 540 | 5.87 | 60.3 |  | |
| 7 | Glyma18g43580 | 18 | 543 | 4.84 | 61.3 |  | |
