# Supplementary material for: Unusual tandem expansion and positive selection in subgroups of the plant GRAS transcription factor superfamily
Source: BMC Plant Biol. 2014 Dec 19;14:373. doi: 10.1186/s12870-014-0373-5 (PMC4279901; doi:10.1186/s12870-014-0373-5)
Supplement: Additional file 7: — Predicted SmGRAS genes and related information. a.aa = amino acids; b. pI = isoelectric point of the deduced polypeptide; c.Mw = molecular weight; d. the relative position of introns are indicated by the red square. [file 12870_2014_373_MOESM7_ESM.doc]

| Group | Gene ID | ORF(aa)^a^ | pI^b^ | Mw(KD)^c^ | Gene structure^d^ |
| --- | --- | --- | --- | --- | --- |
| 1 | 77165 | 375 | 7.04 | 40.9 | 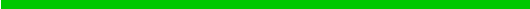 |
| 1 | 444260 | 1011 | 6.09 | 111.4 | 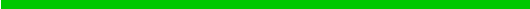 |
| 2 | 74492 | 423 | 6.33 | 47.9 | 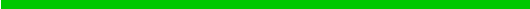 |
| 2 | 232175 | 381 | 8.43 | 43.4 | 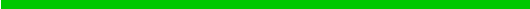 |
| 2 | 80549 | 372 | 8.50 | 41.6 | 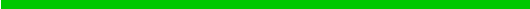 |
| 2 | 142207 | 429 | 6.39 | 47.5 | 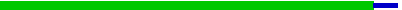 |
| 2 | 424843 | 642 | 5.71 | 69.7 | 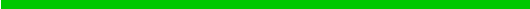 |
| 3 | 113376 | 403 | 6.22 | 45.5 | 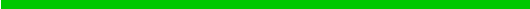 |
| 4 | 139506 | 512 | 5.27 | 55.5 | 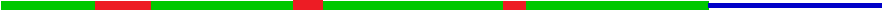 |
| 4 | 122441 | 455 | 5.19 | 50.5 | 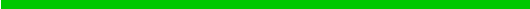 |
| 4 | 83811 | 472 | 5.26 | 52.6 | 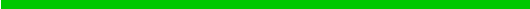 |
| 5a | 85562 | 451 | 5.19 | 48.4 | 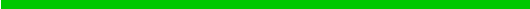 |
| 5a | 84762 | 521 | 5.82 | 56.3 | 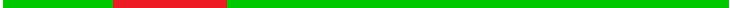 |
| 5b | 102726 | 423 | 7.77 | 47.6 | 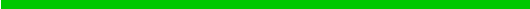 |
| 5b | 113858 | 474 | 8.53 | 52.1 | 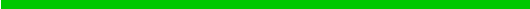 |
| 5b | 84991 | 348 | 5.83 | 39.2 | 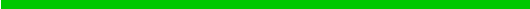 |
| 5b | 84560 | 467 | 5.83 | 51.0 | 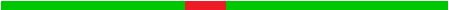 |
| 6 | 88625 | 489 | 4.85 | 54.2 | 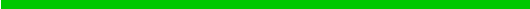 |
| 6 | 122435 | 447 | 5.25 | 48.7 | 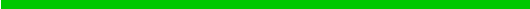 |
| 7 | 96442 | 438 | 5.71 | 49.2 | 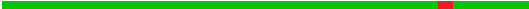 |
| 7 | 83927 | 409 | 6.53 | 46.0 | 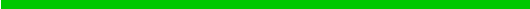 |

**Additional file 7. Predicted SmGRAS genes and related information**
