# Supplementary material for: Unusual tandem expansion and positive selection in subgroups of the plant GRAS transcription factor superfamily
Source: BMC Plant Biol. 2014 Dec 19;14:373. doi: 10.1186/s12870-014-0373-5 (PMC4279901; doi:10.1186/s12870-014-0373-5)
Supplement: Additional file 8: — Predicted PpGRAS genes and related information. a.aa = amino acids; b. pI = isoelectric point of the deduced polypeptide; c.Mw = molecular weight; d. the relative position of introns are indicated by the red square. [file 12870_2014_373_MOESM8_ESM.doc]

| Group | Gene ID | ORF(aa)^a^ | pI^b^ | Mw(KD)^c^ | Gene structure^d^ |
| --- | --- | --- | --- | --- | --- |
| 1 | Pp1s346_13V6 | 657 | 5.49 | 72.2 | 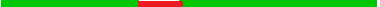 |
| 1 | Pp1s456_3V6 | 648 | 5.61 | 71.8 | 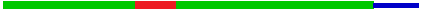 |
| 2 | Pp1s84_299V6 | 774 | 5.47 | 86.2 | 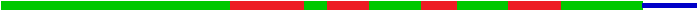 |
| 2 | Pp1s98_14V6 | 816 | 5.43 | 90.9 | 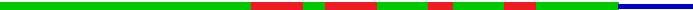 |
| 2 | Pp1s20_86V6 | 727 | 5.70 | 81.8 | 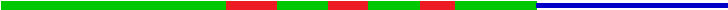 |
| 2 | Pp1s359_32V6 | 854 | 5.62 | 95.4 | 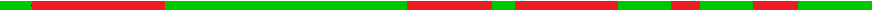 |
| 2 | Pp1s213_67V6 | 763 | 5.53 | 85.1 | 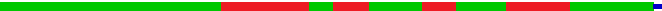 |
| 2 | Pp1s359_34V6 | 764 | 5.92 | 85.6 | 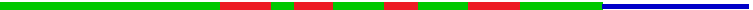 |
| 2 | Pp1s181_36V6 | 777 | 5.77 | 86.8 | 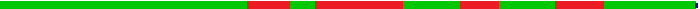 |
| 3 | Pp1s1_711V6 | 457 | 5.82 | 52.0 | 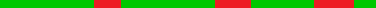 |
| 3 | Pp1s97_39V6 | 555 | 5.13 | 62.8 | 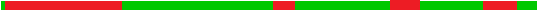 |
| 3 | Pp1s36_131V6 | 682 | 5.90 | 77.1 | 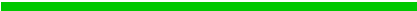 |
| 3 | Pp1s144_114V6 | 407 | 5.44 | 45.7 | 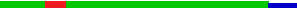 |
| 3 | Pp1s84_112V6 | 725 | 5.00 | 81.3 | 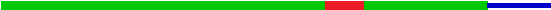 |
| 3 | Pp1s197_153V6 | 882 | 6.17 | 98.2 | 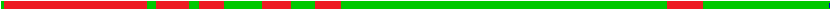 |
| 3 | Pp1s72_74V6 | 991 | 6.62 | 110.6 | 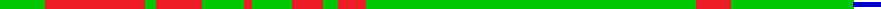 |
| 3 | Pp1s117_143V6 | 647 | 5.85 | 72.0 | 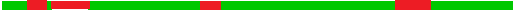 |
| 4 | Pp1s12_244V6 | 712 | 5.70 | 78.7 | 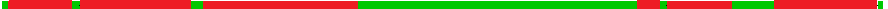 |
| 4 | Pp1s175_16V6 | 595 | 6.18 | 65.8 | 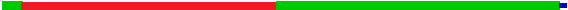 |
| 5a | Pp1s324_56V6 | 755 | 6.14 | 83.4 | 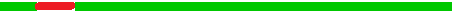 |
| 5a | Pp1s882_1V6 | 852 | 5.69 | 94.0 | 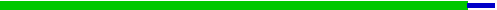 |
| 5a | Pp1s85_139V6 | 891 | 6.15 | 97.7 | 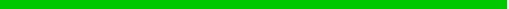 |
| 5a | Pp1s130_153V6 | 549 | 6.17 | 60.8 | 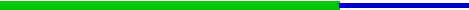 |
| 5a | Pp1s80_27V6 | 544 | 5.87 | 60.2 | 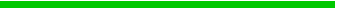 |
| 5b | Pp1s130_58V6 | 625 | 5.47 | 68.9 | 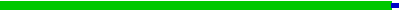 |
| 5b | Pp1s31_35V6 | 653 | 5.56 | 72.8 | 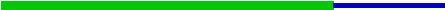 |
| 5b | Pp1s31_40V6 | 641 | 5.16 | 71.1 | 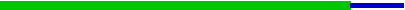 |
| 5b | Pp1s17_52V6 | 655 | 4.45 | 71.1 | 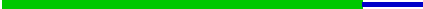 |
| 5b | Pp1s116_166V6 | 495 | 6.22 | 55.8 | 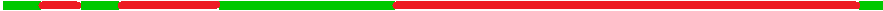 |
| 5b | Pp1s281_32V | 737 | 6.20 | 82.3 | 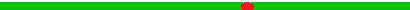 |
| 5b | Pp1s130_63V6 | 658 | 6.17 | 73.4 | 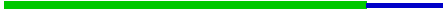 |
| 5b | Pp1s240_118V6 | 449 | 5.67 | 50.8 | 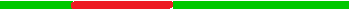 |
| 6 | Pp1s63_181V6 | 768 | 6.33 | 84.2 | 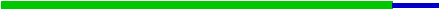 |
| 6 | Pp1s165_99V6 | 419 | 4.96 | 46.6 | 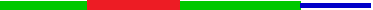 |
| 6 | Pp1s165_77V6 | 770 | 6.11 | 84.1 | 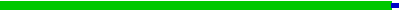 |
| 6 | Pp1s63_198V6 | 818 | 5.26 | 90.2 | 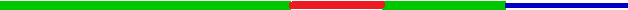 |
| 6 | Pp1s362_33V6 | 684 | 5.14 | 75.9 | 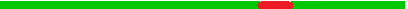 |
| 7 | Pp1s205_1V6 | 815 | 5.53 | 89.3 | 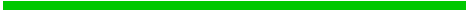 |

**Additional file 8. Predicted PpGRAS genes and related information**
