# Supplementary figures and images for: Unusual tandem expansion and positive selection in subgroups of the plant GRAS transcription factor superfamily
Source: BMC Plant Biol. 2014 Dec 19;14:373. doi: 10.1186/s12870-014-0373-5 (PMC4279901; doi:10.1186/s12870-014-0373-5)

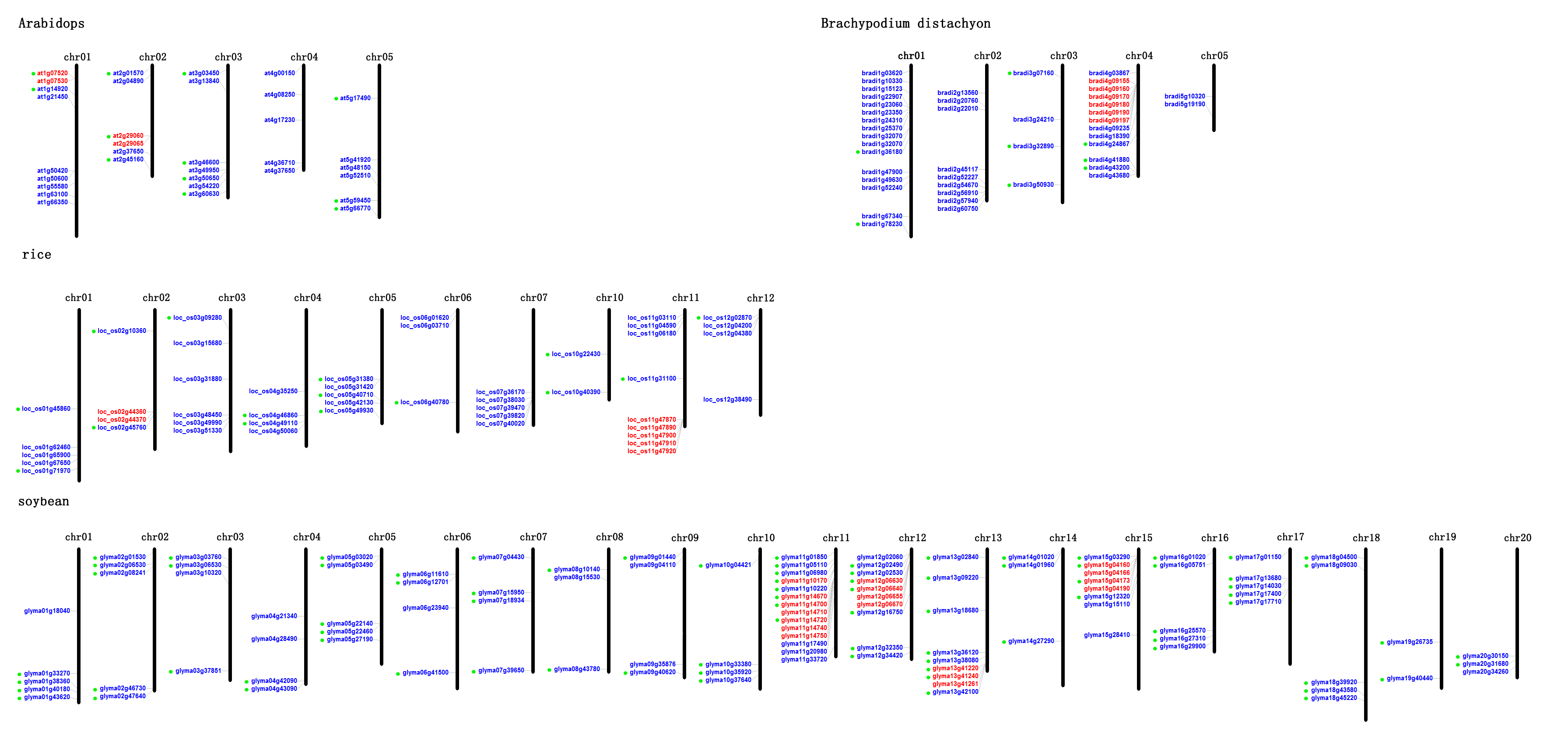

Supplement: Additional file 9: — Chromosome distribution of GRAS genes were from Arabidopsis , Brachypodium distachyon , rice, and soybean. The size of a chromosome is indicated by its relative length. Red genes represent tandemly duplicated genes, and green circle represent segmentally duplicated genes. The location information and chromosome information were obtained from Phytozome. The figure was produced using the MapInspector program. [file 12870_2014_373_MOESM9_ESM.png]

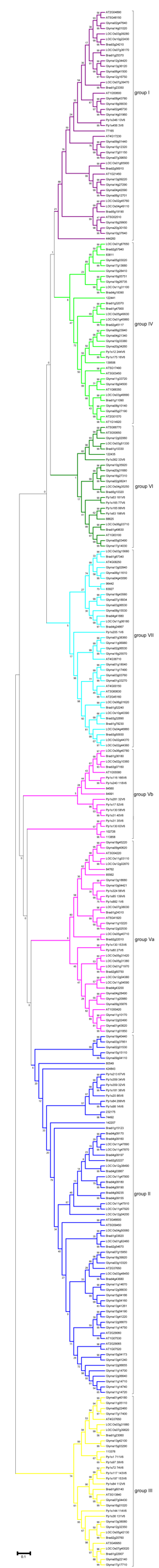

Supplement: Additional file 12: — The ME Phylogenetic tree of GRAS proteins among Arabidopsis , Brachypodium distachyon , rice, soybean, Selaginella moellendorffii , and Physcomitrella patens . The major clusters of orthologous genes are shown in different colors: group I = purple, group II = dark blue, group III = yellow, group IV = light green, group V = pink, group VI = dark green, and group VII = light blue. The scale bar corresponds to 0.1 estimated amino acid substitutions per site. [file 12870_2014_373_MOESM12_ESM.png]

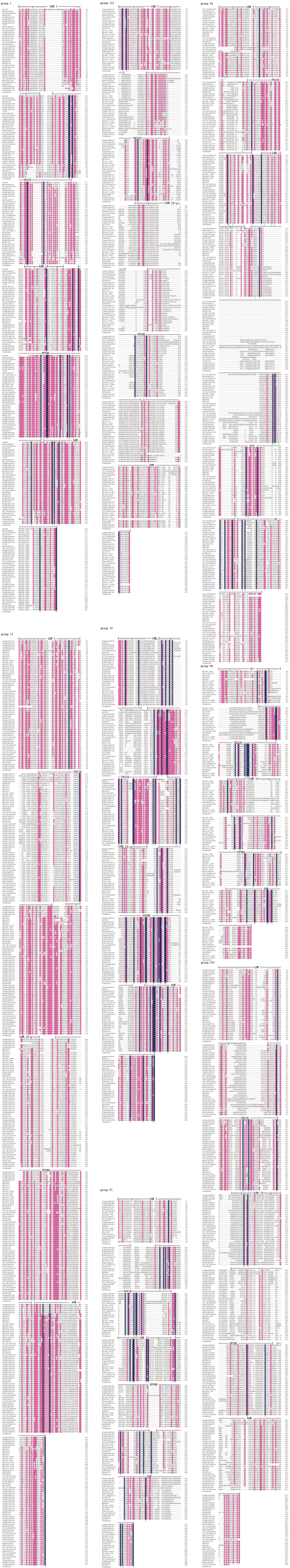

Supplement: Additional file 13: — Multiple sequence alignment of GRAS proteins in seven groups. Multiple sequence alignment (Corpet 1988) was applied to do complete alignment of conserved GRAS domain residues. GRAS proteins share five conserved motifs: LHRI, VHIID, LHRII, PFYRE, and SAW motif. Green arrow represent 16 critical amino acid residues responsible for positive selection and two types of functional divergence. [file 12870_2014_373_MOESM13_ESM.png]

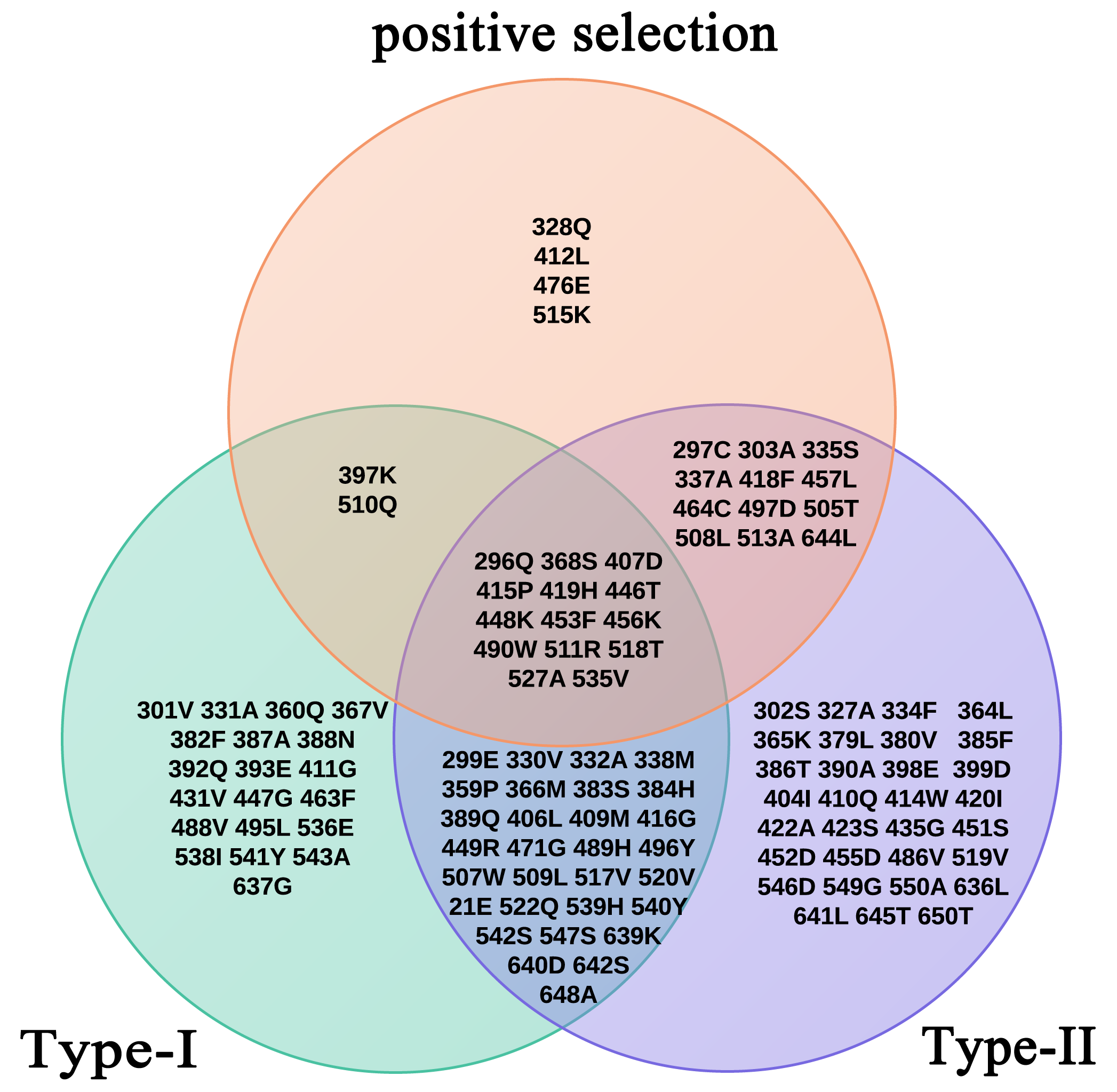

Supplement: Additional file 17: — The relationships between amino acid sites under positive selection and two types functional divergence. [file 12870_2014_373_MOESM17_ESM.png]

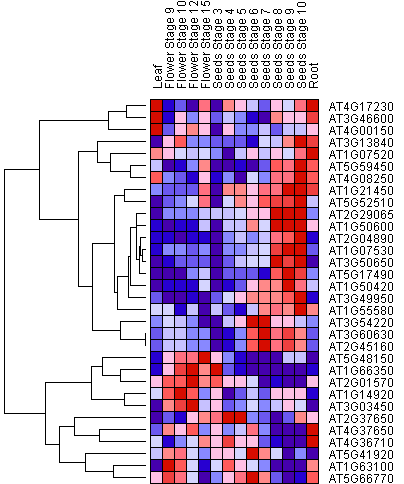

Supplement: Additional file 24: — Expression of the GRAS genes in various organs of Arabidopsis. Gene names are displayed to the right of each row. The color scheme used to represent expression level is red/blue: blue boxes indicate a low expression, red boxes indicate a high expression. [file 12870_2014_373_MOESM24_ESM.png]

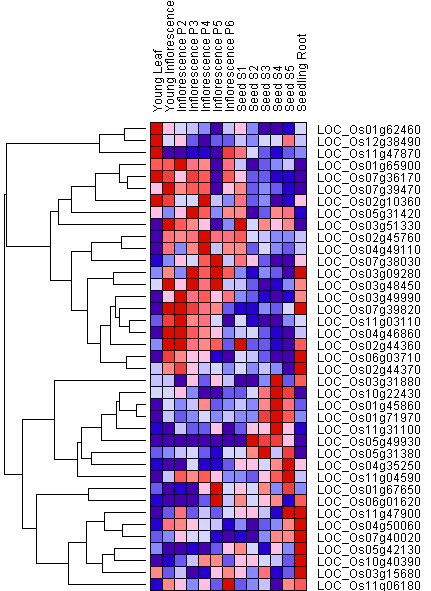

Supplement: Additional file 25: — Expression of the GRAS genes in various organs of rice. Gene names are displayed to the right of each row. The color scheme used to represent expression level is red/blue: blue boxes indicate a low expression, red boxes indicate a high expression. [file 12870_2014_373_MOESM25_ESM.png]

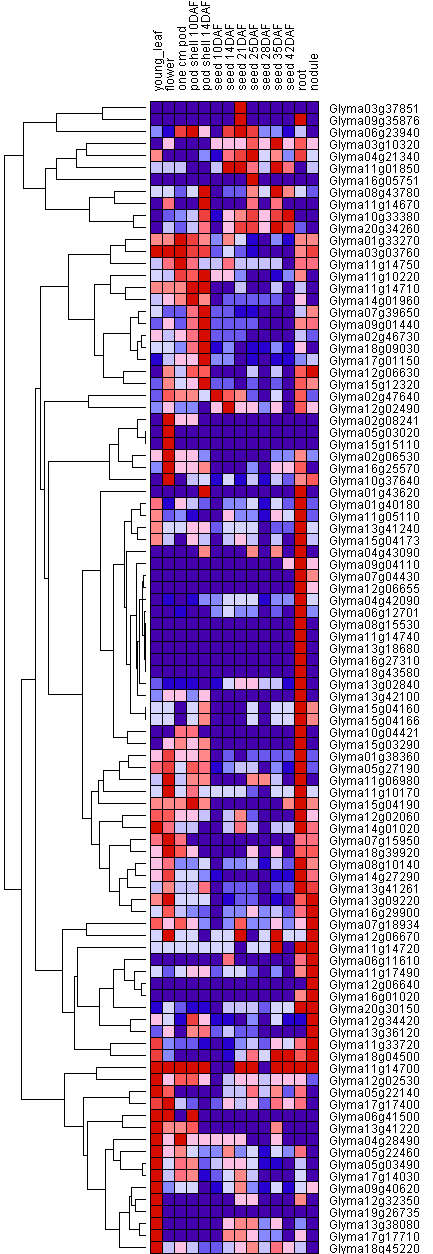

Supplement: Additional file 26: — Expression of the GRAS genes in various organs of soybean. Gene names are displayed to the right of each row. The color scheme used to represent expression level is red/blue: blue boxes indicate a low expression, red boxes indicate a high expression. [file 12870_2014_373_MOESM26_ESM.png]
