# Supplementary material for: Unusual tandem expansion and positive selection in subgroups of the plant GRAS transcription factor superfamily
Source: BMC Plant Biol. 2014 Dec 19;14:373. doi: 10.1186/s12870-014-0373-5 (PMC4279901; doi:10.1186/s12870-014-0373-5)
Supplement: Additional file 10: — The phylogenetic tree data of the GRAS gene subfamily in Arabidopsis , Brachypodium distachyon , rice, soybean, Selaginella moellendorffii , and Physcomitrella patens . [file 12870_2014_373_MOESM10_ESM.doc]

((((((((((((((((AT2G04890,AT5G48150)0.5680,(Glyma02g47640,Glyma14g01020)1.0000)0.9260,(LOC_Os03g09280,(LOC_Os10g22430,Bradi3g24210)0.6550)0.9990)0.3820,((LOC_Os07g36170,Bradi1g25370)1.0000,((Glyma12g34420,Glyma13g36120)1.0000,(Glyma06g41500,Glyma12g16750)0.9880)0.9970)0.9220)0.1910,(LOC_Os07g39470,Bradi1g23350)1.0000)0.3480,(AT1G50600,((Glyma08g43780,Glyma18g09030)0.9960,(Glyma02g46730,Glyma14g01960)1.0000)0.9680)0.8650)0.5400,(Pp1s346_13V6,Pp1s456_3V6)1.0000)0.3300,77165)0.2250,(AT4G17230,((Glyma09g01440,Glyma15g12320)1.0000,(Glyma17g01150,Glyma07g39650)1.0000)0.9990)0.7670)0.5100,((LOC_Os01g65900,Bradi2g56910)1.0000,(AT1G21450,((Glyma13g09220,Glyma14g27290)0.9990,(Glyma04g42090,Glyma06g12701)1.0000)0.9980)0.9090)0.9990)0.7440,(((LOC_Os02g45760,LOC_Os04g49110)0.5960,Bradi5g19190)1.0000,((AT5G52510,Glyma16g29900)0.4670,(Glyma20g30150,Glyma10g37640)1.0000)0.9770)1.0000)0.8990,444260)0.1980,((((((LOC_Os01g67650,Bradi2g57940)1.0000,83811)0.4940,(Glyma05g03020,Glyma17g13680)1.0000)0.9550,Glyma15g28410)0.9110,((Glyma16g05751,Glyma19g26735)1.0000,(LOC_Os11g31100,Bradi4g18390)1.0000)1.0000)0.2150,(122441,((((Bradi1g32070,Bradi1g47900)1.0000,LOC_Os05g49930)0.7200,(LOC_Os01g45860,Bradi2g45117)1.0000)0.7270,((((((Glyma06g23940,Glyma04g21340)0.9360,Glyma10g33380)0.9670,Glyma20g34260)1.0000,(Pp1s12_244V6,Pp1s175_16V6)0.9990)0.5260,139506)0.5470,((AT5G17490,AT3G03450)0.9750,(((Glyma11g33720,Glyma18g04500)1.0000,AT1G66350)0.2930,((LOC_Os03g49990,Bradi1g11090)1.0000,((Glyma08g10140,Glyma05g27190)0.9990,(AT2G01570,AT1G14920)0.9890)0.9890)0.4410)0.6650)0.9970)0.4910)0.7500)0.4470)0.0730)0.0120,((((((AT5G66770,AT3G50650)0.7010,Glyma12g02060)0.9360,(LOC_Os03g51330,Bradi1g10330)1.0000)0.9310,122435)0.9570,Pp1s362_33V6)0.8190,((((Glyma10g35920,Glyma20g31680)0.9690,(Glyma16g27310,Glyma02g08241)0.9970)1.0000,(LOC_Os04g35250,Bradi5g10320)1.0000)0.9960,((Pp1s63_181V6,Pp1s165_77V6)1.0000,(((Pp1s165_99V6,Pp1s63_198V6)1.0000,88625)0.6410,((LOC_Os06g03710,Bradi1g49630)1.0000,(AT1G63100,(Glyma05g03490,Glyma17g14030)1.0000)0.9970)0.9830)0.9880)0.4190)0.1860)0.0150)0.0000,((((((LOC_Os03g15680,Bradi1g67340)1.0000,AT4G08250)0.3590,(Glyma13g02840,(Glyma06g11610,Glyma04g43090)0.9910)0.5780)0.9990,(96442,83927)0.7310)0.7470,(((Glyma18g43580,Glyma07g18934)1.0000,Glyma03g06530)1.0000,(Glyma08g15530,(Bradi4g41880,(LOC_Os11g06180,Bradi4g24867)0.9780)1.0000)0.8030)0.3550)0.3030,(Pp1s205_1V6,((((Glyma01g38360,Glyma11g06980)0.9980,(Glyma02g06530,Glyma16g25570)1.0000)0.9930,AT4G36710)1.0000,((((Glyma01g18040,Glyma11g17490)1.0000,(Glyma03g03760,Glyma01g33270)1.0000)0.9940,(AT4G00150,(AT3G60630,AT2G45160)0.9960)0.4460)0.7620,((LOC_Os06g01620,Bradi1g52240)1.0000,(((LOC_Os10g40390,Bradi3g32890)0.6380,Bradi1g78230)0.9990,(LOC_Os04g46860,(Bradi3g50930,(LOC_Os02g44370,LOC_Os02g44360)0.9710)0.6630)1.0000)0.8040)0.9300)0.9820)0.9480)0.1950)0.0540)0.0000,(((((((Glyma01g40180,Glyma11g05110)1.0000,(Glyma05g22460,Glyma17g17400)0.9620)0.9510,AT4G37650)0.9500,(LOC_Os03g31880,(LOC_Os07g39820,Bradi1g23060)0.6160)0.9990)0.5260,(Glyma13g42100,Glyma15g03290)1.0000)0.9520,(113376,(Pp1s1_711V6,Pp1s97_39V6)0.7160)0.6800)0.9990,(((((Pp1s72_74V6,Pp1s117_143V6)0.4960,Pp1s197_153V6)1.0000,Pp1s84_112V6)1.0000,(Bradi1g60140,(AT3G13840,(Glyma07g04430,Glyma16g01020)1.0000)0.9680)1.0000)0.8900,(Pp1s144_114V6,(Pp1s36_131V6,(((Glyma13g38080,Glyma12g32350)1.0000,(LOC_Os05g42130,Bradi2g20760)1.0000)0.4940,(AT3G49950,((LOC_Os07g40020,Bradi1g22907)0.9800,(Glyma05g22140,Glyma17g17710)1.0000)0.8880)0.9670)0.9890)0.6820)0.9410)0.6020)0.7130,((((((((LOC_Os06g40780,Bradi1g36180)0.9710,(LOC_Os02g10360,Bradi3g07160)0.9280)1.0000,AT1G55580)0.6640,(Pp1s116_166V6,Pp1s240_118V6)1.0000)0.4290,84560)0.4190,84991)0.2430,(Pp1s281_32V6,(Pp1s17_52V6,(Pp1s130_58V6,Pp1s31_40V6)1.0000)0.9510)0.9670)0.0520,(((Pp1s31_35V6,Pp1s130_63V6)1.0000,(102726,113858)0.9770)0.9600,((((((((((Glyma18g45220,Glyma09g40620)1.0000,AT3G54220)0.9540,(LOC_Os11g03110,LOC_Os12g02870)1.0000)0.9800,84762)0.6170,85562)0.6310,((Glyma13g18680,Glyma10g04421)1.0000,(Pp1s324_56V6,(Pp1s85_139V6,Pp1s882_1V6)0.7460)0.9980)0.2940)0.3740,((LOC_Os07g38030,Bradi1g24310)0.9690,(AT5G41920,(Glyma11g10220,Glyma12g02530)0.9910)0.9020)1.0000)0.5910,(LOC_Os05g40710,Bradi2g22010)1.0000)0.7790,((Pp1s130_153V6,Pp1s80_27V6)1.0000,(((LOC_Os05g31420,LOC_Os05g31380)0.5810,(LOC_Os01g71970,Bradi2g60750)1.0000)0.5820,(((LOC_Os12g04380,LOC_Os11g04590)1.0000,Bradi4g43200)0.5920,(((Glyma04g28490,Glyma11g20980)1.0000,Glyma09g35876)0.5590,(AT1G50420,((Glyma11g10170,Glyma12g02490)1.0000,(Glyma01g43620,Glyma11g01850)1.0000)0.8280)0.8840)0.8440)0.3130)0.5110)0.4620)0.0420,((((Glyma19g40440,Glyma03g37851)0.9980,Glyma02g01530)1.0000,(Glyma15g15110,Glyma09g04110)0.9990)1.0000,(80549,(424843,((((((Pp1s213_67V6,Pp1s359_34V6)1.0000,(Pp1s359_32V6,Pp1s181_36V6)0.9910)0.9040,Pp1s20_86V6)0.9780,(Pp1s84_299V6,Pp1s98_14V6)1.0000)0.9620,(232175,74492)0.9740)0.5450,(142207,(Bradi1g15123,((((((((((Bradi4g09170,Bradi4g09160)1.0000,(LOC_Os11g47890,LOC_Os11g47870)0.9970)0.2620,Bradi4g09197)0.1740,Bradi2g52227)0.2390,(LOC_Os12g38490,Bradi4g03867)1.0000)0.0930,(LOC_Os11g47900,(Bradi4g09180,Bradi4g09190)0.9920)0.7750)0.1460,(Bradi4g09235,Bradi4g09155)1.0000)0.1640,(LOC_Os11g47910,LOC_Os11g47920)1.0000)0.4710,LOC_Os12g04200)0.6990,((AT3G46600,AT5G59450)1.0000,(((LOC_Os04g50060,Bradi1g03620)0.9950,(LOC_Os01g62460,Bradi2g54670)1.0000)0.3470,((((((Glyma07g15950,Glyma18g39920)1.0000,Glyma03g10320)0.9840,AT2G37650)0.8840,(LOC_Os03g48450,Bradi4g43680)1.0000)0.2940,((Glyma11g14670,Glyma12g06630)1.0000,(Glyma15g04166,(Glyma15g04160,Glyma13g41261)0.8210)1.0000)0.9930)0.3300,((((Glyma15g04190,Glyma13g41220)1.0000,(Glyma12g06670,Glyma11g14750)1.0000)0.4030,(AT2G29060,AT1G07530)0.9970)0.5970,((AT2G29065,AT1G07520)0.9830,((Glyma15g04173,Glyma13g41240)1.0000,(Glyma12g06655,(Glyma11g14700,(Glyma12g06640,(Glyma11g14710,(Glyma11g14740,Glyma11g14720)0.6600)0.5350)0.6250)0.5890)0.9850)0.7380)0.3270)0.7070)0.1110)0.2890)0.4010)0.3290)0.7770)0.2050)0.3340)0.1130)0.1930)0.0520)0.0020)0.0020)0.0000);
