# Supplementary material for: Unusual tandem expansion and positive selection in subgroups of the plant GRAS transcription factor superfamily
Source: BMC Plant Biol. 2014 Dec 19;14:373. doi: 10.1186/s12870-014-0373-5 (PMC4279901; doi:10.1186/s12870-014-0373-5)
Supplement: Additional file 15: — Schematic distribution of conserved motifs identified by means of MEME software among defined gene clusters. Position of each identified motif in all GRAS proteins represented in parenthesis. The highlighted with blue and red represents the conserved GRAS and DELLA domain, respectively. [file 12870_2014_373_MOESM15_ESM.doc]

**Additional file 15. Schematic distribution of conserved motifs identified by means of MEME software among defined gene clusters.**

| group I | Glyma20g30150 | motif4(167-187) | **GRAS(237-594)** |  |  |  |
| --- | --- | --- | --- | --- | --- | --- |
| group I | Glyma10g37640 | motif4(167-187) | **GRAS(238-596)** |  |  |  |
| group I | AT5G52510 | motif4(191-211) | **GRAS(265-640)** |  |  |  |
| group I | Glyma16g29900 | motif4(210-230) | **GRAS(288-657)** |  |  |  |
| group I | LOC_Os02g45760 | motif4(161-181) | **GRAS(253-618)** |  |  |  |
| group I | Bradi5g19190 | motif4(166-186) | **GRAS(263-631)** |  |  |  |
| group I | LOC_Os04g49110 | motif4(169-189) | **GRAS(261-619)** |  |  |  |
| group I | Bradi2g56910 | motif4(135-155) | **GRAS(201-571)** |  |  |  |
| group I | LOC_Os01g65900 | motif4(117-137) | **GRAS(183-553)** |  |  |  |
| group I | AT1G21450 | motif3(58-78) | motif4(153-173) | **GRAS(222-593)** |  |  |
| group I | Glyma13g09220 | motif4(139-159) | **GRAS(220-591)** |  |  |  |
| group I | Glyma14g27290 | motif4(139-159) | **GRAS(220-591)** |  |  |  |
| group I | Glyma04g42090 | motif4(139-159) | **GRAS(222-593)** |  |  |  |
| group I | Glyma06g12701 | motif4(139-159) | **GRAS(226-597)** |  |  |  |
| group I | Bradi1g23350 | motif4(130-150) | **GRAS(179-549)** |  |  |  |
| group I | LOC_Os07g39470 | motif4(125-145) | **GRAS(174-544)** |  |  |  |
| group I | Glyma12g34420 | motif4(116-136) | **GRAS(200-570)** |  |  |  |
| group I | Glyma13g36120 | motif4(115-135) | **GRAS(206-576)** |  |  |  |
| group I | Glyma06g41500 | motif4(122-142) | **GRAS(198-568)** |  |  |  |
| group I | Glyma12g16750 | motif4(122-142) | **GRAS(198-568)** |  |  |  |
| group I | Bradi1g25370 | motif4(112-132) | **GRAS(201-571)** |  |  |  |
| group I | LOC_Os07g36170 | motif6(82-96) | motif4(112-132) | **GRAS(201-571)** |  |  |
| group I | Glyma02g46730 | motif4(122-142) | **GRAS(175-545)** |  |  |  |
| group I | Glyma14g01960 | motif4(122-142) | **GRAS(175-545)** |  |  |  |
| group I | Glyma08g43780 | motif4(123-143) | **GRAS(175-545)** |  |  |  |
| group I | Glyma18g09030 | motif4(123-143) | **GRAS(175-545)** |  |  |  |
| group I | AT1G50600 | motif4(177-197) | **GRAS(227-597)** |  |  |  |
| group I | Glyma17g01150 | motif4(118-138) | **GRAS(174-545)** |  |  |  |
| group I | Glyma07g39650 | motif4(118-138) | **GRAS(171-542)** |  |  |  |
| group I | Glyma09g01440 | motif4(120-140) | **GRAS(175-546)** |  |  |  |
| group I | Glyma15g12320 | motif4(125-145) | **GRAS(179-550)** |  |  |  |
| group I | AT4G17230 | motif4(108-128) | **GRAS(155-525)** |  |  |  |
| group I | Bradi3g24210 | motif4(121-141) | **GRAS(171-541)** |  |  |  |
| group I | LOC_Os10g22430 | motif4(121-141) | **GRAS(171-541)** |  |  |  |
| group I | LOC_Os03g09280 | motif4(118-138) | **GRAS(166-535)** |  |  |  |
| group I | AT2G04890 | **GRAS(50-413)** |  |  |  |  |
| group I | AT5G48150 | motif4(68-88) | **GRAS(122-493)** |  |  |  |
| group I | Glyma02g47640 | motif4(118-138) | **GRAS(171-541)** |  |  |  |
| group I | Glyma14g01020 | motif4(122-142) | **GRAS(175-545)** |  |  |  |
| group II | Glyma15g04160 | motif1(66-80) | motif3(85-105) | motif6(243-257) | **GRAS(316-682)** |  |
| group II | Glyma13g41261 | motif1(65-79) | motif3(84-104) | motif6(214-228) | **GRAS(287-655)** |  |
| group II | Glyma15g04166 | motif1(10-24) | motif3(29-49) | motif6(163-177) | **GRAS(236-604)** |  |
| group II | Glyma11g14670 | motif1(67-81) | motif3(86-106) | motif6(234-248) | **GRAS(310-678)** |  |
| group II | Glyma12g06630 | motif1(74-88) | motif3(93-113) | motif6(240-254) | **GRAS(317-685)** |  |
| group II | AT2G37650 | motif1(81-95) | motif3(98-118) | motif2(208-236) | motif6(257-271) | **GRAS(344-713)** |
| group II | Glyma03g10320 | motif1(78-92) | motif3(95-115) | motif2(208-236) | motif6(278-292) | **GRAS(357-728)** |
| group II | Glyma07g15950 | motif1(71-85) | motif3(88-108) | motif2(208-236) | motif6(283-297) | **GRAS(362-732)** |
| group II | Glyma18g39920 | motif1(70-84) | motif3(87-107) | motif2(207-235) | motif6(282-296) | **GRAS(367-737)** |
| group II | Bradi2g54670 | motif1(101-115) | motif2(272-300) | motif6(347-361) | **GRAS(429-799)** |  |
| group II | LOC_Os01g62460 | motif3 (12-32) | motif2(171-199) | motif6(250-264) | **GRAS(328-698)** |  |
| group II | Bradi4g03867 | motif1(79-93) | motif5(94-117) | motif2(208-236) | motif6(284-298) | **GRAS(358-730)** |
| group II | LOC_Os12g38490 | motif1(78-92) | motif5(93-116) | motif2(183-211) | motif6(282-296) | **GRAS(358-730)** |
| group II | Bradi1g03620 | motif1(91-105) | motif6(212-226) | **GRAS(286-657)** |  |  |
| group II | LOC_Os04g50060 | motif1(81-95) | motif6(189-203) | **GRAS(261-634)** |  |  |
| group II | Bradi4g43680 | motif1(130-144) | motif3(148-168) | motif2(270-298) | motif6(336-350) | **GRAS(409-780)** |
| group II | LOC_Os03g48450 | motif1(79-93) | motif3 (97-117) | motif2(216-244) | motif6(283-297) | **GRAS(356-727)** |
| group II | AT3G46600 | motif1(62-76) | motif3(79-99) | motif6(148-162) | **GRAS(209-579)** |  |
| group II | AT5G59450 | motif1(61-75) | motif3(76-96) | motif6(153-167) | **GRAS(224-598)** |  |
| group II | AT2G29065 | motif1(37-51) | motif3(55-75) | motif2(123-151) | motif6(182-196) | **GRAS(249-628)** |
| group II | AT1G07520 | motif1(69-83) | motif3(86-106) | motif2(187-215) | motif6(253-267) | **GRAS(315-693)** |
| group II | Glyma12g06670 | motif3(11-31) | motif2(137-165) | motif6(209-223) | **GRAS(287-658)** |  |
| group II | Glyma11g14750 | motif1(80-94) | motif3(97-117) | motif2(219-247) | motif6(291-305) | **GRAS(369-740)** |
| group II | Glyma15g04190 | motif1(26-40) | motif3(43-63) | motif2(152-180) | motif6(227-241) | **GRAS(289-663)** |
| group II | Glyma13g41220 | motif1(16-30) | motif3(33-53) | motif6(206-220) | **GRAS(270-642)** |  |
| group II | Glyma15g04173 | motif1(82-96) | motif3(99-119) | motif2(208-236) | motif6(272-286) | **GRAS(352-725)** |
| group II | Glyma13g41240 | motif1(103-117) | motif3(120-140) | motif2(229-257) | motif6(295-309) | **GRAS(368-741)** |
| group II | Glyma12g06655 | motif1(43-57) | motif3(58-78) | motif2(164-192) | motif6(230-244) | **GRAS(299-674)** |
| group II | Glyma11g14710 | motif1(74-88) | motif3(91-111) | motif2(185-213) | motif6(250-264) | **GRAS(319-694)** |
| group II | Glyma12g06640 | motif1(48-62) | motif3(63-83) | motif2(174-204) | motif6(239-253) | **GRAS(308-678)** |
| group II | Glyma11g14740 | motif1(43-57) | motif2(165-193) | **GRAS(211-569)** |  |  |
| group II | Glyma11g14700 | motif1(26-40) | motif3(41-61) | motif2(105-133) | motif6(169-183) | **GRAS(237-612)** |
| group II | Glyma11g14720 | motif1(34-48) | motif3(49-69) | motif2(162-190) | motif6(227-241) | **GRAS(296-671)** |
| group II | AT2G29060 | motif1(60-74) | motif3(77-97) | motif2 (194-222) | motif6(242-256) | **GRAS(318-692)** |
| group II | AT1G07530 | motif1(86-100) | motif3(103-123) | motif2(230-258) | motif6(315-329) | **GRAS(393-765)** |
| group II | LOC_Os12g04200 | motif1(18-32) | motif5(34-57) | motif6(102-116) | **GRAS(167-547)** |  |
| group II | LOC_Os11g47910 | motif1(50-64) | motif2(89-117) | motif6(146-160) | **GRAS(215-586)** |  |
| group II | LOC_Os11g47920 | motif1(48-62) | motif2(87-115) | motif6(144-158) | **GRAS(213-584)** |  |
| group II | Bradi4g09180 | motif1(45-59) | motif5(61-84) | motif2(133-161) | motif6(181-195) | **GRAS(253-626)** |
| group II | Bradi4g09190 | motif1(54-68) | motif5(70-93) | motif2(138-166) | motif6(185-199) | **GRAS(259-632)** |
| group II | LOC_Os11g47900 | motif1(27-41) | motif5(42-65) | motif2(117-145) | **GRAS(263-639)** |  |
| group II | Bradi4g09235 | motif1(41-55) | motif5(56-79) | motif2(136-164) | motif6(179-193) | **GRAS(251-624)** |
| group II | Bradi4g09155 | motif1(14-28) | motif5(29-52) | motif2(109-137) | motif6(152-166) | **GRAS(224-597)** |
| group II | Bradi4g09170 | motif1(38-52) | motif5(53-76) | motif2(139-167) | motif6(192-206) | **GRAS(262-634)** |
| group II | Bradi4g09160 | motif1(35-49) | motif5(50-73) | motif2(137-165) | motif6(190-204) | **GRAS(260-632)** |
| group II | Bradi4g09197 | motif1(62-76) | motif5(77-100) | motif2(222-250) | motif6(297-311) | **GRAS(379-758)** |
| group II | Bradi2g52227 | motif1(50-64) | motif5(65-88) | motif2(247-275) | **GRAS(387-760)** |  |
| group II | LOC_Os11g47890 | motif1(44-58) | motif5(59-82) | motif2(126-154) | motif6(190-204) | **GRAS(257-634)** |
| group II | LOC_Os11g47870 | motif1(40-54) | motif5(55-78) | motif2(161-189) | **GRAS(303-683)** |  |
| group II | Bradi1g15123 | motif6(137-151) | **GRAS(211-582)** |  |  |  |
| group II | Glyma15g15110 | **GRAS(219-591)** |  |  |  |  |
| group II | Glyma09g04110 | **GRAS(223-593)** |  |  |  |  |
| group II | Glyma19g40440 | **GRAS(194-563)** |  |  |  |  |
| group II | Glyma03g37851 | **GRAS(169-538)** |  |  |  |  |
| group II | Glyma02g01530 | **GRAS(136-493)** |  |  |  |  |
| group III | Bradi1g22907 | **GRAS(70-482)** |  |  |  |  |
| group III | LOC_Os07g40020 | **GRAS(76-471)** |  |  |  |  |
| group III | Glyma05g22140 | **GRAS(57-478)** |  |  |  |  |
| group III | Glyma17g17710 | **GRAS(57-480)** |  |  |  |  |
| group III | AT3G49950 | **GRAS(27-408)** |  |  |  |  |
| group III | Glyma13g38080 | **GRAS(54-450)** |  |  |  |  |
| group III | Glyma12g32350 | **GRAS(50-445)** |  |  |  |  |
| group III | Bradi2g20760 | **GRAS(37-418)** |  |  |  |  |
| group III | LOC_Os05g42130 | **GRAS(36-423)** |  |  |  |  |
| group III | Bradi1g60140 | **GRAS(140-518)** |  |  |  |  |
| group III | AT3G13840 | **GRAS(145-510)** |  |  |  |  |
| group III | Glyma07g04430 | **GRAS(157-533)** |  |  |  |  |
| group III | Glyma16g01020 | **GRAS(157-531)** |  |  |  |  |
| group III | Glyma13g42100 | **GRAS(66-442)** |  |  |  |  |
| group III | Glyma15g03290 | **GRAS(68-442)** |  |  |  |  |
| group III | Bradi1g23060 | **GRAS(183-592)** |  |  |  |  |
| group III | LOC_Os07g39820 | **GRAS(192-601)** |  |  |  |  |
| group III | LOC_Os03g31880 | **GRAS(188-602)** |  |  |  |  |
| group III | AT4G37650 | **GRAS(143-529)** |  |  |  |  |
| group III | Glyma01g40180 | **GRAS(100-473)** |  |  |  |  |
| group III | Glyma11g05110 | **GRAS(105-479)** |  |  |  |  |
| group III | Glyma05g22460 | **GRAS(123-498)** |  |  |  |  |
| group III | Glyma17g17400 | **GRAS(126-502)** |  |  |  |  |
| group IV | Glyma15g28410 | **GRAS(176-545)** |  |  |  |  |
| group IV | Bradi2g57940 | **GRAS(156-530)** |  |  |  |  |
| group IV | LOC_Os01g67650 | **GRAS(157-531)** |  |  |  |  |
| group IV | Glyma05g03020 | **GRAS(138-510)** |  |  |  |  |
| group IV | Glyma17g13680 | **GRAS(141-513)** |  |  |  |  |
| group IV | Bradi4g18390 | **GRAS(374-738)** |  |  |  |  |
| group IV | LOC_Os11g31100 | **GRAS(401-771)** |  |  |  |  |
| group IV | Glyma16g05751 | **GRAS(325-686)** |  |  |  |  |
| group IV | Glyma19g26735 | **GRAS(319-680)** |  |  |  |  |
| group IV | Bradi1g32070 | **GRAS(11-390)** |  |  |  |  |
| group IV | Bradi1g47900 | **GRAS(11-390)** |  |  |  |  |
| group IV | LOC_Os05g49930 | **GRAS(77-454)** |  |  |  |  |
| group IV | Bradi2g45117 | **GRAS(88-461)** |  |  |  |  |
| group IV | LOC_Os01g45860 | **GRAS(86-449)** |  |  |  |  |
| group IV | Glyma10g33380 | **DELLA(24-96)** | **GRAS(122-480)** |  |  |  |
| group IV | Glyma20g34260 | **GRAS(63-420)** |  |  |  |  |
| group IV | Glyma06g23940 | motif6(104-118) | **DELLA(25-100)** | **GRAS(130-497)** |  |  |
| group IV | Glyma04g21340 | **DELLA(19-94)** | **GRAS(124-489)** |  |  |  |
| group IV | AT1G66350 | **DELLA(35-104)** | **GRAS(155-509)** |  |  |  |
| group IV | AT5G17490 | **DELLA(34-106)** | **GRAS(157-516)** |  |  |  |
| group IV | AT3G03450 | **DELLA(44-115)** | **GRAS(180-545)** |  |  |  |
| group IV | Glyma11g33720 | **DELLA(55-128)** | **GRAS(218-583)** |  |  |  |
| group IV | Glyma18g04500 | **DELLA(44-117)** | **GRAS(209-573)** |  |  |  |
| group IV | Bradi1g11090 | **DELLA(36-117)** | **GRAS(240-619)** |  |  |  |
| group IV | LOC_Os03g49990 | **DELLA(39-120)** | **GRAS(241-621)** |  |  |  |
| group IV | AT2G01570 | **DELLA(44-117)** | **GRAS(221-581)** |  |  |  |
| group IV | AT1G14920 | **DELLA(28-101)** | **GRAS(169-529)** |  |  |  |
| group IV | Glyma08g10140 | **DELLA(34-106)** | **GRAS(157-512)** |  |  |  |
| group IV | Glyma05g27190 | **DELLA(34-106)** | **GRAS(158-513)** |  |  |  |
| group Va | Bradi2g22010 | **GRAS(84-455)** |  |  |  |  |
| group Va | LOC_Os05g40710 | **GRAS(116-484)** |  |  |  |  |
| group Va | Glyma18g45220 | **GRAS(297-653)** |  |  |  |  |
| group Va | Glyma09g40620 | **GRAS(456-812)** |  |  |  |  |
| group Va | AT3G54220 | **GRAS(290-650)** |  |  |  |  |
| group Va | LOC_Os11g03110 | **GRAS(283-644)** |  |  |  |  |
| group Va | LOC_Os12g02870 | **GRAS(292-653)** |  |  |  |  |
| group Va | Glyma13g18680 | **GRAS(164-521)** |  |  |  |  |
| group Va | Glyma10g04421 | **GRAS(174-532)** |  |  |  |  |
| group Va | AT5G41920 | **GRAS(40-400)** |  |  |  |  |
| group Va | Bradi1g24310 | **GRAS(75-441)** |  |  |  |  |
| group Va | LOC_Os07g38030 | **GRAS(68-434)** |  |  |  |  |
| group Va | Glyma11g10220 | **GRAS(71-432)** |  |  |  |  |
| group Va | Glyma12g02530 | **GRAS(71-432)** |  |  |  |  |
| group Va | Glyma01g43620 | **GRAS(44-462)** |  |  |  |  |
| group Va | Glyma11g01850 | **GRAS(49-470)** |  |  |  |  |
| group Va | Glyma11g10170 | **GRAS(28-452)** |  |  |  |  |
| group Va | Glyma12g02490 | **GRAS(28-452)** |  |  |  |  |
| group Va | AT1G50420 | **GRAS(57-482)** |  |  |  |  |
| group Va | Glyma09g35876 | **GRAS(11-420)** |  |  |  |  |
| group Va | Glyma04g28490 | **GRAS(23-440)** |  |  |  |  |
| group Va | Glyma11g20980 | **GRAS(23-439)** |  |  |  |  |
| group Va | Bradi4g43200 | **GRAS(52-522)** |  |  |  |  |
| group Va | LOC_Os05g31380 | **GRAS(92-506)** |  |  |  |  |
| group Va | Bradi2g60750 | **GRAS(58-460)** |  |  |  |  |
| group Va | LOC_Os01g71970 | **GRAS(50-437)** |  |  |  |  |
| group Va | LOC_Os05g31420 | **GRAS(154-558)** |  |  |  |  |
| group Va | LOC_Os12g04380 | **GRAS(1-438)** |  |  |  |  |
| group Va | LOC_Os11g04590 | **GRAS(1-434)** |  |  |  |  |
| group Vb | Bradi1g36180 | **GRAS(86-494)** |  |  |  |  |
| group Vb | LOC_Os06g40780 | **GRAS(275-662)** |  |  |  |  |
| group Vb | Bradi3g07160 | **GRAS(27-409)** |  |  |  |  |
| group Vb | LOC_Os02g10360 | **GRAS(39-420)** |  |  |  |  |
| group Vb | AT1G55580 | **GRAS(41-445)** |  |  |  |  |
| group VI | Bradi1g10330 | **GRAS(170-541)** |  |  |  |  |
| group VI | LOC_Os03g51330 | **GRAS(207-578)** |  |  |  |  |
| group VI | Glyma12g02060 | **GRAS(118-481)** |  |  |  |  |
| group VI | AT5G66770 | **GRAS(217-584)** |  |  |  |  |
| group VI | AT3G50650 | **GRAS(180-542)** |  |  |  |  |
| group VI | Glyma05g03490 | **GRAS(275-648)** |  |  |  |  |
| group VI | Glyma17g14030 | **GRAS(280-653)** |  |  |  |  |
| group VI | AT1G63100 | **GRAS(274-654)** |  |  |  |  |
| group VI | Bradi1g49630 | **GRAS(235-609)** |  |  |  |  |
| group VI | LOC_Os06g03710 | **GRAS(217-594)** |  |  |  |  |
| group VI | Bradi5g10320 | **GRAS(73-456)** |  |  |  |  |
| group VI | LOC_Os04g35250 | **GRAS(86-495)** |  |  |  |  |
| group VI | Glyma10g35920 | **GRAS(84-463)** |  |  |  |  |
| group VI | Glyma20g31680 | **GRAS(81-460)** |  |  |  |  |
| group VI | Glyma16g27310 | **GRAS(87-469)** |  |  |  |  |
| group VI | Glyma02g08241 | **GRAS(88-474)** |  |  |  |  |
| group VII | Glyma06g11610 | **GRAS(101-489)** |  |  |  |  |
| group VII | Glyma04g43090 | **GRAS(106-497)** |  |  |  |  |
| group VII | Glyma13g02840 | **GRAS(96-469)** |  |  |  |  |
| group VII | AT4G08250 | **GRAS(104-477)** |  |  |  |  |
| group VII | Bradi1g67340 | **GRAS(125-527)** |  |  |  |  |
| group VII | LOC_Os03g15680 | **GRAS(132-526)** |  |  |  |  |
| group VII | Bradi4g24867 | **GRAS(102-466)** |  |  |  |  |
| group VII | LOC_Os11g06180 | **GRAS(47-399)** |  |  |  |  |
| group VII | Bradi4g41880 | **GRAS(101-478)** |  |  |  |  |
| group VII | Glyma08g15530 | **GRAS(117-477)** |  |  |  |  |
| group VII | Glyma03g06530 | **GRAS(148-518)** |  |  |  |  |
| group VII | Glyma18g43580 | **GRAS(176-542)** |  |  |  |  |
| group VII | Glyma07g18934 | **GRAS(190-557)** |  |  |  |  |
| group VII | Glyma02g06530 | **GRAS(154-522)** |  |  |  |  |
| group VII | Glyma16g25570 | **GRAS(173-539)** |  |  |  |  |
| group VII | Glyma01g38360 | **GRAS(167-524)** |  |  |  |  |
| group VII | Glyma11g06980 | **GRAS(158-515)** |  |  |  |  |
| group VII | AT4G36710 | **GRAS(122-485)** |  |  |  |  |
| group VII | Glyma03g03760 | **GRAS(345-702)** |  |  |  |  |
| group VII | Glyma01g33270 | **GRAS(379-736)** |  |  |  |  |
| group VII | Glyma01g18040 | **GRAS(387-743)** |  |  |  |  |
| group VII | Glyma11g17490 | **GRAS(388-744)** |  |  |  |  |
| group VII | AT4G00150 | **GRAS(205-554)** |  |  |  |  |
| group VII | AT3G60630 | **GRAS(243-622)** |  |  |  |  |
| group VII | AT2G45160 | **GRAS(271-642)** |  |  |  |  |
| group VII | Bradi1g52240 | **GRAS(170-536)** |  |  |  |  |
| group VII | LOC_Os06g01620 | **GRAS(126-479)** |  |  |  |  |
| group VII | Bradi3g32890 | **GRAS(275-652)** |  |  |  |  |
| group VII | LOC_Os10g40390 | **GRAS(35-384)** |  |  |  |  |
| group VII | Bradi1g78230 | **GRAS(253-619)** |  |  |  |  |
| group VII | LOC_Os04g46860 | **GRAS(352-710)** |  |  |  |  |
| group VII | Bradi3g50930 | **GRAS(363-723)** |  |  |  |  |
| group VII | LOC_Os02g44370 | **GRAS(355-714)** |  |  |  |  |
| group VII | LOC_Os02g44360 | **GRAS(349-708)** |  |  |  |  |
