# Supplementary material for: Unusual tandem expansion and positive selection in subgroups of the plant GRAS transcription factor superfamily
Source: BMC Plant Biol. 2014 Dec 19;14:373. doi: 10.1186/s12870-014-0373-5 (PMC4279901; doi:10.1186/s12870-014-0373-5)
Supplement: Additional file 16: — Multilevel consensus sequences for the MEME defined motifs observed among different GRAS proteins from Arabidopsis, Brachypodium distachyon, rice, and soybean. [file 12870_2014_373_MOESM16_ESM.doc]

**Additional file 16. Multilevel consensus sequences for the MEME defined motifs observed among different GRAS proteins from *Arabidopsis*, *Brachypodium distachyon*, rice, and soybean.**

| Motif | No. Of  amino acids | E value | Multilevel consensus sequence |
| --- | --- | --- | --- |
| motif1 | 49 | 2.3e-322 | EVL[KP][YF]IS[QR][IM]LME[ED][DE]I[DE]D |
| motif2 | 38 | 9.4e-407 | [DE]D[MDE][SL][VN][LMS][QA]F[LR][KR]G[MVL]EEA[SN]KFLPx[GN]N[KN]L[VF][IT][DG]L |
| motif3 | 37 | 1.1e-257 | CM[FL][QY]DSLALQA[AT]EKSFY[DE][AV][LI][GT] |
| motif4 | 37 | 4.1e-204 | E[LM][RK]HK[LI][RQ]ELE[KT][AQ][LM]LG[PD][DE]D[DE]IL |
| motif5 | 13 | 6.3e-162 | [KHR]FFYQ[YF]PDHPALL[QR]AQQP[FY]AQILS[DA]P[SA]S |
| motif6 | 54 | 3.4e-153 | EEGRS[SN]K[QL]SAVxx[ED][DE] |
