# Supplementary material for: Unusual tandem expansion and positive selection in subgroups of the plant GRAS transcription factor superfamily
Source: BMC Plant Biol. 2014 Dec 19;14:373. doi: 10.1186/s12870-014-0373-5 (PMC4279901; doi:10.1186/s12870-014-0373-5)
Supplement: Additional file 18: — Parameters estimation and likelihood ratio tests for the site-specific model in Arabidopsis. Note: *p < 0.05 and **p < 0.01 (x 2 test). a ω was estimated under model M0,M3,M7, and M8; p and q are the parameters of the beta distribution. b The number of amino acid sites estimated to have undergone positive selection. [file 12870_2014_373_MOESM18_ESM.doc]

**Additional file 18. Parameters estimation and likelihood ratio tests for the site-specific model in *Arabidopsis*.**

| Model | lnL | Estimates of parameter ^a^ | 2ΔlnL | positive selection sites ^b^ |
| --- | --- | --- | --- | --- |
| M0(one-ratio) | -26102.43 | ω=0.11793 | 932.218  (M3vsM0)** | Not allowed |
| M3(discrete) | -25636.32 | p_0_=0.15370 ω_0_=0.01982 |  | None |
|  |  | p_1_=0.48247 ω_1_=0.09265 |  |  |
|  |  | p_2_=0.36383 ω_2_=0.22128 |  |  |
| M7(beta) | -25632.8 | p=1.52457 q=10.39253 | 0.006 (M8vsM7) | Not allowed |
| M8(beta & ω) | -25632.8 | p_0_=0.99999 p=1.52457 |  | None |
|  |  | q=10.39253 p_1_=0.00001 |  |  |
|  |  | ω=9.29435 |  |  |
