# Supplementary material for: Unusual tandem expansion and positive selection in subgroups of the plant GRAS transcription factor superfamily
Source: BMC Plant Biol. 2014 Dec 19;14:373. doi: 10.1186/s12870-014-0373-5 (PMC4279901; doi:10.1186/s12870-014-0373-5)
Supplement: Additional file 19: — Parameters estimation and likelihood ratio tests for the site-specific model in Brachypodium distachyon . Note: *p < 0.05 and **p < 0.01 (x 2 test). a ω was estimated under model M0,M3,M7, and M8; p and q are the parameters of the beta distribution. b The number of amino acid sites estimated to have undergone positive selection. [file 12870_2014_373_MOESM19_ESM.doc]

| Model | lnL | Estimates of parameter ^a^ | 2ΔlnL | positive selection sites ^b^ |
| --- | --- | --- | --- | --- |
| M0(one-ratio) | -34188.02 | ω=0.15462 | 1308.076 (M3vsM0)** | Not allowed |
| M3(discrete) | -33533.98 | p_0_=0.18514 ω_0_=0.04023 |  | None |
|  |  | p_1_=0.43590 ω_1_=0.12221 |  |  |
|  |  | p_2_=0.37896 ω_2_=0.30378 |  |  |
| M7(beta) | -33490.26 | p=1.32480 q=5.64051 | 43.21 (M8vsM7) | Not allowed |
| M8(beta & ω) | -33468.65 | p_0_=0.96722 p=1.66596 |  | none |
|  |  | q= 8.58279 p_1_=0.03278 |  |  |
|  |  | ω=1.04464 |  |  |

**Additional file 19. Parameters estimation and likelihood ratio tests for the site-specific model in *Brachypodium distachyon*.**
