# Supplementary material for: Unusual tandem expansion and positive selection in subgroups of the plant GRAS transcription factor superfamily
Source: BMC Plant Biol. 2014 Dec 19;14:373. doi: 10.1186/s12870-014-0373-5 (PMC4279901; doi:10.1186/s12870-014-0373-5)
Supplement: Additional file 20: — Parameters estimation and likelihood ratio tests for the site-specific model in rice. Note: *p < 0.05 and **p < 0.01 (x 2 test). a ω was estimated under model M0,M3,M7, and M8; p and q are the parameters of the beta distribution. b The number of amino acid sites estimated to have undergone positive selection. [file 12870_2014_373_MOESM20_ESM.doc]

**Additional file 20. Parameters estimation and likelihood ratio tests for the site-specific model in rice.**

| Model | lnL | Estimates of parameter ^a^ | 2ΔlnL | positive selection sites ^b^ |
| --- | --- | --- | --- | --- |
| M0(one-ratio) | -29767.17 | ω=0.11486 | 1046.63 (M3vsM0)** | Not allowed |
| M3(discrete) | -29243.85 | p_0_=0.21991 ω_0_=0.03238 |  | None |
|  |  | p_1_=0.48573 ω_1_=0.10688 |  |  |
|  |  | p_2_=0.29437 ω_2_=0.23457 |  |  |
| M7(beta) | -29232.56 | p=1.73421 q=11.92543 | 3274.794 (M8vsM7)** | Not allowed |
| M8(beta & ω) | -30869.95 | p_0_=0.99999 p=0.14923 |  | None |
|  |  | q= 1.12252 p_1_=0.00001 |  |  |
|  |  | ω=2.81878 |  |  |
