# Supplementary material for: Unusual tandem expansion and positive selection in subgroups of the plant GRAS transcription factor superfamily
Source: BMC Plant Biol. 2014 Dec 19;14:373. doi: 10.1186/s12870-014-0373-5 (PMC4279901; doi:10.1186/s12870-014-0373-5)
Supplement: Additional file 21: — Parameters estimation and likelihood ratio tests for the site-specific model in soybean. Note: *p < 0.05 and **p < 0.01 (x 2 test). a ω was estimated under model M0,M3,M7, and M8; p and q are the parameters of the beta distribution. b The number of amino acid sites estimated to have undergone positive selection. [file 12870_2014_373_MOESM21_ESM.doc]

| Model | lnL | Estimates of parameter ^a^ | 2ΔlnL | positive selection sites ^b^ |
| --- | --- | --- | --- | --- |
| M0(one-ratio) | -46071.14 | ω=0.16951 | 1425.198 (M3vsM0)  ** | Not allowed |
| M3(discrete) | -45358.54 | p_0_=0.20684 ω_0_=0.04574 |  | None |
|  |  | p_1_=0.44724 ω_1_=0.14196 |  |  |
|  |  | p_2_=0.34591 ω_2_=0.30300 |  |  |
| M7(beta) | -45325.98 | p=1.54997 q=7.29189 | 0.006 (M8vsM7) | Not allowed |
| M8(beta & ω) | -45325.98 | p_0_=0.99999 p=1.54995 |  | None |
|  |  | q=7.29176 p_1_=0.00001 |  |  |
|  |  | ω=3.84401 |  |  |

**Additional file 21. Parameters estimation and likelihood ratio tests for the site-specific model in soybean.**
