# Supplementary material for: Unusual tandem expansion and positive selection in subgroups of the plant GRAS transcription factor superfamily
Source: BMC Plant Biol. 2014 Dec 19;14:373. doi: 10.1186/s12870-014-0373-5 (PMC4279901; doi:10.1186/s12870-014-0373-5)
Supplement: Additional file 22: — Parameters estimation and likelihood ratio tests for the site-specific model in Selaginella moellendorffii . Note: *p < 0.05 and **p < 0.01 (x 2 test). a ω was estimated under model M0,M3,M7, and M8; p and q are the parameters of the beta distribution. b The number of amino acid sites estimated to have undergone positive selection. [file 12870_2014_373_MOESM22_ESM.doc]

**Additional file 22. Parameters estimation and likelihood ratio tests for the site-specific model in *Selaginella moellendorffii*.**

| Model | lnL | Estimates of parameter ^a^ | 2ΔlnL | positive selection sites ^b^ |
| --- | --- | --- | --- | --- |
| M0(one-ratio) | -18808.39 | ω=0.05196 | 938.672 (M3vsM0)** | Not allowed |
| M3(discrete) | -18339.05 | p_0_=0.24092 ω_0_=0.01454 |  | None |
|  |  | p_1_=0.48994 ω_1_=0.05271 |  |  |
|  |  | p_2_=0.26915 ω_2_=0.15366 |  |  |
| M7(beta) | -18331.91 | p=1.31970 q=16.90960 | 1.52 (M8vsM7) | Not allowed |
| M8(beta & ω) | -18331.15 | p_0_=0.98447 p=1.37516 |  | None |
|  |  | q=18.27504 p_1_=0.01553 |  |  |
|  |  | ω=13.54096 |  |  |
