# Supplementary material for: Unusual tandem expansion and positive selection in subgroups of the plant GRAS transcription factor superfamily
Source: BMC Plant Biol. 2014 Dec 19;14:373. doi: 10.1186/s12870-014-0373-5 (PMC4279901; doi:10.1186/s12870-014-0373-5)
Supplement: Additional file 23: — Parameters estimation and likelihood ratio tests for the site-specific model in Physcomitrella patens . Note: *p < 0.05 and **p < 0.01 (x 2 test). a ω was estimated under model M0,M3,M7, and M8; p and q are the parameters of the beta distribution. b The number of amino acid sites estimated to have undergone positive selection, and amino acids refer to Pp1s84_112V6 sequence. [file 12870_2014_373_MOESM23_ESM.doc]

**Additional file 23. Parameters estimation and likelihood ratio tests for the site-specific model in *Physcomitrella patens*.**

| Model | lnL | Estimate of  parameter ^a^ | 2ΔlnL | positive selection sites ^b^ |
| --- | --- | --- | --- | --- |
| M0(one-ratio) | -22962.4 | ω=0.10893 | 753.592  (M3vsM0)** | Not allowed |
| M3(discrete) | -22962.4 | p_0_=0.36931 ω_0_=0.03861 |  | None |
|  |  | p_1_=0.47678 ω_1_=0.12555 |  |  |
|  |  | p_2_=0.15391 ω_2_=0.26557 |  |  |
| M7(beta) | -22962.4 | p=1.52582 q=11.33732 | 4276.754 (M8vsM7)** | Not allowed |
| M8(beta & ω) | -22962.4 | p_0_=0.99999 p=0.80783 |  | **330K****, 333G*, **334R****, **340W****, **344E****, 345L*, **349T****, **372K****, **373E****, **427S****, 428H*, 437I*, **451N****, 453P*, 480V*, **481D****, **483E****, 489S*, **497V****, **499G****, 505N*, **510P****, **559A****, **562D****, **566S****, **636V****, 639I*, 644R*, **645Q****, 647N* |
|  |  | q=1.10818 p_1_=0.00001 |  |  |
|  |  | ω=2.74921 |  |  |
